# Bulky magnesium(ii) and sodium(i) bisphenoxide catalysts for chemoselective transesterification of methyl (meth)acrylates†

**DOI:** 10.1039/d2sc05413b

**Published:** 2022-11-28

**Authors:** Xue Zhao, Manussada Ratanasak, Kazumasa Kon, Jun-ya Hasegawa, Kazuaki Ishihara

**Affiliations:** a Graduate School of Engineering, Nagoya University B2-3(611) Furo-cho, Chikusa Nagoya 464-8603 Japan ishihara@cc.nagoya-u.ac.jp; b Section of Theoretical Catalytic Chemistry, Institute for Catalysis, Hokkaido University Sapporo Hokkaido 011-0021 Japan hesegawa@cat.hokudai.ac.jp; c Venture Business Laboratory, Nagoya University B2-4 Furo-cho, Chikusa Nagoya 464-0814 Japan

## Abstract

Given the industrial importance of (meth)acrylate esters, various groups have devoted considerable effort to investigating their chemoselective transesterification. In 2021, we developed magnesium(ii) and sodium(i) complexes derived from 2,6-di-*tert*-butyl-*p*-cresol (BHT-H) as chemoselective catalysts for the transesterification of methyl acrylate (MA) and methyl methacrylate (MMA), respectively. Based on our results, we report the discovery of magnesium(ii) and sodium(i) salts derived from 6,6′-(propane-2,2′-diyl)bis(2,4-di-*tert*-butylphenol) (PBTP-H_2_), *i.e.* Mg(PBTP) and Na_2_(PBTP), which are 41 and 81 times more effective catalysts than Mg(BHT)_2_ and Na(BHT) for the transesterification of MA and MMA, respectively. These new catalysts are highly effective across an extensive range of alcohols, including primary and secondary alcohols, diols, and triols. Overall, this efficient transesterification technology can be expected to find practical applications in industrial process chemistry.

## Abstract 2

Bulky Mg(ii) and Na(i) bisphenoxides have been developed as extremely effective catalysts for the chemoselective transesterification of methyl (meth)acrylates. Plausible mechanisms are proposed based on the results of XRD and DFT calculations.

## Graphical abstract

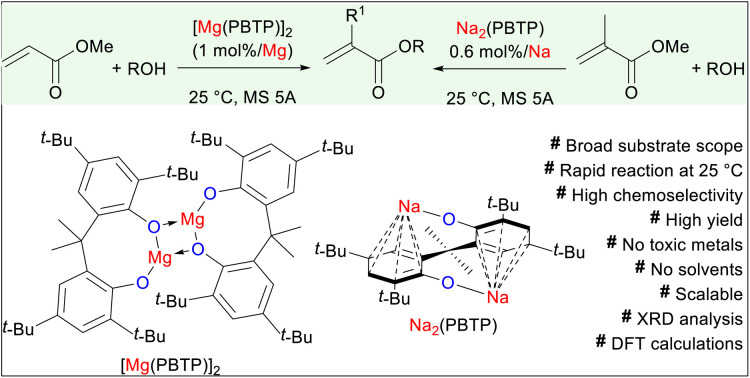

## Introduction

**Scheme 1:**
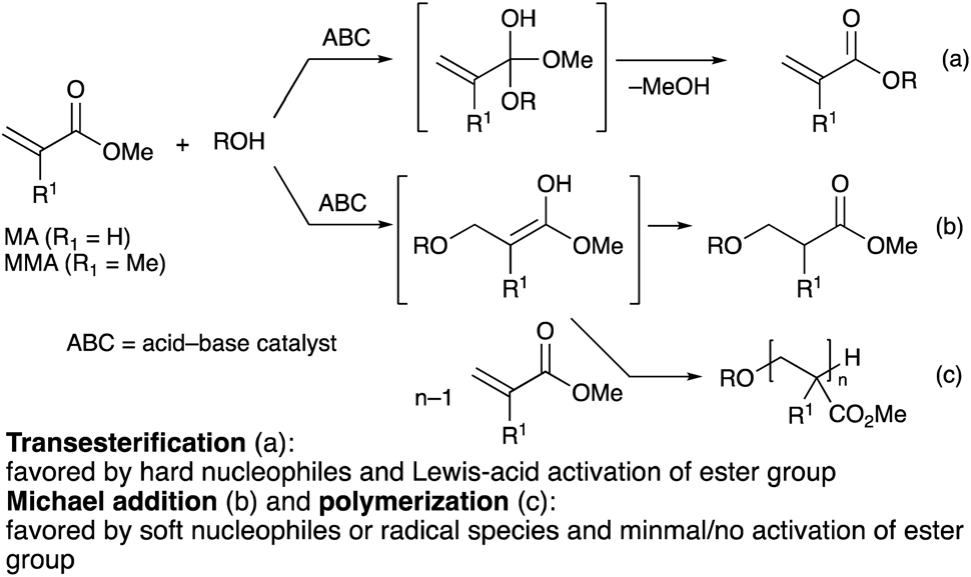
Nucleophilic reactions of alcohols to MA or MMA.

With industrial applications encompassing heat-resistant adhesives, varnishes, UV coatings, textile finishing, and polymeric plastics,^1^ (meth)acrylate esters are produced at the million-ton scale^2^ and are considered to be among the most important manufactured chemicals. The desirable physical properties of poly(meth)acrylate esters, such as their flexibility, transparency, and weatherability, can also be controlled and fine-tuned to the requirements of their intended applications, most commonly *via* the functionalization of the ester group, which adds to their versatility.^2^ While usually produced *via* the reaction of (meth)acryloyl chlorides with alcohols, the production of functionalized (meth)acrylates through a transesterification process^3–7^ instead would theoretically reduce the amount of stoichiometric halide wastes produced. The use of methyl (meth)acrylates (MA and MMA) instead of their corresponding carboxylic acids is also advantageous in terms of handling given their superior solubility profiles in organic solvents.

(Meth)acrylate esters are α,β-unsaturated esters capable of undergoing both Michael addition^8^ and transesterification.^3–7^ The extent of each of the two competing pathways depends on the character of the nucleophile and the mode of activation of the carbonyl group: nucleophiles with higher charge density, also known as “hard” nucleophiles, and carbonyl groups activated by a densely charged Lewis acid, such as that of a metal cation, tend to undergo nucleophilic substitution (Scheme 1a), whereas Michael addition is favored by “soft” nucleophiles that have better molecular orbital matching with the carbon–carbon double bond (Scheme 1b).^9^ As a result, a competent catalytic system for the transesterification of (meth)acrylate esters must exhibit excellent chemoselectivity toward the nucleophilic substitution reaction to maximize the transesterification product yield. This is especially difficult in the case of MA, which has the least sterically hindered α,β-unsaturated end in comparison to other acrylate analogues, and is therefore more prone to undergo the undesired Michael addition reaction. In fact, the harsh reaction conditions employed in many acid/base-related transesterification strategies invariably cause not only undesirable Michael addition reactions, but also the premature Michael-addition-initiated polymerization of MA or MMA (Scheme 1c), which results in low monomeric product yield.

Given the industrial significance of (meth)acrylates, it is hardly surprising that various groups have devoted considerable effort to the investigation of their chemoselective transesterification. In line with the aforementioned Lewis-acid activation strategy to favor transesterification, much attention has been paid to the application of heterogenous metal catalysts such as Ti(v),^10^ Zr(iv),^11^ and Ca(NO_3_)_2_/γ-Al_2_O_3_,^12^ to chemoselective transesterifications. However, the main concerns in all the afore-mentioned studies are the physical and catalytic properties of the heterogenous catalysts; the substrate scopes of the systems are underdeveloped and remains unexplored. It is unclear whether these catalysts can efficiently catalyze the transesterification of acrylates with other structurally distinct alcohols, such as complex primary alcohols, secondary alcohols, and diols.

In 2016, Ohshima accomplished the chemoselective transesterification of MMA and MA with a diverse array of primary alcohols, diols, and triols with excellent yield and selectivity using a zinc(ii) cluster catalyst,^6^ albeit that examples of secondary alcohols were lacking.^6*h*^ It should also be noted here that 20 mol% of toxic 4-(dimethylamino)pyridine (DMAP) is required as a ligand for this catalytic system to function optimally, which poses severe environmental concerns for its widespread adoption as a viable industrial solution. Thus, in 2017, Hashimoto and Ootsuka at Toagosei Co., Ltd. reported a new catalytic method using triethylenediamine (DABCO) and zinc(acrylate).^6*i*^ According to the patent,^6*i*^ Michael addition of DABCO to MMA gives an enolate anion, which is further transformed to a dimeric vinyl ester intermediate through transesterification with MMA. Subsequently the transesterification of this intermediate with primary alcohol occurs to give the corresponding ester and MMA.

In 2018, we developed tetramethylammonium methyl carbonate as an efficient, general and metal-free catalyst for transesterification reactions.^5*b*^ This catalyst has been successfully applied across a wide range of ester and alcohol partners, including examples using MMA as a substrate in reactions with secondary alcohols, diols, and triols; however, reactions using MA give complex mixtures.

It was evident that the chemical research community had made great headway in the development of catalysts capable of the efficient and chemoselective transesterification of (meth)acrylate esters. Nonetheless, a truly robust, economical, and environmentally friendly catalytic system capable of this feat remained elusive and warranted further research.

**Scheme 2:**
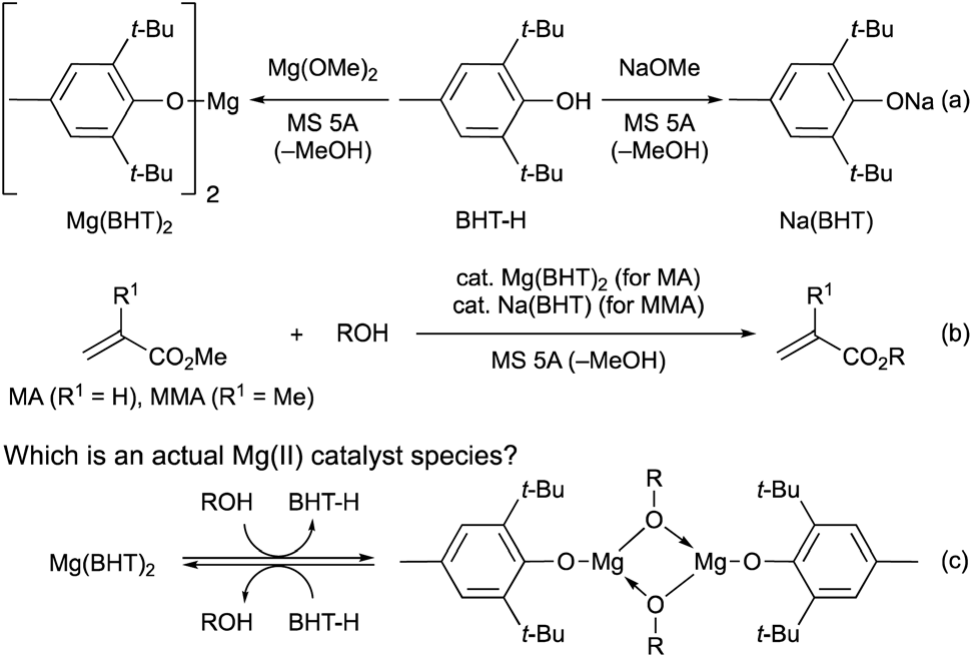
Transesterifications of MA and MMA catalyzed by Mg(BHT)_2_ and Na(BHT), respectively.

In 2021, we developed magnesium(ii) and sodium(i) complexes (Mg(BHT)_2_ and Na(BHT)) derived from 2,6-di-*tert*-butyl-*p*-cresol (BHT-H) as efficient and chemoselective catalysts for the transesterification of MA and MMA, respectively (Schemes 2a and b).^7^ These catalysts are effective for various primary and secondary alcohols, diols, triols, and even tetraols. Interestingly, dimeric Mg(ii) complexes, [Mg(OAr)(OR)]_2_, have been proposed as active species in the ring-opening polymerization (ROP) reactions.^13^ Therefore, we considered a mechanistic possibility including such dimeric Mg(ii) complexes based on DFT calculations (Scheme 2c) in our previous paper.^7^ The observed energy profiles showed that [Mg(OAr)(OR)]_2_ favors the transesterification pathway over the Michael-addition pathway. Additionally, according to the DFT results, the dimeric Mg(ii) complex (R = Bn) is by only 1.0 kcal mol^−1^ more stable than the monomeric Mg(ii) complex.

**Scheme 3:**
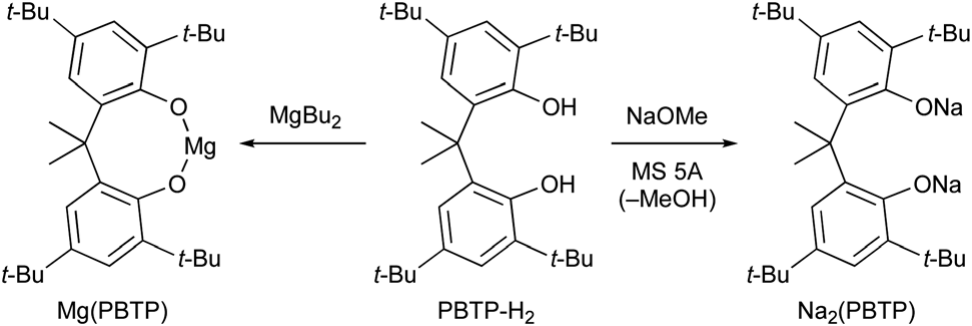
This work: new catalysts for chemoselective transesterification of MA and MMA.

Based on our previous study,^7^ we report herein the discovery of magnesium(ii) and sodium(i) salts derived from 6,6′-(propane-2,2′-diyl)bis(2,4-di-*tert*-butylphenol) (PBTP-H_2_), *i.e.*, Mg(PBTP) and Na_2_(PBTP), which serve as new catalysts for the transesterification of MA and MMA, respectively (Scheme 3). These catalysts are easily prepared from inexpensive chemicals, are much more active than Mg(BHT)_2_ and Na(BHT),^7^ and are effective across an extensive range of alcohols including primary and secondary alcohols as well as diols and triols. In general, this efficient transesterification technology can be expected to be attractive for practical applications in the context of industrial process chemistry.

## Results and discussion

Initially, the chemoselectivity of several magnesium(ii) aryloxides for the transesterification of MA with benzyl alcohol (1a) was estimated under equilibrium conditions without MS 5A. Representative results are shown in Table 1. When the *in situ*-generated Mg(BHT)_2_ was used as the catalyst, the desired product (2a) was obtained in 40% yield together with side product 6a (1% yield) from the competing Michael addition after 30 minutes (entry 1). Much to our delight, Mg(PBTP) was able to furnish 2a in 55% yield after 1 hour without any formation of undesirable side products (entry 2). Dibutylmagnesium also showed high catalytic activity, but exhibited a low level of chemoselectivity (entry 3). Side products 3a (1% yield), 4a (4% yield), and 5a (3% yield) were detected together with the formation of the desired product 2a (67% yield).

**Table 1:** Comparison of the chemoselectivity of catalysts for the transesterification of MA with 1a under equilibrium conditions^a^

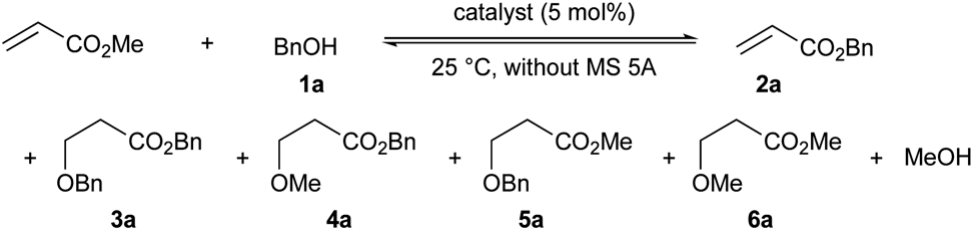				
Entry	Catalyst	Yield [%] of 2a/3a/4a/5a/6a^b^		
		0.5 h	1.0 h	3.0 h
1^c^	Mg(BHT)_2_	40/0/0/0/1	—	70/0/0/1/4
2	Mg(PBTP)	41/0/0/0/0	55/0/0/0/0	—
3	Bu_2_Mg	57/0/2/2/0	67/1/4/3/0	—

a Unless otherwise noted, the reaction was carried out using MA (14 mmol), 1a (2 mmol), catalyst (5 mol%), Cu(CS_2_NMe_2_)_2_ (polymerization inhibitor, 0.2 mol%), and MS 5A (0.4 g) at 25 °C.

b The yield was determined *via*^1^H NMR analysis using dimethylsulfone as an internal standard.

c Previous data from ref. 7.

Next, we attempted the transesterification of MA with isoborneol (1b) as a model reaction using magnesium(ii) aryloxides as catalysts, and monitored the reaction progress at various time intervals to compare their catalytic activity. The respective results are summarized in Table 2. We first tried Mg(BHT)_2_, and after 6, 8, and 12 hours, 2b was detected in 62%, 85%, and 91% yield, respectively; after 19 hours, 2b was obtained in 99% yield (entry 1). The use of Mg(PBTP) provided 2b in 90% and 99% yield after 6 and 8 hours, respectively (entry 2). A comparison of entry 1 with entry 2 suggests that the bidentate ligation of Mg(PBTP) plays an important role in increasing the catalytic activity. Based on this inspired discovery, we then examined Mg (TBP), which was generated *in situ* from Bu_2_Mg and 3,3′,5,5′-*tert*-butyl-1,1′-biiphenyl-2,2′-diol (TBP-H_2_). However, only 35% and 42% yields of 2b were detected after 6 and 8 hours, respectively; after 24 hours the yield of 2b reached just 60% (entry 3). Interestingly, the ring size of the metalacyclic structure, in addition to the bidentate ligation of the magnesium(ii) aryloxides, is also important for increasing the catalytic activity.

**Table 2:** Comparison of catalytic activities for the transesterification of MA with 1b^a^

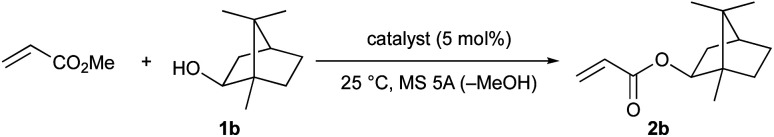						
Entry	Catalyst	Yield [%] of 2b^b^				
		6 h	8 h	12 h	19 h	24 h
1	Mg(BHT)_2_	62	85	91	99	—
2	Mg(PBTP)	90	99	—	—	—
3	Mg (TBP)^c^	35	42	—	—	60

a Unless otherwise noted, the reaction was carried out using MA (14 mmol), 1b (2 mmol), catalyst (5 mol%), Cu(CS_2_NMe_2_)_2_ (polymerization inhibitor, 0.2 mol%), and MS 5A (0.4 g) at 25 °C.

b The yield was determined *via*^1^H NMR analysis using dimethylsulfone as an internal standard.

c TBP-H_2_ and Mg (TBP) are shown as follows.


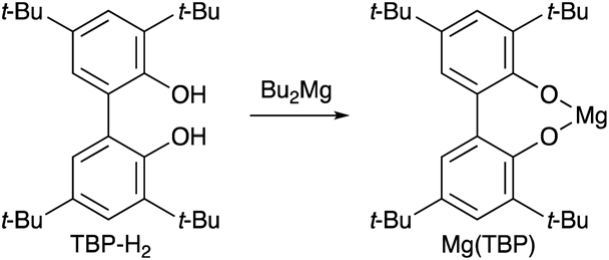
Next, the substrate scope of Mg(PBTP) as a catalyst for the transesterification of MA was investigated as shown in Table 3. Mg(PBTP) was more active than Mg(BHT)_2_, and the transesterification was completed much faster. The transesterification proceeded with primary alcohols such as a saturated fatty alcohol (1c), allylic alcohols (1e, 1f), and (hetero)arylmethyl alcohols (1a, 1d) to give the desired esters in quantitative yield. We were able to scale up the test reaction by a factor of five to obtain 1.62 g of 2a (>99% yield, 4 h) using a lowered catalytic load of 1 mol% Mg(PBTP), demonstrating the viability of the catalytic system for the gram-scale synthesis.

**Table 3:** Substrate scope of Mg(PBTP) as a catalyst for the transesterification of MA^a^

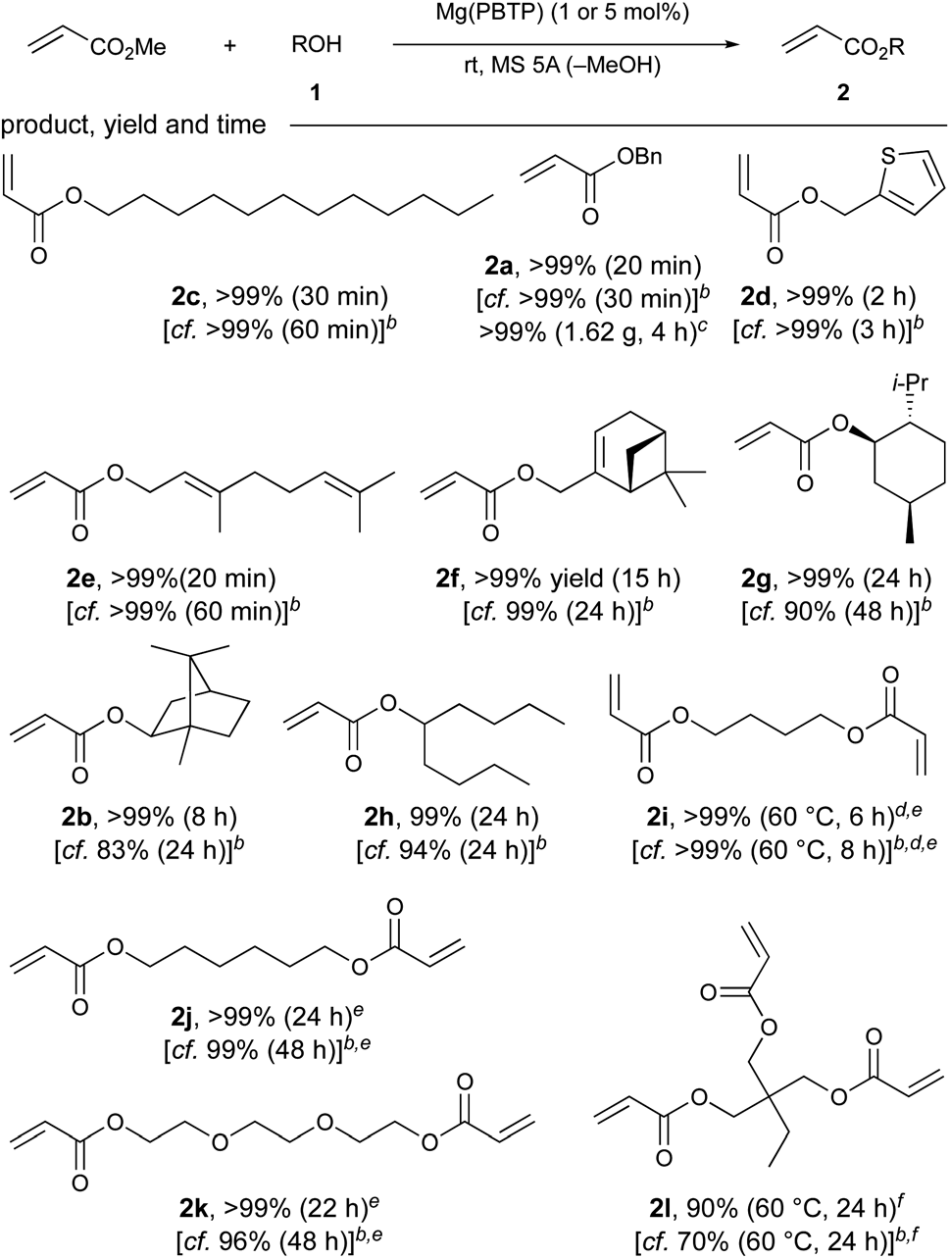

a Unless otherwise noted, the reaction was carried out using MA (14 mmol), 1 (2 mmol), Mg(PBTP) (5 mol%), Cu(CS_2_NMe_2_)_2_ (polymerization inhibitor, 0.2 mol%), and MS 5A (0.4 g) at 25 °C. Isolated yields after flash column chromatography on silica gel are shown.

b Mg(BHT)_2_ was used instead of Mg(PBTP) under otherwise identical conditions. See ref. 7.

c The reaction was carried out using MA (70 mmol), 1a (10 mmol), Mg(PBTP) (1 mol%), Cu(CS_2_NMe_2_)_2_ (polymerization inhibitor, 0.2 mol%), and MS 5A (2 g) at 25 °C.

d Mg(PBTP) (10 mol%) was used.

e MS 5A (0.6 g) was used.

f MS 5A (0.8 g) was used.

Additionally, Mg(PBTP) was applicable to secondary alcohols, although longer reaction times were required. For example, (−)-menthyl acrylate (2g), which is potentially useful for the synthesis of pressure-sensitive adhesives (PSA),^14^ and isoborneol-derived acrylate 2b were obtained in >99% yield. 5-Nonanyl acrylate (2h), a monomer for synthesis that is a common additive for UV-adhesives, was also synthesized successfully.^15^

Trimethylolpropane acrylate 2l, which is commonly used in the production of UV coatings and has also recently found applications in the development of superswelling hydrogels^16^ and as an alumina pigment modifier,^5*c*^ was successfully obtained in 90% yield after 24 hours. This result represents a successful chemoselective transesterification of an industrially useful triol. We increased the amount of 5A molecular sieves to 800 mg to account for the three-fold increase in equivalents of the side product methanol produced per molecule of target product. The transesterification of various industrially useful diols (1i–1k) to produce 2i–2k was also successful with extraordinary yields (>99%) and reaction times of 6–24 hours. Once again, we used a higher amount of 5 Å molecular sieves (600 mg) for these diols to facilitate the removal of methanol.

Next, we focused on the chemoselective transesterification of MMA. As expected, Mg(PBTP), as well as Mg(BHT)_2_, were less effective for MMA. Fortunately, Na_2_(PBTP) (1.25 mol%) was highly effective and superior to Na(BHT) (2.5 mol%), as shown in Table 4. Under the standard reaction conditions, all the primary alcohols (1c, 1e, 1a) gave the corresponding products in quantitative yield within 10 minutes. Glycidyl methacrylate (7m), which is chelating and particularly susceptible to nucleophilic attack, which sometimes results in its decomposition and polymerization, was obtained in 87% yield.^5*b*–*d*^ Trifluoroethyl methacrylate (7n), which is also unstable due to its electron-withdrawing nature was also obtained in 93% yield.^17^ Quinine methacrylate (7p), a potentially useful monomer for the synthesis of molecularly imprinted polymers for the screening of halides and amino acids,^18^ was successfully obtained in quantitative yield despite its notable steric demand. Similarly, other *sec*-alkyl methacrylates such as 7b, 7g, 7h, and 7o were successfully synthesized. The efficacy of Na_2_(PBTP) was highlighted by the facile transesterification of diols to furnish diesters 7r, 7i, and 7k quantitatively in a shorter time. In addition to these successful results, transesterification of trimethylolpropane (1l) quantitatively furnished triester 7l when an increased catalyst loading of Na_2_(PBTP) (2.5 mol%) was used.

**Table 4:** Substrate scope of Na_2_(PBTP) as a catalyst for the transesterification of MMA^a^

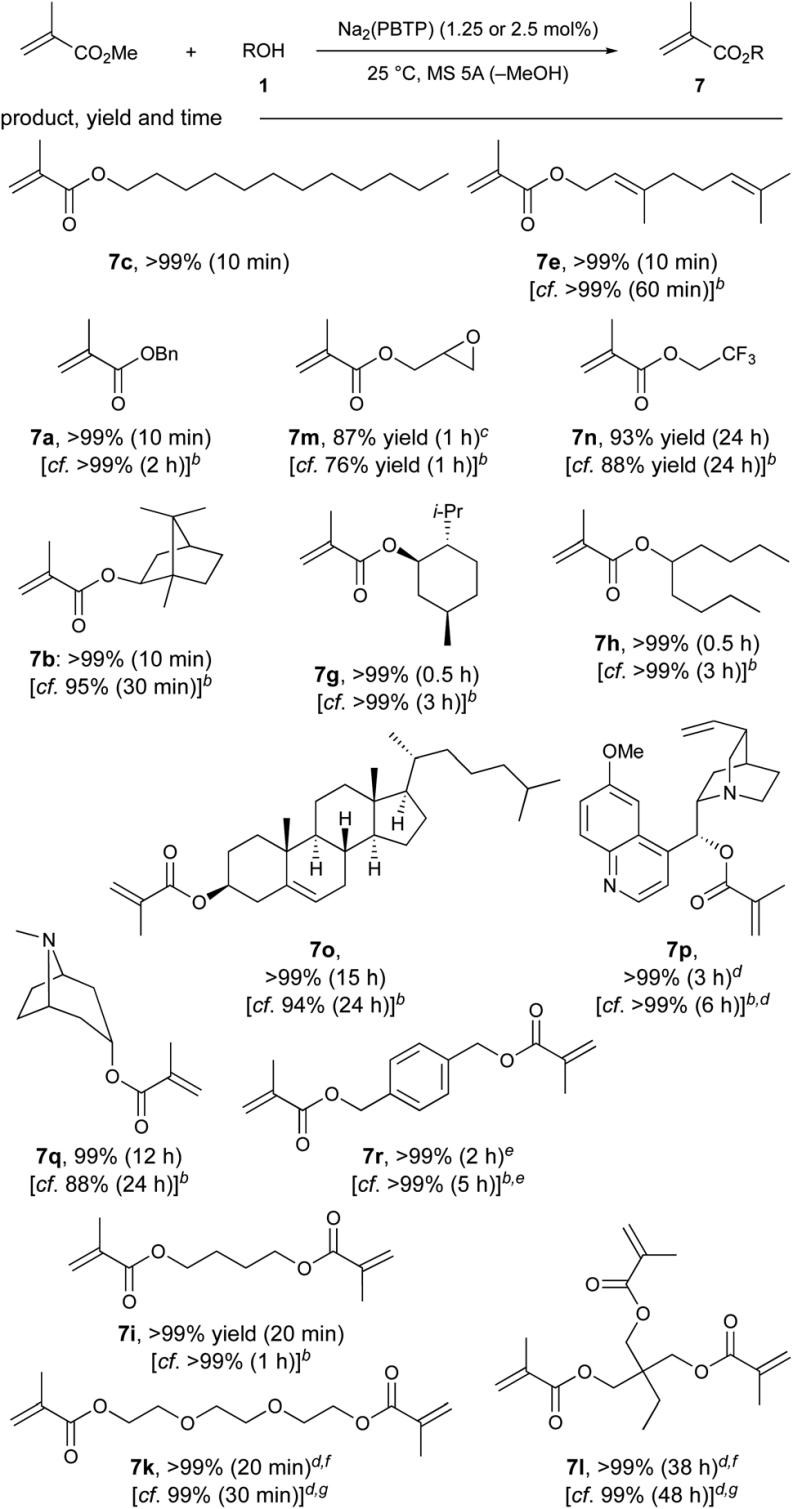

a Unless otherwise noted, the reaction was carried out with MMA (14 mmol), 1 (2 mmol), Na_2_(PBTP) (1.25 mol%), 4-acetamido-TEMPO (polymerization inhibitor, 0.1 mol%), and MS 5A (0.4 g) at 25 °C. Isolated yields after flash column chromatography on silica gel are shown.

b Na(BHT) (2.5 mol%) was used instead of Na_2_(PBTP) (1.25 mol%) under otherwise identical conditions. See ref. 7.

c MS 5A (0.8 g) was used.

d MMA (28 mmol) and MS 5A (0.8 g) were used.

e MS 5A (0.6 g) was used.

f Na_2_(PBTP) (2.5 mol%) was used.

g Na(BHT) (5 mol%) was used.

**Fig. 1:**
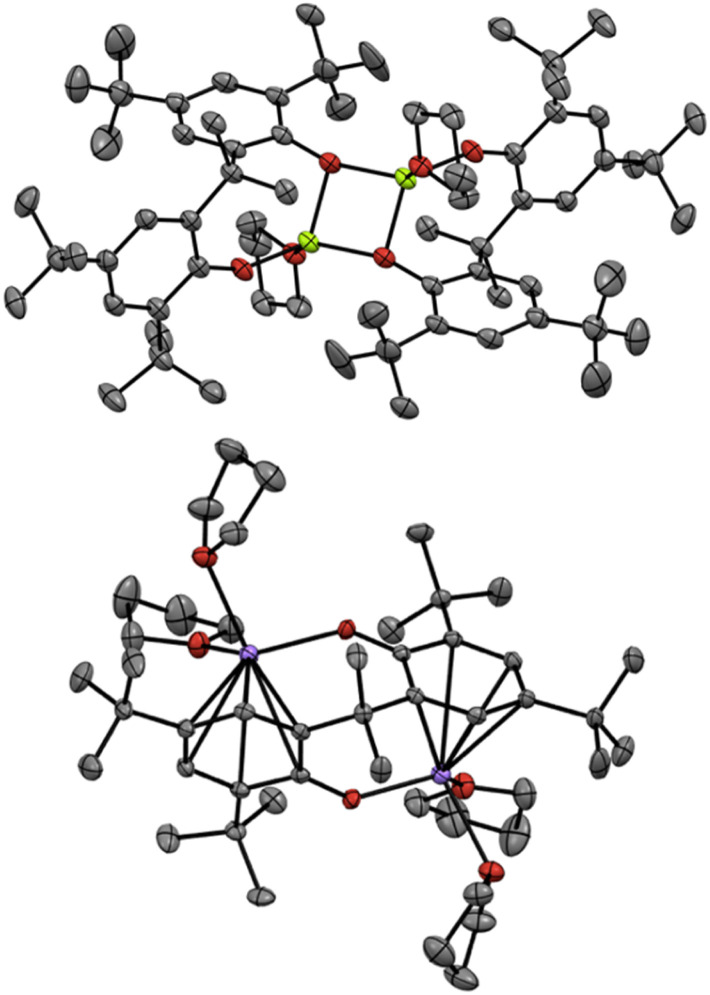
Molecular structures of [Mg(PBTP)·THF]_2_ (top) and Na_2_(PBTP)·4(THF) (bottom) with thermal displacement ellipsoids at 50% probability; Hydrogen atoms are omitted for clarity.

Finally, we turned our attention to the mechanistic aspects. The molecular structures of Mg(PBTP) and Na_2_(PBTP) were determined *via* single-crystal X-ray diffraction analysis (Fig. 1). Interestingly, Mg(PBTP) was crystallized in THF as the dimeric complex [Mg(PBTP)·THF]_2_. On the other hand, Na_2_(PBTP) was crystallized in THF as the monomeric complex Na_2_(PBTP)·4(THF), which was stabilized through a double sodium(i)-η^6^-benzene structure. Under the transesterification conditions, MA, MMA, and 1 coordinative to [Mg(PBTP)]_2_ and Na_2_(PBTP) to replace the THF molecules.

**Fig. 2:**
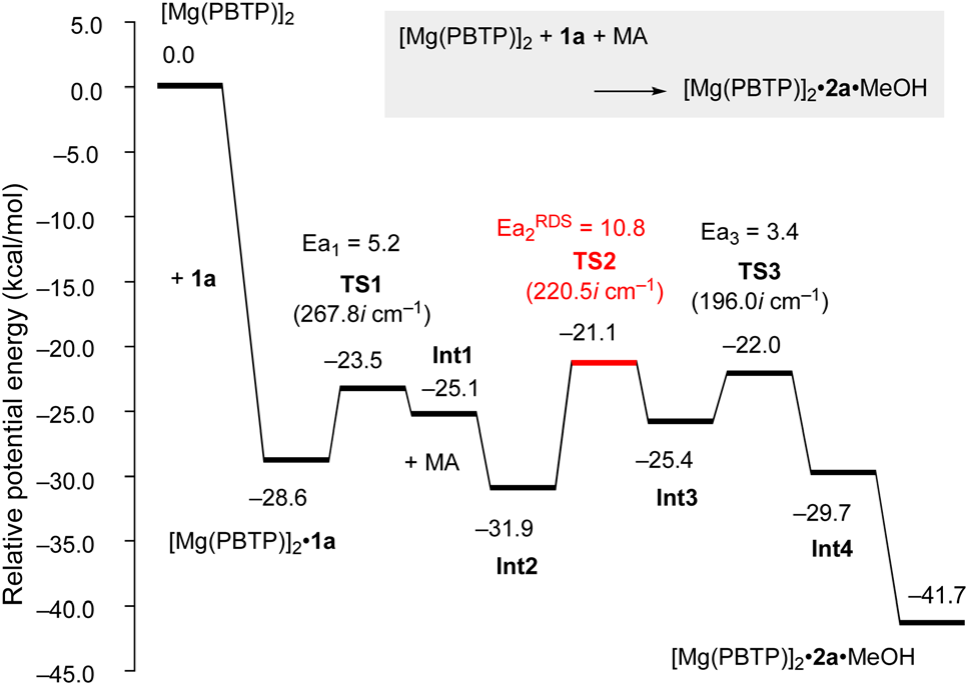
Potential energy profile for the transesterification of MA with 1a using [Mg(PBTP)]_2_. Energies are given in kcal mol^−1^. For transition states, imaginary frequencies are given in parentheses. For computational details, see ref. 19.

**Fig. 3:**
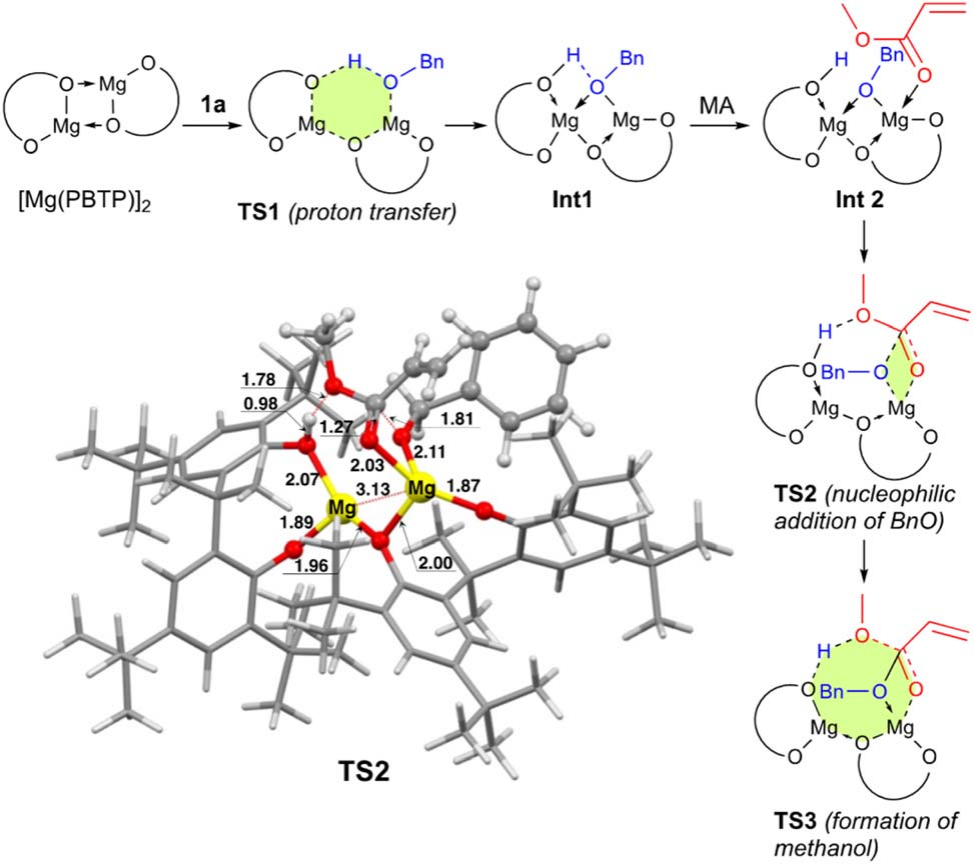
Optimized structure of TS2. Bond lengths are given in Å. Selected transition state and intermediate structures based on Fig. 2 are also shown. For computational details, see ref. 19.

To numerically verify the higher catalytic activity of Mg(PBTP) compared to that of Mg(BHT)_2_, density functional theory (DFT) calculations were performed for the transesterification of MA with 1a catalyzed by [Mg(PBTP)]_2_ based on its crystal structure shown in Fig. 1.^19^ The potential energy profile is explained in Fig. 2 and 3. The pathway shown is the most plausible in the present study. [Mg(PBTP)]_2_ is stabilized by the coordination of 1a. 1a is then activated as int1 by intramolecular proton transfer in [Mg(PBTP)]_2_·1a*via*TS1. In addition, MA is also activated as Int2 by coordination of Int1. Nucleophilic addition of BnO^−^ to MA in Int2 then occurs *via*TS2 to form Int3. This is the rate-determining step (RDS), and the calculated activation energy is 10.8 kcal mol^−1^ (*E*^EDS^_a2_). Thus, this elementary step includes both proton transfer from 1a to OAr and C–O formation in a stepwise manner. Subsequently, methanol is released from Int3*via*TS3. As shown in Table 5, the *E*^EDS^_a2_ value using [Mg(PBTP)]_2_ is 2.2–5.2 kcal mol^−1^ lower than those using Mg(PBTP), Mg(BHT)_2_, and [Mg(BHT)(OBn)]_2_. In particular, the *E*^EDS^_a_ difference (2.2 kcal mol^−1^) between [Mg(PBTP)]_2_ and [Mg(BHT)(OBn)]_2_ enhances the rate constant of RDS for the former, which is by a factor of 41 higher than that of the latter.^20^ This estimation provides a reasonable interpretation for the experimental activity enhancement of [Mg(PBTP)]_2_ as shown in Table 3.
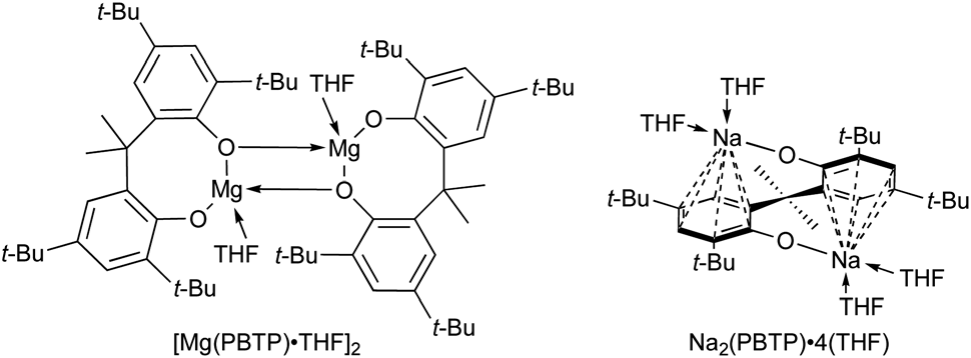


**Table 5:** Comparison of the activation energy for the rate determining step^a^

Catalyst	*E* ^RDS^_a_ [kcal mol^−1^]	Catalyst	*E* ^RDS^_a_ [kcal mol^−1^]
Mg(BHT)_2_^b^^,^^c^	14.9	Mg(PBTP)^b^	16.0
[Mg(BHT)(OBn)]_2_^b^^,^^c^	13.0	[Mg(PBTP)]_2_^b^	10.8
Na(BHT)^c^^,^^d^	21.4	Na_2_(PBTP)^d^	18.8

a For computational details, see ref. 19.

b Calculated for the transesterification of MA with 1a.

c See ref. 7.

d Calculated for transesterification of MMA with 1a.

Next, DFT calculations were performed for the transesterification of MMA with 1a catalyzed by Na_2_(PBTP) based on its crystal structure shown in Fig. 1. In this case, 1a and MMA are activated at the same time by coordination of Na_2_(PBTP). Subsequently both proton transfer from 1a to OAr and nucleophilic addition of BnO^−^ occur *via*TS4 to give Int5 in a concerted manner. This is the RDS, and the calculated activation energy is 18.8 kcal mol^−1^ (*E*^EDS^_a_). As shown in Table 5, the *E*^EDS^_a_ value using Na_2_(PBTP) is 2.6 kcal mol^−1^ lower than that using Na(BHT). This difference in *E*^EDS^_a_ enhances the rate constant of RDS for Na_2_(PBTP), which is by a factor of 81 higher than that for Na(BHT).^20^ This estimation provides a reasonable interpretation for the experimental activity enhancement of Na_2_(PBTP) shown in Table 4.

**Fig. 4:**
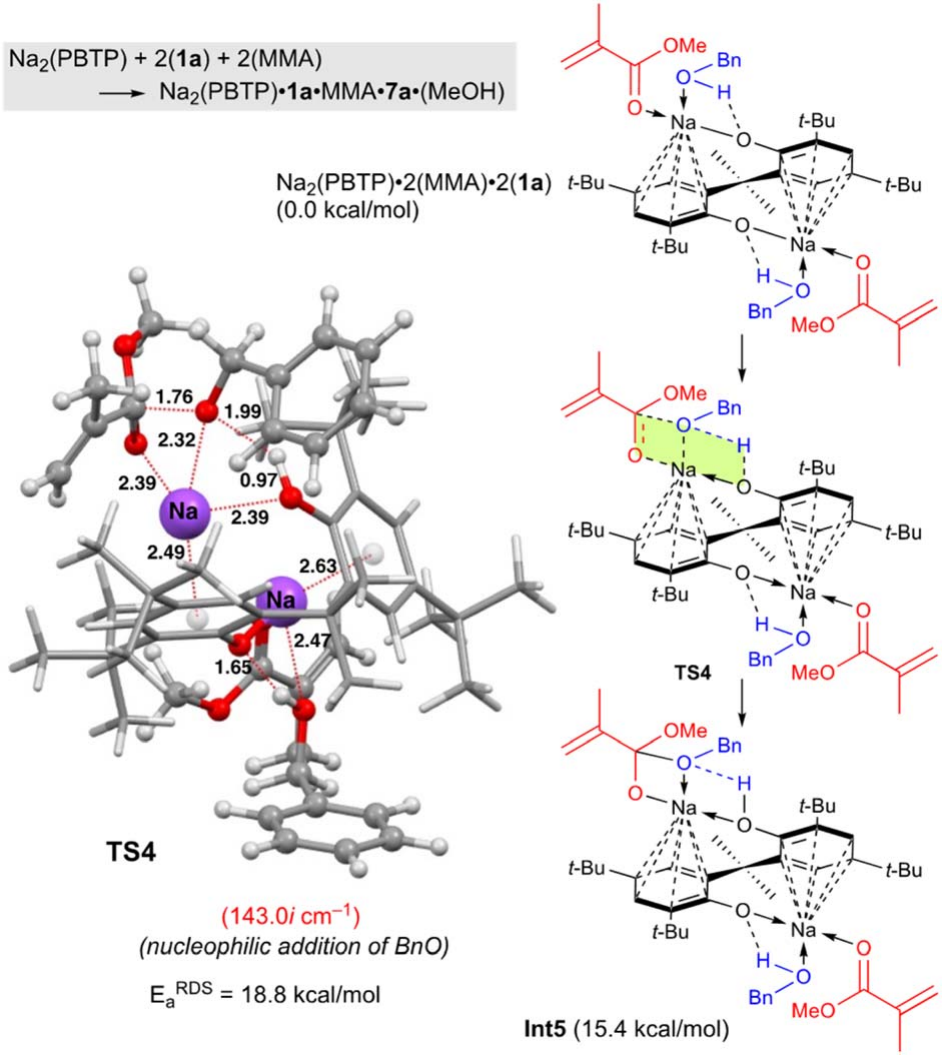
Optimized structure of TS4 for the transesterification of MMA with BnOH using Na_2_(PBTP). Bond lengths are given in Å. Selected transition state and intermediate structures are also shown. For computational details, see ref. 19.

Since Na_2_(PBTP) has two Na sites, another possibility is sequential transesterification at the other Na site. This indicates one Na site has MMA and 1a and ready for the reaction. The other Na site is coordinated by the product of the reaction. In fact, this structure corresponds to Int5 in Fig. 4. The Int5 state is 15.4 kcal mol^−1^ higher than the reactant state, Na_2_(PBTP)·2(MMA)·2(1a). This result indicates that 7a and MeOH are predominantly produced from Int 5, MMA, and 1a, and Na_2_(PBTP)·2(MMA)·2(1a) is regenerated at the same time. Thus, the possibility of the sequential transesterification at the other Na site of Int 5 is ruled out.

## Conclusions

In summary, we have developed the catalysts [Mg(PBTP)]_2_ and Na_2_(PBTP), which are highly effective for the chemoselective transesterification of MA and MMA, respectively, under mild conditions at 25 °C. These catalysts are superior to Mg(BHT)_2_ and Na(BHT), respectively, which we had previously developed.^7^ The results of DFT calculations strongly support our experimental results. Overall, based on the observed chemoselectivity, high yields, mild conditions, and lack of toxic metal species, the catalytic methods reported here represent new practical, green, and sustainable catalyst candidates for the industrial synthesis of acrylates.

## Data availability

The data supporting this study is available within the main text and the associated ESI.†

## Author contributions

K. I. conceived and directed the project. X. Z. and K. K. carried out the experiments and collected data. M. R. performed and analyzed the DFT calculations under the supervision of J. H. K. I. wrote the manuscript with contributions from all authors.

## Conflicts of interest

There are no conflicts to declare.

## Supplementary Material

SC-014-D2SC05413B-s001

SC-014-D2SC05413B-s002
