# Supplementary material for: Bulky magnesium(ii) and sodium(i) bisphenoxide catalysts for chemoselective transesterification of methyl (meth)acrylates
Source: Chem Sci. 2022 Nov 28;14(3):566–72. doi: 10.1039/d2sc05413b (PMC9847673; doi:10.1039/d2sc05413b)

Electronic Supplementary Material (ESI) for Chemical Science.
This journal is © The Royal Society of Chemistry 2022

Bulky Magnesium(II) and Sodium(I) Bisphenoxide Catalysts for the Chemoselective Transesterification of Methyl (Meth)acrylates

Xue Zhao,[a] Manussada Ratanasak,[b] Kazumasa Kon,[a,c] and Jun-ya Hasegawa*[b] and Kazuaki Ishihara*[a]

[a] Graduate School of Engineering, Nagoya University, B2-3(611) Furo-cho, Chikusa, Nagoya 464-8603 (Japan)
E-mail: ishihara@cc.nagoya-u.ac.jp

[b] Section of Theoretical Catalytic Chemistry, Institute for Catalysis, Hokkaido University, Sapporo, Hokkaido 011-0021 (Japan)
E-mail: hasegawa@cat.hokudai.ac.jp

[c] Venture Business Laboratory, Nagoya University, B2-4 Furo-cho, Chikusa, Nagoya 464-0814 (Japan)

Abstract: Given the industrial importance of (meth)acrylate esters, various groups have devoted considerable effort to investigating their chemoselective transesterification. In 2021, we developed magnesium(II) and sodium(I) complexes derived from 2,6-di-*tert*-butyl-*p*-cresol (BHT-H) as chemoselective catalysts for the transesterification of methyl acrylate (MA) and methyl methacrylate (MMA), respectively. Based on our results, we report the discovery of magnesium(II) and sodium(I) salts derived from 6,6'-(propane-2,2'-diyl)bis(2,4-di-*tert*-butylphenol (PBTP-H_2), i.e., Mg(PBTP), and Na_2(PBTP), which are 41 and 81 times more effective catalysts than Mg$(BHT)_2$ and Na(BHT) for the transesterification of MA and MMA, respectively. These new catalysts are highly effective across an extensive range of alcohols, including primary and secondary alcohols, diols, and triols. Overall, this efficient transesterification technology can be expected to find practical applications in industrial process chemistry.

Table of Contents

1. General Methods S2
2. Synthesis of 6,6'-(Propane-2,2-diyl)bis(2,4-di-*tert*-butylphenol)[1] S2
3. Preparation of metal aryloxides S3
4. Products in Tables 1 and 3 S3
5. Representative procedure for the transesterification of methyl acrylate (MA) catalyzed by Mg(PBTP) complex (Tables 2 and 3) S4
6. Representative procedure for the transesterification of methyl methacrylate (MMA) using Na_2(PBTP) complex (Table 4) S5
7. X-ray diffraction analysis of Mg(PBTP) and Na_2(PBTP) S8
8. DFT calculations for potential energy profile including Mg(I) and Na(I) species S10
9. References S28
Appendix ^{1}H, ^{13}N, ^{19}F NMR spectra S29

1. General methods

^{1}H NMR spectra were measured on a JEOL ECS400 (400 MHz) spectrometer at ambient temperature. Data were recorded as follows: chemical shift in ppm from internal tetramethylsilane on the δ scale, multiplicity (s = singlet; d = doublet; t = triplet; q = quartet, m = multiplet, br = broad), coupling constant (Hz), integration, and assignment. ^{13}C NMR spectra were measured on a JEOL ECS400 (100 MHz) spectrometer. Chemical shifts were recorded in ppm from the solvent resonance employed as the internal standard (deuterochloroform at 77.00 ppm). ^{19}F NMR spectra were measured on a JEOL ECS-400 (376 MHz) spectrometer. Chemical shifts were recorded in ppm from the solvent resonance employed as the external standard ($CFCl_3$ at 0 ppm). The products were purified by column chromatography on silica gel (E. Merck Art. 9385; Kanto Chemical Co., Inc. 37560). High resolution mass spectral analyses were performed at Chemical Instrument Center, Nagoya University (JEOL JMS-700 (FAB), JEOL JMS-T100GCV (EI), JEOL JMS-T100TD (DART-MS), Bruker Daltonics micrOTOF-QII (ESI)). Infrared (IR) spectra were recorded on a JASCO FT/IR 460 plus spectrometer. Melting points were measured on MPA100, Standard Research Systems. For thin-layer chromatography (TLC) analysis throughout this work, Merck precoated TLC plates (silica gel 60GF254 0.25 mm) were used. Visualization was accomplished by UV light (254 nm), anisaldehyde, $KMnO_4$, and phosphomolybdic acid. Anhydrous compounds, such as methyl acrylate (MA), methyl methacrylate (MMA), and methanol, were used as received commercially.

2. Synthesis of 6,6'-(Propane-2,2-diyl)bis(2,4-di-*tert*-butylphenol)[1]

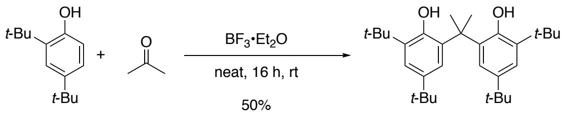

Boron trifluoride diethyl etherate (0.6 mL, 5.0 mmol, 1 equiv) was slowly added to a solution of 2,4-di-*tert*- butylphenol (2.1 g, 10.0 mmol, 2 equiv) in anhydrous acetone (368 μL, 5.0 mmol, 1 equiv) at room temperature under nitrogen. The reaction mixture was stirred at room temperature for 16 h. Methanol at 0 °C was added and stirred for 1 h. The resulting suspension was filtered, and the crude product was washed with methanol at 0 °C. The crude product was dissolved by chloroform, then purified by silica gel column chromatography (eluent: *n*-hexane:EtOAc = 8:1) to give the desired product.

6,6'-(Propane-2,2-diyl)bis(2,4-di-*tert*-butylphenol): White solid; ^{1}H NMR (400 MHz, $CDCl_3$): δ 1.28 (s, 18H, $2C(CH_3)_3$) 1.36 (s, 18H, $2C(CH_3)_3$), 1.73 (s, 6H, $2CH_3$), 4.99 (s, 2H, 2OH), 7.30 (d, J = 2.28 Hz, 2H, 2ArH), 7.40 (d, J = 2.28 Hz, 2H, 2ArH); ^{13}C NMR (100 MHz, $CDCl_3$): δ 29.0 (2C) 29.7 (6C), 31.9 (6C), 34.8 (2C), 35.3 (2C), 40.6 (1C),119.9 (2C), 124.2 (2C), 130.8 (2C), 137.6 (2C), 143.1 (2C), 151.9 (2C).

3. Preparation of metal aryloxides

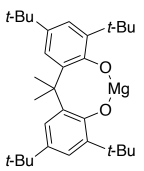

Ma(PBTP): Under nitrogen atmosphere conditions, commercially available dibutylmagnesium (1.0 *M* in heptane)(100 μL, 0.10 mmol, 1.0 equiv) was slowly added to a solution of 6,6´-(propane-2,2-diyl)bis(2,4-di-*tert*-butylphenol) (PBTP-H_2, 45.2 mg, 0.10 mmol, 1.0 equiv) in dehydrated THF (200 μL) at room temperature. The reaction mixture was stirred at room temperature for 30 minutes. Volatiles were removed, and the residue was desiccated at room temperature under reduced pressure (<5 Torr) for 3 h. The resultant compound (>99% yield) as a white solid with a little pale yellow used for reaction directly. ^{1}H NMR (400 MHz, $CDCl_3$) δ 1.28 (s, 18H) 1.36 (s, 18H), 1.73 (s, 6H), 7.30 (d, *J* = 2.28 Hz, 2H, ArH), 7.40 (d, *J* = 2.28 Hz, 2H). ^{13}C NMR (100 MHz, $CDCl_3$) δ 29.0 (2C) 29.7 (6C), 31.9 (6C), 34.8 (2C), 35.3 (2C), 40.5, 119.9 (2C), 124.2 (2C), 130.7 (2C), 137.5 (2C), 143.1 (2C), 151.8 (2C).

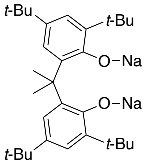

Na_2(PBTP): Under nitrogen atmosphere conditions, commercially available sodium methoxide (5.0 *M* in methanol) (10 μL, 0.05 mmol, 2 equiv) was slowly added to a solution of 6,6'-(propane-2,2-diyl)bis(2,4-di-*tert*-butylphenol) (PBTP-H_2, 11.3 mg, 0.025 mmol, 1 eq) in dehydrated THF (140 μL) at room temperature. The reaction mixture was stirred at room temperature for 30 min. Volatiles were removed, and the residue was desiccated at room temperature under reduced pressure (<5 Torr) for 3 h. The resultant compound (>99% yield) as a pale yellow solid used for reaction directly. ^{1}H NMR (400 MHz, $CDCl_3$) δ 1.28 (s, 18H), 1.36 (s, 18H), 1.73 (s, 6H), 7.30 (d, *J* = 2.28 Hz, 2H), 7.41 (d, *J* = 2.28 Hz, 2H); ^{13}C NMR (100 MHz, $CDCl_3$) δ 29.0 (2C), 29.7 (6C), 31.9 (6C), 34.8 (2C), 35.3 (2C), 40.5, 119.8 (2C), 124.2 (2C), 130.6 (2C), 137.5 (2C), 143.1 (2C), 151.8 (2C).

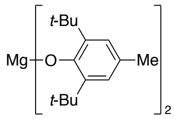

$Mg(BHT)_2$:[2] A mixture of commercially available magnesium ethoxide (229 mg, 2.0 mmol) and 2,6-di-*tert*-butyl-4-methylphenol (BHT-H, 881 mg, 4.0 mmol) in methanol (5 mL) was stirred at room temperature for 30 min. Volatiles were removed, and the residue was desiccated at room temperature under reduced pressure (<5 Torr) for 3 h. The resultant compound (>99% yield) as a white solid was stored in vial tube at room temperature. M.p. 101-104 °C (decomposition).^{1}H NMR (400 MHz, benzene-*d*$_6$) δ 1.38 (s, 36H), 2.25 (s, 6H), 7.06 (s, 4H); ^{13}C NMR (100 MHz, benzene-*d*$_6$) δ 21.5 (2C), 30.5 (12C), 34.4 (4C), 125.9 (4C), 128.5 (2C), 136.0 (4C), 152.1 (2C). IR (KBr) 3626, 2956, 2871, 1433, 1396, 1362, 1230, 1214, 1150, 1120 cm^{-1}.

4. Products in Tables 1 and 3

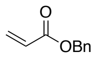

Benzyl acrylate (2a):[2] Colorless oil. ^{1}H NMR (400 MHz, $CDCl_3$) δ 5.19 (s, 2H), 5.84 (dd, *J* = 10.4, 1.6 Hz, 1H), 6.16 (dd, *J* = 17.4, 10.5 Hz, 1H), 6.44 (dd, *J* = 17.2, 1.4 Hz, 1H), 7.31-7.41 (m, 5H). ^{13}C NMR (100 MHz, $CDCl_3$) δ 66.4, 128.3 (2C), 128.4 (2C), 128.7 (2C), 131.2, 135.9, 166.1. IR (neat) 3034, 2954, 1953, 1725, 1634, 1455, 1406, 1295, 1269, 1186, 1049 cm^{-1}. HRMS (FAB+) calcd for $C_{10}H_{10}O_2$ [M]$^+$ 162.0681, found 162.0680.

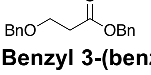

Benzyl 3-(benzyloxy)propanoate (3a):[2] Colorless oil. ^{1}H NMR (400 MHz, $CDCl_3$) δ 2.66 (t, *J* = 6.4 Hz, 2H), 3.76 (t, *J* = 6.4 Hz, 2H), 4.51 (s, 2H), 5.14 (s, 2H), 7.25-7.40 (m, 10H). ^{13}C NMR (100 MHz, $CDCl_3$) δ 35.3, 65.6, 66.4 73.2, 127.7 (2C), 128.2 (4C), 128.4 (2C), 128.6 (2C), 135.9, 138.1, 171.5. IR (neat) 3032, 2868, 1737, 1496, 1455, 1363, 1258, 1173, 1105, 1071, 1027 cm^{-1}. HRMS (FAB+) calcd for $C_{17}H_{18}O_3$ [M]$^+$ 270.1256, found 270.1254.

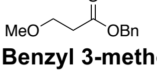

Benzyl 3-methoxypropanoate (4a):[2] Colorless oil. ^{1}H NMR (400 MHz, $CDCl_3$) δ 2.62 (t, *J* = 6.4 Hz, 2H), 3.34 (s, 3H), 3.67 (t, *J* = 6.4 Hz, 2H), 5.13 (s, 2H), 7.30-7.40 (m, 5H). ^{13}C NMR (100 MHz, $CDCl_3$) δ 35.1, 58.9, 66.4, 68.0, 128.3 (2C), 128.4, 128.6 (2C), 135.9, 171.5. IR (neat) 2893, 1738, 1455, 1391, 1350, 1169, 1118, 1070, 1015 cm^{-1}. HRMS (FAB+) calcd for $C_{11}H_{14}O_3$ [M]$^+$ 194.0943, found 194.0948.

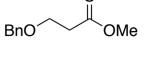

Methyl 3-(benzyloxy)propanoate (5a):[2] Colorless oil. ^{1}H NMR (400 MHz, $CDCl_3$) δ 2.62 (t, *J* = 6.4 Hz, 2H), 3.69 (s, 3H), 3.75 (t, *J* = 6.4 Hz, 2H), 4.53 (s, 2H), 7.26-7.40 (m, 5H). ^{13}C NMR (100 MHz, $CDCl_3$) δ 35.1, 51.8, 65.6, 73.2, 127.8 (3C), 128.5 (2C), 138.1, 172.2. IR (neat) 2868, 1741, 1437, 1366, 1195, 1176, 1105, 1073 cm^{-1}. HRMS (FAB+) calcd for $C_{11}H_{14}O_3$ $[M]^+$ 194.0943, found 194.09452.

5. Representative procedure for the transesterification of methyl acrylate (MA) catalyzed by Mg(PBTP) complex (Tables 2 and 3)

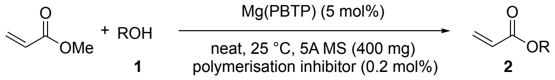

To a mixture of *in situ*-generated Mg(PBTP) (47.5 mg, 0.10 mmol), activated 5A molecular sieves (powder, 400 mg), copper(II) dimethyldithiocarbamate (1.2 mg, 0.0040 mmol) as a polymerization inhibitor, and dimethyl sulfone (18.8 mg, 0.20 mmol) as an internal standard for the crude ^{1}H NMR analysis, methyl acrylate (MA, 1.26 mL, 14 mmol) was added at 25 °C. After 1 min, alcohol (2.0 mmol) was added to the mixture at 25 °C. The mixture was stirred at room temperature for 20 min–38 h, and the reaction was monitored by TLC. After the reaction was completed, by using a drop of the mixture, the rough yield was determined by ^{1}H NMR ($CDCl_3$) analysis based on dimethyl sulfone as an internal standard.

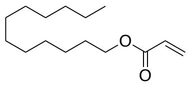

Dodecyl acrylate (2c):[2] Colorless oil. ^{1}H NMR (400 MHz, $CDCl_3$) δ 0.88 (t, *J* = 6.4 Hz, 3H), 1.26-1.38 (m, 18H), 1.63-1.70 (m, 2H), 4.15 (t, *J* = 6.4 Hz, 2H), 5.81 (dd, *J* = 10.5, 1.4 Hz, 1H), 6.12 (dd, *J* = 17.4, 10.0 Hz, 1H), 6.40 (dd, *J* = 17.4, 1.4 Hz, 1H); ^{13}C NMR (100 MHz, $CDCl_3$) δ 14.3, 22.8, 26.1, 28.8, 29.4, 29.5, 29.6, 29.7, 29.8 (2C), 32.1, 64.9, 128.8, 130.6, 166.5; IR (neat) 2925, 2854, 1729, 1636, 1467, 1407, 1295, 1272, 1192, 1059 cm^{-1}. HRMS (DART+) calcd for $C_{15}H_{29}O_2$ $[M+H]^+$ 241.2168, found 241.2165.

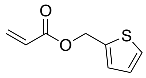

Thiophen-2-ylmethyl acrylate (2d):[2] Colorless oil. ^{1}H NMR (400 MHz, $CDCl_3$) δ 5.35 (s, 2H), 5.85 (dd, *J* = 10.6, 1.4 Hz, 1H), 6.14 (dd, *J* = 17.4, 10.5 Hz, 1H), 6.45 (dd, *J* = 17.4, 1.4 Hz, 1H), 6.99 (dd, *J* = 5.0, 3.2 Hz, 1H), 7.12 (d, *J* = 3.7 Hz, 1H), 7.33 (dd, *J* = 5.0, 1.4, Hz 1H); ^{13}C NMR (100 MHz, $CDCl_3$) δ 60.7, 126.9, 127.0, 128.2, 128.4, 131.5, 137.9, 165.9; IR (neat) 3108, 2954, 1725, 1634, 1441, 1407, 1295, 1261, 1183 cm^{-1}. HRMS (DART+) calcd for $C_8H_8O_2S$ $[M]^+$ 168.0245, found 168.0246.

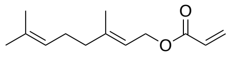

Geranyl acrylate (2e):[2] Colorless oil. ^{1}H NMR (400 MHz, $CDCl_3$) δ 1.60 (s, 3H), 1.68 (s, 3H), 1.72 (s, 3H), 2.03-2.14 (m, 4H), 4.68 (d, *J* = 7.4 Hz, 2H), 5.08 (m, 1H), 5.38 (m, 1H), 5.82 (dd, *J* = 10.5, 1.4 Hz, 1H), 6.13 (dd, *J* = 17.0, 10.0 Hz, 1H), 6.41 (dd, *J* = 17.4, 1.4 Hz, 1H); ^{13}C NMR (100 MHz, $CDCl_3$) δ 16.6, 17.8, 25.8, 26.4, 39.7, 61.6, 118.3, 123.8, 128.7, 130.7, 132.0, 142.5, 166.4; IR (neat) 2968, 2925, 2857, 1725, 1636, 1445, 1407, 1378, 1294, 1270, 1184, 1045 cm^{-1}. HRMS (DART+) calcd for $C_{13}H_{21}O_2$ $[M+H]^+$ 209.1542, found 209.1540.

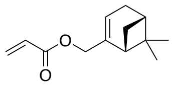

(1*R*)-(–)-Myrtenyl acrylate (2f):[2] Colorless oil. ^{1}H NMR (400 MHz, $CDCl_3$) δ 0.83 (s, 3H), 1.19 (d, *J* = 8.7 Hz, 1H), 1.29 (s, 3H), 2.08-2.16 (m, 2H), 2.25 (dm, *J* = 17.8 Hz, 1H), 2.32 (dm, *J* = 17.8 Hz, 1H), 2.41 (dt, *J* = 8.7, 5.8 Hz, 1H), 4.19-4.59 (m, 2H), 5.59 (m, 1H), 5.82 (dd, *J* = 10.1, 1.4 Hz, 1H), 6.12 (dd, *J* = 17.4, 10.5 Hz, 1H), 6.40 (dd, *J* = 17.0, 1.4 Hz, 1H); ^{13}C NMR (100 MHz, $CDCl_3$) δ 21.2, 26.2, 31.4, 31.6, 38.2, 40.8, 43.7, 67.2, 121.7, 128.7, 130.7, 143.0, 166.2; IR (neat) 2987, 2918, 2833, 1728, 1636, 1619, 1406, 1366, 1294, 1268, 1184, 1045 cm^{-1}. HRMS (DART+) calcd for $C_{13}H_{19}O_2$ [M+H]+ 207.1385, found 207.1381.

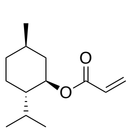

L-Menthyl acrylate (2g):[2] Colorless oil. ^{1}H NMR (400 MHz, $CDCl_3$) δ 0.77 (d, *J* = 6.9 Hz, 3H), 0.85-0.92 (m, 1H), 0.89 (d, *J* = 6.9 Hz, 3H), 0.91 (d, *J* = 6.9 Hz, 3H), 0.96-1.14 (m, 2H), 1.38-1.56 (m, 2H), 1.66-1.72 (m, 2H), 1.87 (m, 1H), 2.03 (m, 1H), 4.76 (td, *J* = 10.6, 4.1 Hz, 1H), 5.80 (dd, *J* = 10.1, 1.4 Hz, 1H), 6.11 (dd, *J* = 17.4, 10.1 Hz, 1H), 6.39 (dd, *J* = 17.4, 1.4 Hz, 1H); ^{13}C NMR (100 MHz, $CDCl_3$) δ 16.5, 20.9, 22.2, 23.6, 26.4, 31.5, 34.4, 41.0, 47.2, 74.5, 129.2, 130.4, 166.0; IR (neat) 2956, 2871, 1723, 1633, 1456, 1405, 1296, 1270, 1199, 1046 cm^{-1}. HRMS (DART+) calcd for $C_{13}H_{23}O_2$ $[M+H]^+$ 211.1698, found 211.1700.

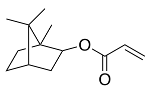

Isobornyl acrylate (2b):[2] Colorless oil. ^{1}H NMR (400 MHz, $CDCl_3$) δ 0.85 (d, *J* = 3.2 Hz, 6H), 1.01 (s, 3H), 1.07-1.21 (m, 2H), 1.57 (m, 1H), 1.67-1.87 (m, 4H), 4.75 (dd, *J* = 7.3, 3.7 Hz, 1H), 5.79 (dd, *J* = 10.5, 1.4 Hz, 1H), 6.09 (dd, *J* = 17.4, 10.5 Hz, 1H), 6.34 (dd, *J* = 17.4, 1.4 Hz, 1H); ^{13}C NMR (100 MHz, $CDCl_3$) δ 11.5, 19.9, 20.2, 27.1, 33.8, 38.8, 45.1, 47.0, 48.9, 81.2, 129.3, 130.0, 165.8; IR (neat) 2956, 2879, 1720, 1636, 1619, 1455, 1406, 1390, 1296, 1276, 1199, 1109 cm^{-1}. HRMS (ESI+) calcd for $C_{13}H_{20}NaO_2$ [M+Na]$^+$ 231.1356, found 231.1353.

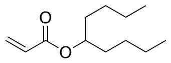

Nonan-5-yl acrylate (2h):[2] Colorless oil. ^{1}H NMR (400 MHz, $CDCl_3$) δ 0.88 (m, 6H), 1.28 (m, 8H), 1.56 (m, 4H), 4.95 (m, 1H), 5.80 (dd, *J* = 10.4, 1.3 Hz, 1H), 6.11 (dd, *J* = 17.2, 10.5 Hz, 1H), 6.38 (dd, *J* = 17.2, 1.84 Hz, 1H); ^{13}C NMR (100 MHz, $CDCl_3$) δ 14.1 (2C), 22.7 (2C), 27.6 (2C), 33.9 (2C), 74.7, 129.1, 130.3, 166.2; IR (neat) 2957, 2861, 1724, 1404, 1196 cm^{-1}. HRMS (FAB$^+$) calcd for $C_{12}H_{23}O_2$ [M+H]$^+$199.16980, found 199.17063.

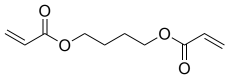

Butane-1,4-diyl diacrylate (2i):[2] Colorless liquid. ^{1}H NMR (400 MHz, $CDCl_3$) δ) 1.75–1.82 (m, 4H), 4.19–4.22 (m, 4H), 5.83 (dd, *J* = 1.4, 10.6 Hz, 2H), 6.12 (ddd, *J* = 1.8, 10.6, 17.4 Hz, 2H), 6.41 (dd, *J* = 1.4, 17.4 Hz, 2H); ^{13}C NMR (100 MHz, $CDCl_3$) δ 18.4(2C), 25.5(2C), 64.3(2C), 125.5(2C), 136.5(2C), 167.5(2C); IR (neat) 2970, 1730, 1636, 1620, 1468, 1410, 1297, 1272, 1186, 1061 cm^{-1}. HRMS (DART+) calcd for $C_{10}H_{15}O_4$ [M+H]$^+$ 199.0970, found 199.0966.

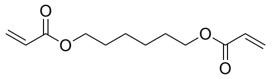

hexane-1,6-diyl diacrylate (2j):[2] Colorless liquid. ^{1}H NMR (400 MHz, $CDCl_3$) δ 1.41-1.44 (m, 4H), 1.66-1.73 (m, 4H), 4.16 (t, *J* = 6.4 Hz, 4H), 5.82 (dd, *J* = 1.4, 10.1 Hz, 2H), 6.12 (dd, *J* = 10.5, 17.4 Hz, 2H), 6.40 (dd, *J* = 1.4, 17.4 Hz, 2H); ^{13}C NMR (100 MHz, $CDCl_3$) δ 25.7(2C), 28.6(2C), 64.6(2C), 128.6(2C), 130.7(2C), 166.4(2C); IR (neat) 2941, 2862, 1718, 1636, 1620, 1467, 1409, 1274, 1197 cm^{-1}. HRMS (DART+) calcd for $C_{12}H_{19}O_4$ [M+H]$^+$ 227.1283, found 227.1284.

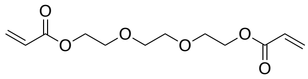

(Ethane-1,2-diylbis(oxy))bis(ethane-2,1-diyl) diacrylate (2k):[2] Colorless oil. ^{1}H NMR (400 MHz, $CDCl_3$) δ 3.68 (s, 4H), 3.74-3.76 (m, 4H), 4.31-4.33 (m, 4H), 5.84 (dd, *J* = 10.6, 1.4 Hz, 2H), 6.16 (dd, *J* = 17.4, 10.6 Hz, 2H), 6.43 (dd, *J* = 17.4, 1.4 Hz, 2H). ^{13}C NMR (100 MHz, $CDCl_3$) δ 63.8 (2C), 69.3 (2C), 70.7 (2C), 128.4 (2C), 131.2 (2C), 166.3 (2C). IR (neat) 2952, 2875, 1725, 1636, 1619, 1454, 1409, 1352, 1298, 1197, 1131, 1067 cm^{-1}. HRMS (DART+) calcd for $C_{12}H_{19}O_6$ [M+H]$^+$ 259.1182, found 259.1189.

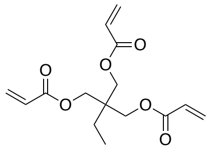

2-((Acryloyloxy)methyl)-2-ethylpropane-1,3-diyl diacrylate (2l):[2] Colorless oil. ^{1}H NMR (400 MHz, $CDCl_3$) δ 0.93 (t, *J* = 7.8 Hz, 3H), 1.56 (q, *J* = 6.8 Hz, 2H), 4.18 (s, 6H), 5.86 (d, *J* = 10.6 Hz, 3H), 6.11 (dd, *J* = 17.0, 10.0 Hz, 3H), 6.41 (d, *J* = 17.4 Hz, 3H); ^{13}C NMR (100 MHz, $CDCl_3$) δ 7.6, 23.3, 41.0, 64.3 (3C), 128.1 (3C), 131.5 (3C), 166.0 (3C); IR (neat) 2970, 1725, 1635, 1619, 1467, 1408, 1270, 1182, 1061 cm^{-1}. HRMS (DART+) calcd for $C_{15}H_{21}O_6$ [M+H]$^+$ 297.1338, found 297.1340.

6. Representative procedure for the transesterification of methyl methacrylate (MMA) using Na_2(PBTP) complex (Table 4)

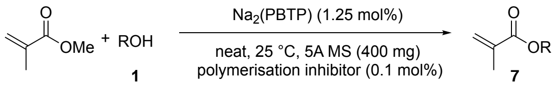

To a mixture of *in situ*-generated Na_2(PBTP) (12.4 mg, 0.025 mmol), activated 5A molecular sieves (powder, 400 mg), 4-acetamido-2,2,6,6-tetramethylpiperidine 1-oxyl (0.42 mg, 0.0020 mmol) as a polymerization inhibitor, and dimethyl sulfone (18.8 mg, 0.20 mmol) as an internal standard for the crude ^{1}H NMR analysis, methyl methacrylate (MMA, 1.49 mL, 14 mmol) was added at 25 °C. After 1 min, **1** (2.0 mmol) was added to the mixture at 25 °C. The mixture was stirred at room temperature for 10 min–38 h, and the reaction was monitored by TLC. After the reaction was completed, by using a drop of the mixture, the rough yield was determined by ^{1}H NMR ($CDCl_3$) analysis based on dimethyl sulfone as an internal standard.

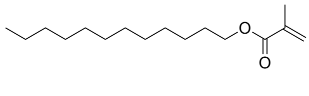

Dodecyl methacrylate (7c):[2] Colorless liquid. ^{1}H NMR (400 MHz, $CDCl_3$) δ 0.88 (t, *J* = 6.9 Hz, 3H), 1.26-1.39 (m, 18H), 1.63-1.70 (m, 2H), 1.95 (s, 3H), 4.14 (t, *J* = 6.9 Hz, 2H), 5.55 (m, 1H), 6.10 (s, 1H); ^{13}C NMR (100 MHz, $CDCl_3$) δ 14.3, 18.5, 22.8, 26.1, 28.7, 29.4, 29.5, 29.7 (2C), 29.8 (2C), 32.1, 65.0, 125.3, 136.7, 167.6; IR (neat) 2921, 2854, 1719, 1638, 1467, 1321, 1296, 1165 cm^{-1}. HRMS (FAB+) calcd for $C_{16}H_{31}O_2$ [M+H]$^+$ 255.2324, found 255.2324.

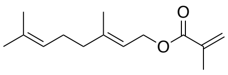

Geranyl methacrylate (7e):[2] Colorless oil. ^{1}H NMR (400 MHz, $CDCl_3$) δ 1.60 (s, 3H), 1.68 (s, 3H), 1.72 (s, 3H), 1.94-1.96 (m, 3H), 2.03-2.14 (m, 4H), 4.67 (d, *J* = 7.3 Hz, 2H), 5.08 (tm, *J* = 6.9 Hz, 1H), 5.38 (tm, *J* = 6.9 Hz, 1H), 5.55 (m, 1H), 6.10 (m, 1H); ^{13}C NMR (100 MHz, $CDCl_3$) δ 16.6, 17.8, 18.5, 25.8, 26.4, 39.6, 61.8, 118.6, 123.9, 125.4, 131.9, 136.7, 142.1, 167.7; IR (neat) 2926, 1719, 1638, 1451, 1377, 1313, 1293, 1162, 1010 cm^{-1}. HRMS (DART+) calcd for $C_{14}H_{23}O_2$ [M+H]$^+$ 223.1698, found 223.1696.

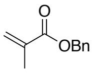

Benzyl methacrylate (7a):[2] Colorless oil. ^{1}H NMR (400 MHz, $CDCl_3$) δ 1.97 (s, 3H), 5.20 (s, 2H), 5.59 (s, 1H), 6.16 (s, 1H), 7.30-7.40 (m, 5H); ^{13}C NMR (100 MHz, $CDCl_3$) δ 18.5, 66.5, 125.9, 128.1 (2C), 128.2, 128.6 (2C), 136.2, 136.3, 167.3; IR (neat) 3034, 2957, 1719, 1637, 1454, 1319, 1294, 1159 cm^{-1}. HRMS (DART+) calcd for $C_{11}H_{13}O_2$ [M+H]$^+$ 177.0916, found 177.0912.

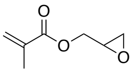

Oxiran-2-ylmethyl methacrylate (7m):[2] Colorless oil. ^{1}H NMR (400 MHz, $CDCl_3$) δ 1.97 (s, 3H), 2.68 (dd, *J* = 4.6, 2.5 Hz, 1H), 2.87 (dd, *J* = 4.6, 4.1 Hz, 1H), 3.26 (m, 1H), 4.01 (dd, *J* = 6.0, 12.4 Hz, 1H), 4.49 (dd, *J* = 3.2, 12.4 Hz, 1H), 5.62 (m, 1H), 6.17 (s, 1H); ^{13}C NMR (100 MHz, $CDCl_3$) δ 18.4, 44.7, 49.5, 65.2, 126.3, 135.9, 167.1; IR (neat) 3002, 2958, 2930, 1722, 1637, 1454, 1349, 1317, 1296, 1170 cm^{-1}. HRMS (DART+) calcd for C7H11O3 [M+H]+ 143.0708, found 143.0707.

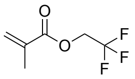

2,2,2-Trifluoromethyl methacrylate (7n):[2] Colorless oil. ^{1}H NMR (400 MHz, $CDCl_3$) δ 1.99 (s, 3H), 4.54 (q, *J* = 7.8 Hz, 2H), 5.71 (s, 1H), 6.23 (s, 1H); ^{13}C NMR (100 MHz, $CDCl_3$) δ 18.2, 60.7 (q, *J* = 36.2 Hz), 123.2 (q, *J* = 275 Hz), 127.8, 134.9, 165.7; ^{19}F NMR (376 MHz, $CDCl_3$) δ –73.8; IR (neat) 2974, 1758, 1284, 1183, 1150 cm^{-1}. HRMS (DART+) calcd for $C_6H_8F_3O_2$ [M+H]$^+$ 169.0476, found 169.0469.

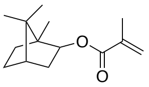

Isobornyl methacrylate (7b):[2] Colorless oil. ^{1}H NMR (400 MHz, $CDCl_3$) δ 0.85 (s, 3H), 0.86 (s, 3H), 1.02 (s, 3H), 1.07-1.21 (m, 2H), 1.57 (m, 1H), 1.67-1.88 (m, 4H), 1.93 (s, 3H), 4.71 (m, 1H), 5.52 (d, J = 1.4 Hz, 1H), 6.07 (s, 1H); ^{13}C NMR (100 MHz, CDCl3) d 11.5, 18.4, 19.9, 20.1, 27.1, 33.7, 38.9, 45.1, 47.0, 48.9, 81.2, 125.0, 136.9, 166.9. IR (neat) 2955, 2879, 1717, 1638, 1455, 1327, 1298, 1163, 1054 cm^{-1}. HRMS (FAB+) calcd for $C_{14}H_{22}NaO_2$ [M+Na]$^+$ 245.1517, found 245.1509.

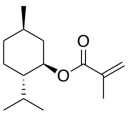

L-Menthyl methacrylate (7g):[2] Colorless oil. ^{1}H NMR (400 MHz, $CDCl_3$) δ 0.77 (d, *J* = 6.9 Hz, 3H), 0.82-0.93 (m, 1H), 0.89 (d, *J* = 6.4 Hz, 3H), 0.91 (d, *J* = 6.4 Hz, 3H), 0.95-1.14 (m, 2H), 1.40-1.58 (m, 2H), 1.66-1.73 (m, 2H), 1.88 (m, 1H), 1.94 (m, 3H), 2.03 (m, 1H), 4.73 (td, *J* = 11.0, 4.1 Hz, 1H), 5.53 (m, 1H), 6.08 (m, 1H); ^{13}C NMR (100 MHz, $CDCl_3$) δ 16.6, 18.6, 20.9, 22.2, 23.7, 26.5, 31.5, 34.4, 41.0, 47.3, 74.6, 125.1, 137.0, 167.1; IR (neat) 2956, 2871, 1715, 1638, 1456, 1316, 1297, 1171 cm^{-1}. HRMS (DART+) calcd for $C_{14}H_{25}O_2$ [M+H]$^+$ 225.1855, found 225.1856.

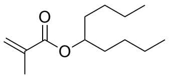

Nonan-5-yl methacrylate 7h):[2] Colorless oil. ^{1}H NMR (400 MHz, $CDCl_3$) d 0.89 (t, *J* = 6.9 Hz, 6H), 1.23–1.37 (m, 8H), 1.53–1.62 (m, 4H), 1.95 (s, 3H), 4.93 (m, 1H), 5.53 (m, 1H), 6.09 (m, 1H); ^{13}C NMR (100 MHz, $CDCl_3$) d 14.2 (2C), 18.6, 22.8 (2C), 27.6 (2C), 33.9 (2C), 74.8 124.9, 137.0, 167.4; IR (neat) 2958, 2932, 2862, 1717, 1638, 1456, 1319, 1296, 1170 cm^{-1}. HRMS (DART+) calcd for $C_{13}H_{25}O_2$ [M+H]$^+$ 213.1855, found 213.1853.

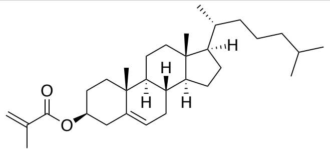

Cholesteryl methacrylate (7o):[2] White solid. M.p. 112-114 °C; ^{1}H NMR (400 MHz, $CDCl_3$) δ 0.68 (s, 3H), 0.86 (d, J = 6.6 Hz, 3H), 0.87 (d, J = 6.6 Hz, 3H), 0.92 (d, J = 6.4 Hz, 3H), 0.95-1.69 (m, 24H), 1.77-2.04 (m, 8H), 2.36 (d, J = 8.2 Hz, 2H), 4.67 (m, 1H), 5.38 (d, J = 4.6 Hz, 1H), 5.53 (m, 1H), 6.08 (s, 1H); ^{13}C NMR (100 MHz, $CDCl_3$) δ 12.0, 18.5, 18.9, 19.5, 21.2, 22.7, 23.0, 24.0, 24.4, 27.9, 28.2, 28.4, 32.0, 32.1, 36.0, 36.3, 36.8, 37.2, 38.3, 39.7, 39.9, 42.5, 50.2, 56.3, 56.8, 74.4, 122.8, 125.1, 137.0, 139.9, 167.0; IR (KBr) 2947, 1718, 1638, 1467, 1375, 1324, 1295, 1171, 1012 cm^{-1}. HRMS (EI) calcd for $C_{31}H_{50}O_2$ $[M]^+$ 454.3811, found 454.3818.

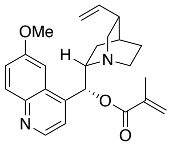

Quinine methacrylate (7p):[2] White solid. M.p. 124-129 °C; ^{1}H NMR (400 MHz, $CDCl_3$) δ 1.50-1.65 (m, 2H), 1.72 (m, 1H), 1.83-1.91 (m, 2H), 1.98 (s, 3H), 2.28 (m, 1H), 2.58-2.73 (m, 2H), 3.06 (dd, J = 13.7, 10.1 Hz, 1H), 3.15 (m, 1H), 3.41 (q, J = 7.8 Hz, 1H), 3.96 (s, 3H), 4.98-5.03 (m, 2H), 5.66 (m, 1H), 5.83 (m, 1H), 6.23 (s, 1H), 6.34 (d, J = 6.9 Hz, 1H), 7.34 (d, J = 4.6 Hz, 1H), 7.34 (dd, J = 9.2, 2.8 Hz, 1H), 7.44 (d, J = 2.3 Hz, 1H), 8.02 (d, J = 9.2 Hz, 1H), 8.73 (d, J = 4.6 Hz, 1H); ^{13}C NMR (100 MHz, $CDCl_3$) δ 18.5, 24.3, 27.7, 28.0, 39.8, 42.6, 55.7, 56.8, 59.4, 74.5, 101.5, 114.6, 118.8, 121.9, 126.5, 127.0, 131.9, 136.2, 141.9, 143.8, 144.9, 147.6, 158.0, 166.4; IR (KBr) 2925, 1715, 1622, 1594, 1508, 1469, 1446, 1375, 1324, 1303, 1230, 1156 cm^{-1}. HRMS (DART+) calcd for $C_{24}H_{29}N_2O_3$ $[M+H]^+$ 393.2178, found 393.2187.

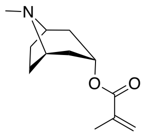

Tropinyl methacrylate (7q):[2] Colorless oil. ^{1}H NMR (400 MHz, $CDCl_3$) δ 1.74 (d, J = 14.7 Hz, 2H), 1.92-2.06 (m, 4H), 1.97 (s, 3H), 2.15 (dt, J = 14.7, 4.4 Hz, 2H), 2.29 (s, 3H), 3.20-3.05 (m, 2H), 5.06 (t, J = 5.5 Hz, 1H), 5.57 (t, J = 1.6 Hz, 1H), 6.09 (s, 1H); ^{13}C NMR (100 MHz,$CDCl_3$) δ 18.2, 25.5(2C), 36.5(2C), 40.3(2C), 59.6, 67.5, 125.0, 136.7, 166.5.; IR (neat) 2943, 1715, 1448, 1316, 1296, 1170, 1063, 1036 cm^{-1}. HRMS (FAB+) calcd for $C_{12}H_{20}NO_2$ $[M+H]^+$ 210.1494, found 210.1485.

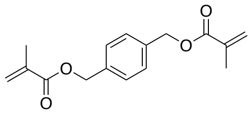

1,4-Phenylenebis(methylene) bis(2-methylacrylate) (7r):[2] White solid. M.p. 68-70 ºC; ^{1}H NMR (400 MHz, $CDCl_3$) δ 1.97 (s, 6H), 5.20 (s, 4H), 5.59-5.60 (m, 2H), 6.16 (s, 2H), 7.38 (s, 4H); ^{13}C NMR (100 MHz, $CDCl_3$) δ 18.5 (2C), 66.2 (2C), 126.1 (2C), 128.4 (4C), 136.2 (2C), 136.3 (2C), 167.3 (2C); IR (neat) 3040, 2966, 2931, 1701, 1635, 1456, 1373, 1319, 1295, 1156 cm^{-1}. HRMS (FAB+) calcd for $C_{16}H_{18}O_4Na$ $[M+Na]^+$ 297.1103, found 297.1099.

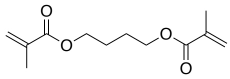

Butane-1,4-diyl dimethacrylate (7i):[2] Colorless oil. ^{1}H NMR (400 MHz, $CDCl_3$) δ 1.76–1.83 (m, 4H), 1.95 (t, J = 1.4 Hz, 6H), 4.17–4.22 (m, 4H), 5.55–5.57 (m, 2H), 6.10 (s, 2H); ^{13}C NMR (100 MHz, $CDCl_3$) δ 18.4(2C), 25.5(2C), 64.3(2C), 125.5(2C), 136.5(2C), 167.5(2C); IR (neat) 2959, 2928, 2855, 1713, 1637, 1453, 1404, 1377, 1323, 1297, 1165 cm^{-1}. HRMS (DART+) calcd for $C_{12}H_{19}O_4$ $[M+H]^+$ 227.1283, found 227.1287.

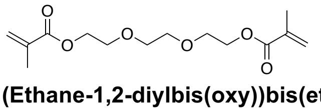

(Ethane-1,2-diylbis(oxy))bis(ethane-2,1-diyl) dimethacrylate (7k):[2] Colorless oil. ^{1}H NMR (400 MHz, $CDCl_3$) δ 1.95 (s, 6H), 3.68 (s, 4H), 3.74-3.77 (m, 4H), 4.29-4.32 (m, 4H), 5.57-5.59 (m, 2H), 6.13-6.14 (m, 2H); ^{13}C NMR (100 MHz, $CDCl_3$) δ 18.4 (2C), 63.9 (2C), 69.3 (2C), 70.7 (2C), 125.9 (2C), 136.2 (2C), 167.5 (2C); IR (neat) 2955, 2874, 1719, 1637, 1454, 1319, 1297, 1171, 1043 cm^{-1}. HRMS (DART+) calcd for $C_{14}H_{23}O_6$ $[M+H]^+$ 287.1495, found 287.1495.

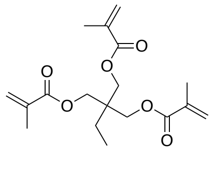

1,1,1-Trimethylolpropane trimethacrylate (7i):[2] Pale yellow oil. ^{1}H NMR (400 MHz, $CDCl_3$) δ 0.95 (t, *J* = 7.6 Hz, 3H), 1.59 (q, *J* = 7.9 Hz, 2H), 1.94 (s, 9H), 4.16 (s, 6H), 5.58-5.60 (m, 3H), 6.10 (s, 3H); ^{13}C NMR (100 MHz, $CDCl_3$) δ 7.3, 18.1(3C), 23.3, 41.0, 64.1(3C), 125.9(3C), 135.8(3C), 166.8(3C); IR (neat) 2967, 1724, 1638, 1455, 1322, 1294, 1154 cm^{-1}. HRMS (FAB+) calcd for $C_{18}H_{26}NaO_6$ $[M+Na]^+$ 361.1627, found 361.1623.

7. X-ray diffraction analysis of Mg(PBTP) and Na_2(PBTP)

Single Crystal Structure of $[Mg(PBTP)\cdot THF]_2$

In a solution of PBTP-H_2 (136 mg, 0.3 mmol) in THF (3 mL), dibutylmagnesium (300 μL, 1.0 *M* in hepatne) was added under argon atmosphere. After 0.5 h, the resultant solution was concentrated *in vacuo* to give white solid. The solid was dissolved in toluene and hexane and recrystallized overnight. All manipulations were carried out in glove box.

Crystal data of $[Mga(PBTP)\cdot THF]_2$ (Figure S1): Formula $C_{70}H_{108}Mg_2O_6$, colorless, monoclinic, space group *P* 21/c, *a* = 16.2490(5) Å, *b* = 10.6440(3) Å, *c* =19.0878(5) Å, α = 90°, β = 96.071(3)°, γ = 90 °, *V* = 3282.80(16) Å^3, Z = 2, ρcalc = 1.107 g/cm^3, λ(MoKα) = 0.71073 Å, *T* = 93 K. 8210 reflections collected, and 6084 parameters were used for the solution of the structure. R_1 = 0.0659 and wR_2 = 0.1828. GOF = 1.044. Crystallographic data (excluding structure factors) for the structure reported in this paper have been deposited with the Cambridge Crystallographic Data Centre as supplementary publication no. CCDC-2151275. Copies of the data can be obtained free of charge on application to CCDC, 12 Union Road, Cambridge CB2 1EZ, UK [Fax: int. code + 44(1223)336-033; E-mail: deposit@ccdc.cam.ac.uk; Web page: http://www.ccdc.cam.ac.uk/pages/Home.aspx].

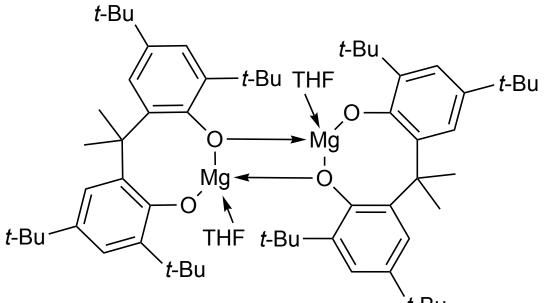

$[Mg(PBTP)\bullet THF]_2$

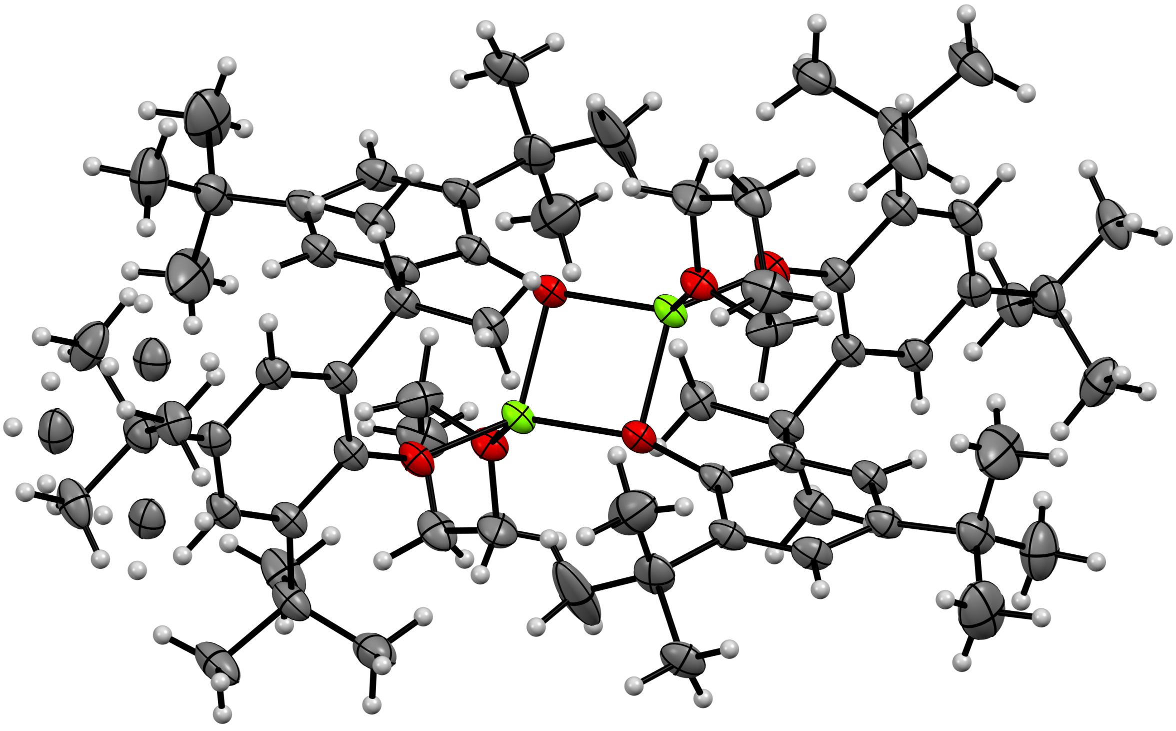

Figure S1. X-ray crystal structure of $[Mg(PBTP)\cdot THF]_2$ with thermal ellipsoids at 50% probability.

Single Crystal Structure of $Na_2(PBTP)\cdot 4THF$

In a solution of PBTP-H_2 (136 mg, 0.3 mmol) in THF (3 mL), sodium hydride (9 mmol) was added under argon atmosphere. After 14 h, the resultant mixture was filtrated through celite pad, and concentrated *in vacuo* to give white solid. The solid was dissolved in THF and recrystallized by adsorption of THF using alumina overnight. All manipulations were carried out in glove box.

Crystal data of Na_2(PBTP)·4THF (Figure S2): Formula $C_{47}H_{78}Na_2O_6$, colorless, monoclinic, space group *C* 2/c, a = 13.2993(4) Å, b = 14.9598(4) Å, c =23.1561(7) Å, α = 90°, β = 92.864(3)°, γ = 90 °, V = 4601.3(2) Å^3, Z = 4, ρcalc = 1.133 g/cm^3, λ(MoKα) = 0.71073 Å, T = 93 K. 5819 reflections collected, and 4503 parameters were used for the solution of the structure. R_1 = 0.0572 and wR_2 = 0.1463. GOF = 1.016. Crystallographic data (excluding structure factors) for the structure reported in this paper have been deposited with the Cambridge Crystallographic Data Centre as supplementary publication no. CCDC-2152692. Copies of the data can be obtained free of charge on application to CCDC, 12 Union Road, Cambridge CB2 1EZ, UK [Fax: int. code + 44(1223)336-033; E-mail: deposit@ccdc.cam.ac.uk; Web page: http://www.ccdc.cam.ac.uk/pages/Home.aspx].

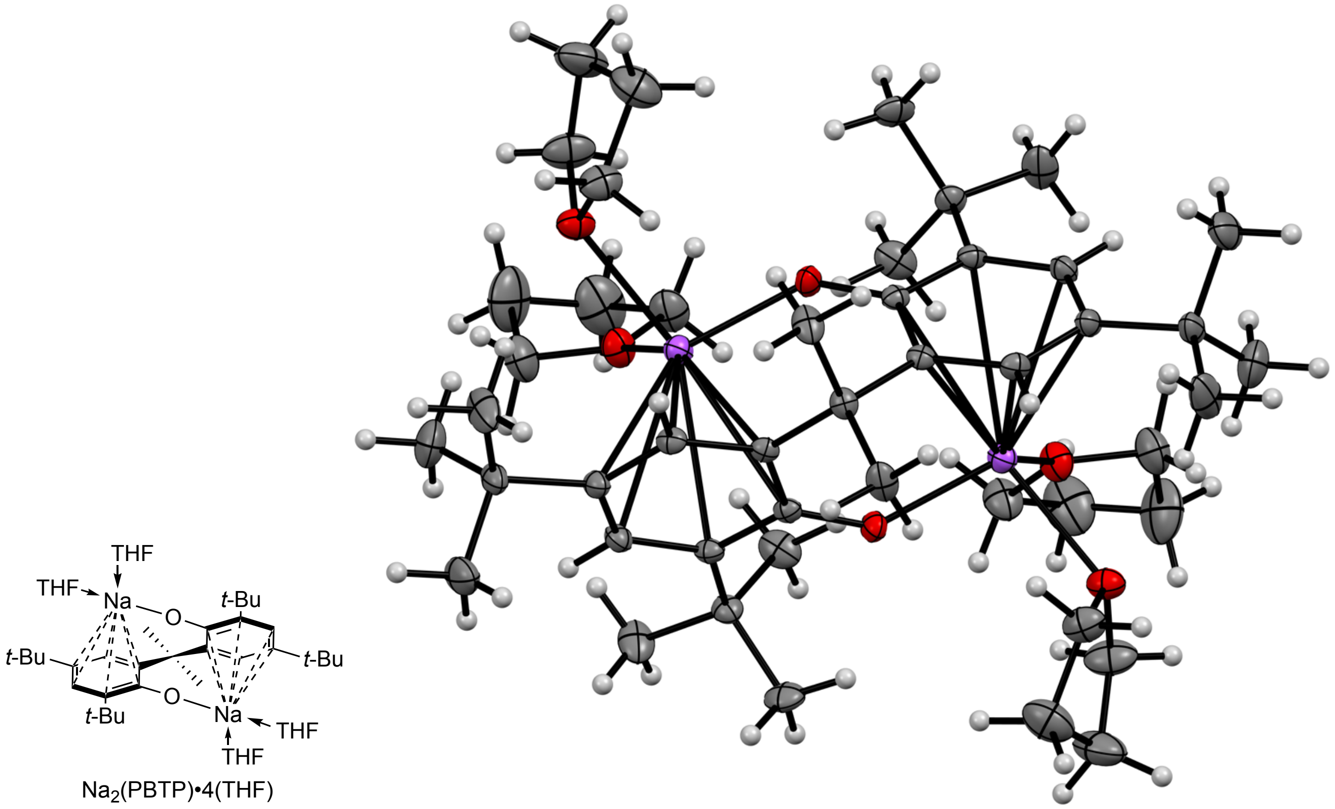

Figure S2. X-crystal structure of Na_2(PBTP)·4THF with thermal ellipsoids at 50% probability.

8. DFT calculations for potential energy profile including Mg(II) and Na(I) species.

8-1. Computational details.

DFT calculations were performed at the wb97XD[3]/6-31G(d,p)[4] level. Solvent effect was taken into account by a self-consistent reaction field method with polarizable continuum model.[5] Solvent parameters for methylpropanoate was adopted because of the structural similarity to methyl acrylate (MA). For computational software, Gaussian 16 package[6] was used. For the structural optimization, frequency analysis was performed to check number of imaginary frequency of energy minimum and transition state.

8-2. Comparison between X-ray structure and DFT optimized structure for [Mg(PBTP)•THF]$_2$ and Na$_2$(PBTP)•4(THF).

The computed geometrical parameter (bond lengths are given in Å) of the X-ray structures of [Mg(PBTP)•THF]$_2$ and Na$_2$(PBTP)•4(THF) are represented in **Figure S3** and **Figure S4**, respectively. Calculated bond lengths around the Mg and Na moieties were in good agreement with the X-ray structures, indicating that the reliability of the present DFT calculation set-up.

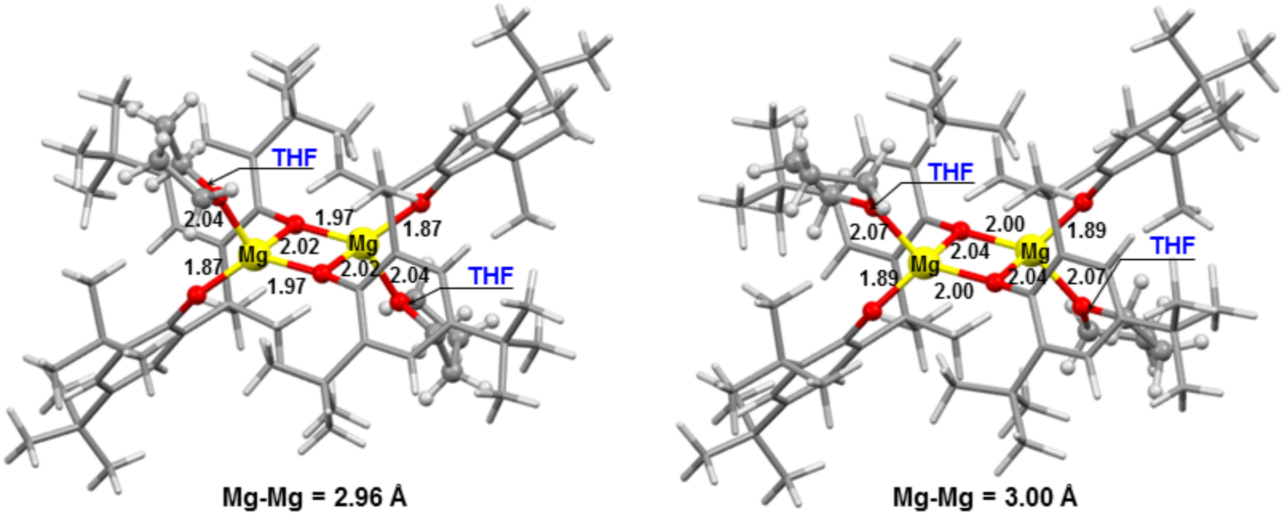

Figure S3. X-ray structure of [Mg(PBTP)•THF]$_2$ (left) and optimized structure from the DFT calculation (right). Bond lengths are given in Å.

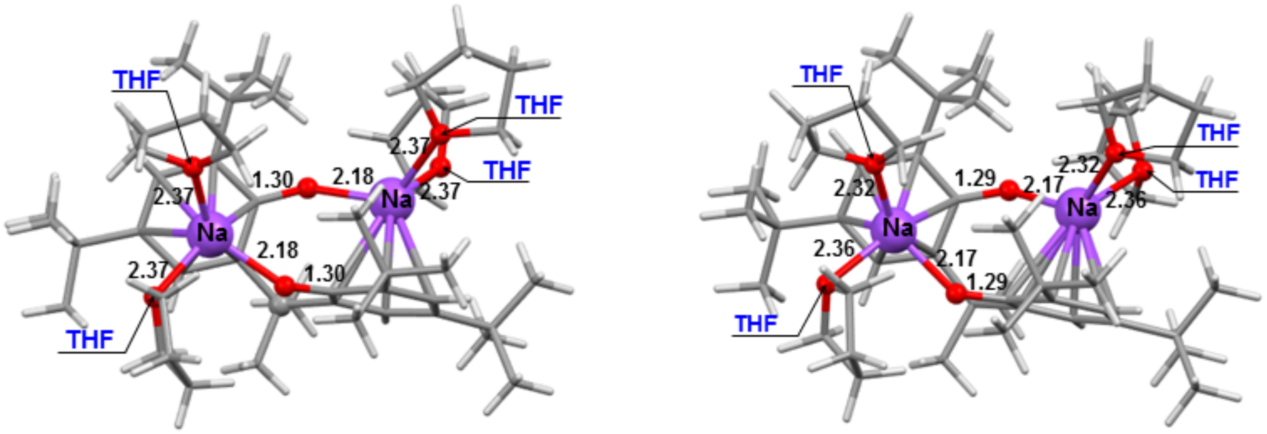

Figure S4. X-ray structure of Na$_2$(PBTP)•4(THF) (left) and optimized structure from the DFT calculation (right). Bond lengths are given in Å.

8-3. Optimized structures for intermediate and transition states shown in Figure 2 for the transesterification of MA with 1a using [Mg(PBTP)]$_2$ catalyst.

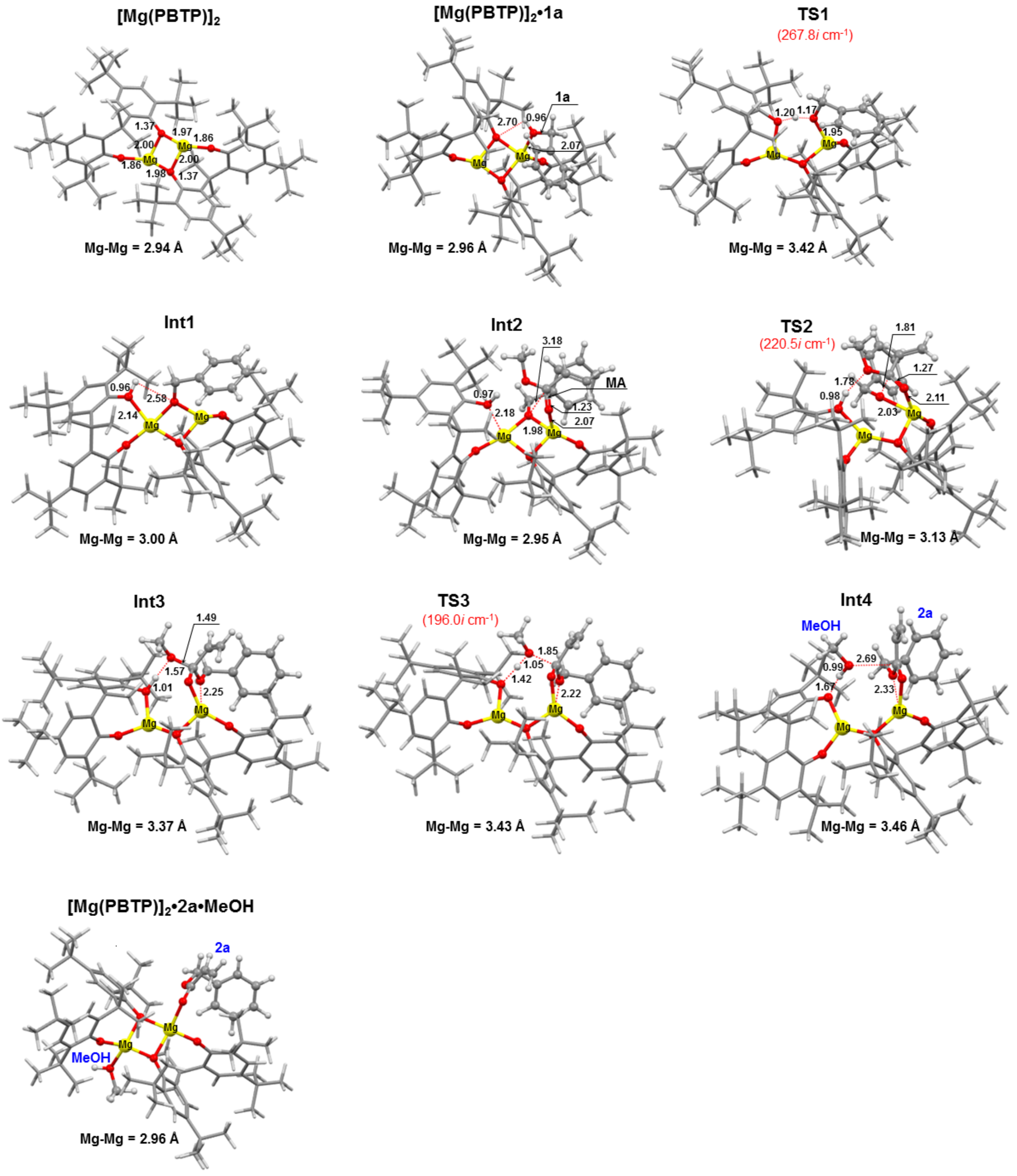

Figure S5. Optimized structures of intermediates and transition states for the transesterification of MA with **1a** using [Mg(PBTP)]$_2$ catalyst. Bond lengths are given in Å.

8-4. Optimized structures for intermediate and transition states at the rate determining step (RDS) in Table 5 for the transesterification of MMA with 1a using Na_2(PBTP)

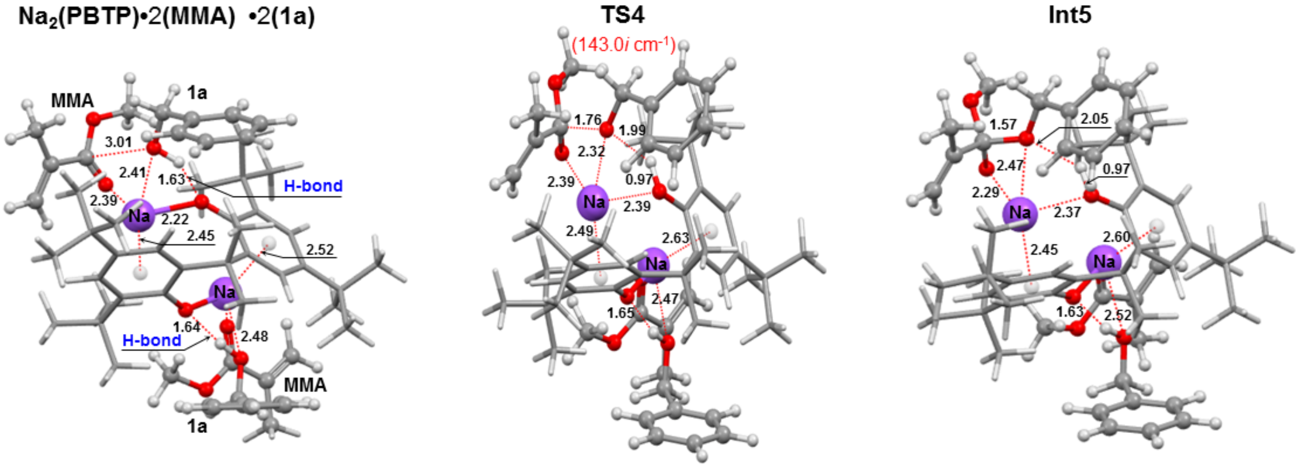

Figure S6. Optimized structures of intermediates and transition at the RDS for the transesterification of MMA with 1a using Na_2(PBTP) catalyst. Bond lengths are given in Å.

8-5. Optimized structures for intermediate and transition states at the rate determining step (RDS) in Table 5 for the transesterification of MA with 1a using Mg(PBTP) catalyst.

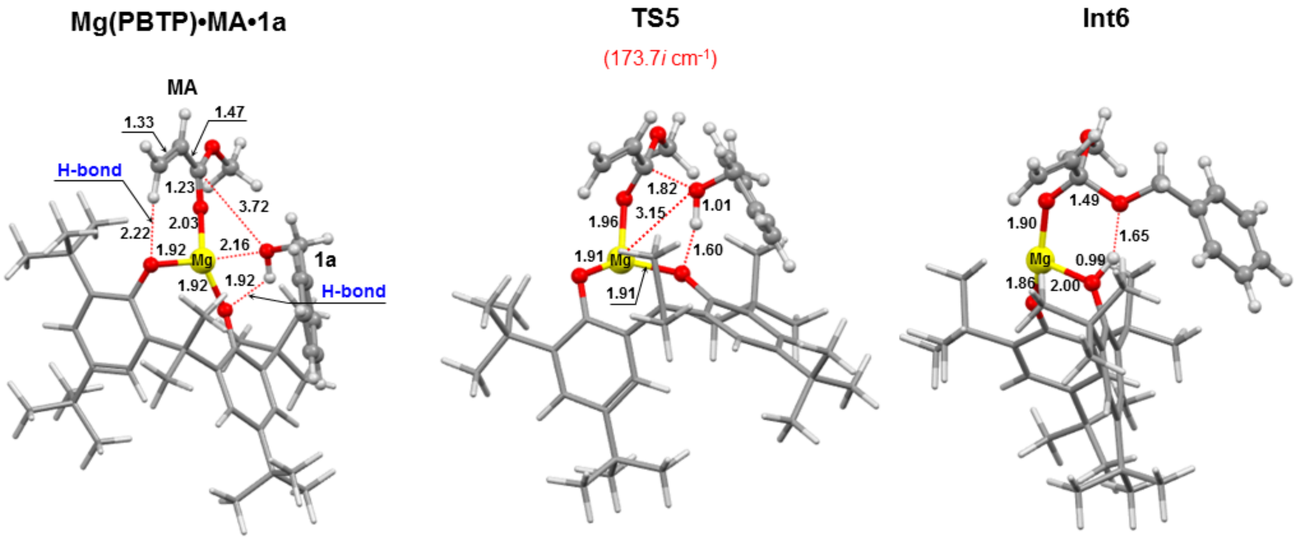

Figure S7. Optimized structures of intermediates and transition states for the transesterification of MA with **1a** using Mg(PBTP) catalyst. Bond lengths are given in Å

8-6. Optimized structures for intermediate and transition states at the rate determining step (RDS) in Table 5 for the transesterification of MMA with 1a using Na(BHT) catalyst.

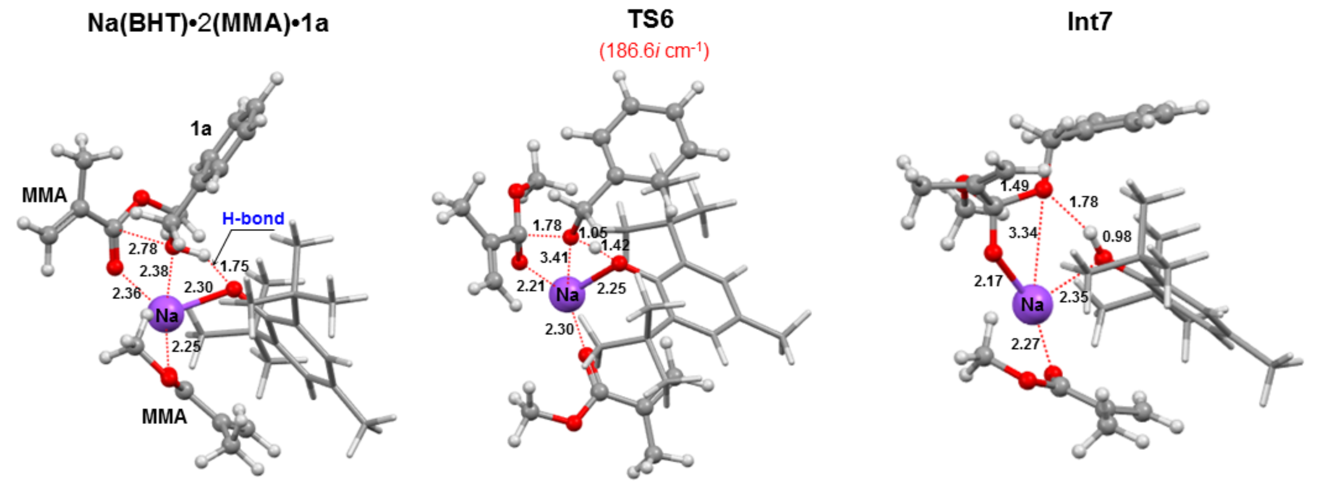

Figure S8. Optimized structures of intermediates and transition at the RDS for the transesterification of MMA with 1a using Na(BHT) catalyst. Bond lengths are given in Å.

8-7. Atomic coordinates of the optimized structures

[Mg(PBTP)•THF]2-optXray-byDFT

Mg	-1.065000	-0.163000	-1.044000
O	-0.610000	1.100000	0.487000
O	-2.784000	-0.955000	-0.974000
O	-1.522000	1.088000	-2.628000
C	-1.703000	1.884000	0.719000
C	-2.922000	1.276000	1.109000
C	-4.099000	2.014000	0.997000
H	-5.031000	1.498000	1.205000
C	-4.136000	3.349000	0.624000
C	-2.908000	3.965000	0.417000
H	-2.896000	5.017000	0.176000
C	-1.687000	3.282000	0.445000
C	-3.135000	-0.125000	1.747000
C	-1.910000	-1.031000	1.926000
H	-2.210000	-1.906000	2.513000
H	-1.129000	-0.517000	2.490000
H	-1.500000	-1.416000	0.998000
C	-3.584000	0.173000	3.200000
H	-4.479000	0.796000	3.254000
H	-2.780000	0.712000	3.713000
H	-3.773000	-0.753000	3.752000
C	-5.484000	4.052000	0.439000
C	-6.253000	3.344000	-0.694000
H	-6.424000	2.288000	-0.465000
H	-5.692000	3.397000	-1.633000
H	-7.228000	3.819000	-0.851000
C	-5.321000	5.531000	0.064000
H	-6.306000	5.992000	-0.058000
H	-4.780000	5.651000	-0.881000
H	-4.786000	6.089000	0.839000
C	-6.306000	3.971000	1.739000
H	-7.272000	4.471000	1.610000
H	-5.776000	4.457000	2.565000
H	-6.505000	2.935000	2.028000
C	-0.421000	4.077000	0.061000
C	0.780000	3.709000	0.942000
H	0.515000	3.761000	2.004000
H	1.599000	4.415000	0.771000
H	1.174000	2.720000	0.729000
C	-0.091000	3.821000	-1.421000
H	0.867000	4.281000	-1.687000
H	-0.866000	4.252000	-2.066000
H	-0.033000	2.755000	-1.634000
C	-0.619000	5.597000	0.232000
H	-0.933000	5.852000	1.249000
H	-1.345000	6.015000	-0.471000
H	0.333000	6.100000	0.038000
C	-3.950000	-1.227000	-0.420000
C	-4.204000	-0.878000	0.939000
C	-5.442000	-1.164000	1.502000
H	-5.618000	-0.894000	2.537000
C	-6.481000	-1.759000	0.788000
C	-6.218000	-2.087000	-0.538000
H	-7.007000	-2.547000	-1.116000
C	-4.991000	-1.852000	-1.166000
C	-7.836000	-1.999000	1.465000
C	-7.658000	-2.973000	2.646000
H	-6.955000	-2.579000	3.387000
H	-8.615000	-3.148000	3.150000
H	-7.272000	-3.936000	2.296000
C	-8.869000	-2.599000	0.502000
H	-9.056000	-1.938000	-0.351000
H	-8.548000	-3.572000	0.117000
H	-9.820000	-2.746000	1.024000
C	-8.394000	-0.662000	1.989000
H	-8.520000	0.053000	1.169000
H	-9.369000	-0.812000	2.468000
H	-7.727000	-0.208000	2.729000
C	-4.796000	-2.271000	-2.637000
C	-3.687000	-3.332000	-2.735000
H	-3.941000	-4.219000	-2.145000
H	-2.748000	-2.927000	-2.361000
H	-3.546000	-3.645000	-3.777000
C	-6.069000	-2.874000	-3.253000
H	-5.867000	-3.146000	-4.294000
H	-6.902000	-2.163000	-3.253000
H	-6.390000	-3.782000	-2.733000
C	-4.400000	-1.063000	-3.504000
H	-3.435000	-0.674000	-3.181000
H	-5.146000	-0.263000	-3.429000
H	-4.326000	-1.359000	-4.558000
C	-0.980000	1.182000	-3.960000
H	-0.510000	0.226000	-4.198000
H	-0.221000	1.973000	-3.975000
C	-2.173000	1.523000	-4.836000
H	-1.870000	2.010000	-5.764000
H	-2.731000	0.617000	-5.083000
C	-2.993000	2.437000	-3.920000
H	-4.055000	2.446000	-4.173000
H	-2.619000	3.464000	-3.980000
C	-2.755000	1.864000	-2.526000
H	-2.611000	2.631000	-1.767000
H	-3.532000	1.175000	-2.195000
Mg	1.066000	0.161000	1.044000
O	0.610000	-1.102000	-0.487000
O	2.786000	0.951000	0.976000
O	1.521000	-1.090000	2.629000
C	1.704000	-1.886000	-0.718000
C	2.922000	-1.276000	-1.108000
C	4.100000	-2.015000	-0.996000
H	5.032000	-1.498000	-1.204000
C	4.138000	-3.349000	-0.622000
C	2.909000	-3.965000	-0.415000
H	2.898000	-5.018000	-0.175000
C	1.688000	-3.283000	-0.444000
C	3.134000	0.124000	-1.747000
C	1.909000	1.030000	-1.926000
H	2.210000	1.904000	-2.512000
H	1.129000	0.516000	-2.490000
H	1.499000	1.414000	-0.998000
C	3.583000	-0.175000	-3.200000
H	4.479000	-0.797000	-3.254000
H	2.780000	-0.715000	-3.712000
H	3.771000	0.752000	-3.753000
C	5.486000	-4.052000	-0.438000
C	6.255000	-3.343000	0.695000
H	6.425000	-2.287000	0.466000
H	5.694000	-3.396000	1.635000
H	7.230000	-3.817000	0.852000
C	5.323000	-5.530000	-0.062000
H	6.309000	-5.991000	0.060000
H	4.783000	-5.650000	0.883000
H	4.788000	-6.088000	-0.837000

C	6.307000	-3.971000	-1.738000
H	7.274000	-4.470000	-1.609000
H	5.778000	-4.457000	-2.563000
H	6.506000	-2.934000	-2.027000
C	0.423000	-4.079000	-0.060000
C	-0.778000	-3.712000	-0.942000
H	-0.512000	-3.763000	-2.004000
H	-1.596000	-4.418000	-0.772000
H	-1.173000	-2.723000	-0.729000
C	0.092000	-3.823000	1.422000
H	-0.867000	-4.283000	1.687000
H	0.866000	-4.253000	2.067000
H	0.033000	-2.756000	1.634000
C	0.621000	-5.599000	-0.231000
H	0.936000	-5.854000	-1.248000
H	1.348000	-6.016000	0.472000
H	-0.331000	-6.102000	-0.037000
C	3.951000	1.225000	0.420000
C	4.204000	0.878000	-0.940000
C	5.440000	1.166000	-1.504000
H	5.616000	0.897000	-2.540000
C	6.479000	1.763000	-0.790000
C	6.216000	2.090000	0.536000
H	7.005000	2.551000	1.113000
C	4.991000	1.852000	1.165000
C	7.833000	2.005000	-1.469000
C	7.652000	2.979000	-2.649000
H	6.949000	2.585000	-3.389000
H	8.609000	3.156000	-3.154000
H	7.266000	3.941000	-2.298000
C	8.866000	2.606000	-0.506000
H	9.055000	1.945000	0.346000
H	8.544000	3.579000	-0.121000
H	9.817000	2.755000	-1.029000
C	8.392000	0.669000	-1.994000
H	8.520000	-0.046000	-1.175000
H	9.367000	0.820000	-2.473000
H	7.726000	0.214000	-2.733000
C	4.797000	2.270000	2.637000
C	3.686000	3.329000	2.737000
H	3.939000	4.217000	2.148000
H	2.748000	2.923000	2.363000
H	3.546000	3.640000	3.779000
C	6.070000	2.874000	3.252000
H	5.869000	3.145000	4.293000
H	6.904000	2.164000	3.251000
H	6.389000	3.783000	2.732000
C	4.404000	1.060000	3.503000
H	3.439000	0.670000	3.181000
H	5.151000	0.262000	3.426000
H	4.332000	1.355000	4.557000
C	0.979000	-1.181000	3.961000
H	0.511000	-0.223000	4.198000
H	0.219000	-1.970000	3.978000
C	2.171000	-1.523000	4.838000
H	1.868000	-2.009000	5.767000
H	2.732000	-0.618000	5.083000
C	2.989000	-2.440000	3.922000
H	4.051000	-2.451000	4.176000
H	2.612000	-3.465000	3.982000
C	2.753000	-1.867000	2.528000
H	2.610000	-2.633000	1.768000
H	3.531000	-1.178000	2.198000

Na2(PBTP)•4(THF)-optXray-byDFT

Na	-2.283000	-0.392000	0.119000
O	-0.762000	-1.736000	-0.648000
C	0.499000	-1.647000	-0.915000
C	0.983000	-0.833000	-1.997000
C	2.333000	-0.883000	-2.346000
H	2.662000	-0.300000	-3.196000
C	3.287000	-1.621000	-1.640000
C	2.827000	-2.313000	-0.514000
H	3.553000	-2.855000	0.082000
C	1.489000	-2.337000	-0.121000
C	0.000000	0.002000	-2.840000
C	-0.725000	-1.011000	-3.752000
H	0.024000	-1.600000	-4.289000
H	-1.356000	-0.523000	-4.502000
H	-1.334000	-1.692000	-3.151000
C	4.768000	-1.688000	-2.034000
C	5.170000	-3.155000	-2.279000
H	4.571000	-3.590000	-3.085000
H	5.021000	-3.767000	-1.384000
H	6.227000	-3.224000	-2.560000
C	5.066000	-0.899000	-3.317000
H	6.126000	-0.998000	-3.573000
H	4.855000	0.169000	-3.197000
H	4.481000	-1.270000	-4.165000
C	5.639000	-1.107000	-0.904000
H	6.701000	-1.150000	-1.172000
H	5.507000	-1.667000	0.027000
H	5.372000	-0.063000	-0.709000
C	1.058000	-3.096000	1.145000
C	0.107000	-4.246000	0.767000
H	0.623000	-4.982000	0.141000
H	-0.743000	-3.848000	0.212000
H	-0.255000	-4.759000	1.667000
C	2.245000	-3.698000	1.910000
H	1.876000	-4.206000	2.808000
H	2.951000	-2.924000	2.234000
H	2.791000	-4.437000	1.315000
C	0.350000	-2.127000	2.110000
H	0.020000	-2.654000	3.014000
H	-0.520000	-1.681000	1.629000
H	1.037000	-1.328000	2.410000
O	-3.996000	-1.991000	-0.130000
C	-3.609000	-3.059000	0.750000
H	-4.329000	-3.107000	1.572000
H	-2.616000	-2.836000	1.160000
C	-3.569000	-4.319000	-0.109000
H	-4.557000	-4.790000	-0.151000
H	-2.852000	-5.050000	0.270000
C	-3.183000	-3.750000	-1.478000
H	-2.112000	-3.528000	-1.508000
H	-3.450000	-4.404000	-2.311000
C	-3.952000	-2.437000	-1.495000
H	-3.457000	-1.662000	-2.087000
H	-4.980000	-2.563000	-1.859000
O	-2.971000	-0.274000	2.333000
C	-4.363000	0.071000	2.439000
H	-4.897000	-0.399000	1.610000
H	-4.453000	1.160000	2.345000
C	-4.816000	-0.420000	3.813000
H	-5.603000	0.206000	4.237000
H	-5.192000	-1.446000	3.747000
C	-3.511000	-0.387000	4.613000
H	-3.522000	-1.038000	5.488000
H	-3.287000	0.634000	4.940000
C	-2.506000	-0.836000	3.563000
H	-1.488000	-0.477000	3.737000
H	-2.479000	-1.931000	3.476000
O	0.762000	1.737000	-0.645000
C	-0.498000	1.648000	-0.913000
C	-0.983000	0.836000	-1.995000
C	-2.332000	0.886000	-2.345000
H	-2.661000	0.303000	-3.195000
C	-3.287000	1.623000	-1.638000
C	-2.827000	2.314000	-0.511000
H	-3.553000	2.855000	0.086000
C	-1.489000	2.337000	-0.117000
C	0.726000	1.017000	-3.749000
H	-0.023000	1.607000	-4.286000
H	1.357000	0.531000	-4.501000
H	1.335000	1.697000	-3.148000
C	-4.768000	1.690000	-2.032000
C	-5.169000	3.158000	-2.275000
H	-4.570000	3.594000	-3.081000
H	-5.021000	3.768000	-1.379000
H	-6.226000	3.228000	-2.557000
C	-5.065000	0.903000	-3.316000
H	-6.125000	1.003000	-3.573000
H	-4.855000	-0.164000	-3.197000
H	-4.480000	1.276000	-4.163000
C	-5.639000	1.108000	-0.903000
H	-6.701000	1.152000	-1.171000
H	-5.507000	1.667000	0.029000
H	-5.372000	0.064000	-0.709000
C	-1.058000	3.094000	1.150000
C	-0.107000	4.245000	0.773000
H	-0.624000	4.982000	0.148000
H	0.742000	3.848000	0.218000
H	0.255000	4.757000	1.674000
C	-2.246000	3.695000	1.916000
H	-1.876000	4.202000	2.813000
H	-2.951000	2.921000	2.238000
H	-2.791000	4.435000	1.321000
C	-0.350000	2.124000	2.113000
H	-0.020000	2.650000	3.018000
H	0.520000	1.679000	1.631000
H	-1.037000	1.325000	2.412000
Na	2.284000	0.392000	0.119000
O	3.996000	1.991000	-0.127000
C	3.608000	3.058000	0.755000
H	4.329000	3.104000	1.577000
H	2.616000	2.834000	1.164000
C	3.568000	4.319000	-0.102000
H	4.555000	4.791000	-0.142000
H	2.850000	5.049000	0.279000
C	3.182000	3.753000	-1.472000
H	2.112000	3.530000	-1.503000
H	3.450000	4.408000	-2.303000
C	3.953000	2.440000	-1.491000
H	3.459000	1.666000	-2.085000
H	4.981000	2.568000	-1.854000
O	2.971000	0.270000	2.333000
C	4.363000	-0.075000	2.439000
H	4.897000	0.398000	1.611000
H	4.453000	-1.164000	2.343000
C	4.815000	0.413000	3.814000
H	5.602000	-0.214000	4.238000
H	5.192000	1.439000	3.750000
C	3.510000	0.379000	4.613000
H	3.521000	1.029000	5.490000
H	3.285000	-0.642000	4.938000
C	2.505000	0.830000	3.564000
H	1.487000	0.472000	3.737000
H	2.479000	1.925000	3.479000

[Mg(PBTP)]2

Mg	0.966000	-0.413000	0.994000
O	0.655000	1.189000	-0.164000
O	2.773000	-0.824000	1.095000
C	1.723000	2.048000	-0.198000
C	2.979000	1.591000	-0.661000
C	4.098000	2.387000	-0.416000
H	5.067000	1.984000	-0.693000
C	4.035000	3.647000	0.159000
C	2.767000	4.120000	0.478000
H	2.681000	5.115000	0.887000
C	1.602000	3.362000	0.325000
C	3.305000	0.318000	-1.490000
C	2.132000	-0.563000	-1.944000
H	2.519000	-1.331000	-2.620000
H	1.400000	0.023000	-2.509000
H	1.629000	-1.103000	-1.148000
C	3.886000	0.843000	-2.830000
H	4.791000	1.440000	-2.706000
H	3.138000	1.480000	-3.311000
H	4.112000	0.017000	-3.512000
C	5.321000	4.449000	0.387000
C	6.260000	3.649000	1.310000
H	6.521000	2.678000	0.879000
H	5.787000	3.468000	2.281000
H	7.191000	4.202000	1.479000
C	5.050000	5.812000	1.038000
H	5.996000	6.341000	1.191000
H	4.567000	5.704000	2.014000
H	4.416000	6.443000	0.407000

C	6.021000	4.690000	-0.965000
H	6.943000	5.264000	-0.821000
H	5.370000	5.251000	-1.643000
H	6.287000	3.748000	-1.454000
C	0.265000	3.977000	0.797000
C	-0.878000	3.745000	-0.212000
H	-0.536000	3.898000	-1.240000
H	-1.696000	4.448000	-0.028000
H	-1.324000	2.755000	-0.135000
C	-0.100000	3.398000	2.177000
H	-1.076000	3.772000	2.508000
H	0.649000	3.690000	2.919000
H	-0.151000	2.310000	2.156000
C	0.366000	5.507000	0.965000
H	0.675000	5.997000	0.036000
H	1.059000	5.801000	1.758000
H	-0.617000	5.899000	1.242000
C	3.956000	-1.082000	0.561000
C	4.304000	-0.559000	-0.715000
C	5.557000	-0.844000	-1.247000
H	5.815000	-0.442000	-2.220000
C	6.506000	-1.617000	-0.578000
C	6.149000	-2.106000	0.675000
H	6.868000	-2.702000	1.220000
C	4.909000	-1.860000	1.269000
C	7.872000	-1.876000	-1.228000
C	7.676000	-2.607000	-2.570000
H	7.067000	-2.020000	-3.264000
H	8.643000	-2.795000	-3.050000
H	7.177000	-3.569000	-2.417000
C	8.782000	-2.739000	-0.344000
H	8.988000	-2.257000	0.617000
H	8.341000	-3.722000	-0.147000
H	9.741000	-2.900000	-0.848000
C	8.583000	-0.533000	-1.480000
H	8.735000	0.006000	-0.539000
H	9.563000	-0.697000	-1.944000
H	8.002000	0.112000	-2.146000
C	4.595000	-2.407000	2.674000
C	3.375000	-3.347000	2.622000
H	3.571000	-4.195000	1.957000
H	2.496000	-2.822000	2.251000
H	3.157000	-3.743000	3.621000
C	5.762000	-3.215000	3.265000
H	5.482000	-3.570000	4.262000
H	6.668000	-2.610000	3.374000
H	6.003000	-4.093000	2.657000
C	4.320000	-1.235000	3.637000
H	3.494000	-0.622000	3.274000
H	5.207000	-0.599000	3.726000
H	4.072000	-1.612000	4.636000
Mg	-0.967000	0.470000	-1.030000
O	-0.647000	-1.145000	0.109000
O	-2.786000	0.829000	-1.107000
C	-1.706000	-2.014000	0.167000
C	-2.952000	-1.577000	0.678000
C	-4.066000	-2.394000	0.483000
H	-5.030000	-2.005000	0.793000
C	-4.005000	-3.658000	-0.084000
C	-2.746000	-4.105000	-0.467000
H	-2.661000	-5.097000	-0.882000
C	-1.591000	-3.321000	-0.376000
C	-3.272000	-0.297000	1.497000
C	-2.098000	0.595000	1.920000
H	-2.478000	1.375000	2.586000
H	-1.360000	0.020000	2.488000
H	-1.606000	1.121000	1.109000
C	-3.826000	-0.803000	2.856000
H	-4.729000	-1.408000	2.763000
H	-3.065000	-1.426000	3.335000
H	-4.045000	0.034000	3.527000
C	-5.285000	-4.485000	-0.247000
C	-6.278000	-3.713000	-1.136000
H	-6.540000	-2.743000	-0.704000
H	-5.851000	-3.534000	-2.128000
H	-7.204000	-4.286000	-1.259000
C	-5.017000	-5.850000	-0.894000
H	-5.958000	-6.400000	-0.996000
H	-4.583000	-5.745000	-1.894000
H	-4.340000	-6.460000	-0.287000
C	-5.920000	-4.723000	1.137000
H	-6.837000	-5.316000	1.040000
H	-5.231000	-5.265000	1.792000
H	-6.182000	-3.781000	1.628000
C	-0.278000	-3.893000	-0.956000
C	0.912000	-3.726000	0.007000
H	0.655000	-4.093000	1.007000
H	1.770000	-4.305000	-0.349000
H	1.259000	-2.701000	0.097000
C	-0.000000	-3.215000	-2.311000
H	0.974000	-3.521000	-2.708000
H	-0.770000	-3.497000	-3.036000
H	-0.003000	-2.129000	-2.226000
C	-0.372000	-5.408000	-1.229000
H	-0.612000	-5.971000	-0.321000
H	-1.112000	-5.654000	-1.996000
H	0.597000	-5.761000	-1.595000
C	-3.966000	1.074000	-0.561000
C	-4.291000	0.560000	0.726000
C	-5.542000	0.833000	1.268000
H	-5.783000	0.439000	2.249000
C	-6.511000	1.584000	0.602000
C	-6.177000	2.063000	-0.661000
H	-6.911000	2.641000	-1.205000
C	-4.940000	1.829000	-1.267000
C	-7.873000	1.828000	1.265000
C	-7.674000	2.576000	2.597000
H	-7.048000	2.006000	3.290000
H	-8.638000	2.754000	3.086000
H	-7.192000	3.544000	2.428000
C	-8.807000	2.667000	0.382000
H	-9.017000	2.170000	-0.571000
H	-8.384000	3.654000	0.169000
H	-9.762000	2.818000	0.896000
C	-8.560000	0.476000	1.540000
H	-8.713000	-0.076000	0.606000
H	-9.537000	0.629000	2.012000
H	-7.961000	-0.152000	2.206000
C	-4.651000	2.362000	-2.682000
C	-3.447000	3.322000	-2.657000
H	-3.651000	4.179000	-2.007000
H	-2.556000	2.819000	-2.282000
H	-3.242000	3.703000	-3.665000
C	-5.838000	3.143000	-3.270000
H	-5.575000	3.491000	-4.274000
H	-6.735000	2.522000	-3.360000
H	-6.087000	4.025000	-2.670000
C	-4.368000	1.182000	-3.633000
H	-3.529000	0.587000	-3.271000
H	-5.246000	0.531000	-3.703000
H	-4.138000	1.550000	-4.640000

[Mg(PBTP)]2-1a

Mg	-0.981000	-0.306000	-1.422000
O	-0.723000	1.198000	-0.153000
O	-2.780000	-0.722000	-1.650000
C	-1.793000	2.031000	0.011000
C	-3.042000	1.507000	0.426000
C	-4.165000	2.331000	0.333000
H	-5.129000	1.884000	0.554000
C	-4.114000	3.667000	-0.030000
C	-2.854000	4.184000	-0.311000
H	-2.777000	5.230000	-0.564000
C	-1.690000	3.411000	-0.321000
C	-3.364000	0.115000	1.040000
C	-2.203000	-0.844000	1.337000
H	-2.581000	-1.659000	1.961000
H	-1.406000	-0.360000	1.901000
H	-1.787000	-1.330000	0.461000
C	-3.937000	0.415000	2.451000
H	-4.814000	1.063000	2.442000
H	-3.168000	0.929000	3.037000
H	-4.198000	-0.510000	2.975000
C	-5.404000	4.492000	-0.096000
C	-6.344000	3.874000	-1.149000
H	-6.591000	2.835000	-0.911000
H	-5.877000	3.889000	-2.139000
H	-7.282000	4.439000	-1.204000
C	-5.140000	5.954000	-0.479000
H	-6.088000	6.501000	-0.514000
H	-4.672000	6.037000	-1.465000
H	-4.495000	6.454000	0.251000
C	-6.099000	4.472000	1.279000
H	-7.022000	5.062000	1.250000
H	-5.446000	4.894000	2.050000
H	-6.365000	3.454000	1.582000
C	-0.381000	4.080000	-0.794000
C	0.820000	3.746000	0.110000
H	0.563000	3.860000	1.168000
H	1.650000	4.429000	-0.100000
H	1.215000	2.746000	-0.045000
C	-0.108000	3.649000	-2.247000
H	0.862000	4.027000	-2.590000
H	-0.885000	4.042000	-2.910000
H	-0.100000	2.564000	-2.345000
C	-0.483000	5.619000	-0.795000
H	-0.727000	6.012000	0.198000
H	-1.224000	5.993000	-1.507000
H	0.483000	6.038000	-1.093000
C	-3.985000	-1.008000	-1.182000
C	-4.367000	-0.615000	0.132000
C	-5.656000	-0.887000	0.575000
H	-5.940000	-0.568000	1.572000
C	-6.606000	-1.539000	-0.210000
C	-6.204000	-1.935000	-1.483000
H	-6.918000	-2.450000	-2.111000
C	-4.926000	-1.694000	-1.994000
C	-8.023000	-1.759000	0.337000
C	-7.959000	-2.544000	1.661000
H	-7.382000	-2.010000	2.422000
H	-8.966000	-2.706000	2.060000
H	-7.490000	-3.522000	1.508000
C	-8.908000	-2.548000	-0.638000
H	-9.036000	-2.021000	-1.589000
H	-8.490000	-3.538000	-0.847000
H	-9.902000	-2.690000	-0.201000
C	-8.685000	-0.390000	0.589000
H	-8.760000	0.178000	-0.344000
H	-9.694000	-0.517000	0.997000
H	-8.108000	0.209000	1.300000
C	-4.555000	-2.149000	-3.417000
C	-3.366000	-3.129000	-3.366000
H	-3.628000	-4.021000	-2.786000
H	-2.497000	-2.665000	-2.902000
H	-3.096000	-3.452000	-4.379000
C	-5.710000	-2.879000	-4.123000
H	-5.385000	-3.180000	-5.124000
H	-6.590000	-2.238000	-4.241000
H	-6.012000	-3.784000	-3.587000
C	-4.195000	-0.926000	-4.283000
H	-3.358000	-0.379000	-3.849000
H	-5.050000	-0.245000	-4.355000
H	-3.927000	-1.243000	-5.298000
Mg	0.905000	0.428000	0.738000
O	0.617000	-1.078000	-0.597000
O	2.730000	0.842000	0.628000
O	0.258000	1.240000	2.527000
C	1.695000	-1.903000	-0.768000
C	2.890000	-1.396000	-1.324000
C	4.044000	-2.175000	-1.226000
H	4.975000	-1.732000	-1.566000
C	4.065000	-3.456000	-0.697000
C	2.844000	-3.977000	-0.281000
H	2.818000	-4.987000	0.100000
C	1.656000	-3.241000	-0.291000
C	3.114000	-0.055000	-2.072000
C	1.895000	0.834000	-2.354000
H	2.220000	1.678000	-2.969000
H	1.139000	0.297000	-2.939000
H	1.439000	1.263000	-1.469000

C	3.605000	-0.444000	-3.491000
H	4.528000	-1.027000	-3.489000
H	2.835000	-1.054000	-3.975000
H	3.766000	0.445000	-4.108000
C	5.393000	-4.213000	-0.583000
C	6.354000	-3.398000	0.306000
H	6.551000	-2.408000	-0.117000
H	5.929000	-3.257000	1.305000
H	7.313000	-3.917000	0.410000
C	5.218000	-5.603000	0.045000
H	6.191000	-6.097000	0.124000
H	4.793000	-5.540000	1.052000
H	4.569000	-6.242000	-0.563000
C	6.016000	-4.387000	-1.980000
H	6.976000	-4.910000	-1.908000
H	5.356000	-4.972000	-2.629000
H	6.197000	-3.422000	-2.464000
C	0.385000	-3.885000	0.297000
C	-0.803000	-3.797000	-0.680000
H	-0.570000	-4.318000	-1.614000
H	-1.690000	-4.266000	-0.243000
H	-1.079000	-2.776000	-0.931000
C	0.068000	-3.215000	1.644000
H	-0.861000	-3.609000	2.071000
H	0.873000	-3.403000	2.359000
H	-0.045000	-2.138000	1.542000
C	0.565000	-5.388000	0.587000
H	0.840000	-5.949000	-0.312000
H	1.319000	-5.575000	1.358000
H	-0.382000	-5.792000	0.957000
C	3.874000	1.164000	0.051000
C	4.147000	0.774000	-1.291000
C	5.364000	1.117000	-1.868000
H	5.563000	0.803000	-2.887000
C	6.352000	1.830000	-1.190000
C	6.071000	2.194000	0.123000
H	6.822000	2.739000	0.678000
C	4.872000	1.880000	0.768000
C	7.691000	2.122000	-1.880000
C	7.451000	2.870000	-3.205000
H	6.829000	2.288000	-3.892000
H	8.403000	3.071000	-3.709000
H	6.949000	3.826000	-3.026000
C	8.618000	2.983000	-1.011000
H	8.877000	2.480000	-0.073000
H	8.159000	3.947000	-0.768000
H	9.551000	3.181000	-1.548000
C	8.405000	0.788000	-2.173000
H	8.594000	0.243000	-1.242000
H	9.367000	0.963000	-2.670000
H	7.802000	0.146000	-2.822000
C	4.662000	2.257000	2.245000
C	3.426000	3.162000	2.404000
H	3.560000	4.096000	1.849000
H	2.531000	2.669000	2.027000
H	3.275000	3.417000	3.460000
C	5.857000	3.017000	2.841000
H	5.648000	3.244000	3.892000
H	6.779000	2.428000	2.810000
H	6.037000	3.968000	2.327000
C	4.485000	0.968000	3.070000
H	3.655000	0.379000	2.678000
H	5.392000	0.356000	3.023000
H	4.293000	1.208000	4.124000
C	0.721000	1.079000	3.889000
H	1.768000	1.386000	3.902000
H	0.150000	1.763000	4.520000
H	-0.536000	1.783000	2.480000
C	0.571000	-0.346000	4.347000
C	-0.699000	-0.888000	4.572000
C	1.696000	-1.142000	4.561000
C	-0.839000	-2.205000	4.993000
H	-1.584000	-0.276000	4.419000
C	1.558000	-2.458000	4.997000
H	2.686000	-0.726000	4.398000
C	0.292000	-2.991000	5.210000
H	-1.829000	-2.617000	5.158000
H	2.442000	-3.065000	5.165000
H	0.182000	-4.018000	5.543000

[Mg(PBTP)]2-1a-TS1

Mg	1.019000	0.071000	-0.707000
O	1.218000	-1.665000	0.188000
O	2.656000	0.856000	-1.122000
C	2.423000	-2.290000	-0.030000
C	3.621000	-1.627000	0.298000
C	4.812000	-2.167000	-0.193000
H	5.726000	-1.608000	-0.018000
C	4.876000	-3.362000	-0.896000
C	3.679000	-4.050000	-1.085000
H	3.707000	-5.003000	-1.592000
C	2.442000	-3.545000	-0.681000
C	3.807000	-0.382000	1.209000
C	2.563000	0.209000	1.898000
H	2.903000	0.919000	2.658000
H	1.952000	-0.548000	2.393000
H	1.927000	0.797000	1.241000
C	4.667000	-0.883000	2.397000
H	5.627000	-1.301000	2.091000
H	4.117000	-1.673000	2.918000
H	4.857000	-0.078000	3.114000
C	6.225000	-3.862000	-1.429000
C	6.774000	-2.832000	-2.436000
H	6.922000	-1.854000	-1.969000
H	6.079000	-2.702000	-3.272000
H	7.737000	-3.166000	-2.838000
C	6.103000	-5.217000	-2.140000
H	7.086000	-5.529000	-2.506000
H	5.429000	-5.164000	-3.001000
H	5.737000	-5.996000	-1.463000
C	7.222000	-4.017000	-0.266000
H	8.186000	-4.379000	-0.638000
H	6.849000	-4.735000	0.472000
H	7.398000	-3.066000	0.246000
C	1.153000	-4.329000	-1.013000
C	0.233000	-4.463000	0.217000
H	0.783000	-4.873000	1.071000
H	-0.590000	-5.148000	-0.011000
H	-0.204000	-3.511000	0.513000
C	0.405000	-3.619000	-2.154000
H	-0.527000	-4.145000	-2.390000
H	1.020000	-3.590000	-3.060000
H	0.148000	-2.597000	-1.877000
C	1.459000	-5.761000	-1.488000
H	2.028000	-6.326000	-0.742000
H	2.012000	-5.779000	-2.432000
H	0.515000	-6.287000	-1.658000
C	3.852000	1.281000	-0.747000
C	4.493000	0.733000	0.401000
C	5.750000	1.203000	0.766000
H	6.230000	0.781000	1.642000
C	6.429000	2.187000	0.048000
C	5.792000	2.694000	-1.081000
H	6.301000	3.450000	-1.663000
C	4.530000	2.270000	-1.506000
C	7.816000	2.649000	0.511000
C	7.710000	3.255000	1.924000
H	7.331000	2.525000	2.646000
H	8.691000	3.595000	2.274000
H	7.030000	4.113000	1.925000
C	8.418000	3.710000	-0.421000
H	8.543000	3.328000	-1.439000
H	7.795000	4.610000	-0.466000
H	9.405000	4.007000	-0.053000
C	8.776000	1.444000	0.546000
H	8.865000	0.992000	-0.447000
H	9.775000	1.757000	0.871000
H	8.429000	0.671000	1.237000
C	3.912000	2.842000	-2.796000
C	2.570000	3.536000	-2.498000
H	2.707000	4.345000	-1.772000
H	1.848000	2.831000	-2.088000
H	2.155000	3.970000	-3.415000
C	4.820000	3.888000	-3.465000
H	4.333000	4.257000	-4.374000
H	5.787000	3.467000	-3.757000
H	5.001000	4.751000	-2.815000
C	3.697000	1.707000	-3.818000
H	3.036000	0.941000	-3.411000
H	4.653000	1.238000	-4.074000
H	3.256000	2.105000	-4.739000
Mg	-1.663000	-0.459000	1.353000
O	-0.629000	0.732000	0.107000
O	-3.272000	-0.958000	0.600000
O	-0.244000	-1.612000	2.026000
C	-1.553000	1.728000	-0.115000
C	-2.646000	1.489000	-0.974000
C	-3.678000	2.429000	-0.991000
H	-4.551000	2.201000	-1.595000
C	-3.660000	3.604000	-0.255000
C	-2.535000	3.837000	0.532000
H	-2.489000	4.753000	1.103000
C	-1.481000	2.928000	0.642000
C	-2.875000	0.291000	-1.936000
C	-1.779000	-0.786000	-2.047000
H	-2.094000	-1.513000	-2.801000
H	-0.841000	-0.357000	-2.420000
H	-1.612000	-1.341000	-1.129000
C	-2.942000	0.899000	-3.359000
H	-3.731000	1.644000	-3.475000
H	-1.991000	1.396000	-3.576000
H	-3.093000	0.120000	-4.112000
C	-4.859000	4.558000	-0.314000
C	-6.096000	3.839000	0.258000
H	-6.328000	2.927000	-0.302000
H	-5.930000	3.558000	1.303000
H	-6.973000	4.494000	0.215000
C	-4.623000	5.839000	0.497000
H	-5.496000	6.493000	0.415000
H	-4.470000	5.625000	1.560000
H	-3.754000	6.394000	0.129000
C	-5.133000	4.958000	-1.776000
H	-5.978000	5.653000	-1.827000
H	-4.259000	5.450000	-2.215000
H	-5.379000	4.091000	-2.396000
C	-0.295000	3.252000	1.576000
C	1.000000	3.387000	0.756000
H	0.919000	4.227000	0.059000
H	1.856000	3.572000	1.414000
H	1.233000	2.502000	0.165000
C	-0.148000	2.185000	2.678000
H	0.662000	2.454000	3.364000
H	-1.067000	2.121000	3.273000
H	0.090000	1.199000	2.282000
C	-0.481000	4.594000	2.309000
H	-0.555000	5.436000	1.615000
H	-1.366000	4.593000	2.954000
H	0.391000	4.771000	2.946000
C	-4.289000	-1.003000	-0.240000
C	-4.183000	-0.407000	-1.526000
C	-5.276000	-0.452000	-2.385000
H	-5.190000	0.011000	-3.362000
C	-6.485000	-1.056000	-2.039000
C	-6.559000	-1.647000	-0.780000
H	-7.483000	-2.128000	-0.491000
C	-5.499000	-1.644000	0.130000
C	-7.657000	-1.034000	-3.029000
C	-7.252000	-1.760000	-4.326000
H	-6.381000	-1.293000	-4.795000
H	-8.073000	-1.740000	-5.052000
H	-7.003000	-2.806000	-4.121000
C	-8.908000	-1.722000	-2.467000
H	-9.264000	-1.234000	-1.554000
H	-8.723000	-2.778000	-2.243000
H	-9.715000	-1.677000	-3.206000
C	-8.022000	0.427000	-3.357000
H	-8.313000	0.965000	-2.449000
H	-8.859000	0.465000	-4.063000
H	-7.180000	0.961000	-3.809000
C	-5.641000	-2.309000	1.512000

C	-4.578000	-3.413000	1.681000
H	-4.716000	-4.196000	0.927000
H	-3.573000	-3.006000	1.573000
H	-4.670000	-3.879000	2.670000
C	-7.016000	-2.967000	1.708000
H	-7.055000	-3.431000	2.699000
H	-7.831000	-2.238000	1.653000
H	-7.204000	-3.751000	0.967000
C	-5.480000	-1.249000	2.619000
H	-4.514000	-0.750000	2.538000
H	-6.268000	-0.492000	2.542000
H	-5.553000	-1.717000	3.608000
C	-0.125000	-2.322000	3.247000
H	-0.529000	-3.335000	3.131000
H	0.937000	-2.410000	3.508000
H	0.575000	-1.761000	1.202000
C	-0.872000	-1.570000	4.320000
C	-0.273000	-0.478000	4.955000
C	-2.208000	-1.870000	4.601000
C	-0.994000	0.297000	5.857000
H	0.763000	-0.234000	4.734000
C	-2.934000	-1.092000	5.501000
H	-2.686000	-2.712000	4.105000
C	-2.327000	-0.007000	6.129000
H	-0.519000	1.143000	6.344000
H	-3.972000	-1.331000	5.708000
H	-2.891000	0.600000	6.829000

[Mg(PBTP)]2-1a-Int1

Mg	1.005000	-0.786000	0.933000
O	2.256000	-2.515000	0.963000
O	2.391000	0.291000	0.212000
C	3.302000	-2.577000	0.043000
C	4.391000	-1.746000	0.288000
C	5.346000	-1.662000	-0.726000
H	6.154000	-0.950000	-0.598000
C	5.274000	-2.393000	-1.905000
C	4.189000	-3.253000	-2.064000
H	4.115000	-3.834000	-2.972000
C	3.166000	-3.353000	-1.120000
C	4.639000	-0.839000	1.522000
C	3.677000	-0.942000	2.727000
H	4.143000	-0.421000	3.569000
H	3.487000	-1.974000	3.027000
H	2.729000	-0.431000	2.561000
C	6.010000	-1.265000	2.098000
H	6.821000	-1.211000	1.370000
H	5.942000	-2.301000	2.445000
H	6.285000	-0.644000	2.956000
C	6.336000	-2.180000	-2.991000
C	6.213000	-0.736000	-3.519000
H	6.348000	-0.001000	-2.719000
H	5.225000	-0.570000	-3.960000
H	6.970000	-0.545000	-4.288000
C	6.162000	-3.144000	-4.173000
H	6.948000	-2.964000	-4.912000
H	5.200000	-3.002000	-4.676000
H	6.235000	-4.190000	-3.856000
C	7.742000	-2.392000	-2.401000
H	8.502000	-2.258000	-3.178000
H	7.846000	-3.403000	-1.993000
H	7.960000	-1.680000	-1.600000
C	1.929000	-4.241000	-1.383000
C	1.860000	-5.400000	-0.365000
H	2.740000	-6.043000	-0.456000
H	0.968000	-6.006000	-0.547000
H	1.812000	-5.085000	0.685000
C	0.626000	-3.406000	-1.344000
H	-0.183000	-3.953000	-1.837000
H	0.758000	-2.459000	-1.877000
H	0.275000	-3.186000	-0.333000
C	1.992000	-4.891000	-2.776000
H	2.872000	-5.530000	-2.893000
H	1.997000	-4.139000	-3.571000
H	1.109000	-5.520000	-2.917000
C	3.485000	1.031000	0.239000
C	4.628000	0.593000	0.969000
C	5.751000	1.405000	1.055000
H	6.609000	1.052000	1.617000
C	5.832000	2.642000	0.415000
C	4.743000	3.011000	-0.370000
H	4.800000	3.943000	-0.916000
C	3.586000	2.238000	-0.504000
C	7.108000	3.484000	0.534000
C	7.416000	3.760000	2.018000
H	7.566000	2.832000	2.578000
H	8.329000	4.359000	2.116000
H	6.593000	4.307000	2.488000
C	6.984000	4.835000	-0.184000
H	6.824000	4.709000	-1.259000
H	6.158000	5.430000	0.218000
H	7.907000	5.408000	-0.051000
C	8.285000	2.713000	-0.095000
H	8.093000	2.514000	-1.154000
H	9.214000	3.290000	-0.017000
H	8.445000	1.751000	0.403000
C	2.483000	2.659000	-1.492000
C	1.163000	2.928000	-0.755000
H	1.297000	3.686000	0.022000
H	0.789000	2.020000	-0.287000
H	0.401000	3.296000	-1.451000
C	2.843000	3.936000	-2.268000
H	2.044000	4.158000	-2.982000
H	3.773000	3.826000	-2.835000
H	2.943000	4.804000	-1.607000
C	2.281000	1.537000	-2.531000
H	2.023000	0.597000	-2.041000
H	3.199000	1.383000	-3.110000
H	1.479000	1.804000	-3.230000
Mg	-1.948000	-1.130000	1.333000
O	-0.649000	0.307000	0.936000
O	-3.427000	-1.209000	0.224000
O	-0.309000	-2.092000	1.775000
C	-1.385000	1.439000	0.842000
C	-2.237000	1.625000	-0.268000
C	-3.128000	2.698000	-0.229000
H	-3.829000	2.800000	-1.051000
C	-3.181000	3.616000	0.811000
C	-2.281000	3.436000	1.860000
H	-2.296000	4.144000	2.675000
C	-1.386000	2.367000	1.918000
C	-2.306000	0.766000	-1.566000
C	-1.278000	-0.376000	-1.739000
H	-1.346000	-0.735000	-2.772000
H	-0.259000	-0.013000	-1.580000
H	-1.465000	-1.241000	-1.105000
C	-1.959000	1.726000	-2.731000
H	-2.574000	2.628000	-2.754000
H	-0.919000	2.041000	-2.623000
H	-2.059000	1.219000	-3.696000
C	-4.205000	4.757000	0.763000
C	-5.624000	4.166000	0.644000
H	-5.741000	3.567000	-0.264000
H	-5.848000	3.523000	1.501000
H	-6.369000	4.969000	0.613000
C	-4.157000	5.638000	2.018000
H	-4.911000	6.427000	1.942000
H	-4.368000	5.062000	2.925000
H	-3.182000	6.122000	2.138000
C	-3.919000	5.645000	-0.463000
H	-4.647000	6.462000	-0.522000
H	-2.918000	6.083000	-0.399000
H	-3.979000	5.075000	-1.395000
C	-0.459000	2.205000	3.144000
C	1.021000	2.309000	2.730000
H	1.255000	3.328000	2.406000
H	1.670000	2.072000	3.581000
H	1.284000	1.648000	1.902000
C	-0.710000	0.862000	3.865000
H	-0.197000	0.853000	4.833000
H	-1.780000	0.716000	4.056000
H	-0.341000	0.011000	3.292000
C	-0.697000	3.305000	4.196000
H	-0.526000	4.306000	3.788000
H	-1.709000	3.266000	4.612000
H	0.008000	3.164000	5.021000
C	-4.210000	-0.764000	-0.745000
C	-3.735000	0.198000	-1.681000
C	-4.607000	0.663000	-2.661000
H	-4.251000	1.410000	-3.362000
C	-5.928000	0.231000	-2.774000
C	-6.358000	-0.730000	-1.863000
H	-7.375000	-1.090000	-1.935000
C	-5.540000	-1.246000	-0.855000
C	-6.839000	0.837000	-3.849000
C	-6.231000	0.603000	-5.244000
H	-5.246000	1.071000	-5.340000
H	-6.877000	1.026000	-6.021000
H	-6.115000	-0.467000	-5.440000
C	-8.246000	0.224000	-3.833000
H	-8.750000	0.391000	-2.876000
H	-8.218000	-0.855000	-4.022000
H	-8.858000	0.683000	-4.616000
C	-6.975000	2.353000	-3.602000
H	-7.413000	2.545000	-2.617000
H	-7.621000	2.811000	-4.360000
H	-6.004000	2.855000	-3.641000
C	-6.079000	-2.308000	0.123000
C	-5.242000	-3.598000	0.013000
H	-5.308000	-4.011000	-1.000000
H	-4.194000	-3.404000	0.243000
H	-5.618000	-4.354000	0.712000
C	-7.538000	-2.691000	-0.175000
H	-7.863000	-3.449000	0.545000
H	-8.216000	-1.836000	-0.081000
H	-7.656000	-3.117000	-1.177000
C	-6.040000	-1.779000	1.570000
H	-5.020000	-1.539000	1.870000
H	-6.652000	-0.876000	1.663000
H	-6.434000	-2.533000	2.262000
C	-0.270000	-3.239000	2.586000
H	-0.057000	-4.139000	1.984000
H	0.528000	-3.148000	3.336000
H	1.811000	-3.364000	1.037000
C	-1.604000	-3.425000	3.277000
C	-1.934000	-2.637000	4.388000
C	-2.566000	-4.295000	2.756000
C	-3.199000	-2.719000	4.962000
H	-1.191000	-1.959000	4.801000
C	-3.831000	-4.382000	3.334000
H	-2.322000	-4.905000	1.890000
C	-4.149000	-3.593000	4.435000
H	-3.442000	-2.107000	5.825000
H	-4.568000	-5.061000	2.919000
H	-5.135000	-3.659000	4.883000

[Mg(PBTP)]2-1a-MA-Int2

Mg	-0.955000	-0.484000	0.288000
O	-2.300000	-1.989000	-0.550000
O	-2.469000	0.528000	0.803000
C	-3.658000	-2.159000	-0.238000
C	-4.552000	-1.123000	-0.526000
C	-5.829000	-1.214000	0.031000
H	-6.497000	-0.371000	-0.101000
C	-6.267000	-2.310000	0.762000
C	-5.370000	-3.361000	0.930000
H	-5.694000	-4.237000	1.472000
C	-4.053000	-3.312000	0.468000
C	-4.296000	0.129000	-1.395000
C	-3.012000	0.139000	-2.245000
H	-3.100000	0.933000	-2.991000
H	-2.861000	-0.799000	-2.786000
H	-2.114000	0.383000	-1.686000
C	-5.421000	0.151000	-2.461000
H	-6.429000	0.178000	-2.045000
H	-5.343000	-0.751000	-3.077000
H	-5.307000	1.016000	-3.121000
C	-7.684000	-2.316000	1.347000
C	-7.831000	-1.126000	2.316000

H	-7.666000	-0.169000	1.812000
H	-7.109000	-1.203000	3.135000
H	-8.838000	-1.111000	2.746000
C	-7.991000	-3.607000	2.118000
H	-9.008000	-3.561000	2.518000
H	-7.308000	-3.747000	2.962000
H	-7.928000	-4.489000	1.473000
C	-8.711000	-2.178000	0.207000
H	-9.729000	-2.179000	0.611000
H	-8.622000	-3.010000	-0.499000
H	-8.574000	-1.246000	-0.350000
C	-3.088000	-4.459000	0.825000
C	-2.301000	-4.953000	-0.405000
H	-2.990000	-5.269000	-1.196000
H	-1.685000	-5.816000	-0.131000
H	-1.627000	-4.199000	-0.810000
C	-2.151000	-3.961000	1.942000
H	-1.391000	-4.714000	2.179000
H	-2.730000	-3.760000	2.849000
H	-1.642000	-3.040000	1.660000
C	-3.828000	-5.696000	1.369000
H	-4.563000	-6.078000	0.654000
H	-4.335000	-5.496000	2.317000
H	-3.098000	-6.489000	1.558000
C	-3.397000	1.451000	0.610000
C	-4.329000	1.350000	-0.465000
C	-5.285000	2.343000	-0.639000
H	-5.988000	2.254000	-1.460000
C	-5.385000	3.452000	0.201000
C	-4.474000	3.528000	1.249000
H	-4.532000	4.375000	1.919000
C	-3.488000	2.567000	1.484000
C	-6.469000	4.507000	-0.053000
C	-6.284000	5.110000	-1.459000
H	-6.360000	4.346000	-2.239000
H	-7.050000	5.868000	-1.657000
H	-5.300000	5.583000	-1.548000
C	-6.417000	5.653000	0.966000
H	-6.575000	5.292000	1.988000
H	-5.458000	6.179000	0.933000
H	-7.205000	6.380000	0.745000
C	-7.859000	3.848000	0.038000
H	-8.015000	3.412000	1.030000
H	-8.649000	4.586000	-0.141000
H	-7.975000	3.049000	-0.701000
C	-2.504000	2.734000	2.657000
C	-1.075000	2.949000	2.122000
H	-1.014000	3.874000	1.539000
H	-0.754000	2.133000	1.475000
H	-0.369000	3.029000	2.957000
C	-2.841000	3.951000	3.535000
H	-2.126000	4.005000	4.362000
H	-3.845000	3.878000	3.967000
H	-2.771000	4.892000	2.980000
C	-2.544000	1.496000	3.575000
H	-2.284000	0.594000	3.023000
H	-3.547000	1.365000	3.996000
H	-1.841000	1.621000	4.408000
Mg	1.666000	-0.833000	-1.011000
O	0.507000	0.723000	-0.465000
O	3.457000	-0.366000	-0.741000
O	0.461000	-1.902000	0.136000
C	1.291000	1.838000	-0.549000
C	2.396000	2.006000	0.325000
C	3.333000	2.994000	0.023000
H	4.224000	3.040000	0.642000
C	3.218000	3.875000	-1.039000
C	2.066000	3.765000	-1.808000
H	1.923000	4.461000	-2.622000
C	1.098000	2.780000	-1.603000
C	2.762000	1.207000	1.609000
C	1.731000	0.199000	2.143000
H	2.115000	-0.215000	3.081000
H	0.785000	0.702000	2.369000
H	1.540000	-0.634000	1.475000
C	2.853000	2.264000	2.744000
H	3.684000	2.960000	2.623000
H	1.927000	2.845000	2.757000
H	2.955000	1.786000	3.723000
C	4.336000	4.884000	-1.324000
C	5.626000	4.113000	-1.664000
H	5.926000	3.449000	-0.847000
H	5.480000	3.495000	-2.556000
H	6.451000	4.808000	-1.858000
C	4.001000	5.806000	-2.504000
H	4.824000	6.509000	-2.669000
H	3.857000	5.241000	-3.431000
H	3.095000	6.391000	-2.315000
C	4.578000	5.762000	-0.082000
H	5.378000	6.486000	-0.275000
H	3.671000	6.316000	0.183000
H	4.872000	5.164000	0.785000
C	-0.128000	2.762000	-2.541000
C	-1.397000	2.904000	-1.690000
H	-1.411000	3.880000	-1.194000
H	-2.302000	2.828000	-2.302000
H	-1.448000	2.145000	-0.913000
C	-0.153000	1.477000	-3.391000
H	-1.006000	1.491000	-4.078000
H	0.761000	1.409000	-3.993000
H	-0.226000	0.577000	-2.786000
C	-0.132000	3.933000	-3.540000
H	-0.115000	4.905000	-3.037000
H	0.709000	3.889000	-4.240000
H	-1.053000	3.885000	-4.130000
C	4.404000	-0.152000	0.147000
C	4.120000	0.506000	1.378000
C	5.109000	0.547000	2.356000
H	4.875000	1.011000	3.309000
C	6.394000	0.037000	2.163000
C	6.689000	-0.456000	0.894000
H	7.699000	-0.786000	0.688000
C	5.744000	-0.535000	-0.131000
C	7.416000	0.088000	3.305000
C	6.876000	-0.702000	4.512000
H	5.930000	-0.288000	4.873000
H	7.591000	-0.677000	5.343000
H	6.702000	-1.749000	4.241000
C	8.767000	-0.521000	2.905000
H	9.230000	0.027000	2.078000
H	8.663000	-1.569000	2.604000
H	9.456000	-0.484000	3.755000
C	7.658000	1.551000	3.725000
H	8.045000	2.134000	2.883000
H	8.387000	1.603000	4.542000
H	6.736000	2.030000	4.068000
C	6.152000	-0.949000	-1.557000
C	5.442000	-2.240000	-2.002000
H	5.808000	-3.099000	-1.433000
H	4.366000	-2.168000	-1.843000
H	5.638000	-2.431000	-3.065000
C	7.663000	-1.201000	-1.685000
H	7.899000	-1.458000	-2.723000
H	8.250000	-0.314000	-1.421000
H	7.996000	-2.034000	-1.056000
C	5.803000	0.205000	-2.520000
H	4.738000	0.438000	-2.478000
H	6.360000	1.107000	-2.247000
H	6.070000	-0.063000	-3.550000
C	0.701000	-3.030000	0.936000
H	0.680000	-2.748000	2.001000
H	-0.092000	-3.772000	0.797000
H	-2.046000	-2.375000	-1.399000
C	2.026000	-3.702000	0.652000
C	2.075000	-4.895000	-0.073000
C	3.221000	-3.153000	1.126000
C	3.289000	-5.535000	-0.311000
H	1.149000	-5.335000	-0.436000
C	4.435000	-3.788000	0.892000
H	3.207000	-2.222000	1.684000
C	4.471000	-4.985000	0.178000
H	3.312000	-6.466000	-0.869000
H	5.353000	-3.335000	1.254000
H	5.418000	-5.483000	-0.002000
C	0.472000	-2.891000	-2.883000
O	0.774000	-1.722000	-2.649000
O	-0.801000	-3.231000	-2.727000
C	-1.293000	-4.507000	-3.186000
H	-0.937000	-5.309000	-2.537000
H	-2.376000	-4.435000	-3.116000
H	-1.000000	-4.679000	-4.221000
C	1.460000	-3.894000	-3.313000
C	2.660000	-3.496000	-3.740000
H	1.203000	-4.945000	-3.268000
H	3.412000	-4.216000	-4.042000
H	2.917000	-2.444000	-3.800000

[Mg(PBTP)]2-1a-MA-TS2

Mg	-0.914000	-0.498000	-0.048000
O	-1.955000	-2.255000	-0.409000
O	-2.397000	0.358000	0.747000
C	-3.294000	-2.434000	-0.010000
C	-4.254000	-1.504000	-0.420000
C	-5.511000	-1.589000	0.182000
H	-6.236000	-0.821000	-0.060000
C	-5.854000	-2.562000	1.108000
C	-4.886000	-3.513000	1.420000
H	-5.134000	-4.294000	2.122000
C	-3.593000	-3.472000	0.895000
C	-4.104000	-0.340000	-1.430000
C	-2.824000	-0.278000	-2.291000
H	-3.010000	0.404000	-3.125000
H	-2.536000	-1.240000	-2.716000
H	-1.964000	0.178000	-1.800000
C	-5.222000	-0.537000	-2.485000
H	-6.227000	-0.556000	-2.062000
H	-5.060000	-1.490000	-2.998000
H	-5.189000	0.256000	-3.237000
C	-7.246000	-2.536000	1.751000
C	-7.441000	-1.184000	2.465000
H	-7.376000	-0.342000	1.770000
H	-6.678000	-1.040000	3.237000
H	-8.426000	-1.147000	2.943000
C	-7.433000	-3.656000	2.782000
H	-8.431000	-3.585000	3.224000
H	-6.703000	-3.582000	3.595000
H	-7.341000	-4.647000	2.326000
C	-8.319000	-2.698000	0.657000
H	-9.319000	-2.667000	1.101000
H	-8.203000	-3.656000	0.140000
H	-8.258000	-1.901000	-0.090000
C	-2.558000	-4.517000	1.349000
C	-1.988000	-5.263000	0.129000
H	-2.783000	-5.815000	-0.383000
H	-1.223000	-5.982000	0.445000
H	-1.531000	-4.591000	-0.594000
C	-1.455000	-3.822000	2.170000
H	-0.669000	-4.535000	2.439000
H	-1.878000	-3.414000	3.093000
H	-0.998000	-3.001000	1.621000
C	-3.169000	-5.596000	2.262000
H	-3.978000	-6.142000	1.767000
H	-3.552000	-5.178000	3.197000
H	-2.391000	-6.319000	2.523000
C	-3.387000	1.198000	0.483000
C	-4.261000	0.954000	-0.615000
C	-5.261000	1.870000	-0.914000
H	-5.915000	1.672000	-1.757000
C	-5.464000	3.032000	-0.170000
C	-4.625000	3.232000	0.923000
H	-4.769000	4.118000	1.525000
C	-3.600000	2.352000	1.280000
C	-6.576000	4.009000	-0.569000
C	-6.328000	4.507000	-2.006000
H	-6.331000	3.682000	-2.725000
H	-7.107000	5.217000	-2.308000
H	-5.358000	5.010000	-2.078000
C	-6.633000	5.234000	0.354000
H	-6.841000	4.951000	1.391000
H	-5.695000	5.797000	0.334000

H	-7.432000	5.906000	0.026000
C	-7.939000	3.295000	-0.506000
H	-8.136000	2.928000	0.507000
H	-8.747000	3.980000	-0.787000
H	-7.976000	2.438000	-1.186000
C	-2.700000	2.652000	2.493000
C	-1.255000	2.904000	2.026000
H	-1.208000	3.735000	1.315000
H	-0.837000	2.026000	1.534000
H	-0.619000	3.158000	2.883000
C	-3.152000	3.905000	3.262000
H	-2.494000	4.050000	4.125000
H	-4.176000	3.807000	3.639000
H	-3.094000	4.810000	2.650000
C	-2.730000	1.475000	3.487000
H	-2.394000	0.555000	3.008000
H	-3.746000	1.318000	3.865000
H	-2.079000	1.688000	4.343000
Mg	1.708000	-0.383000	-1.760000
O	0.418000	0.758000	-0.740000
O	3.368000	0.050000	-1.027000
O	0.881000	-2.185000	-1.041000
C	1.055000	1.942000	-0.468000
C	2.042000	2.001000	0.539000
C	2.821000	3.154000	0.634000
H	3.628000	3.155000	1.360000
C	2.654000	4.264000	-0.180000
C	1.624000	4.203000	-1.112000
H	1.453000	5.060000	-1.746000
C	0.818000	3.076000	-1.290000
C	2.426000	0.902000	1.562000
C	1.504000	-0.329000	1.638000
H	1.844000	-0.979000	2.450000
H	0.482000	-0.013000	1.897000
H	1.516000	-0.924000	0.730000
C	2.293000	1.539000	2.969000
H	2.997000	2.354000	3.141000
H	1.282000	1.939000	3.082000
H	2.444000	0.792000	3.755000
C	3.595000	5.464000	-0.034000
C	5.043000	5.001000	-0.287000
H	5.354000	4.234000	0.428000
H	5.144000	4.580000	-1.292000
H	5.737000	5.845000	-0.196000
C	3.270000	6.585000	-1.031000
H	3.974000	7.412000	-0.895000
H	3.358000	6.241000	-2.067000
H	2.260000	6.980000	-0.883000
C	3.482000	6.038000	1.391000
H	4.155000	6.894000	1.514000
H	2.459000	6.372000	1.593000
H	3.748000	5.292000	2.146000
C	-0.298000	3.119000	-2.355000
C	-1.655000	3.000000	-1.647000
H	-1.862000	3.896000	-1.054000
H	-2.470000	2.878000	-2.369000
H	-1.675000	2.154000	-0.966000
C	-0.133000	1.997000	-3.397000
H	-0.875000	2.116000	-4.194000
H	0.860000	2.047000	-3.862000
H	-0.272000	1.008000	-2.969000
C	-0.312000	4.440000	-3.145000
H	-0.478000	5.307000	-2.499000
H	0.614000	4.596000	-3.707000
H	-1.136000	4.409000	-3.865000
C	4.262000	0.112000	-0.064000
C	3.872000	0.450000	1.265000
C	4.820000	0.382000	2.281000
H	4.514000	0.615000	3.295000
C	6.152000	0.034000	2.055000
C	6.527000	-0.200000	0.734000
H	7.564000	-0.426000	0.525000
C	5.630000	-0.155000	-0.335000
C	7.131000	-0.046000	3.233000
C	6.618000	-1.076000	4.258000
H	5.638000	-0.796000	4.656000
H	7.312000	-1.156000	5.103000
H	6.522000	-2.064000	3.796000
C	8.537000	-0.475000	2.792000
H	8.978000	0.242000	2.092000
H	8.526000	-1.460000	2.312000
H	9.196000	-0.536000	3.664000
C	7.242000	1.334000	3.909000
H	7.600000	2.084000	3.196000
H	7.944000	1.297000	4.750000
H	6.275000	1.673000	4.294000
C	6.116000	-0.350000	-1.784000
C	5.470000	-1.591000	-2.430000
H	5.803000	-2.507000	-1.932000
H	4.383000	-1.552000	-2.359000
H	5.754000	-1.657000	-3.488000
C	7.640000	-0.541000	-1.869000
H	7.929000	-0.653000	-2.919000
H	8.183000	0.319000	-1.464000
H	7.971000	-1.441000	-1.339000
C	5.770000	0.910000	-2.603000
H	4.695000	1.092000	-2.600000
H	6.268000	1.789000	-2.180000
H	6.108000	0.794000	-3.640000
C	1.104000	-3.324000	-0.224000
H	0.834000	-3.061000	0.803000
H	0.404000	-4.109000	-0.529000
H	-1.713000	-2.671000	-1.261000
C	2.505000	-3.890000	-0.190000
C	2.725000	-5.238000	-0.483000
C	3.580000	-3.113000	0.240000
C	3.996000	-5.795000	-0.361000
H	1.892000	-5.862000	-0.801000
C	4.851000	-3.664000	0.364000
H	3.432000	-2.069000	0.483000
C	5.063000	-5.007000	0.063000
H	4.150000	-6.844000	-0.592000
H	5.669000	-3.030000	0.693000
H	6.053000	-5.440000	0.162000
C	0.514000	-2.455000	-2.793000
O	0.515000	-1.237000	-3.159000
O	-0.732000	-3.055000	-2.695000
C	-0.953000	-4.329000	-3.314000
H	-0.478000	-5.138000	-2.753000
H	-2.033000	-4.472000	-3.307000
H	-0.591000	-4.325000	-4.344000
C	1.629000	-3.357000	-3.225000
C	2.520000	-2.959000	-4.128000
H	1.698000	-4.339000	-2.773000
H	3.338000	-3.604000	-4.429000
H	2.449000	-1.979000	-4.588000

[Mg(PBTP)]2-1a-MA-Int3

Mg	-1.229000	0.041000	0.262000
O	-1.571000	-1.898000	-0.146000
O	-2.883000	0.728000	0.803000
C	-2.842000	-2.335000	0.261000
C	-3.965000	-1.701000	-0.281000
C	-5.203000	-2.025000	0.277000
H	-6.074000	-1.489000	-0.086000
C	-5.362000	-2.948000	1.300000
C	-4.215000	-3.581000	1.773000
H	-4.315000	-4.309000	2.563000
C	-2.937000	-3.290000	1.292000
C	-4.034000	-0.640000	-1.414000
C	-2.737000	-0.233000	-2.145000
H	-3.018000	0.285000	-3.066000
H	-2.087000	-1.062000	-2.422000
H	-2.153000	0.521000	-1.616000
C	-4.906000	-1.267000	-2.528000
H	-5.895000	-1.574000	-2.183000
H	-4.399000	-2.156000	-2.918000
H	-5.037000	-0.569000	-3.360000
C	-6.759000	-3.200000	1.879000
C	-7.299000	-1.881000	2.466000
H	-7.378000	-1.100000	1.704000
H	-6.638000	-1.511000	3.256000
H	-8.295000	-2.036000	2.895000
C	-6.746000	-4.257000	2.991000
H	-7.762000	-4.404000	3.368000
H	-6.121000	-3.950000	3.835000
H	-6.383000	-5.223000	2.626000
C	-7.699000	-3.685000	0.759000
H	-8.701000	-3.869000	1.161000
H	-7.330000	-4.617000	0.318000
H	-7.791000	-2.945000	-0.041000
C	-1.715000	-4.023000	1.885000
C	-1.142000	-4.982000	0.825000
H	-1.884000	-5.745000	0.567000
H	-0.254000	-5.491000	1.216000
H	-0.861000	-4.466000	-0.094000
C	-0.633000	-3.032000	2.357000
H	0.164000	-3.571000	2.880000
H	-1.052000	-2.298000	3.053000
H	-0.183000	-2.501000	1.521000
C	-2.091000	-4.873000	3.112000
H	-2.797000	-5.670000	2.863000
H	-2.519000	-4.265000	3.915000
H	-1.187000	-5.351000	3.500000
C	-4.033000	1.207000	0.349000
C	-4.656000	0.620000	-0.788000
C	-5.843000	1.157000	-1.271000
H	-6.311000	0.698000	-2.135000
C	-6.468000	2.252000	-0.673000
C	-5.865000	2.779000	0.466000
H	-6.346000	3.611000	0.962000
C	-4.676000	2.283000	1.009000
C	-7.785000	2.787000	-1.250000
C	-7.592000	3.160000	-2.732000
H	-7.290000	2.297000	-3.332000
H	-8.525000	3.550000	-3.154000
H	-6.820000	3.929000	-2.838000
C	-8.281000	4.035000	-0.507000
H	-8.496000	3.824000	0.545000
H	-7.547000	4.846000	-0.550000
H	-9.206000	4.396000	-0.968000
C	-8.869000	1.697000	-1.135000
H	-9.033000	1.427000	-0.087000
H	-9.819000	2.052000	-1.551000
H	-8.584000	0.789000	-1.676000
C	-4.110000	2.857000	2.322000
C	-2.717000	3.476000	2.100000
H	-2.775000	4.311000	1.395000
H	-2.015000	2.743000	1.701000
H	-2.320000	3.861000	3.047000
C	-5.005000	3.960000	2.909000
H	-4.566000	4.319000	3.845000
H	-6.013000	3.594000	3.133000
H	-5.092000	4.820000	2.236000
C	-4.021000	1.735000	3.376000
H	-3.364000	0.934000	3.036000
H	-5.012000	1.309000	3.567000
H	-3.634000	2.135000	4.321000
Mg	1.600000	-0.257000	-1.536000
O	0.369000	0.879000	-0.425000
O	3.328000	0.231000	-1.047000
O	1.436000	-2.481000	-1.185000
C	1.042000	2.057000	-0.183000
C	2.106000	2.072000	0.744000
C	2.859000	3.241000	0.854000
H	3.717000	3.225000	1.517000
C	2.599000	4.394000	0.128000
C	1.533000	4.347000	-0.763000
H	1.314000	5.229000	-1.346000
C	0.753000	3.206000	-0.965000
C	2.616000	0.915000	1.648000
C	1.772000	-0.371000	1.729000
H	2.251000	-1.048000	2.444000
H	0.781000	-0.152000	2.139000
H	1.671000	-0.928000	0.798000
C	2.575000	1.450000	3.103000
H	3.246000	2.293000	3.274000
H	1.558000	1.784000	3.331000
H	2.835000	0.665000	3.820000
C	3.492000	5.626000	0.315000

C	4.943000	5.261000	-0.051000
H	5.328000	4.451000	0.576000
H	5.009000	4.934000	-1.094000
H	5.600000	6.128000	0.080000
C	3.053000	6.804000	-0.565000
H	3.713000	7.659000	-0.389000
H	3.109000	6.556000	-1.630000
H	2.030000	7.120000	-0.338000
C	3.434000	6.078000	1.787000
H	4.069000	6.958000	1.942000
H	2.410000	6.339000	2.071000
H	3.781000	5.292000	2.465000
C	-0.361000	3.250000	-2.038000
C	-1.742000	3.193000	-1.363000
H	-1.924000	4.116000	-0.806000
H	-2.538000	3.086000	-2.109000
H	-1.841000	2.376000	-0.651000
C	-0.200000	2.118000	-3.073000
H	-0.945000	2.230000	-3.868000
H	0.791000	2.166000	-3.540000
H	-0.338000	1.127000	-2.645000
C	-0.333000	4.557000	-2.855000
H	-0.516000	5.440000	-2.237000
H	0.617000	4.691000	-3.382000
H	-1.128000	4.518000	-3.605000
C	4.311000	0.271000	-0.174000
C	4.047000	0.557000	1.196000
C	5.096000	0.519000	2.108000
H	4.888000	0.717000	3.154000
C	6.414000	0.255000	1.734000
C	6.656000	0.053000	0.376000
H	7.674000	-0.128000	0.057000
C	5.651000	0.057000	-0.591000
C	7.519000	0.227000	2.798000
C	7.194000	-0.844000	3.856000
H	6.236000	-0.650000	4.348000
H	7.969000	-0.868000	4.630000
H	7.140000	-1.837000	3.398000
C	8.894000	-0.100000	2.200000
H	9.207000	0.651000	1.467000
H	8.898000	-1.079000	1.710000
H	9.647000	-0.121000	2.994000
C	7.610000	1.605000	3.483000
H	7.847000	2.383000	2.750000
H	8.394000	1.604000	4.249000
H	6.667000	1.875000	3.967000
C	5.984000	-0.145000	-2.081000
C	5.277000	-1.388000	-2.655000
H	5.588000	-2.293000	-2.126000
H	4.194000	-1.307000	-2.574000
H	5.535000	-1.507000	-3.715000
C	7.490000	-0.344000	-2.316000
H	7.673000	-0.469000	-3.388000
H	8.078000	0.516000	-1.978000
H	7.866000	-1.240000	-1.811000
C	5.557000	1.110000	-2.870000
H	4.489000	1.295000	-2.747000
H	6.102000	1.991000	-2.515000
H	5.774000	0.981000	-3.937000
C	1.872000	-3.641000	-0.431000
H	1.490000	-3.479000	0.579000
H	1.374000	-4.521000	-0.836000
H	-1.154000	-2.451000	-0.879000
C	3.367000	-3.809000	-0.399000
C	4.001000	-4.778000	-1.180000
C	4.136000	-3.034000	0.470000
C	5.377000	-4.968000	-1.092000
H	3.414000	-5.401000	-1.849000
C	5.512000	-3.217000	0.558000
H	3.664000	-2.276000	1.085000
C	6.134000	-4.187000	-0.222000
H	5.856000	-5.729000	-1.700000
H	6.091000	-2.588000	1.225000
H	7.207000	-4.336000	-0.152000
C	0.831000	-2.586000	-2.343000
O	0.594000	-1.351000	-2.791000
O	-0.450000	-3.273000	-2.015000
C	-1.294000	-3.553000	-3.124000
H	-0.923000	-4.413000	-3.688000
H	-2.281000	-3.786000	-2.717000
H	-1.363000	-2.686000	-3.787000
C	1.565000	-3.482000	-3.338000
C	2.117000	-3.010000	-4.448000
H	1.628000	-4.540000	-3.096000
H	2.660000	-3.656000	-5.130000
H	2.036000	-1.956000	-4.695000

[Mg(PBTP)]2-1a-MA-TS3

Mg	-1.293000	-0.005000	0.274000
O	-1.490000	-1.890000	-0.194000
O	-2.904000	0.811000	0.770000
C	-2.703000	-2.318000	0.278000
C	-3.890000	-1.741000	-0.224000
C	-5.099000	-2.089000	0.378000
H	-5.995000	-1.584000	0.033000
C	-5.205000	-3.002000	1.416000
C	-4.022000	-3.590000	1.856000
H	-4.075000	-4.310000	2.660000
C	-2.768000	-3.270000	1.331000
C	-4.044000	-0.703000	-1.375000
C	-2.787000	-0.250000	-2.150000
H	-3.114000	0.252000	-3.065000
H	-2.124000	-1.066000	-2.430000
H	-2.212000	0.518000	-1.633000
C	-4.915000	-1.391000	-2.453000
H	-5.879000	-1.733000	-2.073000
H	-4.381000	-2.266000	-2.835000
H	-5.100000	-0.721000	-3.298000
C	-6.575000	-3.286000	2.043000
C	-7.134000	-1.978000	2.636000
H	-7.251000	-1.205000	1.870000
H	-6.462000	-1.586000	3.405000
H	-8.115000	-2.153000	3.092000
C	-6.494000	-4.330000	3.164000
H	-7.491000	-4.501000	3.582000
H	-5.845000	-3.998000	3.981000
H	-6.119000	-5.291000	2.795000
C	-7.540000	-3.810000	0.963000
H	-8.524000	-4.016000	1.399000
H	-7.161000	-4.737000	0.520000
H	-7.678000	-3.082000	0.158000
C	-1.518000	-3.966000	1.912000
C	-0.967000	-4.967000	0.879000
H	-1.707000	-5.750000	0.681000
H	-0.058000	-5.448000	1.260000
H	-0.730000	-4.484000	-0.068000
C	-0.429000	-2.948000	2.294000
H	0.400000	-3.452000	2.806000
H	-0.826000	-2.187000	2.976000
H	-0.038000	-2.451000	1.410000
C	-1.834000	-4.764000	3.190000
H	-2.530000	-5.589000	3.007000
H	-2.251000	-4.127000	3.977000
H	-0.908000	-5.204000	3.572000
C	-4.088000	1.205000	0.330000
C	-4.709000	0.548000	-0.770000
C	-5.929000	1.016000	-1.243000
H	-6.393000	0.507000	-2.082000
C	-6.591000	2.103000	-0.672000
C	-5.992000	2.696000	0.436000
H	-6.500000	3.523000	0.913000
C	-4.770000	2.273000	0.968000
C	-7.935000	2.566000	-1.250000
C	-7.763000	2.920000	-2.740000
H	-7.428000	2.057000	-3.324000
H	-8.713000	3.262000	-3.166000
H	-7.024000	3.717000	-2.864000
C	-8.485000	3.805000	-0.529000
H	-8.678000	3.605000	0.530000
H	-7.793000	4.651000	-0.597000
H	-9.431000	4.108000	-0.988000
C	-8.969000	1.433000	-1.112000
H	-9.121000	1.177000	-0.058000
H	-9.934000	1.737000	-1.534000
H	-8.645000	0.527000	-1.634000
C	-4.206000	2.917000	2.249000
C	-2.846000	3.588000	1.983000
H	-2.952000	4.396000	1.251000
H	-2.121000	2.872000	1.597000
H	-2.451000	4.023000	2.909000
C	-5.139000	3.998000	2.818000
H	-4.699000	4.408000	3.733000
H	-6.124000	3.595000	3.076000
H	-5.279000	4.829000	2.118000
C	-4.048000	1.834000	3.336000
H	-3.363000	1.053000	3.006000
H	-5.016000	1.372000	3.558000
H	-3.661000	2.279000	4.261000
Mg	1.612000	-0.196000	-1.534000
O	0.313000	0.845000	-0.450000
O	3.334000	0.304000	-1.056000
O	1.699000	-2.406000	-1.297000
C	0.961000	2.046000	-0.220000
C	2.021000	2.096000	0.712000
C	2.744000	3.284000	0.816000
H	3.596000	3.295000	1.488000
C	2.465000	4.422000	0.075000
C	1.413000	4.338000	-0.829000
H	1.182000	5.206000	-1.426000
C	0.661000	3.176000	-1.026000
C	2.561000	0.964000	1.630000
C	1.770000	-0.354000	1.703000
H	2.273000	-1.015000	2.417000
H	0.767000	-0.181000	2.105000
H	1.696000	-0.909000	0.769000
C	2.479000	1.504000	3.081000
H	3.114000	2.374000	3.254000
H	1.447000	1.798000	3.293000
H	2.760000	0.734000	3.805000
C	3.321000	5.680000	0.258000
C	4.789000	5.348000	-0.072000
H	5.185000	4.567000	0.583000
H	4.885000	4.998000	-1.105000
H	5.417000	6.238000	0.050000
C	2.868000	6.829000	-0.652000
H	3.499000	7.707000	-0.476000
H	2.954000	6.566000	-1.711000
H	1.832000	7.120000	-0.452000
C	3.220000	6.154000	1.720000
H	3.826000	7.055000	1.873000
H	2.183000	6.391000	1.978000
H	3.574000	5.390000	2.418000
C	-0.438000	3.186000	-2.114000
C	-1.825000	3.163000	-1.450000
H	-2.005000	4.110000	-0.933000
H	-2.615000	3.029000	-2.198000
H	-1.933000	2.376000	-0.705000
C	-0.270000	2.018000	-3.107000
H	-1.002000	2.107000	-3.916000
H	0.728000	2.045000	-3.561000
H	-0.426000	1.044000	-2.647000
C	-0.392000	4.461000	-2.981000
H	-0.584000	5.368000	-2.401000
H	0.567000	4.573000	-3.497000
H	-1.176000	4.395000	-3.741000
C	4.300000	0.357000	-0.163000
C	4.008000	0.647000	1.200000
C	5.041000	0.630000	2.131000
H	4.813000	0.832000	3.172000
C	6.369000	0.375000	1.782000
C	6.636000	0.159000	0.431000
H	7.660000	-0.019000	0.131000
C	5.648000	0.146000	-0.553000
C	7.454000	0.368000	2.866000
C	7.127000	-0.707000	3.920000
H	6.157000	-0.527000	4.393000
H	7.888000	-0.717000	4.708000
H	7.097000	-1.702000	3.463000
C	8.845000	0.063000	2.293000
H	9.156000	0.815000	1.561000

H	8.876000	-0.919000	1.809000
H	9.585000	0.059000	3.100000
C	7.510000	1.747000	3.550000
H	7.743000	2.530000	2.821000
H	8.283000	1.761000	4.327000
H	6.556000	2.001000	4.022000
C	6.007000	-0.076000	-2.033000
C	5.328000	-1.344000	-2.590000
H	5.711000	-2.238000	-2.090000
H	4.249000	-1.316000	-2.446000
H	5.537000	-1.439000	-3.663000
C	7.519000	-0.258000	-2.244000
H	7.718000	-0.400000	-3.312000
H	8.089000	0.617000	-1.915000
H	7.901000	-1.139000	-1.717000
C	5.575000	1.158000	-2.851000
H	4.503000	1.330000	-2.754000
H	6.101000	2.052000	-2.500000
H	5.817000	1.016000	-3.912000
C	2.061000	-3.555000	-0.484000
H	1.643000	-3.337000	0.499000
H	1.544000	-4.432000	-0.874000
C	3.548000	-3.754000	-0.403000
C	4.184000	-4.735000	-1.167000
C	4.305000	-2.977000	0.475000
C	5.556000	-4.937000	-1.052000
H	3.604000	-5.356000	-1.843000
C	5.677000	-3.174000	0.589000
H	3.828000	-2.206000	1.070000
C	6.304000	-4.157000	-0.172000
H	6.040000	-5.707000	-1.644000
H	6.251000	-2.545000	1.260000
H	7.373000	-4.315000	-0.080000
C	1.122000	-2.510000	-2.546000
O	0.853000	-1.363000	-3.002000
C	1.556000	-3.616000	-3.441000
C	1.779000	-3.385000	-4.730000
H	1.672000	-4.603000	-3.011000
H	2.106000	-4.179000	-5.392000
H	1.644000	-2.394000	-5.151000
H	-0.887000	-2.660000	-1.227000
O	-0.464000	-3.274000	-1.970000
C	-1.421000	-3.555000	-2.982000
H	-1.155000	-4.494000	-3.469000
H	-2.404000	-3.655000	-2.514000
H	-1.448000	-2.754000	-3.728000

[Mg(PBTP)]2-1a-MA-Int4

Mg	-1.126000	-0.337000	-0.498000
O	-1.422000	-2.231000	-0.674000
O	-2.458000	0.609000	0.405000
C	-2.541000	-2.621000	-0.016000
C	-3.775000	-1.962000	-0.228000
C	-4.835000	-2.248000	0.634000
H	-5.745000	-1.667000	0.517000
C	-4.779000	-3.204000	1.637000
C	-3.589000	-3.919000	1.748000
H	-3.520000	-4.694000	2.499000
C	-2.469000	-3.657000	0.959000
C	-4.138000	-0.929000	-1.338000
C	-3.078000	-0.605000	-2.420000
H	-3.590000	-0.153000	-3.274000
H	-2.547000	-1.490000	-2.771000
H	-2.360000	0.172000	-2.143000
C	-5.272000	-1.596000	-2.158000
H	-6.133000	-1.878000	-1.549000
H	-4.881000	-2.508000	-2.620000
H	-5.622000	-0.938000	-2.960000
C	-5.986000	-3.414000	2.558000
C	-6.290000	-2.098000	3.300000
H	-6.516000	-1.285000	2.603000
H	-5.432000	-1.789000	3.905000
H	-7.153000	-2.223000	3.964000
C	-5.736000	-4.510000	3.603000
H	-6.619000	-4.621000	4.240000
H	-4.888000	-4.263000	4.250000
H	-5.539000	-5.479000	3.134000
C	-7.214000	-3.820000	1.720000
H	-8.086000	-3.971000	2.365000
H	-7.023000	-4.752000	1.178000
H	-7.471000	-3.051000	0.985000
C	-1.171000	-4.451000	1.199000
C	-0.700000	-5.116000	-0.109000
H	-1.449000	-5.837000	-0.453000
H	0.238000	-5.659000	0.058000
H	-0.541000	-4.387000	-0.901000
C	-0.095000	-3.505000	1.763000
H	0.865000	-4.023000	1.878000
H	-0.397000	-3.129000	2.746000
H	0.040000	-2.648000	1.106000
C	-1.347000	-5.586000	2.223000
H	-2.110000	-6.306000	1.909000
H	-1.611000	-5.212000	3.217000
H	-0.400000	-6.127000	2.319000
C	-3.696000	1.042000	0.242000
C	-4.588000	0.372000	-0.645000
C	-5.876000	0.868000	-0.809000
H	-6.553000	0.350000	-1.480000
C	-6.347000	1.991000	-0.127000
C	-5.464000	2.613000	0.753000
H	-5.810000	3.475000	1.306000
C	-4.155000	2.172000	0.965000
C	-7.792000	2.459000	-0.339000
C	-8.017000	2.794000	-1.826000
H	-7.835000	1.927000	-2.467000
H	-9.049000	3.124000	-1.994000
H	-7.345000	3.596000	-2.147000
C	-8.128000	3.709000	0.486000
H	-8.028000	3.524000	1.560000
H	-7.482000	4.553000	0.222000
H	-9.163000	4.010000	0.296000
C	-8.761000	1.336000	0.081000
H	-8.619000	1.080000	1.136000
H	-9.801000	1.650000	-0.060000
H	-8.602000	0.427000	-0.508000
C	-3.239000	2.876000	1.985000
C	-2.000000	3.467000	1.287000
H	-2.292000	4.234000	0.562000
H	-1.437000	2.697000	0.760000
H	-1.336000	3.938000	2.023000
C	-3.950000	4.032000	2.707000
H	-3.258000	4.486000	3.424000
H	-4.828000	3.690000	3.265000
H	-4.266000	4.817000	2.013000
C	-2.791000	1.871000	3.067000
H	-2.239000	1.043000	2.622000
H	-3.660000	1.463000	3.595000
H	-2.150000	2.371000	3.803000
Mg	1.924000	0.073000	-2.084000
O	0.270000	0.785000	-1.272000
O	3.381000	0.532000	-1.037000
O	2.217000	-2.227000	-1.891000
C	0.665000	2.052000	-0.881000
C	1.490000	2.203000	0.253000
C	2.054000	3.457000	0.484000
H	2.747000	3.550000	1.314000
C	1.797000	4.568000	-0.307000
C	0.916000	4.396000	-1.371000
H	0.680000	5.251000	-1.985000
C	0.340000	3.165000	-1.695000
C	1.849000	1.139000	1.320000
C	1.137000	-0.225000	1.239000
H	1.474000	-0.842000	2.077000
H	0.057000	-0.098000	1.403000
H	1.347000	-0.793000	0.331000
C	1.346000	1.716000	2.670000
H	1.884000	2.615000	2.976000
H	0.288000	1.975000	2.572000
H	1.435000	0.980000	3.474000
C	2.467000	5.907000	0.021000
C	3.996000	5.739000	-0.049000
H	4.355000	4.985000	0.658000
H	4.306000	5.431000	-1.053000
H	4.494000	6.686000	0.189000
C	2.058000	7.018000	-0.955000
H	2.564000	7.950000	-0.683000
H	2.340000	6.776000	-1.985000
H	0.980000	7.206000	-0.928000
C	2.062000	6.342000	1.443000
H	2.527000	7.302000	1.695000
H	0.976000	6.457000	1.518000
H	2.376000	5.611000	2.193000
C	-0.664000	3.090000	-2.868000
C	-2.076000	2.888000	-2.285000
H	-2.411000	3.796000	-1.776000
H	-2.795000	2.658000	-3.079000
H	-2.106000	2.090000	-1.544000
C	-0.333000	1.967000	-3.872000
H	-0.961000	2.072000	-4.763000
H	0.712000	2.033000	-4.203000
H	-0.520000	0.976000	-3.460000
C	-0.695000	4.392000	-3.690000
H	-1.011000	5.254000	-3.095000
H	0.278000	4.617000	-4.139000
H	-1.421000	4.280000	-4.501000
C	4.046000	0.593000	0.098000
C	3.368000	0.880000	1.316000
C	4.086000	0.876000	2.508000
H	3.561000	1.082000	3.434000
C	5.457000	0.624000	2.564000
C	6.107000	0.399000	1.352000
H	7.173000	0.219000	1.365000
C	5.450000	0.382000	0.121000
C	6.180000	0.627000	3.917000
C	5.540000	-0.418000	4.850000
H	4.478000	-0.214000	5.019000
H	6.039000	-0.418000	5.826000
H	5.625000	-1.424000	4.425000
C	7.671000	0.289000	3.780000
H	8.199000	1.023000	3.163000
H	7.821000	-0.702000	3.338000
H	8.141000	0.288000	4.769000
C	6.060000	2.022000	4.561000
H	6.511000	2.784000	3.917000
H	6.571000	2.045000	5.530000
H	5.014000	2.299000	4.727000
C	6.232000	0.147000	-1.185000
C	5.744000	-1.129000	-1.898000
H	5.931000	-2.013000	-1.282000
H	4.675000	-1.076000	-2.100000
H	6.276000	-1.254000	-2.849000
C	7.739000	-0.032000	-0.939000
H	8.243000	-0.181000	-1.899000
H	8.186000	0.847000	-0.463000
H	7.949000	-0.909000	-0.317000
C	6.060000	1.368000	-2.111000
H	5.007000	1.535000	-2.342000
H	6.455000	2.270000	-1.632000
H	6.608000	1.214000	-3.049000
C	2.171000	-3.395000	-1.014000
H	1.405000	-3.128000	-0.290000
H	1.805000	-4.248000	-1.583000
C	3.505000	-3.620000	-0.370000
C	4.374000	-4.606000	-0.841000
C	3.897000	-2.817000	0.702000
C	5.622000	-4.781000	-0.251000
H	4.075000	-5.248000	-1.665000
C	5.145000	-2.985000	1.289000
H	3.236000	-2.040000	1.071000
C	6.009000	-3.968000	0.813000
H	6.291000	-5.550000	-0.620000
H	5.444000	-2.332000	2.102000
H	6.985000	-4.101000	1.269000
C	2.311000	-2.256000	-3.215000
O	2.237000	-1.137000	-3.740000
C	2.556000	-3.489000	-3.962000
C	2.651000	-3.443000	-5.291000
H	2.660000	-4.421000	-3.423000
H	2.837000	-4.343000	-5.866000
H	2.544000	-2.507000	-5.829000

H	-0.776000	-2.759000	-2.118000
O	-0.281000	-2.920000	-2.956000
C	-1.031000	-3.797000	-3.766000
H	-0.445000	-4.007000	-4.665000
H	-1.235000	-4.749000	-3.258000
H	-1.990000	-3.360000	-4.075000

[Mg(PBTP)]2-2a-MeOH

Mg	1.374000	0.655000	-1.229000
O	1.056000	-1.158000	-0.471000
O	3.189000	1.062000	-0.951000
C	2.190000	-1.917000	-0.557000
C	3.332000	-1.579000	0.223000
C	4.548000	-2.195000	-0.059000
H	5.420000	-1.848000	0.487000
C	4.712000	-3.191000	-1.010000
C	3.566000	-3.573000	-1.685000
H	3.639000	-4.370000	-2.414000
C	2.313000	-2.966000	-1.511000
C	3.445000	-0.569000	1.396000
C	2.163000	0.107000	1.889000
H	2.411000	0.722000	2.759000
H	1.432000	-0.637000	2.218000
H	1.717000	0.781000	1.168000
C	3.909000	-1.393000	2.625000
H	4.881000	-1.872000	2.490000
H	3.174000	-2.183000	2.813000
H	3.961000	-0.767000	3.522000
C	6.097000	-3.792000	-1.265000
C	7.046000	-2.677000	-1.748000
H	7.136000	-1.877000	-1.008000
H	6.676000	-2.232000	-2.678000
H	8.047000	-3.082000	-1.934000
C	6.062000	-4.895000	-2.330000
H	7.069000	-5.298000	-2.480000
H	5.710000	-4.513000	-3.294000
H	5.414000	-5.725000	-2.031000
C	6.646000	-4.396000	0.041000
H	7.637000	-4.833000	-0.125000
H	5.982000	-5.184000	0.412000
H	6.742000	-3.639000	0.826000
C	1.211000	-3.577000	-2.411000
C	1.026000	-5.053000	-1.997000
H	1.922000	-5.649000	-2.188000
H	0.199000	-5.508000	-2.554000
H	0.809000	-5.123000	-0.926000
C	-0.152000	-2.894000	-2.345000
H	-0.865000	-3.448000	-2.966000
H	-0.089000	-1.881000	-2.739000
H	-0.541000	-2.848000	-1.330000
C	1.640000	-3.516000	-3.894000
H	2.557000	-4.072000	-4.098000
H	1.801000	-2.478000	-4.202000
H	0.851000	-3.937000	-4.527000
C	4.259000	1.259000	-0.206000
C	4.456000	0.521000	0.996000
C	5.579000	0.778000	1.773000
H	5.713000	0.219000	2.693000
C	6.552000	1.713000	1.417000
C	6.363000	2.391000	0.216000
H	7.111000	3.107000	-0.097000
C	5.254000	2.189000	-0.609000
C	7.764000	1.936000	2.331000
C	7.282000	2.443000	3.704000
H	6.597000	1.731000	4.176000
H	8.130000	2.593000	4.381000
H	6.755000	3.397000	3.599000
C	8.746000	2.968000	1.759000
H	9.151000	2.648000	0.793000
H	8.274000	3.947000	1.627000
H	9.588000	3.095000	2.448000
C	8.523000	0.608000	2.519000
H	8.872000	0.223000	1.555000
H	9.395000	0.752000	3.167000
H	7.891000	-0.158000	2.977000
C	5.120000	2.947000	-1.942000
C	3.863000	3.837000	-1.918000
H	3.956000	4.612000	-1.150000
H	2.977000	3.248000	-1.683000
H	3.723000	4.335000	-2.886000
C	6.317000	3.868000	-2.228000
H	6.167000	4.363000	-3.193000
H	7.258000	3.312000	-2.284000
H	6.423000	4.650000	-1.469000
C	5.040000	1.940000	-3.108000
H	4.252000	1.205000	-2.939000
H	5.984000	1.393000	-3.204000
H	4.859000	2.462000	-4.056000
Mg	-0.601000	-0.614000	0.579000
O	-0.238000	1.219000	-0.231000
O	-2.493000	-0.414000	0.693000
O	-0.962000	-3.673000	0.772000
C	-1.173000	2.213000	-0.242000
C	-2.360000	2.083000	-1.005000
C	-3.319000	3.095000	-0.913000
H	-4.267000	2.934000	-1.411000
C	-3.163000	4.253000	-0.175000
C	-1.974000	4.374000	0.533000
H	-1.822000	5.262000	1.126000
C	-0.989000	3.385000	0.552000
C	-2.850000	0.877000	-1.863000
C	-1.828000	-0.188000	-2.262000
H	-2.288000	-0.859000	-2.995000
H	-0.949000	0.253000	-2.743000
H	-1.523000	-0.827000	-1.445000
C	-3.324000	1.418000	-3.237000
H	-4.089000	2.193000	-3.184000
H	-2.466000	1.843000	-3.767000
H	-3.720000	0.603000	-3.851000
C	-4.298000	5.281000	-0.120000
C	-5.533000	4.625000	0.527000
H	-5.856000	3.738000	-0.027000
H	-5.311000	4.312000	1.552000
H	-6.371000	5.332000	0.557000
C	-3.918000	6.520000	0.702000
H	-4.747000	7.236000	0.696000
H	-3.708000	6.267000	1.746000
H	-3.039000	7.024000	0.287000
C	-4.653000	5.741000	-1.547000
H	-5.460000	6.481000	-1.520000
H	-3.785000	6.198000	-2.034000
H	-4.988000	4.905000	-2.169000
C	0.184000	3.569000	1.540000
C	1.550000	3.513000	0.837000
H	1.632000	4.337000	0.120000
H	2.363000	3.618000	1.564000
H	1.732000	2.590000	0.297000
C	0.041000	2.514000	2.653000
H	0.867000	2.584000	3.369000
H	-0.898000	2.672000	3.192000
H	0.028000	1.501000	2.257000
C	0.157000	4.935000	2.255000
H	0.227000	5.770000	1.551000
H	-0.739000	5.070000	2.868000
H	1.021000	4.995000	2.925000
C	-3.726000	-0.183000	0.296000
C	-3.995000	0.269000	-1.031000
C	-5.310000	0.346000	-1.473000
H	-5.498000	0.656000	-2.496000
C	-6.404000	0.090000	-0.643000
C	-6.121000	-0.196000	0.690000
H	-6.948000	-0.332000	1.374000
C	-4.823000	-0.323000	1.190000
C	-7.827000	0.176000	-1.205000
C	-7.997000	-0.874000	-2.320000
H	-7.270000	-0.725000	-3.124000
H	-9.001000	-0.818000	-2.758000
H	-7.853000	-1.884000	-1.921000
C	-8.894000	-0.088000	-0.134000
H	-8.847000	0.651000	0.672000
H	-8.784000	-1.085000	0.307000
H	-9.891000	-0.029000	-0.583000
C	-8.075000	1.581000	-1.787000
H	-7.974000	2.344000	-1.008000
H	-9.085000	1.654000	-2.207000
H	-7.365000	1.821000	-2.584000
C	-4.584000	-0.574000	2.688000
C	-3.904000	-1.936000	2.896000
H	-4.550000	-2.749000	2.552000
H	-2.977000	-1.970000	2.330000
H	-3.681000	-2.097000	3.958000
C	-5.888000	-0.582000	3.502000
H	-5.650000	-0.740000	4.559000
H	-6.429000	0.367000	3.420000
H	-6.559000	-1.391000	3.192000
C	-3.697000	0.551000	3.260000
H	-2.739000	0.596000	2.740000
H	-4.191000	1.522000	3.148000
H	-3.513000	0.384000	4.328000
C	-1.731000	-4.857000	0.439000
H	-1.371000	-5.100000	-0.561000
H	-1.472000	-5.685000	1.101000
H	2.206000	1.875000	-3.297000
C	-3.202000	-4.557000	0.429000
C	-4.089000	-5.303000	1.203000
C	-3.688000	-3.530000	-0.383000
C	-5.456000	-5.029000	1.157000
H	-3.716000	-6.099000	1.841000
C	-5.046000	-3.244000	-0.415000
H	-2.999000	-2.935000	-0.976000
C	-5.932000	-3.999000	0.352000
H	-6.142000	-5.615000	1.760000
H	-5.409000	-2.421000	-1.021000
H	-6.994000	-3.773000	0.328000
C	-0.801000	-3.208000	1.998000
O	-0.422000	-2.038000	2.103000
O	1.294000	1.695000	-3.031000
C	0.499000	2.876000	-3.235000
H	-0.476000	2.685000	-2.788000
H	0.956000	3.734000	-2.736000
H	0.389000	3.069000	-4.303000
C	-1.036000	-4.063000	3.172000
C	-1.047000	-3.523000	4.390000
H	-1.208000	-5.121000	3.028000
H	-1.220000	-4.133000	5.269000
H	-0.894000	-2.458000	4.532000

Na2(PBTP)•2(MMA)•2(1a)

Na	2.207000	0.135000	-1.060000
O	1.279000	-1.453000	0.190000
C	0.089000	-1.759000	0.623000
C	-0.632000	-0.865000	1.476000
C	-1.926000	-1.184000	1.880000
H	-2.463000	-0.469000	2.487000
C	-2.581000	-2.354000	1.493000
C	-1.862000	-3.233000	0.678000
H	-2.343000	-4.156000	0.373000
C	-0.566000	-2.974000	0.225000
C	0.057000	0.388000	2.037000
C	1.069000	-0.144000	3.076000
H	0.545000	-0.812000	3.766000
H	1.509000	0.659000	3.676000
H	1.873000	-0.706000	2.598000
C	-4.019000	-2.682000	1.915000
C	-4.018000	-3.917000	2.835000
H	-3.432000	-3.725000	3.739000
H	-3.585000	-4.787000	2.332000
H	-5.040000	-4.175000	3.137000
C	-4.677000	-1.510000	2.658000
H	-5.720000	-1.755000	2.886000
H	-4.660000	-0.603000	2.045000
H	-4.173000	-1.294000	3.606000
C	-4.874000	-2.979000	0.670000
H	-5.906000	-3.211000	0.956000
H	-4.489000	-3.832000	0.102000
H	-4.897000	-2.114000	0.001000
C	0.144000	-3.965000	-0.714000
C	1.453000	-4.463000	-0.071000
H	1.243000	-5.008000	0.855000

H	2.106000	-3.623000	0.166000
H	1.981000	-5.141000	-0.752000
C	-0.711000	-5.204000	-1.022000
H	-0.148000	-5.873000	-1.681000
H	-1.643000	-4.943000	-1.535000
H	-0.962000	-5.767000	-0.116000
C	0.450000	-3.275000	-2.056000
H	0.923000	-3.980000	-2.750000
H	1.117000	-2.425000	-1.916000
H	-0.477000	-2.922000	-2.525000
O	-1.158000	1.200000	-0.502000
C	0.035000	1.658000	-0.221000
C	0.728000	1.272000	0.971000
C	2.005000	1.773000	1.218000
H	2.512000	1.443000	2.114000
C	2.680000	2.639000	0.356000
C	2.012000	2.987000	-0.819000
H	2.512000	3.639000	-1.523000
C	0.727000	2.528000	-1.134000
C	-0.917000	1.316000	2.791000
H	-0.370000	2.197000	3.139000
H	-1.348000	0.829000	3.672000
H	-1.732000	1.659000	2.151000
C	4.112000	3.106000	0.652000
C	4.365000	4.520000	0.101000
H	3.647000	5.237000	0.513000
H	4.294000	4.559000	-0.990000
H	5.373000	4.852000	0.370000
C	4.390000	3.141000	2.164000
H	5.388000	3.553000	2.348000
H	4.363000	2.145000	2.615000
H	3.660000	3.768000	2.686000
C	5.102000	2.132000	-0.016000
H	6.138000	2.371000	0.254000
H	5.012000	2.190000	-1.104000
H	4.895000	1.098000	0.279000
C	0.060000	2.957000	-2.451000
C	-1.243000	3.718000	-2.137000
H	-1.015000	4.655000	-1.617000
H	-1.890000	3.117000	-1.500000
H	-1.777000	3.968000	-3.062000
C	0.938000	3.896000	-3.292000
H	0.402000	4.160000	-4.210000
H	1.882000	3.426000	-3.587000
H	1.169000	4.828000	-2.765000
C	-0.223000	1.712000	-3.315000
H	-0.762000	1.988000	-4.229000
H	-0.813000	0.984000	-2.760000
H	0.718000	1.236000	-3.616000
Na	-2.201000	-0.791000	-0.886000
C	3.930000	-1.400000	4.819000
C	4.652000	-0.448000	4.102000
C	4.954000	-0.671000	2.762000
C	4.538000	-1.840000	2.122000
C	3.817000	-2.788000	2.850000
C	3.515000	-2.571000	4.192000
C	4.792000	-2.042000	0.647000
O	3.796000	-1.432000	-0.147000
H	3.694000	-1.228000	5.865000
H	4.983000	0.465000	4.586000
H	5.525000	0.068000	2.206000
H	3.483000	-3.699000	2.360000
H	2.951000	-3.315000	4.746000
H	4.852000	-3.118000	0.425000
H	5.747000	-1.591000	0.360000
H	2.894000	-1.669000	0.192000
C	-3.123000	4.211000	3.759000
C	-4.035000	3.164000	3.873000
C	-4.501000	2.520000	2.732000
C	-4.063000	2.913000	1.465000
C	-3.156000	3.968000	1.360000
C	-2.686000	4.615000	2.500000
C	-4.459000	2.121000	0.241000
O	-3.641000	0.978000	0.097000
H	-2.753000	4.710000	4.649000
H	-4.381000	2.847000	4.852000
H	-5.203000	1.695000	2.820000
H	-2.800000	4.273000	0.380000
H	-1.973000	5.428000	2.406000
H	-4.402000	2.761000	-0.653000
H	-5.490000	1.765000	0.329000
H	-2.690000	1.264000	0.075000
C	-4.909000	-0.432000	-2.628000
O	-3.871000	-1.071000	-2.525000
O	-4.938000	0.858000	-2.936000
C	-3.672000	1.490000	-3.156000
H	-3.899000	2.527000	-3.394000
H	-3.061000	1.435000	-2.255000
H	-3.152000	1.014000	-3.989000
C	-6.278000	-0.999000	-2.456000
C	-6.386000	-2.324000	-2.351000
H	-7.353000	-2.801000	-2.232000
H	-5.507000	-2.958000	-2.377000
C	-7.446000	-0.053000	-2.435000
H	-8.375000	-0.598000	-2.261000
H	-7.329000	0.698000	-1.648000
H	-7.530000	0.487000	-3.383000
C	4.019000	-0.960000	-3.114000
O	2.796000	-0.947000	-3.111000
O	4.736000	-2.070000	-3.038000
C	3.999000	-3.282000	-2.825000
H	4.745000	-4.074000	-2.797000
H	3.464000	-3.211000	-1.876000
H	3.290000	-3.446000	-3.638000
C	4.861000	0.270000	-3.200000
C	4.256000	1.392000	-3.600000
H	4.799000	2.328000	-3.679000
H	3.203000	1.393000	-3.860000
C	6.309000	0.178000	-2.816000
H	6.795000	1.150000	-2.917000
H	6.408000	-0.155000	-1.778000
H	6.838000	-0.547000	-3.441000

Na2(PBTP)•2(MMA)•2(1a)-TS4

Na	2.209000	0.329000	-1.004000
O	1.411000	-1.160000	0.684000
C	0.166000	-1.582000	1.047000
C	-0.623000	-0.617000	1.702000
C	-1.922000	-0.968000	2.068000
H	-2.541000	-0.215000	2.530000
C	-2.463000	-2.226000	1.816000
C	-1.636000	-3.159000	1.190000
H	-2.034000	-4.148000	1.002000
C	-0.323000	-2.883000	0.793000
C	-0.044000	0.743000	2.127000
C	0.940000	0.416000	3.275000
H	0.421000	-0.195000	4.020000
H	1.282000	1.323000	3.783000
H	1.810000	-0.138000	2.917000
C	-3.900000	-2.600000	2.199000
C	-3.871000	-3.725000	3.249000
H	-3.338000	-3.402000	4.149000
H	-3.374000	-4.621000	2.865000
H	-4.891000	-4.003000	3.536000
C	-4.666000	-1.401000	2.773000
H	-5.698000	-1.693000	2.990000
H	-4.684000	-0.577000	2.052000
H	-4.221000	-1.037000	3.705000
C	-4.657000	-3.080000	0.948000
H	-5.687000	-3.351000	1.206000
H	-4.188000	-3.958000	0.492000
H	-4.696000	-2.289000	0.194000
C	0.519000	-3.964000	0.091000
C	1.803000	-4.255000	0.898000
H	1.548000	-4.733000	1.849000
H	2.391000	-3.369000	1.141000
H	2.449000	-4.938000	0.336000
C	-0.234000	-5.301000	-0.018000
H	0.427000	-6.039000	-0.483000
H	-1.128000	-5.220000	-0.646000
H	-0.527000	-5.692000	0.961000
C	0.841000	-3.524000	-1.352000
H	1.306000	-4.356000	-1.892000
H	1.525000	-2.677000	-1.442000
H	-0.085000	-3.268000	-1.878000
O	-1.192000	1.100000	-0.513000
C	-0.065000	1.715000	-0.259000
C	0.613000	1.537000	0.987000
C	1.827000	2.186000	1.219000
H	2.320000	2.023000	2.168000
C	2.435000	3.025000	0.287000
C	1.775000	3.181000	-0.935000
H	2.229000	3.824000	-1.680000
C	0.568000	2.551000	-1.244000
C	-1.122000	1.678000	2.713000
H	-0.651000	2.628000	2.977000
H	-1.572000	1.269000	3.624000
H	-1.915000	1.886000	1.993000
C	3.719000	3.813000	0.579000
C	3.379000	5.316000	0.619000
H	2.642000	5.524000	1.402000
H	2.961000	5.655000	-0.334000
H	4.277000	5.909000	0.826000
C	4.349000	3.425000	1.923000
H	5.257000	4.013000	2.087000
H	4.633000	2.369000	1.939000
H	3.674000	3.617000	2.763000
C	4.767000	3.562000	-0.519000
H	5.688000	4.114000	-0.303000
H	4.409000	3.884000	-1.501000
H	5.010000	2.497000	-0.586000
C	-0.078000	2.757000	-2.624000
C	-1.456000	3.422000	-2.443000
H	-1.337000	4.424000	-2.015000
H	-2.076000	2.831000	-1.770000
H	-1.966000	3.524000	-3.408000
C	0.755000	3.667000	-3.540000
H	0.245000	3.770000	-4.504000
H	1.748000	3.250000	-3.736000
H	0.877000	4.672000	-3.123000
C	-0.215000	1.401000	-3.343000
H	-0.710000	1.529000	-4.313000
H	-0.797000	0.705000	-2.741000
H	0.769000	0.956000	-3.527000
Na	-2.066000	-0.956000	-0.828000
C	5.053000	-1.235000	4.024000
C	4.611000	-0.276000	3.117000
C	4.467000	-0.596000	1.769000
C	4.760000	-1.880000	1.308000
C	5.196000	-2.837000	2.228000
C	5.348000	-2.520000	3.574000
C	4.617000	-2.247000	-0.156000
O	3.662000	-1.465000	-0.800000
H	5.160000	-0.986000	5.075000
H	4.365000	0.724000	3.463000
H	4.104000	0.150000	1.069000
H	5.409000	-3.847000	1.884000
H	5.685000	-3.278000	4.274000
H	4.363000	-3.320000	-0.215000
H	5.598000	-2.158000	-0.644000
H	1.970000	-1.792000	0.202000
C	-3.579000	4.473000	3.166000
C	-4.358000	3.351000	3.448000
C	-4.741000	2.495000	2.420000
C	-4.353000	2.748000	1.103000
C	-3.582000	3.879000	0.830000
C	-3.195000	4.738000	1.855000
C	-4.651000	1.753000	0.007000
O	-3.682000	0.722000	-0.019000
H	-3.276000	5.140000	3.967000
H	-4.663000	3.144000	4.468000
H	-5.341000	1.617000	2.642000
H	-3.271000	4.081000	-0.191000
H	-2.591000	5.611000	1.629000
H	-4.697000	2.269000	-0.964000
H	-5.620000	1.272000	0.172000
H	-2.775000	1.122000	-0.036000
C	-4.773000	-1.072000	-2.587000
O	-3.697000	-1.613000	-2.369000
O	-4.880000	0.162000	-3.062000

C	-3.654000	0.856000	-3.318000
H	-3.941000	1.806000	-3.764000
H	-3.123000	1.026000	-2.380000
H	-3.028000	0.284000	-4.004000
C	-6.105000	-1.711000	-2.383000
C	-6.127000	-3.025000	-2.149000
H	-7.064000	-3.552000	-2.001000
H	-5.208000	-3.597000	-2.101000
C	-7.332000	-0.846000	-2.463000
H	-8.228000	-1.438000	-2.268000
H	-7.285000	-0.035000	-1.730000
H	-7.425000	-0.383000	-3.449000
C	3.814000	-1.151000	-2.523000
O	2.613000	-1.077000	-2.899000
O	4.626000	-2.183000	-2.965000
C	3.958000	-3.408000	-3.203000
H	4.703000	-4.091000	-3.612000
H	3.557000	-3.821000	-2.271000
H	3.136000	-3.281000	-3.912000
C	4.660000	0.127000	-2.519000
C	4.139000	1.208000	-3.103000
H	4.682000	2.147000	-3.149000
H	3.156000	1.163000	-3.560000
C	6.025000	0.129000	-1.893000
H	6.556000	1.057000	-2.121000
H	5.950000	0.040000	-0.804000
H	6.621000	-0.714000	-2.253000

Na2(PBTP)•2(MMA)•2(1a)-Int5

Na	2.242000	0.401000	-0.957000
O	1.405000	-1.082000	0.691000
C	0.157000	-1.457000	1.100000
C	-0.594000	-0.456000	1.745000
C	-1.891000	-0.770000	2.152000
H	-2.484000	0.009000	2.603000
C	-2.464000	-2.023000	1.952000
C	-1.673000	-2.992000	1.332000
H	-2.094000	-3.977000	1.184000
C	-0.369000	-2.752000	0.887000
C	0.016000	0.912000	2.100000
C	1.062000	0.622000	3.202000
H	0.589000	0.027000	3.989000
H	1.422000	1.545000	3.666000
H	1.918000	0.064000	2.816000
C	-3.893000	-2.358000	2.397000
C	-3.830000	-3.378000	3.550000
H	-3.276000	-2.968000	4.400000
H	-3.334000	-4.302000	3.238000
H	-4.840000	-3.634000	3.889000
C	-4.648000	-1.109000	2.874000
H	-5.679000	-1.377000	3.125000
H	-4.666000	-0.345000	2.090000
H	-4.194000	-0.675000	3.771000
C	-4.685000	-2.964000	1.225000
H	-5.706000	-3.202000	1.543000
H	-4.234000	-3.890000	0.854000
H	-4.750000	-2.264000	0.387000
C	0.427000	-3.866000	0.182000
C	1.708000	-4.192000	0.980000
H	1.444000	-4.638000	1.944000
H	2.335000	-3.325000	1.198000
H	2.320000	-4.913000	0.427000
C	-0.372000	-5.177000	0.096000
H	0.250000	-5.937000	-0.386000
H	-1.280000	-5.065000	-0.507000
H	-0.652000	-5.557000	1.083000
C	0.744000	-3.456000	-1.272000
H	1.201000	-4.301000	-1.798000
H	1.430000	-2.614000	-1.392000
H	-0.184000	-3.206000	-1.798000
O	-1.247000	1.124000	-0.496000
C	-0.117000	1.758000	-0.333000
C	0.610000	1.664000	0.898000
C	1.818000	2.342000	1.040000
H	2.356000	2.250000	1.976000
C	2.386000	3.120000	0.026000
C	1.683000	3.184000	-1.177000
H	2.104000	3.761000	-1.989000
C	0.469000	2.523000	-1.395000
C	-1.028000	1.867000	2.713000
H	-0.542000	2.823000	2.927000
H	-1.432000	1.484000	3.655000
H	-1.855000	2.060000	2.027000
C	3.719000	3.841000	0.262000
C	3.572000	4.815000	1.447000
H	3.291000	4.293000	2.366000
H	2.804000	5.566000	1.237000
H	4.518000	5.336000	1.634000
C	4.815000	2.810000	0.587000
H	5.782000	3.305000	0.730000
H	4.917000	2.086000	-0.230000
H	4.588000	2.261000	1.506000
C	4.167000	4.645000	-0.965000
H	5.118000	5.144000	-0.751000
H	3.438000	5.417000	-1.232000
H	4.319000	4.000000	-1.836000
C	-0.218000	2.600000	-2.770000
C	-1.617000	3.225000	-2.620000
H	-1.536000	4.262000	-2.278000
H	-2.208000	2.664000	-1.894000
H	-2.142000	3.228000	-3.583000
C	0.565000	3.459000	-3.776000
H	0.024000	3.477000	-4.727000
H	1.562000	3.051000	-3.973000
H	0.674000	4.495000	-3.439000
C	-0.322000	1.187000	-3.374000
H	-0.797000	1.227000	-4.361000
H	-0.916000	0.541000	-2.728000
H	0.671000	0.738000	-3.492000
Na	-2.133000	-0.911000	-0.724000
C	5.046000	-1.334000	4.019000
C	4.726000	-0.367000	3.070000
C	4.581000	-0.721000	1.732000
C	4.757000	-2.043000	1.321000
C	5.074000	-3.007000	2.280000
C	5.221000	-2.657000	3.619000
C	4.613000	-2.430000	-0.133000
O	3.692000	-1.585000	-0.776000
H	5.152000	-1.061000	5.065000
H	4.578000	0.666000	3.373000
H	4.319000	0.030000	0.996000
H	5.194000	-4.044000	1.976000
H	5.462000	-3.420000	4.353000
H	4.279000	-3.478000	-0.192000
H	5.588000	-2.398000	-0.630000
H	1.922000	-1.767000	0.242000
C	-3.375000	4.699000	3.093000
C	-4.220000	3.633000	3.395000
C	-4.656000	2.782000	2.384000
C	-4.255000	2.986000	1.061000
C	-3.414000	4.060000	0.768000
C	-2.974000	4.913000	1.777000
C	-4.623000	1.991000	-0.014000
O	-3.740000	0.886000	-0.004000
H	-3.031000	5.361000	3.881000
H	-4.538000	3.464000	4.419000
H	-5.308000	1.946000	2.621000
H	-3.088000	4.220000	-0.256000
H	-2.315000	5.741000	1.536000
H	-4.618000	2.485000	-0.997000
H	-5.630000	1.595000	0.155000
H	-2.807000	1.224000	-0.037000
C	-4.802000	-1.377000	-2.548000
O	-3.683000	-1.753000	-2.227000
O	-5.021000	-0.201000	-3.115000
C	-3.858000	0.603000	-3.340000
H	-4.220000	1.510000	-3.820000
H	-3.382000	0.847000	-2.389000
H	-3.156000	0.084000	-3.995000
C	-6.052000	-2.170000	-2.375000
C	-5.925000	-3.435000	-1.971000
H	-6.794000	-4.070000	-1.832000
H	-4.948000	-3.860000	-1.769000
C	-7.366000	-1.506000	-2.677000
H	-8.192000	-2.197000	-2.495000
H	-7.510000	-0.619000	-2.054000
H	-7.410000	-1.177000	-3.719000
C	3.856000	-1.224000	-2.291000
O	2.668000	-1.001000	-2.720000
O	4.586000	-2.290000	-2.873000
C	3.810000	-3.431000	-3.175000
H	4.410000	-4.057000	-3.838000
H	3.568000	-4.003000	-2.268000
H	2.875000	-3.149000	-3.665000
C	4.824000	-0.015000	-2.308000
C	4.393000	1.129000	-2.845000
H	5.028000	2.010000	-2.882000
H	3.398000	1.194000	-3.276000
C	6.209000	-0.141000	-1.734000
H	6.805000	0.749000	-1.955000
H	6.179000	-0.259000	-0.645000
H	6.721000	-1.015000	-2.146000

Mg(PBTP)•MA•1a

O	-2.320000	-0.199000	-1.273000
O	0.083000	-0.752000	0.867000
C	1.289000	-0.227000	0.689000
C	2.249000	-0.304000	1.732000
C	1.642000	0.415000	-0.532000
C	3.548000	0.152000	1.484000
C	2.948000	0.857000	-0.702000
C	3.935000	0.723000	0.276000
H	4.282000	0.071000	2.274000
H	3.215000	1.335000	-1.638000
C	-1.955000	1.069000	-1.041000
C	-0.594000	1.454000	-1.060000
C	-2.940000	2.055000	-0.716000
C	-0.242000	2.717000	-0.568000
C	-2.517000	3.307000	-0.285000
C	-1.170000	3.660000	-0.155000
H	0.817000	2.946000	-0.506000
H	-3.265000	4.043000	-0.012000
C	0.591000	0.632000	-1.635000
C	-4.442000	1.731000	-0.803000
C	-4.785000	1.185000	-2.203000
C	-4.819000	0.708000	0.284000
C	-5.327000	2.970000	-0.582000
H	-4.162000	0.326000	-2.452000
H	-5.840000	0.887000	-2.247000
H	-4.622000	1.959000	-2.960000
H	-4.640000	1.129000	1.280000
H	-5.881000	0.441000	0.214000
H	-4.227000	-0.200000	0.185000
H	-6.376000	2.689000	-0.715000
H	-5.224000	3.377000	0.430000
H	-5.100000	3.766000	-1.299000
C	1.858000	-0.818000	3.134000
C	0.727000	0.066000	3.697000
C	1.391000	-2.286000	3.105000
C	3.031000	-0.747000	4.126000
H	1.067000	1.102000	3.797000
H	-0.140000	0.050000	3.035000
H	0.423000	-0.290000	4.689000
H	2.151000	-2.933000	2.651000
H	1.221000	-2.648000	4.125000
H	0.455000	-2.393000	2.557000
H	2.690000	-1.085000	5.110000
H	3.864000	-1.391000	3.826000
H	3.409000	0.274000	4.241000
C	-0.782000	5.031000	0.411000
C	-1.332000	5.166000	1.843000
C	0.740000	5.226000	0.464000
C	-1.378000	6.146000	-0.469000
H	-2.422000	5.076000	1.865000
H	-0.918000	4.385000	2.490000
H	-1.067000	6.141000	2.268000
H	1.190000	5.171000	-0.533000
H	0.971000	6.212000	0.881000
H	1.222000	4.474000	1.097000

H	-1.111000	7.132000	-0.074000
H	-0.999000	6.073000	-1.494000
H	-2.470000	6.087000	-0.511000
C	5.352000	1.246000	0.005000
C	6.295000	1.017000	1.193000
C	5.949000	0.530000	-1.222000
C	5.292000	2.760000	-0.275000
H	5.954000	1.547000	2.088000
H	6.386000	-0.046000	1.438000
H	7.295000	1.389000	0.948000
H	5.315000	0.644000	-2.106000
H	6.938000	0.938000	-1.462000
H	6.066000	-0.542000	-1.027000
H	6.296000	3.158000	-0.460000
H	4.677000	2.984000	-1.152000
H	4.863000	3.293000	0.580000
C	1.137000	1.484000	-2.810000
C	0.266000	-0.721000	-2.301000
H	1.481000	2.473000	-2.499000
H	1.963000	0.979000	-3.322000
H	0.333000	1.629000	-3.537000
H	-0.577000	-0.643000	-2.988000
H	1.152000	-1.050000	-2.854000
H	0.075000	-1.530000	-1.598000
C	3.560000	-2.827000	-2.829000
C	2.546000	-3.555000	-3.446000
C	1.481000	-4.046000	-2.694000
C	1.432000	-3.832000	-1.317000
C	2.468000	-3.123000	-0.703000
C	3.517000	-2.609000	-1.454000
C	0.258000	-4.302000	-0.493000
O	-0.398000	-3.216000	0.177000
H	4.378000	-2.426000	-3.418000
H	2.573000	-3.728000	-4.517000
H	0.678000	-4.591000	-3.183000
H	2.452000	-2.939000	0.368000
H	4.287000	-2.026000	-0.962000
H	0.579000	-5.023000	0.268000
H	-0.496000	-4.780000	-1.119000
H	0.266000	-2.646000	0.612000
C	-4.029000	-2.985000	0.946000
O	-3.050000	-2.239000	1.023000
O	-4.481000	-3.632000	1.996000
C	-3.776000	-3.441000	3.236000
H	-4.292000	-4.067000	3.959000
H	-2.736000	-3.753000	3.126000
H	-3.818000	-2.392000	3.532000
C	-4.814000	-3.249000	-0.270000
C	-4.649000	-2.533000	-1.383000
H	-5.259000	-2.740000	-2.256000
H	-3.935000	-1.714000	-1.455000
Mg	-1.519000	-1.372000	0.019000
H	-5.557000	-4.035000	-0.190000

Mg(PBTP)•MA•1a-TS5

O	-1.370000	-2.540000	-0.727000
O	0.731000	-0.568000	1.022000
C	0.704000	0.747000	0.740000
C	1.148000	1.675000	1.712000
C	0.259000	1.207000	-0.527000
C	1.230000	3.025000	1.352000
C	0.369000	2.564000	-0.811000
C	0.865000	3.498000	0.098000
H	1.582000	3.733000	2.090000
H	0.043000	2.916000	-1.782000
C	-2.225000	-1.508000	-0.688000
C	-1.785000	-0.182000	-0.910000
C	-3.598000	-1.734000	-0.348000
C	-2.658000	0.873000	-0.603000
C	-4.413000	-0.638000	-0.101000
C	-3.961000	0.686000	-0.178000
H	-2.270000	1.881000	-0.697000
H	-5.445000	-0.810000	0.184000
C	-0.424000	0.256000	-1.530000
C	-4.154000	-3.167000	-0.253000
C	-3.907000	-3.908000	-1.582000
C	-3.489000	-3.942000	0.902000
C	-5.670000	-3.184000	0.006000
H	-2.841000	-3.935000	-1.814000
H	-4.281000	-4.936000	-1.518000
H	-4.433000	-3.406000	-2.401000
H	-3.603000	-3.399000	1.847000
H	-3.960000	-4.924000	1.021000
H	-2.428000	-4.096000	0.702000
H	-6.022000	-4.221000	0.009000
H	-5.928000	-2.747000	0.977000
H	-6.223000	-2.648000	-0.772000
C	1.480000	1.240000	3.155000
C	0.253000	0.549000	3.782000
C	2.692000	0.291000	3.193000
C	1.827000	2.438000	4.056000
H	-0.601000	1.234000	3.803000
H	-0.032000	-0.338000	3.214000
H	0.473000	0.248000	4.812000
H	3.567000	0.769000	2.740000
H	2.946000	0.045000	4.231000
H	2.488000	-0.639000	2.664000
H	2.016000	2.077000	5.072000
H	2.729000	2.960000	3.720000
H	1.008000	3.162000	4.110000
C	-4.895000	1.845000	0.191000
C	-5.346000	1.690000	1.656000
C	-4.207000	3.210000	0.046000
C	-6.132000	1.829000	-0.727000
H	-5.879000	0.748000	1.816000
H	-4.482000	1.707000	2.329000
H	-6.018000	2.508000	1.940000
H	-3.893000	3.398000	-0.986000
H	-4.903000	4.005000	0.332000
H	-3.325000	3.289000	0.690000
H	-6.810000	2.651000	-0.471000
H	-5.837000	1.941000	-1.775000
H	-6.691000	0.893000	-0.633000
C	0.922000	4.981000	-0.291000
C	1.491000	5.857000	0.833000
C	1.814000	5.161000	-1.533000
C	-0.503000	5.476000	-0.612000
H	0.870000	5.814000	1.734000
H	2.509000	5.558000	1.102000
H	1.526000	6.901000	0.505000
H	1.445000	4.579000	-2.384000
H	1.842000	6.213000	-1.838000
H	2.840000	4.842000	-1.321000
H	-0.489000	6.537000	-0.885000
H	-0.945000	4.921000	-1.444000
H	-1.157000	5.354000	0.257000
C	-0.792000	0.972000	-2.855000
C	0.545000	-0.853000	-1.994000
H	-1.504000	1.790000	-2.725000
H	0.103000	1.364000	-3.349000
H	-1.255000	0.241000	-3.524000
H	0.020000	-1.611000	-2.577000
H	1.315000	-0.392000	-2.620000
H	1.084000	-1.351000	-1.194000
C	2.476000	-3.564000	0.554000
O	1.314000	-3.481000	1.052000
O	3.483000	-4.060000	1.286000
C	3.370000	-3.904000	2.701000
H	4.280000	-4.331000	3.117000
H	3.302000	-2.843000	2.955000
H	2.495000	-4.433000	3.080000
C	2.693000	-3.878000	-0.889000
C	1.690000	-4.061000	-1.739000
H	1.884000	-4.298000	-2.780000
H	0.650000	-3.966000	-1.440000
Mg	-0.163000	-2.233000	0.717000
H	3.729000	-3.980000	-1.194000
C	3.648000	0.999000	-3.499000
C	4.095000	-0.321000	-3.537000
C	4.280000	-1.025000	-2.352000
C	4.021000	-0.422000	-1.119000
C	3.580000	0.901000	-1.090000
C	3.391000	1.608000	-2.274000
C	4.173000	-1.196000	0.165000
O	2.954000	-1.810000	0.590000
H	3.501000	1.550000	-4.422000
H	4.301000	-0.800000	-4.489000
H	4.632000	-2.052000	-2.385000
H	3.360000	1.381000	-0.139000
H	3.031000	2.629000	-2.235000
H	4.520000	-0.545000	0.974000
H	4.891000	-2.014000	0.058000
H	2.172000	-1.184000	0.714000

Mg(PBTP)•MA•1a-Int6

Mg	-1.094000	-1.577000	1.482000
O	-1.699000	0.136000	0.639000
O	0.749000	-1.538000	1.222000
C	1.884000	-1.417000	0.555000
C	3.073000	-2.013000	1.055000
C	1.944000	-0.678000	-0.662000
C	4.269000	-1.815000	0.362000
C	3.171000	-0.526000	-1.300000
C	4.358000	-1.070000	-0.810000
H	5.171000	-2.260000	0.760000
H	3.210000	0.051000	-2.217000
C	-0.911000	1.259000	0.443000
C	0.259000	1.144000	-0.308000
C	-1.251000	2.424000	1.163000
C	1.161000	2.215000	-0.229000
C	-0.334000	3.469000	1.135000
C	0.898000	3.376000	0.477000
H	2.114000	2.102000	-0.731000
H	-0.560000	4.380000	1.673000
C	0.679000	-0.021000	-1.244000
C	-2.559000	2.538000	1.975000
C	-3.792000	2.316000	1.076000
C	-2.578000	1.515000	3.130000
C	-2.709000	3.935000	2.604000
H	-3.850000	1.296000	0.694000
H	-4.707000	2.493000	1.651000
H	-3.786000	3.014000	0.232000
H	-1.663000	1.585000	3.728000
H	-3.430000	1.714000	3.789000
H	-2.687000	0.496000	2.757000
H	-3.659000	3.983000	3.143000
H	-1.912000	4.148000	3.323000
H	-2.716000	4.725000	1.847000
C	3.046000	-2.862000	2.340000
C	2.579000	-2.007000	3.534000
C	2.108000	-4.069000	2.145000
C	4.430000	-3.424000	2.705000
H	3.276000	-1.180000	3.704000
H	1.590000	-1.587000	3.349000
H	2.545000	-2.614000	4.446000
H	2.489000	-4.722000	1.353000
H	2.045000	-4.658000	3.068000
H	1.105000	-3.745000	1.866000
H	4.344000	-4.023000	3.618000
H	4.831000	-4.074000	1.920000
H	5.158000	-2.629000	2.901000
C	1.895000	4.539000	0.561000
C	2.257000	4.793000	2.037000
C	3.192000	4.248000	-0.207000
C	1.253000	5.805000	-0.036000
H	1.378000	5.057000	2.631000
H	2.712000	3.903000	2.482000
H	2.973000	5.619000	2.115000
H	3.004000	4.090000	-1.275000
H	3.872000	5.101000	-0.113000
H	3.708000	3.366000	0.185000
H	1.954000	6.646000	0.015000
H	0.986000	5.646000	-1.085000
H	0.347000	6.094000	0.504000
C	5.675000	-0.817000	-1.554000
C	6.870000	-1.492000	-0.866000
C	5.577000	-1.366000	-2.990000
C	5.947000	0.699000	-1.607000
H	7.014000	-1.118000	0.153000

H	6.749000	-2.579000	-0.819000
H	7.786000	-1.283000	-1.430000
H	4.767000	-0.889000	-3.550000
H	6.511000	-1.187000	-3.535000
H	5.388000	-2.444000	-2.979000
H	6.886000	0.904000	-2.135000
H	5.147000	1.234000	-2.127000
H	6.025000	1.113000	-0.596000
C	0.926000	0.630000	-2.630000
C	-0.379000	-1.101000	-1.562000
H	1.649000	1.448000	-2.616000
H	1.275000	-0.118000	-3.348000
H	-0.021000	1.033000	-2.998000
H	-1.344000	-0.667000	-1.826000
H	-0.028000	-1.668000	-2.429000
H	-0.519000	-1.837000	-0.772000
C	-2.382000	0.678000	-4.630000
C	-2.756000	1.518000	-3.584000
C	-3.521000	1.021000	-2.533000
C	-3.915000	-0.319000	-2.513000
C	-3.541000	-1.151000	-3.569000
C	-2.779000	-0.657000	-4.623000
C	-4.631000	-0.877000	-1.312000
O	-3.634000	-1.165000	-0.333000
H	-1.782000	1.063000	-5.448000
H	-2.450000	2.560000	-3.585000
H	-3.811000	1.677000	-1.717000
H	-3.843000	-2.195000	-3.559000
H	-2.492000	-1.313000	-5.438000
H	-5.163000	-1.799000	-1.566000
H	-5.350000	-0.152000	-0.911000
C	-3.911000	-2.220000	0.676000
O	-2.816000	-2.370000	1.400000
O	-4.288000	-3.371000	-0.042000
C	-3.235000	-3.963000	-0.782000
H	-3.681000	-4.776000	-1.356000
H	-2.778000	-3.241000	-1.471000
H	-2.459000	-4.365000	-0.124000
C	-5.147000	-1.799000	1.449000
C	-5.112000	-1.404000	2.715000
H	-6.082000	-1.820000	0.895000
H	-6.009000	-1.085000	3.236000
H	-4.177000	-1.398000	3.266000
H	-2.473000	-0.060000	0.055000

Na(BHT)•2(MMA)•1a

C	-3.050000	-2.197000	1.282000
O	-1.989000	-2.547000	1.779000
O	0.319000	-0.723000	-0.592000
O	-1.824000	0.196000	0.561000
C	1.563000	-0.563000	-0.986000
C	2.448000	-1.692000	-1.089000
C	2.087000	0.724000	-1.349000
C	3.803000	-1.474000	-1.345000
C	3.450000	0.858000	-1.607000
C	4.340000	-0.211000	-1.573000
H	4.483000	-2.318000	-1.380000
H	3.854000	1.838000	-1.843000
H	-1.107000	0.167000	-0.119000
C	-2.570000	1.389000	0.537000
H	-3.370000	1.254000	1.276000
O	-3.356000	-2.402000	0.008000
C	-2.296000	-2.885000	-0.829000
H	-1.916000	-3.838000	-0.456000
H	-2.738000	-3.012000	-1.816000
H	-1.489000	-2.146000	-0.857000
C	-4.152000	-1.511000	2.021000
C	-3.979000	-1.308000	3.328000
H	-4.734000	-0.806000	3.924000
H	-3.078000	-1.647000	3.828000
H	-1.967000	2.244000	0.872000
C	-3.164000	1.707000	-0.816000
C	-3.540000	0.685000	-1.688000
C	-3.343000	3.034000	-1.206000
C	-4.083000	0.987000	-2.933000
H	-3.397000	-0.347000	-1.385000
C	-3.893000	3.338000	-2.448000
H	-3.039000	3.836000	-0.538000
C	-4.261000	2.314000	-3.317000
H	-4.365000	0.184000	-3.607000
H	-4.025000	4.375000	-2.741000
H	-4.682000	2.548000	-4.290000
C	5.821000	-0.010000	-1.770000
H	6.342000	0.155000	-0.817000
H	6.286000	-0.882000	-2.240000
H	6.028000	0.860000	-2.402000
C	1.910000	-3.136000	-1.027000
C	1.220000	-3.463000	0.311000
C	0.908000	-3.331000	-2.183000
C	3.021000	-4.185000	-1.212000
H	1.829000	-3.127000	1.158000
H	0.232000	-3.011000	0.365000
H	1.080000	-4.546000	0.415000
H	1.425000	-3.282000	-3.147000
H	0.416000	-4.309000	-2.111000
H	0.147000	-2.550000	-2.164000
H	2.572000	-5.184000	-1.214000
H	3.550000	-4.061000	-2.161000
H	3.756000	-4.154000	-0.400000
C	1.167000	1.952000	-1.465000
C	-0.009000	1.639000	-2.412000
C	0.678000	2.376000	-0.069000
C	1.881000	3.179000	-2.061000
H	-0.535000	0.730000	-2.120000
H	-0.727000	2.466000	-2.429000
H	0.369000	1.494000	-3.430000
H	1.518000	2.760000	0.519000
H	-0.072000	3.172000	-0.143000
H	0.245000	1.536000	0.475000
H	1.156000	3.992000	-2.171000
H	2.687000	3.545000	-1.417000
H	2.299000	2.968000	-3.050000
Na	-0.135000	-1.090000	1.631000
C	1.957000	1.215000	2.807000
O	1.414000	0.124000	2.717000
O	1.326000	2.285000	3.285000
C	-0.050000	2.106000	3.632000
H	-0.627000	1.816000	2.750000
H	-0.155000	1.342000	4.405000
H	-0.388000	3.071000	4.003000
C	3.356000	1.507000	2.406000
C	3.969000	0.607000	1.634000
H	4.981000	0.772000	1.280000
H	3.467000	-0.296000	1.301000
C	-5.367000	-1.078000	1.251000
C	3.965000	2.816000	2.829000
H	-5.094000	-0.388000	0.447000
H	-5.861000	-1.933000	0.782000
H	-6.080000	-0.580000	1.911000
H	3.430000	3.658000	2.379000
H	3.917000	2.947000	3.914000
H	5.010000	2.866000	2.517000

Na(BHT)•2(MMA)•1a-TS6

C	-2.474000	-2.401000	-0.592000
O	-1.348000	-2.570000	-1.101000
O	-0.252000	0.208000	-0.168000
O	-2.174000	-1.113000	0.604000
C	0.695000	1.063000	0.188000
C	0.934000	2.213000	-0.634000
C	1.512000	0.861000	1.341000
C	1.943000	3.104000	-0.275000
C	2.504000	1.801000	1.633000
C	2.741000	2.924000	0.852000
H	2.131000	3.980000	-0.887000
H	3.135000	1.661000	2.503000
H	-1.367000	-0.472000	0.382000
C	-3.207000	-0.584000	1.423000
H	-4.063000	-1.255000	1.310000
O	-3.476000	-1.813000	-1.324000
C	-3.026000	-0.890000	-2.304000
H	-2.390000	-1.382000	-3.045000
H	-3.920000	-0.495000	-2.786000
H	-2.472000	-0.074000	-1.829000
C	-3.071000	-3.465000	0.318000
C	-2.297000	-4.012000	1.253000
H	-2.663000	-4.803000	1.901000
H	-1.277000	-3.671000	1.398000
H	-2.891000	-0.631000	2.471000
C	-3.608000	0.825000	1.060000
C	-4.494000	1.052000	0.003000
C	-3.109000	1.917000	1.769000
C	-4.862000	2.346000	-0.347000
H	-4.889000	0.200000	-0.542000
C	-3.479000	3.215000	1.424000
H	-2.424000	1.754000	2.597000
C	-4.354000	3.432000	0.364000
H	-5.552000	2.509000	-1.170000
H	-3.079000	4.056000	1.981000
H	-4.641000	4.443000	0.094000
C	3.841000	3.902000	1.185000
H	4.654000	3.863000	0.449000
H	3.475000	4.934000	1.200000
H	4.277000	3.690000	2.166000
C	0.065000	2.493000	-1.877000
C	0.121000	1.318000	-2.877000
C	-1.394000	2.726000	-1.446000
C	0.527000	3.745000	-2.640000
H	1.156000	1.000000	-3.049000
H	-0.460000	0.479000	-2.501000
H	-0.307000	1.616000	-3.841000
H	-1.477000	3.632000	-0.838000
H	-2.041000	2.850000	-2.323000
H	-1.764000	1.887000	-0.855000
H	-0.132000	3.905000	-3.500000
H	0.479000	4.646000	-2.021000
H	1.550000	3.641000	-3.020000
C	1.281000	-0.316000	2.312000
C	-0.021000	-0.058000	3.089000
C	1.181000	-1.677000	1.597000
C	2.419000	-0.457000	3.340000
H	-0.846000	0.101000	2.396000
H	-0.272000	-0.908000	3.734000
H	0.075000	0.835000	3.714000
H	2.040000	-1.828000	0.935000
H	1.193000	-2.486000	2.336000
H	0.260000	-1.770000	1.024000
H	2.220000	-1.323000	3.981000
H	3.387000	-0.616000	2.851000
H	2.501000	0.414000	3.996000
Na	0.536000	-1.472000	-1.443000
C	3.837000	-1.727000	-0.996000
O	2.822000	-1.557000	-1.658000
O	4.196000	-2.925000	-0.545000
C	3.324000	-4.015000	-0.864000
H	2.345000	-3.865000	-0.401000
H	3.207000	-4.106000	-1.945000
H	3.801000	-4.902000	-0.454000
C	4.787000	-0.657000	-0.599000
C	4.390000	0.604000	-0.781000
H	5.025000	1.437000	-0.496000
H	3.413000	0.841000	-1.189000
C	-4.494000	-3.892000	0.076000
C	6.098000	-1.052000	0.023000
H	-5.189000	-3.056000	0.197000
H	-4.623000	-4.259000	-0.947000
H	-4.779000	-4.687000	0.770000
H	5.939000	-1.588000	0.964000
H	6.667000	-1.716000	-0.634000
H	6.701000	-0.165000	0.229000

Na(BHT)•2(MMA)•1a-Int7

C	-2.310000	-2.210000	-0.273000
O	-1.213000	-2.375000	-0.946000
O	-0.172000	0.608000	-0.278000

O	-2.224000	-0.903000	0.442000
C	0.941000	1.228000	0.220000
C	1.364000	2.354000	-0.525000
C	1.651000	0.776000	1.353000
C	2.564000	2.960000	-0.157000
C	2.835000	1.454000	1.666000
C	3.322000	2.519000	0.922000
H	2.929000	3.815000	-0.714000
H	3.417000	1.133000	2.521000
H	-0.734000	0.065000	0.312000
C	-3.379000	-0.436000	1.125000
H	-4.249000	-1.003000	0.785000
O	-3.503000	-2.124000	-1.075000
C	-3.321000	-1.421000	-2.279000
H	-2.672000	-1.965000	-2.976000
H	-4.310000	-1.305000	-2.732000
H	-2.897000	-0.422000	-2.108000
C	-2.577000	-3.341000	0.730000
C	-2.261000	-3.239000	2.020000
H	-2.371000	-4.082000	2.697000
H	-1.861000	-2.319000	2.433000
H	-3.264000	-0.604000	2.204000
C	-3.589000	1.036000	0.863000
C	-4.223000	1.465000	-0.308000
C	-3.168000	1.996000	1.784000
C	-4.424000	2.819000	-0.553000
H	-4.577000	0.725000	-1.019000
C	-3.367000	3.353000	1.543000
H	-2.687000	1.677000	2.705000
C	-3.995000	3.768000	0.373000
H	-4.922000	3.135000	-1.465000
H	-3.033000	4.086000	2.271000
H	-4.153000	4.825000	0.184000
C	4.639000	3.171000	1.260000
H	5.444000	2.793000	0.619000
H	4.595000	4.255000	1.117000
H	4.926000	2.978000	2.297000
C	0.494000	2.966000	-1.644000
C	0.187000	1.963000	-2.779000
C	-0.833000	3.458000	-1.034000
C	1.181000	4.178000	-2.298000
H	1.072000	1.370000	-3.038000
H	-0.638000	1.307000	-2.501000
H	-0.124000	2.500000	-3.681000
H	-0.648000	4.230000	-0.281000
H	-1.470000	3.891000	-1.814000
H	-1.382000	2.641000	-0.564000
H	0.515000	4.596000	-3.058000
H	1.389000	4.971000	-1.574000
H	2.119000	3.903000	-2.792000
C	1.161000	-0.362000	2.272000
C	-0.172000	0.053000	2.927000
C	1.008000	-1.694000	1.516000
C	2.148000	-0.635000	3.422000
H	-0.972000	0.190000	2.201000
H	-0.502000	-0.719000	3.631000
H	-0.050000	0.991000	3.478000
H	1.978000	-2.014000	1.125000
H	0.665000	-2.469000	2.210000
H	0.285000	-1.678000	0.700000
H	1.756000	-1.452000	4.035000
H	3.130000	-0.945000	3.049000
H	2.278000	0.233000	4.075000
Na	0.055000	-0.970000	-2.001000
C	3.020000	-2.038000	-1.249000
O	2.274000	-1.420000	-1.997000
O	2.823000	-3.307000	-0.920000
C	1.629000	-3.933000	-1.417000
H	0.736000	-3.467000	-0.987000
H	1.596000	-3.864000	-2.507000
H	1.702000	-4.974000	-1.110000
C	4.229000	-1.462000	-0.601000
C	4.431000	-0.151000	-0.743000
H	5.280000	0.337000	-0.276000
H	3.741000	0.467000	-1.307000
C	-3.097000	-4.617000	0.128000
C	5.107000	-2.369000	0.217000
H	-4.100000	-4.473000	-0.283000
H	-2.455000	-4.928000	-0.702000
H	-3.127000	-5.423000	0.867000
H	4.552000	-2.791000	1.060000
H	5.475000	-3.210000	-0.378000
H	5.963000	-1.816000	0.608000

9. References

1. T. Söhner, I. K. Küppers, W. Wackerow, F. Rominger, B. F. Straub, *ARKIVOC* **2014** (iv) 296–318.
2. J. Q. Ng, H. Arima, T. Mochizuki, K. Toh, K. Matsui, M. Ratanasak, J. Hasegawa, M. Hatano, K. Ishihara *ACS Catal.* **2021**, *11*, 199–207.
3. Chai, J.-D.; Head-Gordon, M. Long-range corrected hybrid density functionals with damped atom-atom dispersion corrections. *Phys. Chem. Chem. Phys.* **2008**, *10*, 6615–6620.
4. Ditchfield, R.; Hehre, W. J.; Pople, J. A. Self-Consistent Molecular Orbital Methods. IX. Extended Gaussian-type basis for molecular-orbital studies of organic molecules. *J. Chem. Phys.* **1971**, *54*, 724–728.
5. Tomasi, J.; Mennucci, B.; Cammi, R. Quantum mechanical continuum solvation models. Chem. Rev. 2005, 105, 2999–3093.
6. Gaussian 16, Revision A.03, Frisch, M. J.; Trucks, G. W.; Schlegel, H. B.; Scuseria, G. E.; Robb, M. A.; Cheeseman, J. R.; Scalmani, G.; Barone, V.; Petersson, G. A.; Nakatsuji, H.; Li, X.; Caricato, M.; Marenich, A. V.; Bloino, J.; Janesko, B. G.; Gomperts, R.; Mennucci, B.; Hratchian, H. P.; Ortiz, J. V.; Izmaylov, A. F.; Sonnenberg, J. L.; Williams-Young, D.; Ding, F.; Lipparini, F.; Egidi, F.; Goings, J.; Peng, B.; Petrone, A.; Henderson, T.; Ranasinghe, D.; Zakrzewski, V. G.; Gao, J.; Rega, N.; Zheng, G.; Liang, W.; Hada, M.; Ehara, M.; Toyota, K.; Fukuda, R.; Hasegawa, J.; Ishida, M.; Nakajima, T.; Honda, Y.; Kitao, O.; Nakai, H.; Vreven, T.; Throssell, K.; Montgomery, J. A., Jr.; Peralta, J. E.; Ogliaro, F.; Bearpark, M. J.; Heyd, J. J.; Brothers, E. N.; Kudin, K. N.; Staroverov, V. N.; Keith, T. A.; Kobayashi, R.; Normand, J.; Raghavachari, K.; Rendell, A. P.; Burant, J. C.; Iyengar, S. S.; Tomasi, J.; Cossi, M.; Millam, J. M.; Klene, M.; Adamo, C.; Cammi, R.; Ochterski, J. W.; Martin, R. L.; Morokuma, K.; Farkas, O.; Foresman, J. B.; Fox, D. J. Gaussian, Inc., Wallingford CT, 2016.

^{1}H and ^{13}C NMR spectra of PBTP-H_2

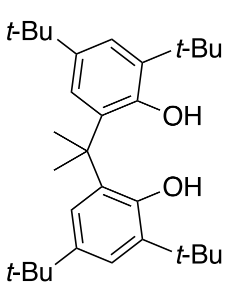

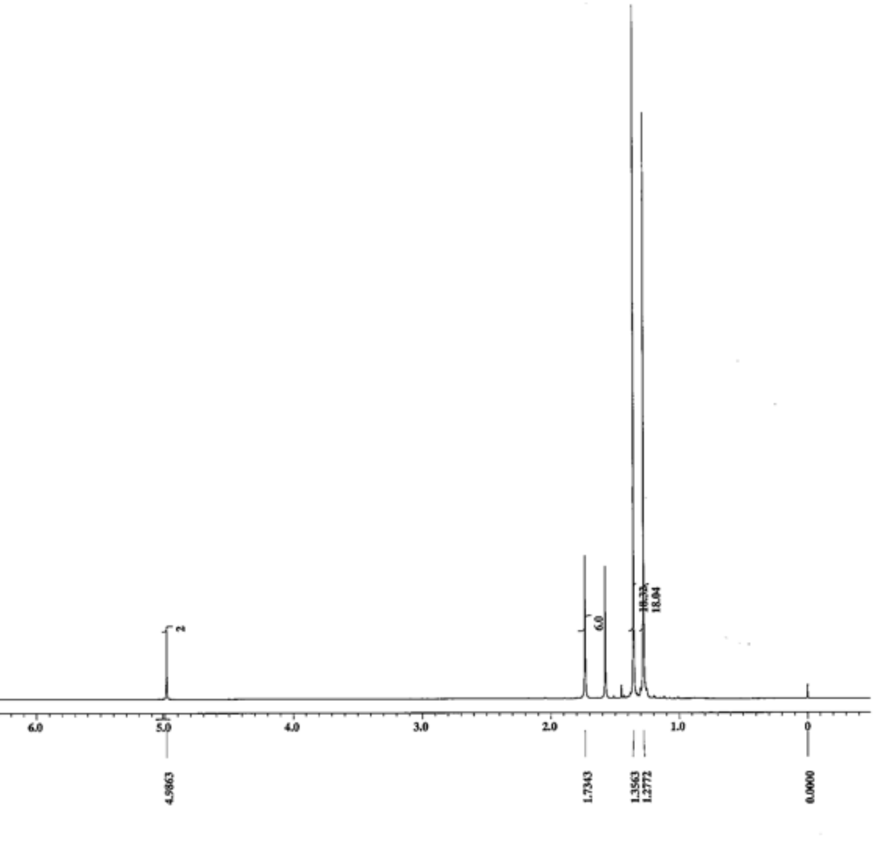

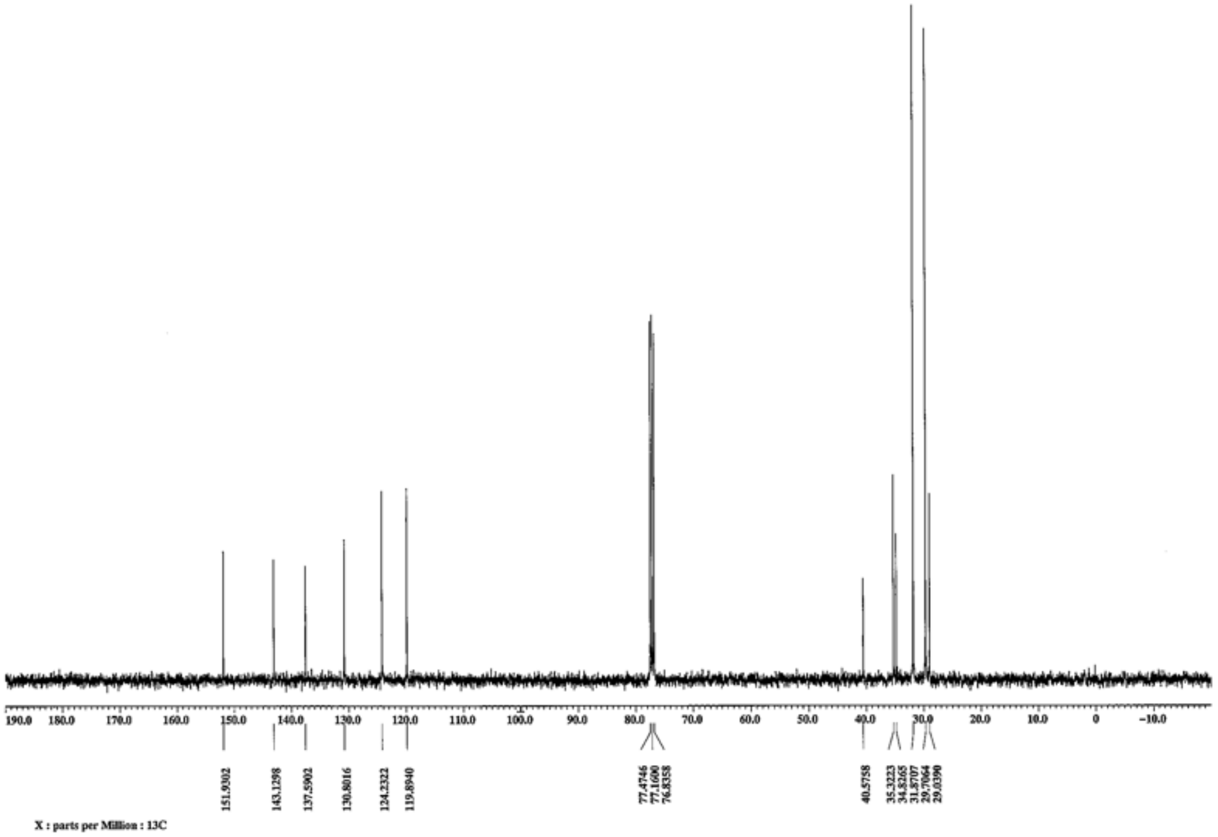

^{1}H and ^{13}C NMR spectra of Mg(PBTP)

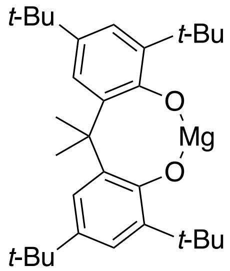

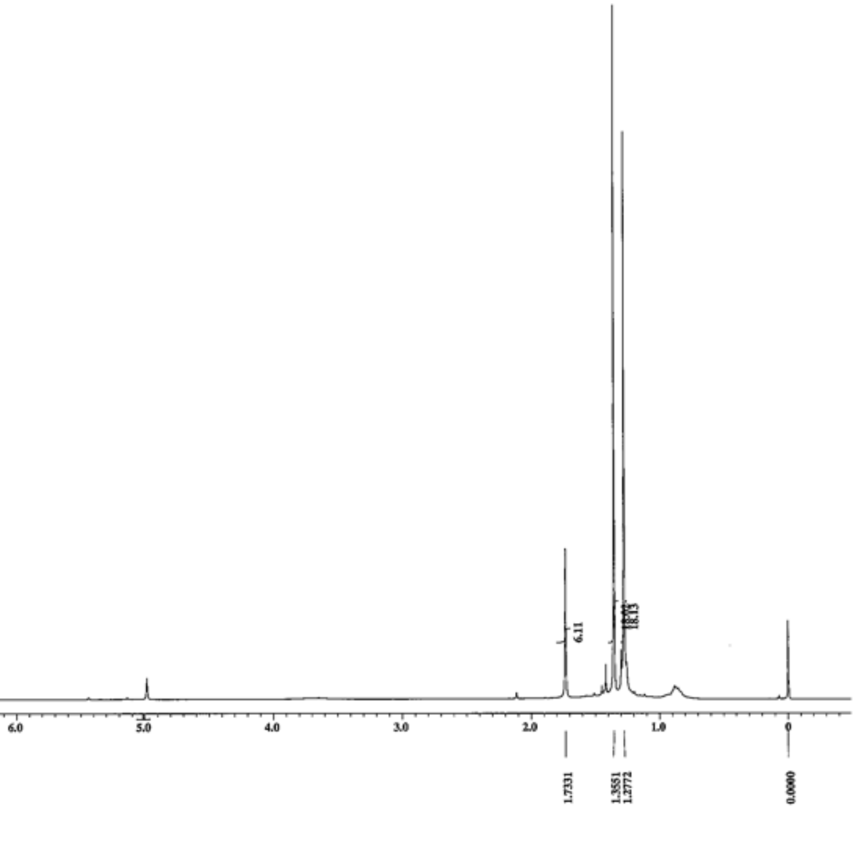

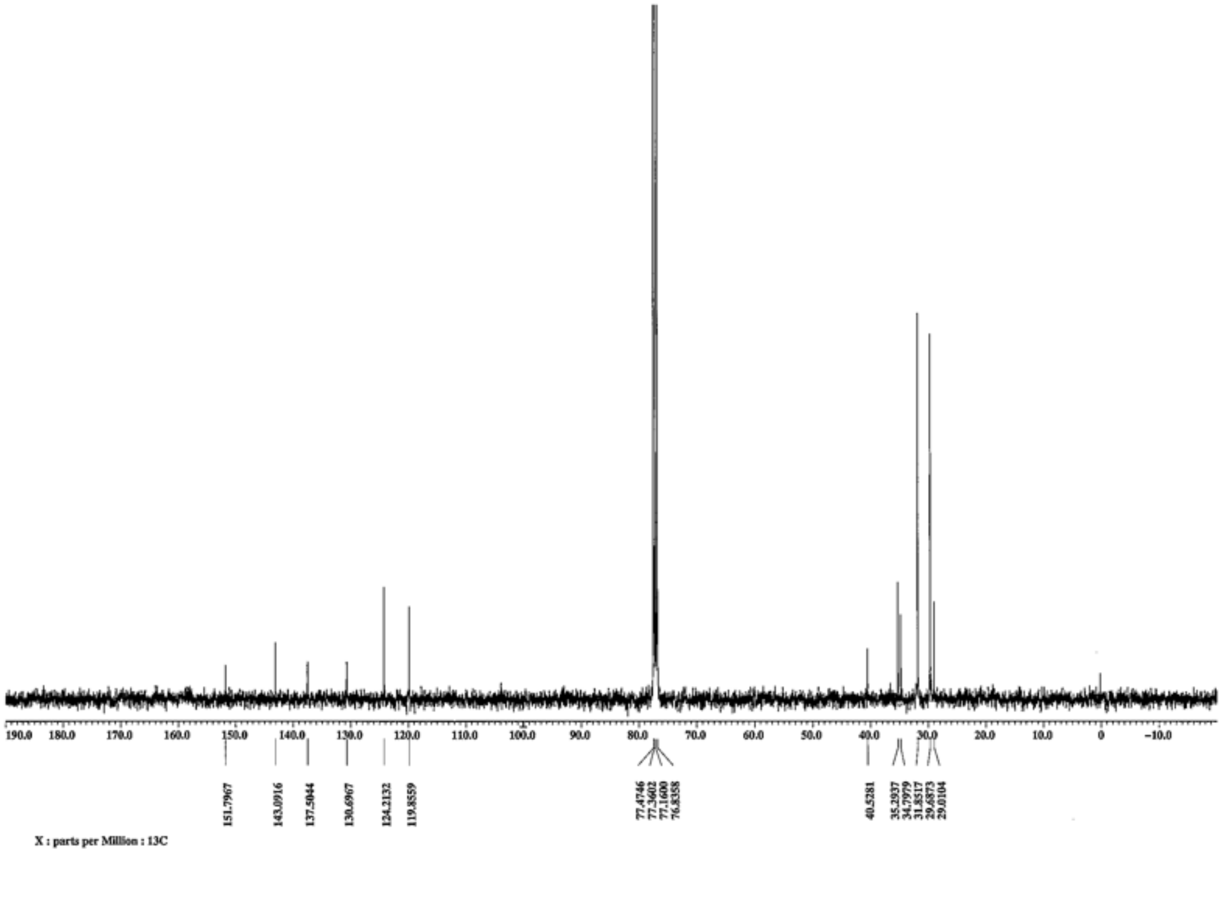

^{1}H and ^{13}C NMR spectra of Na_2(PBTP)

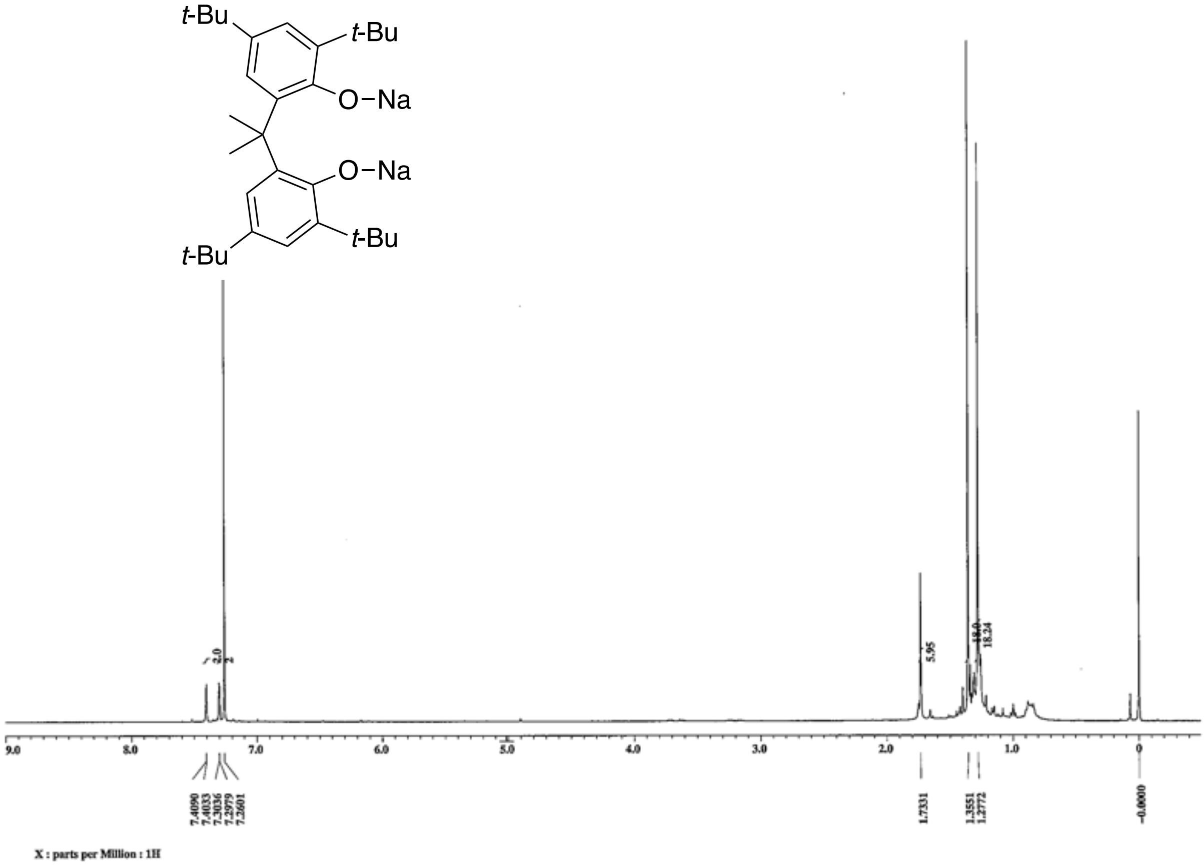

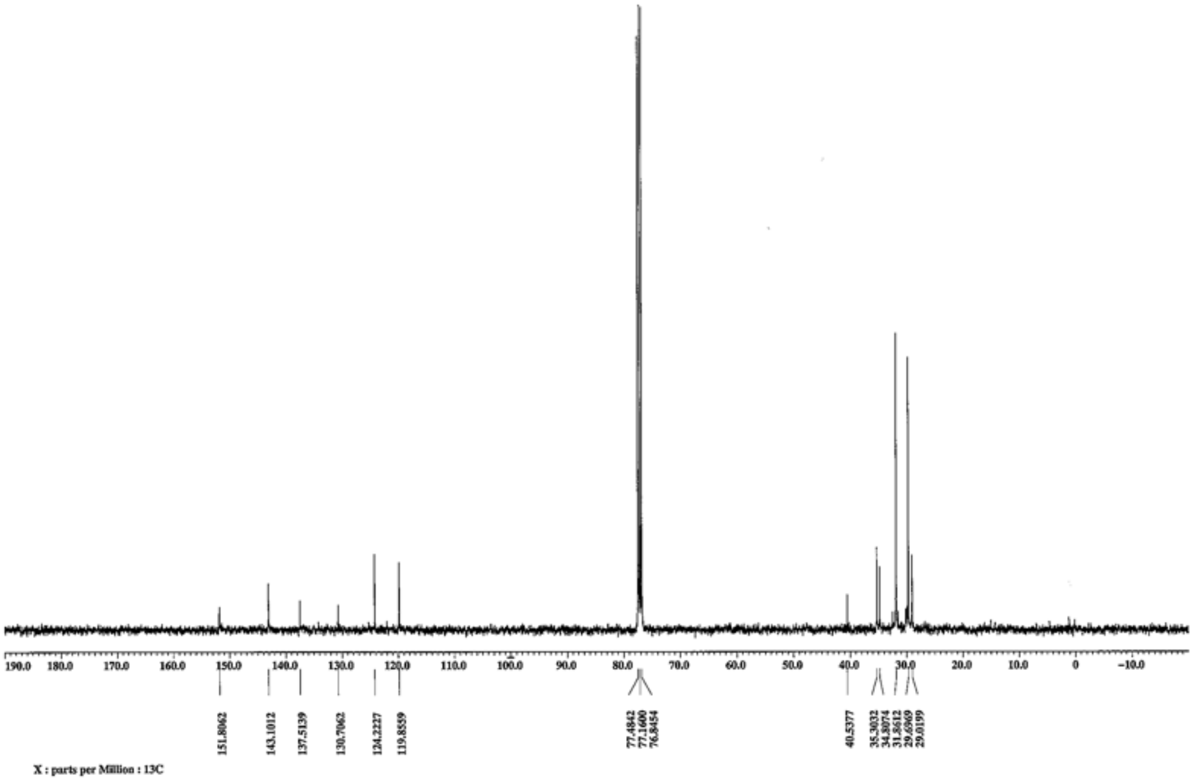

^{1}H and ^{13}C NMR spectra of benzyl acrylate (2a)

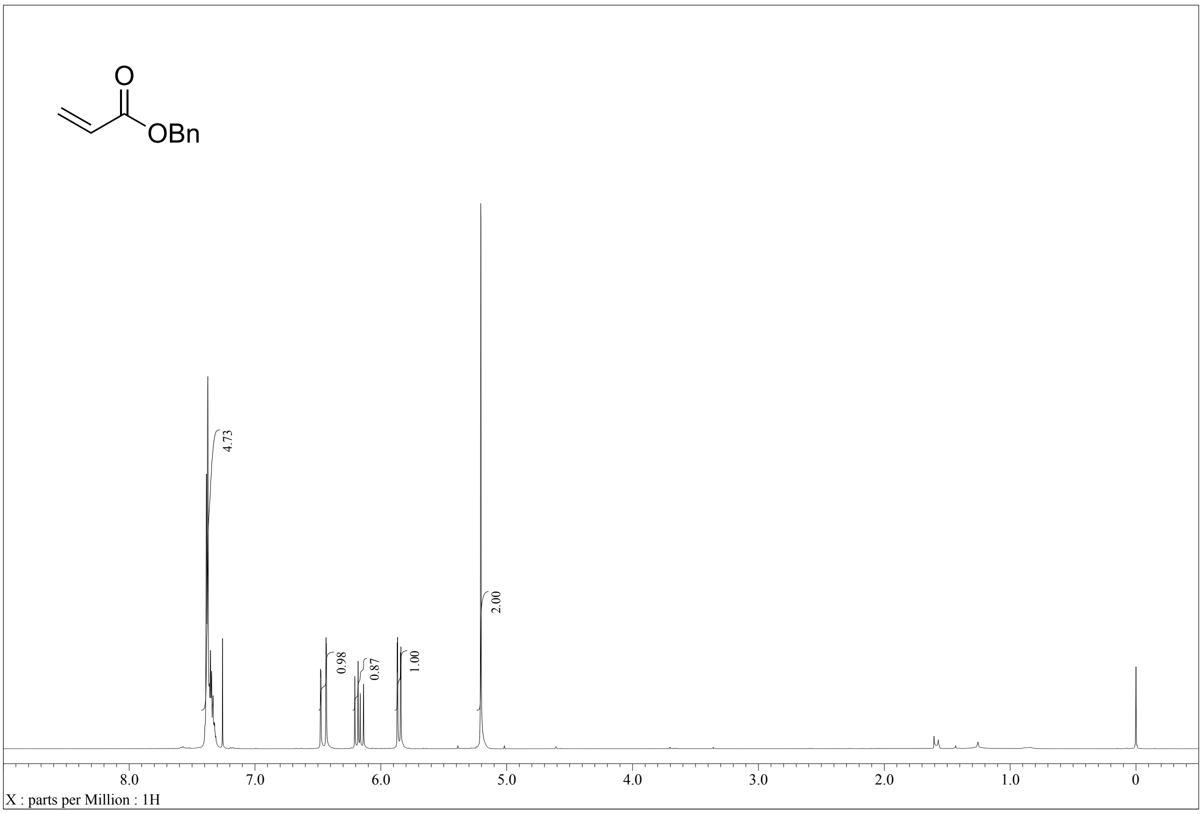

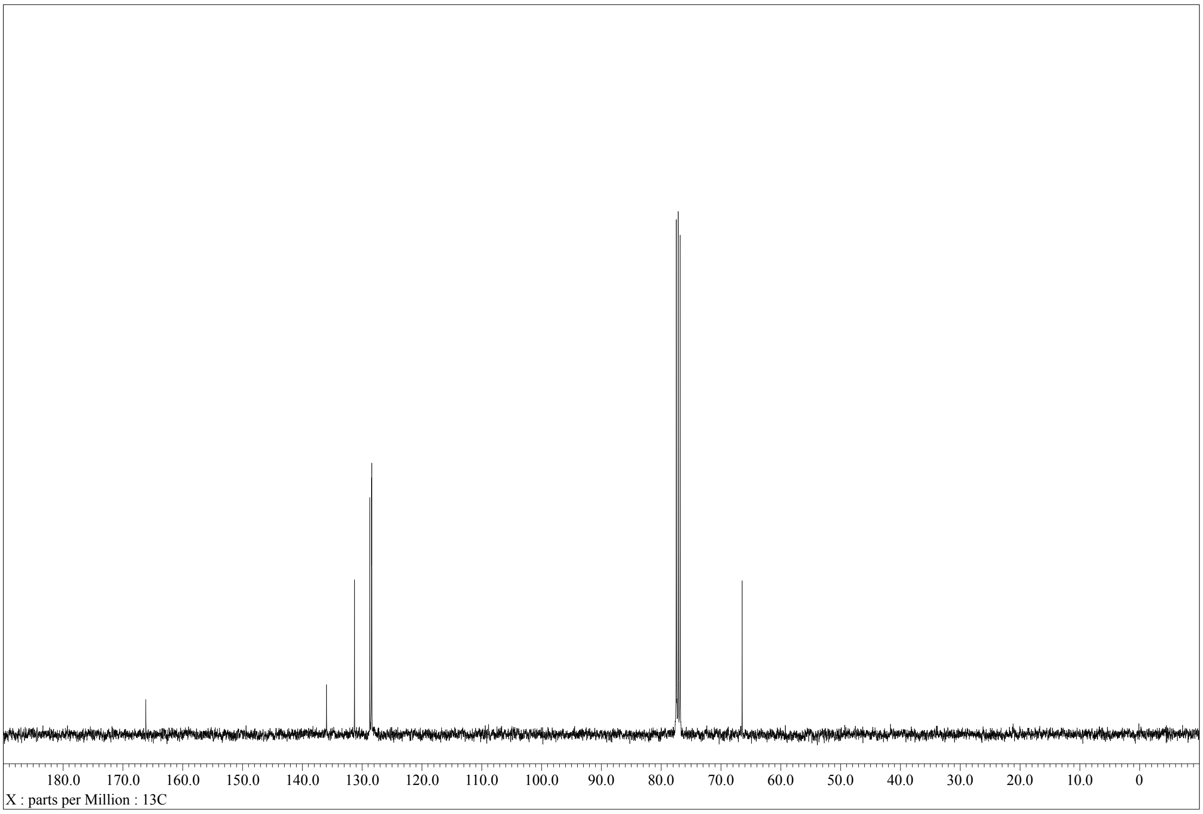

^{1}H and ^{13}C NMR spectra of benzyl 3-(benzyloxy)propanoate (3a)

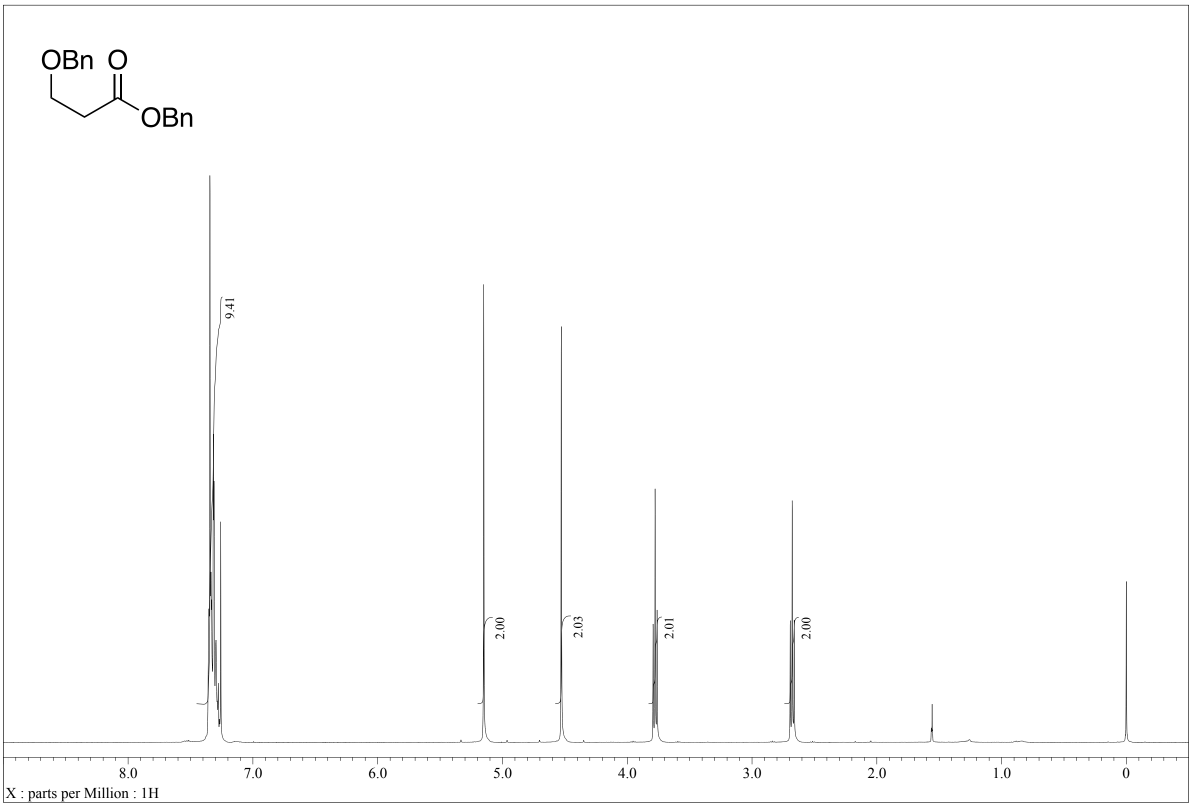

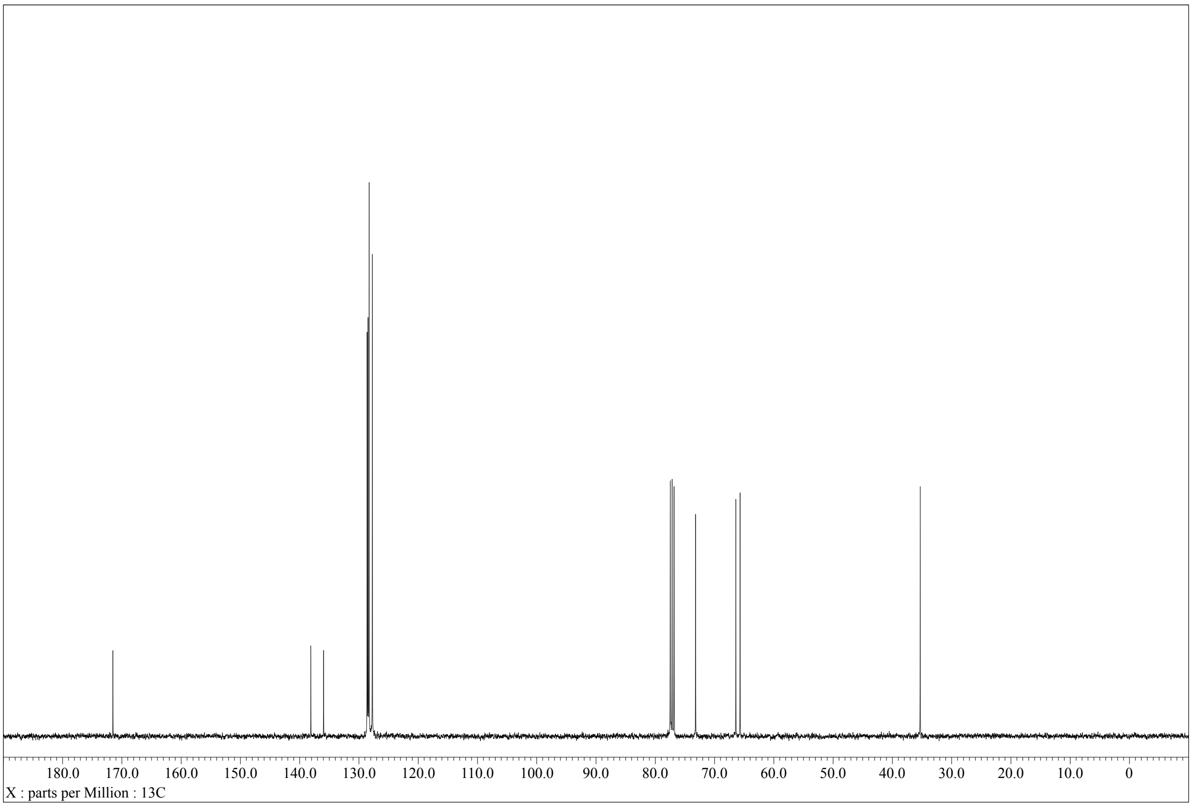

^{1}H and ^{13}C NMR spectra of Benzyl 3-methoxypropanoate (4a):

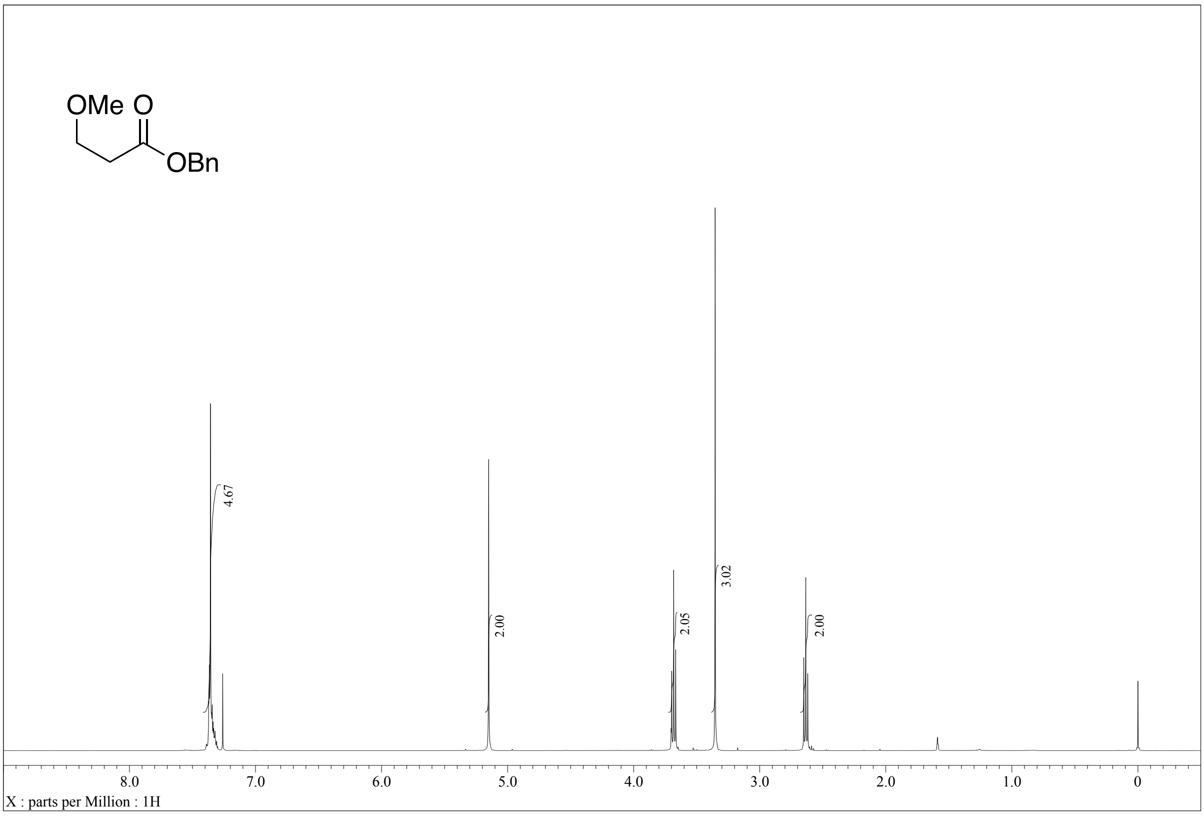

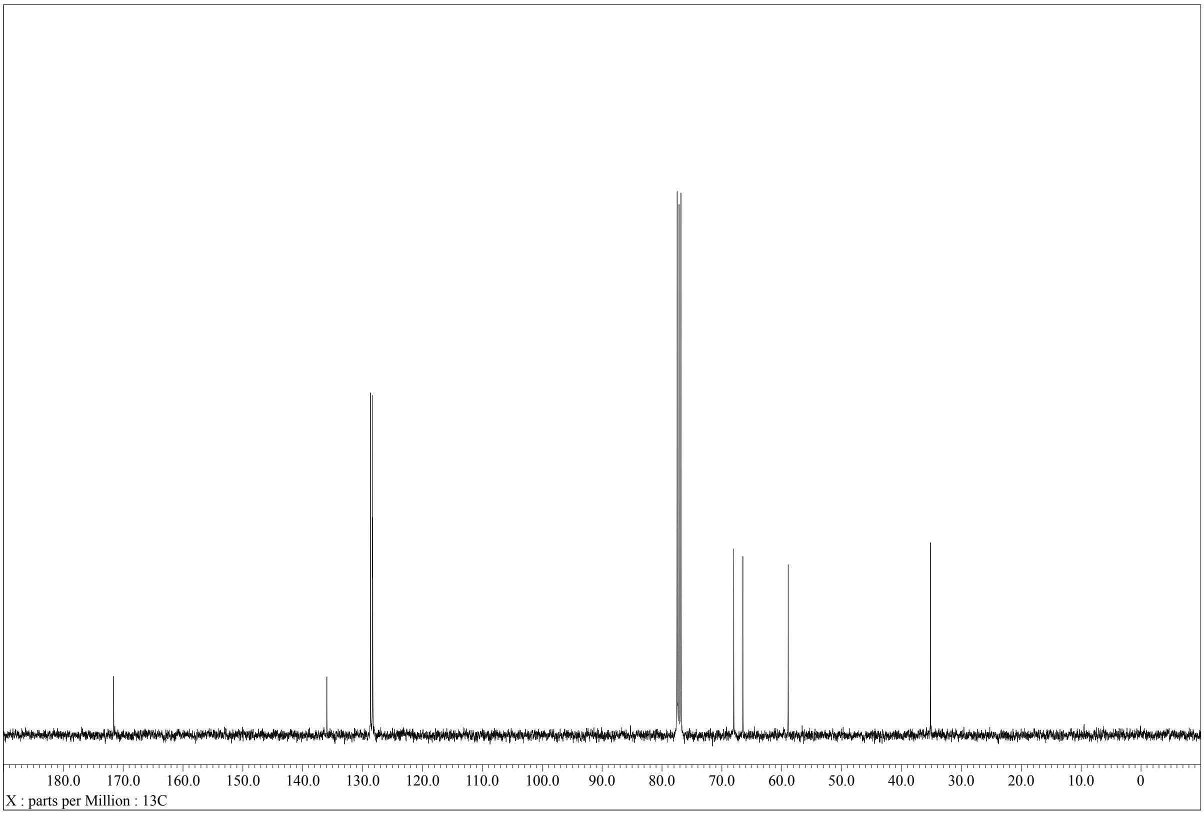

^{1}H and ^{13}C NMR spectra of Methyl 3-(benzyloxy)propanoate (5a)

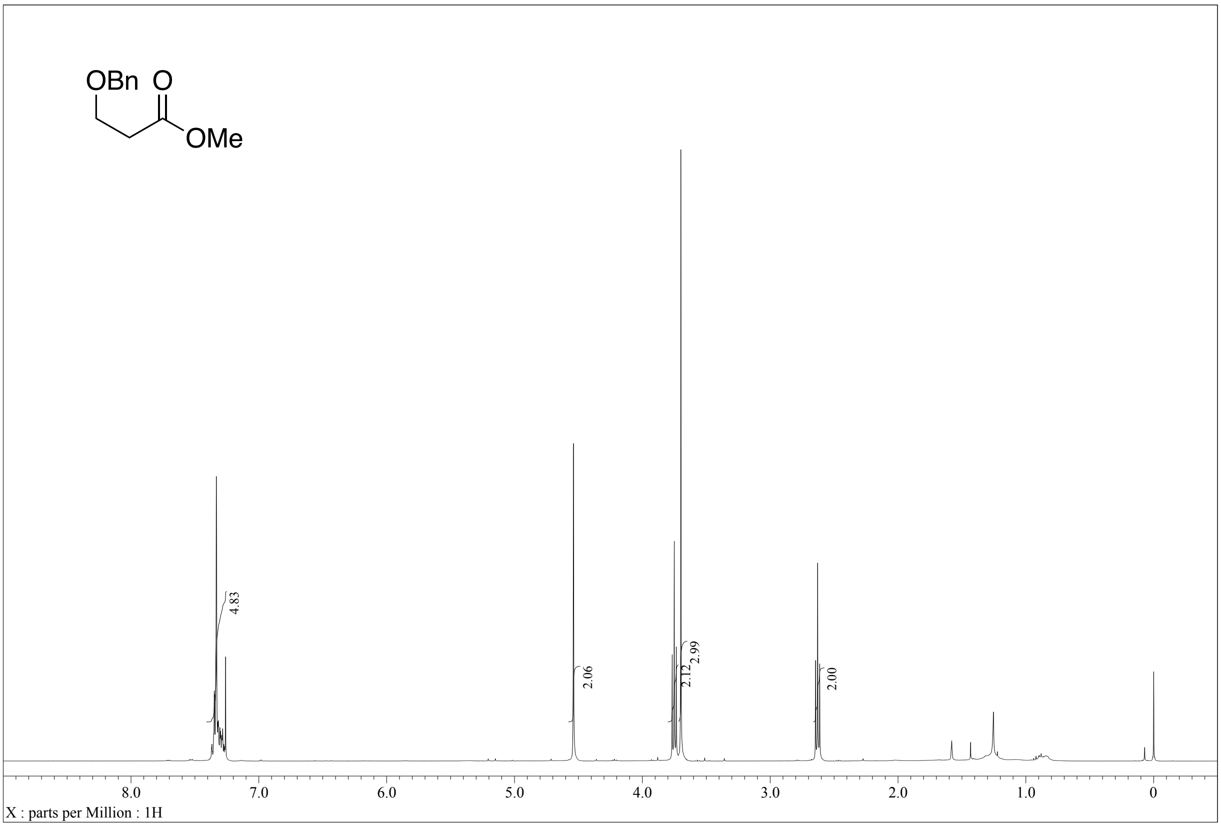

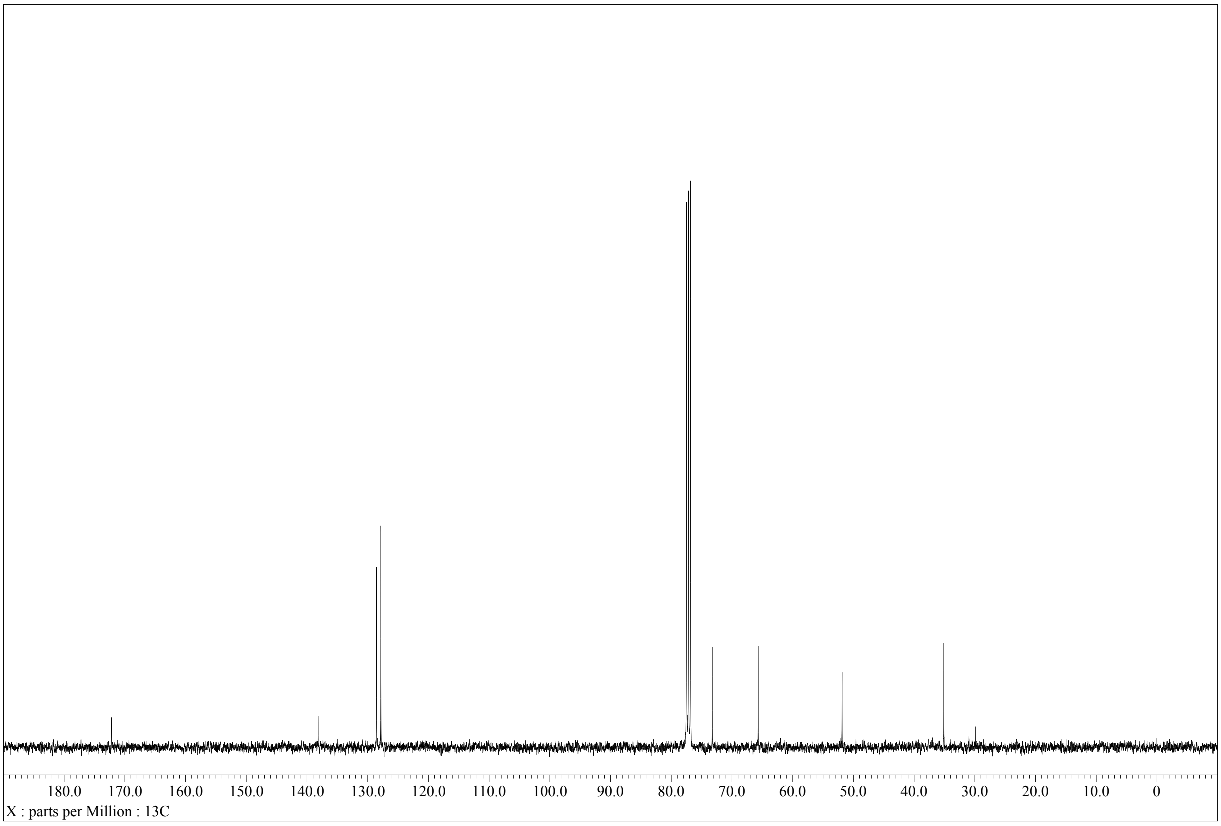

^{1}H and ^{13}C NMR spectra of Dodecyl acrylate (2c)

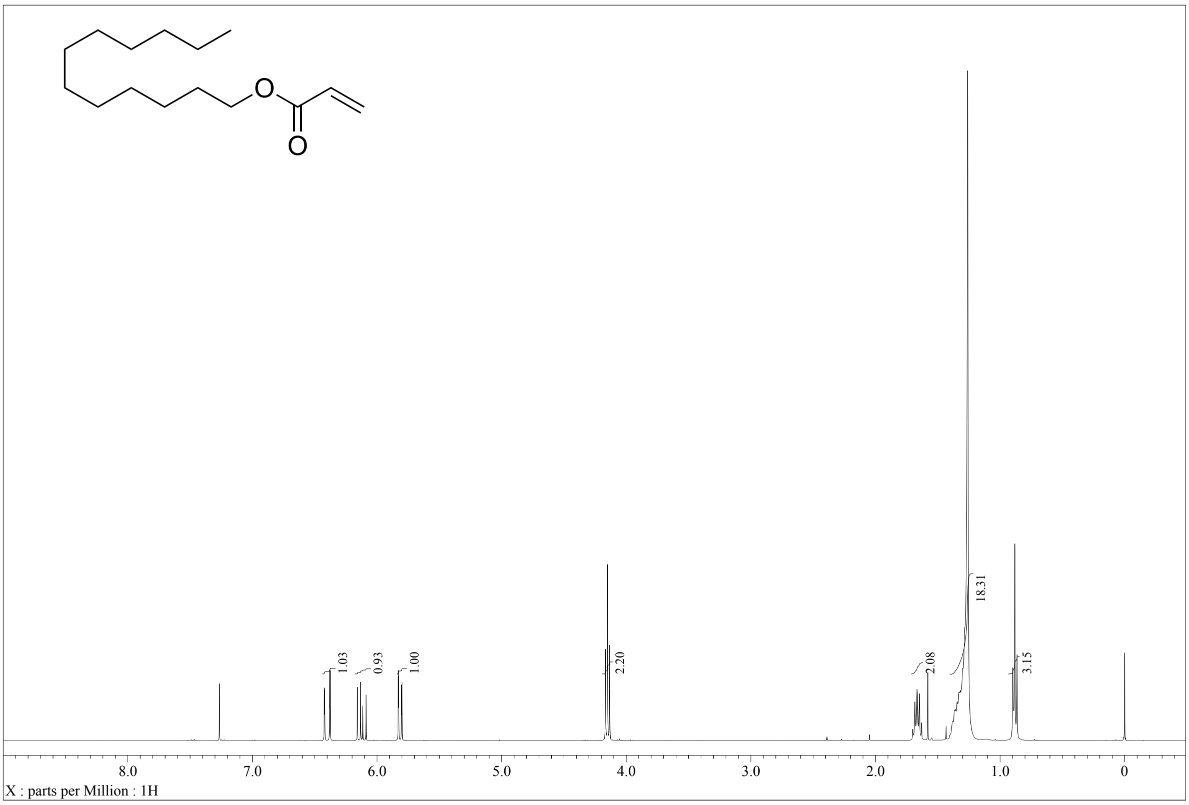

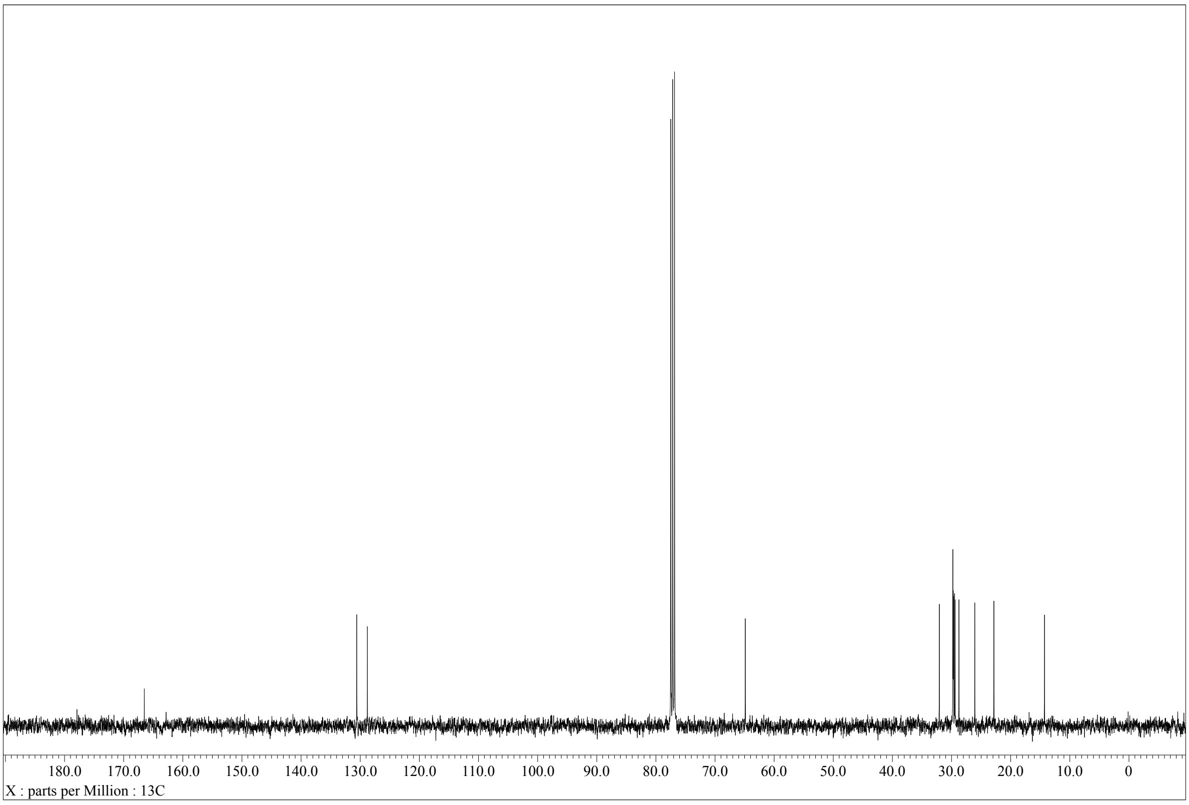

^{1}H and ^{13}C NMR spectra of Thiophen-2-ylmethyl acrylate (2d)

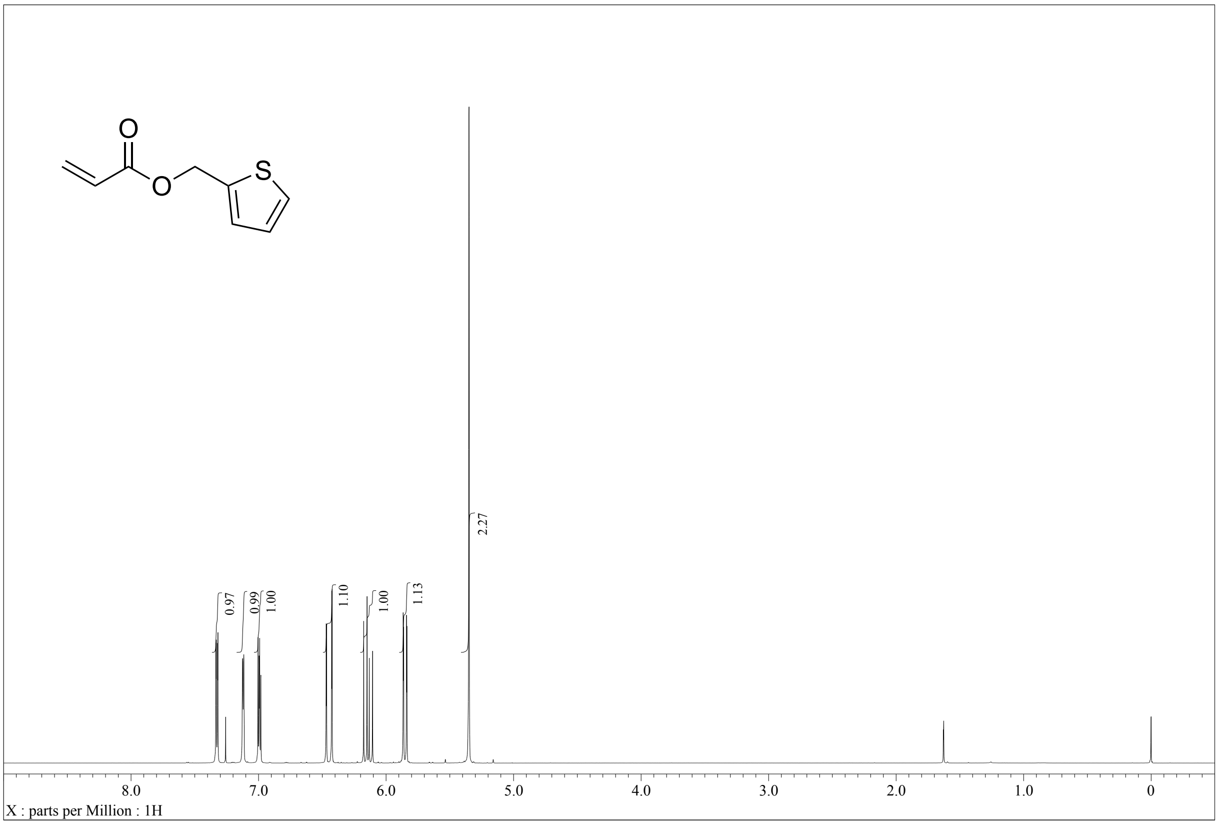

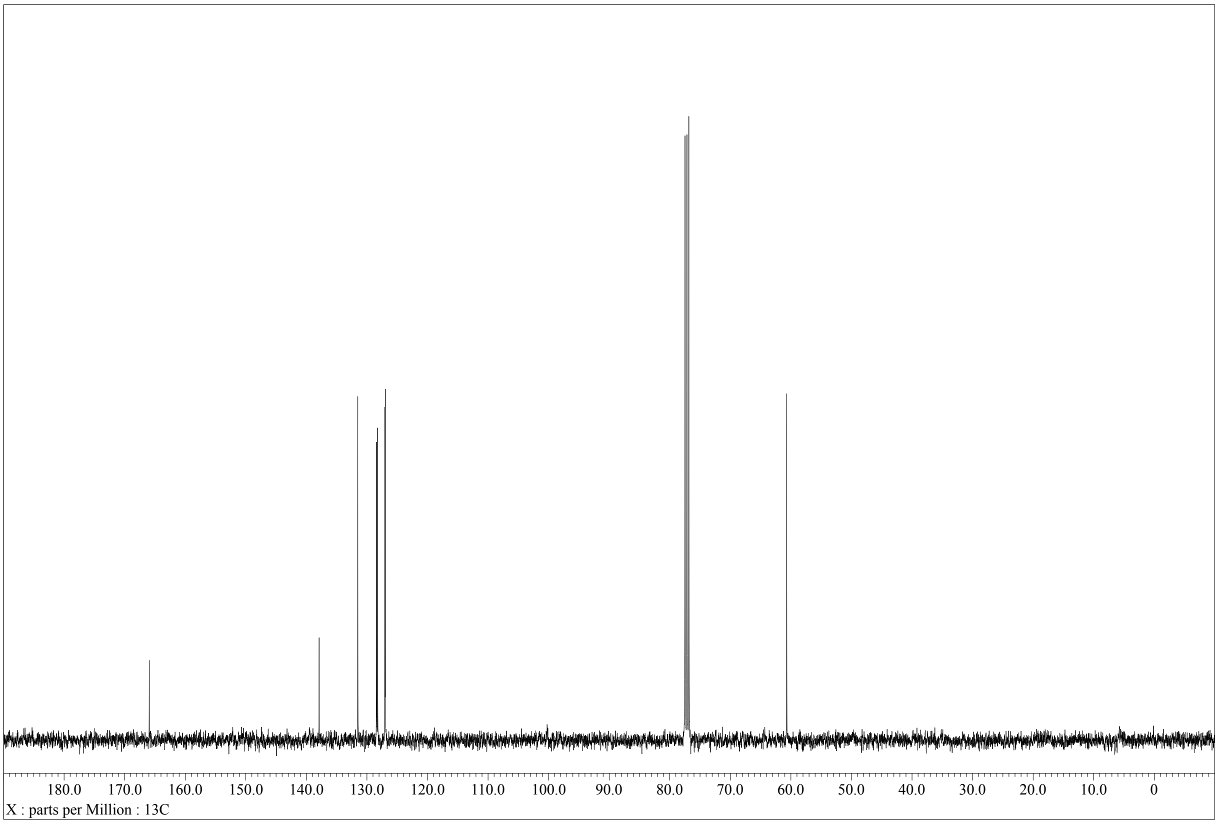

^{1}H and ^{13}C NMR spectra of Geranyl acrylate (2e)

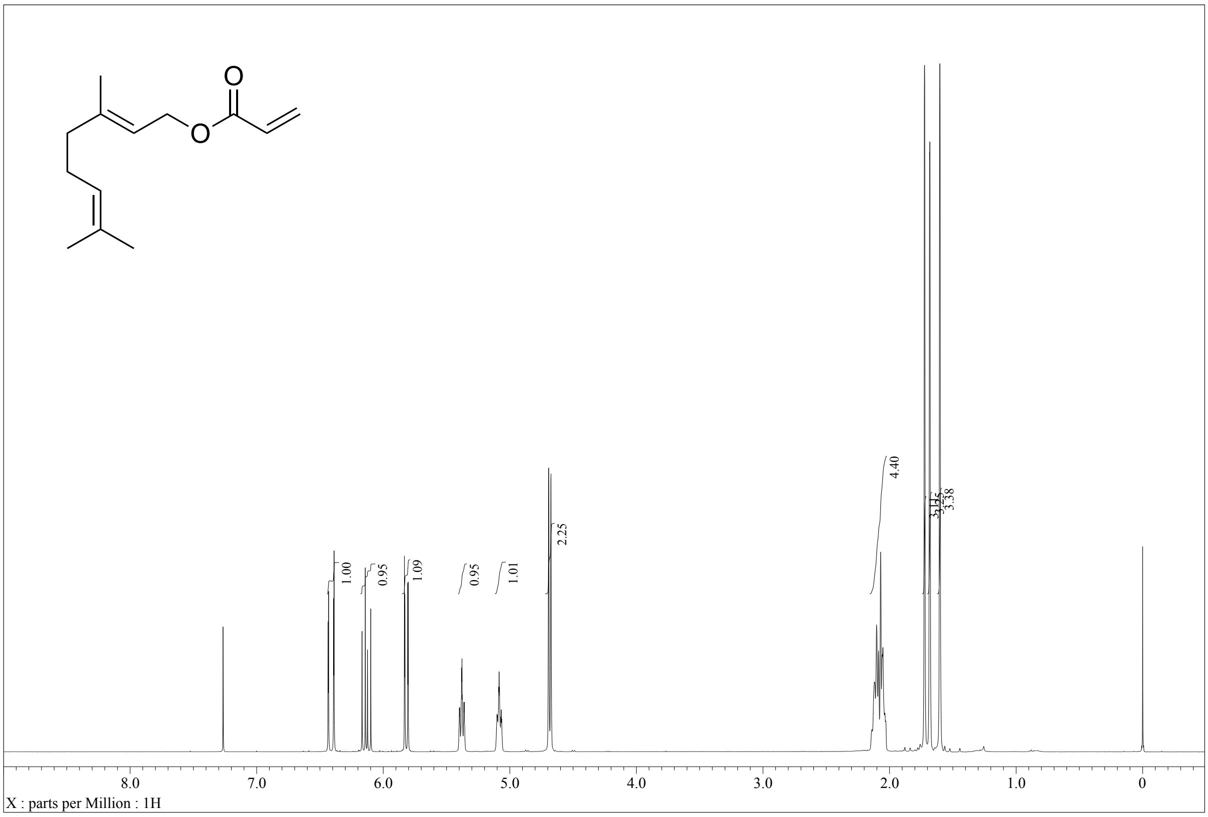

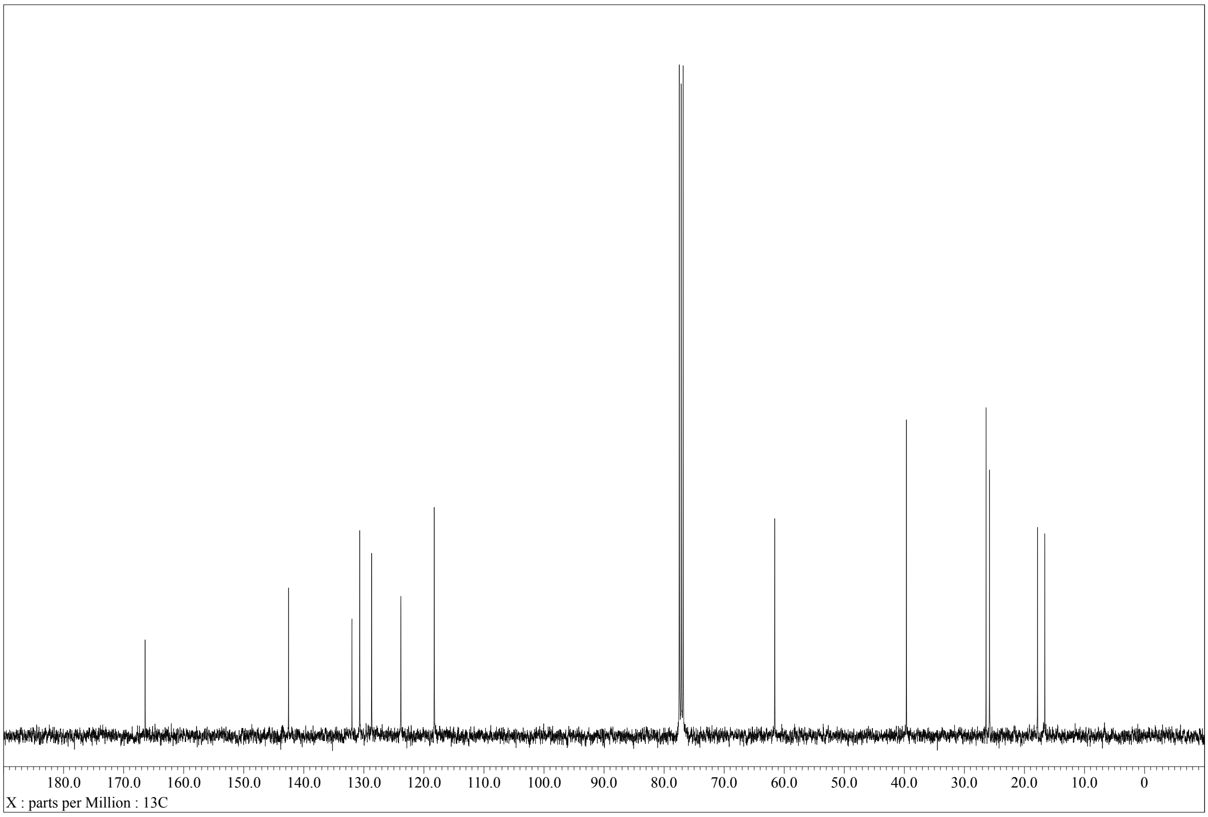

^{1}H and ^{13}C NMR spectra of (1*R*)-(–)-Myrtenyl acrylate (2f)

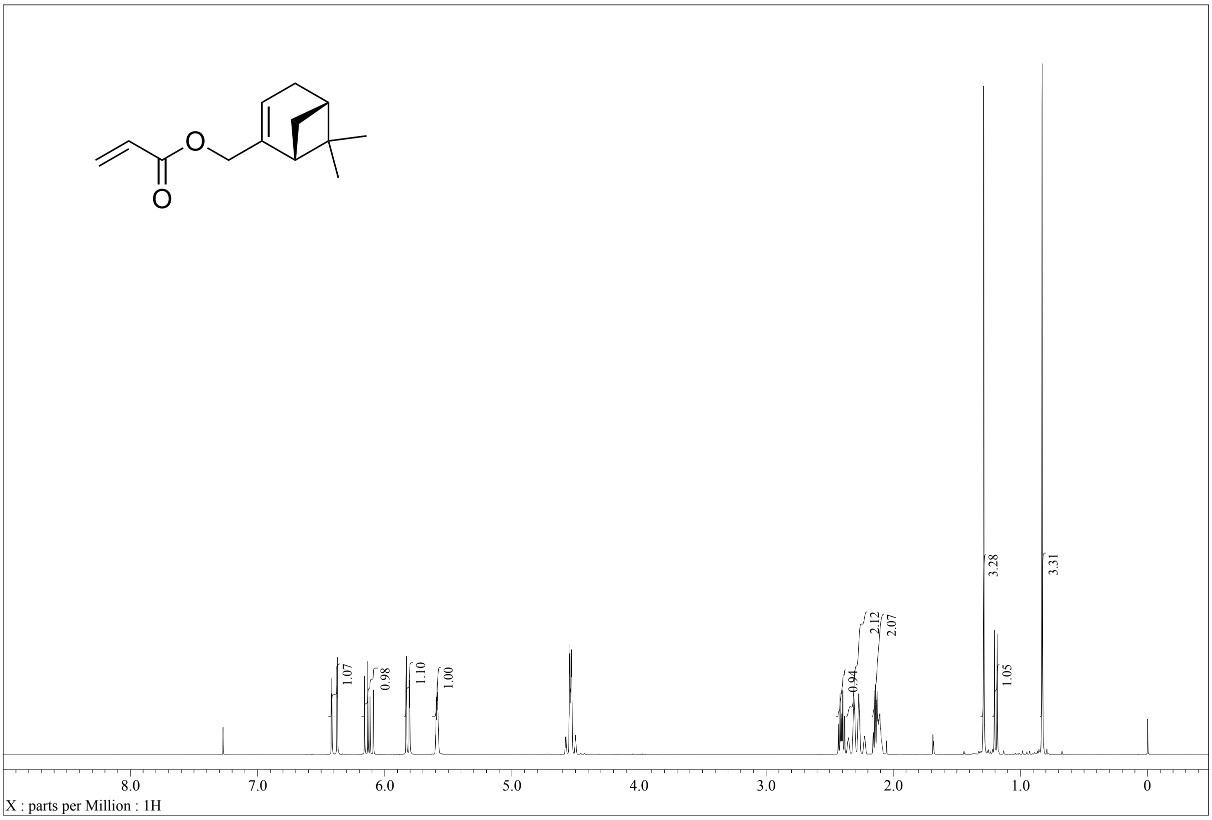

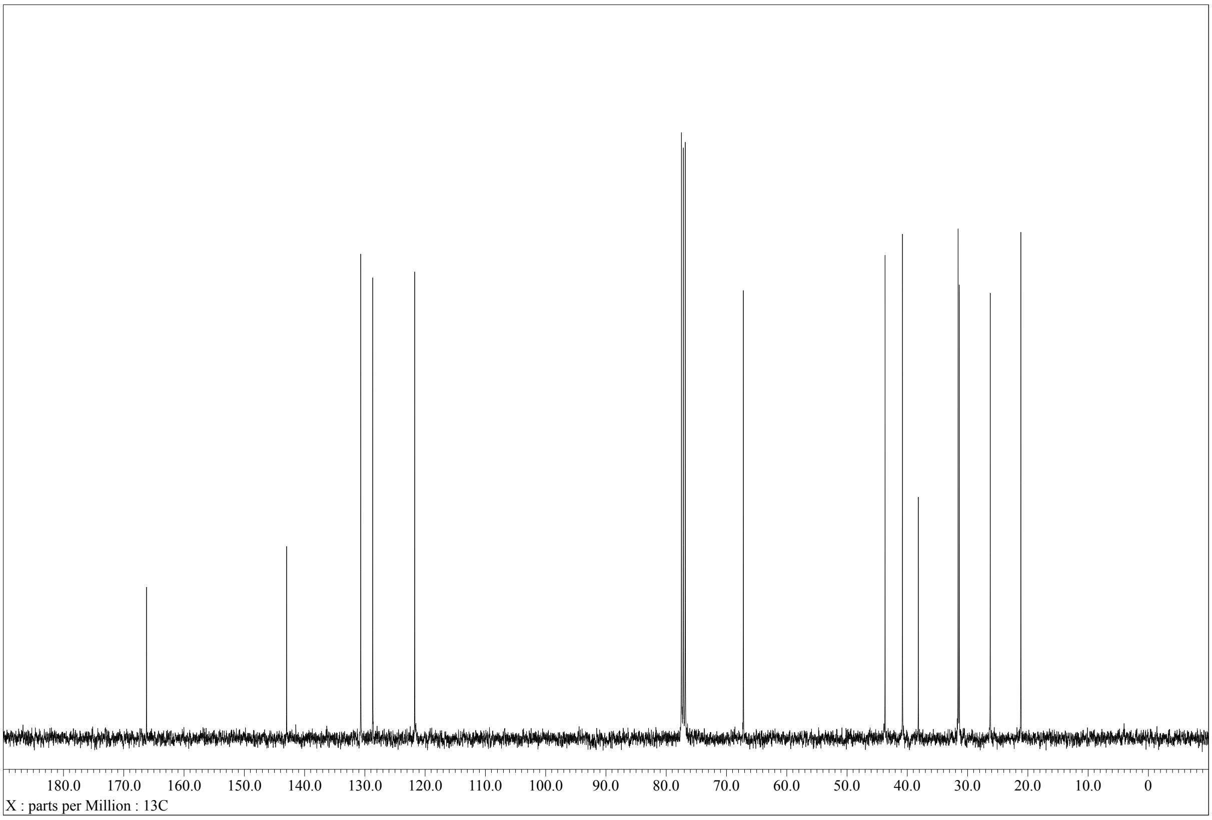

^{1}H and ^{13}C NMR spectra of L-Menthyl acrylate (2g)

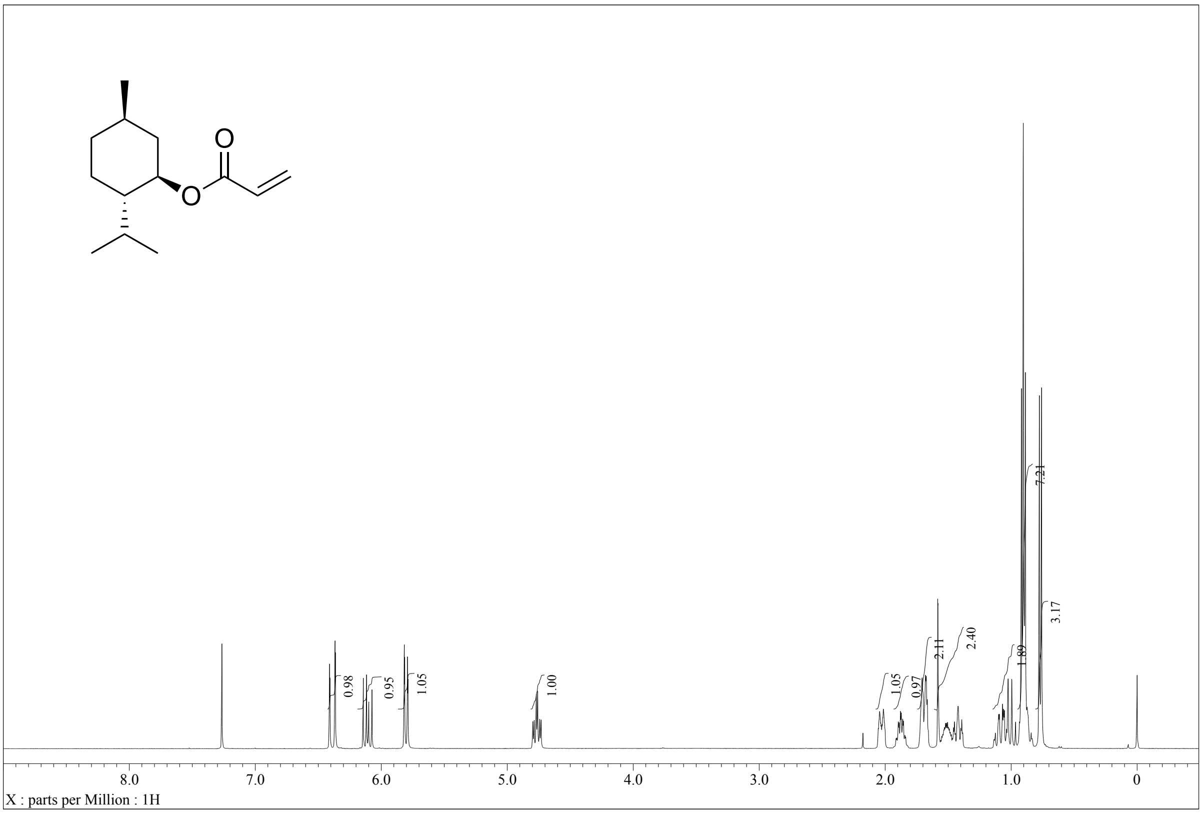

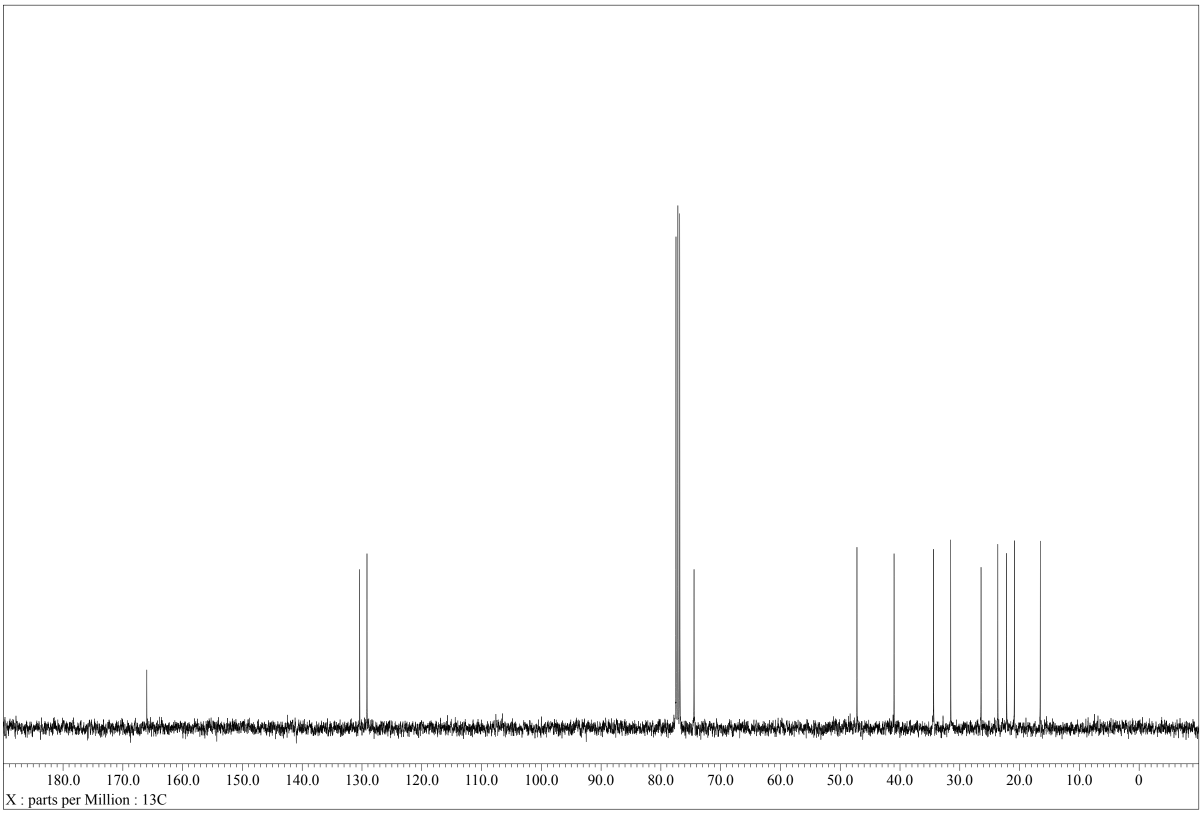

^{1}H and ^{13}C NMR spectra of Isobornyl acrylate (2b)

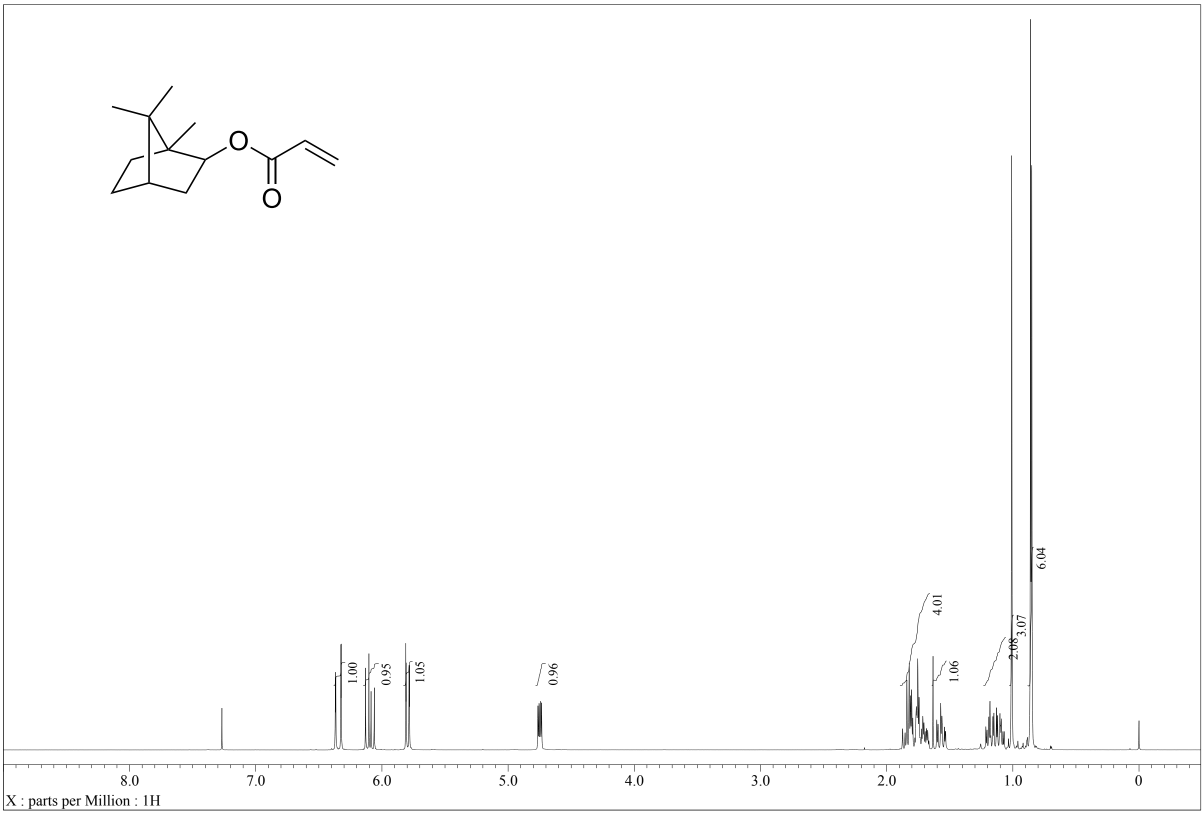

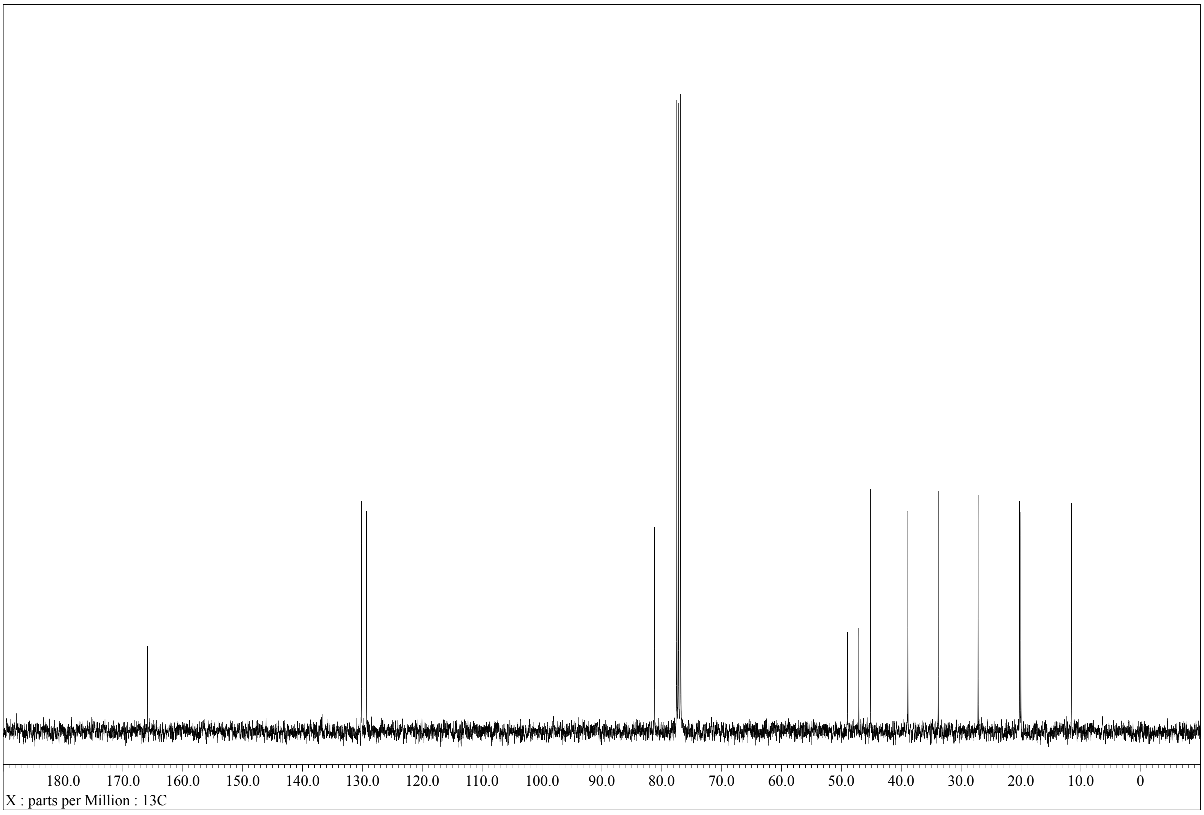

^{1}H and ^{13}C NMR spectra of Nonan-5-yl acrylate (2h)

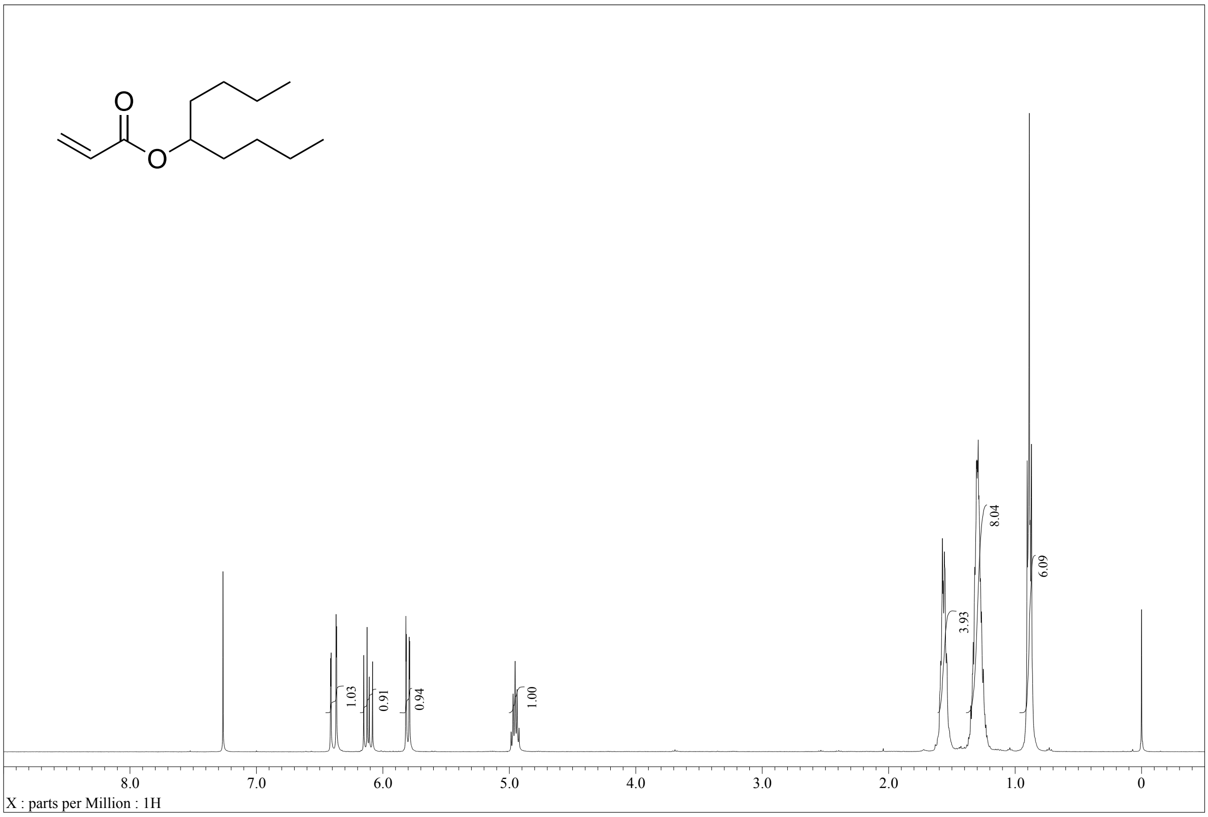

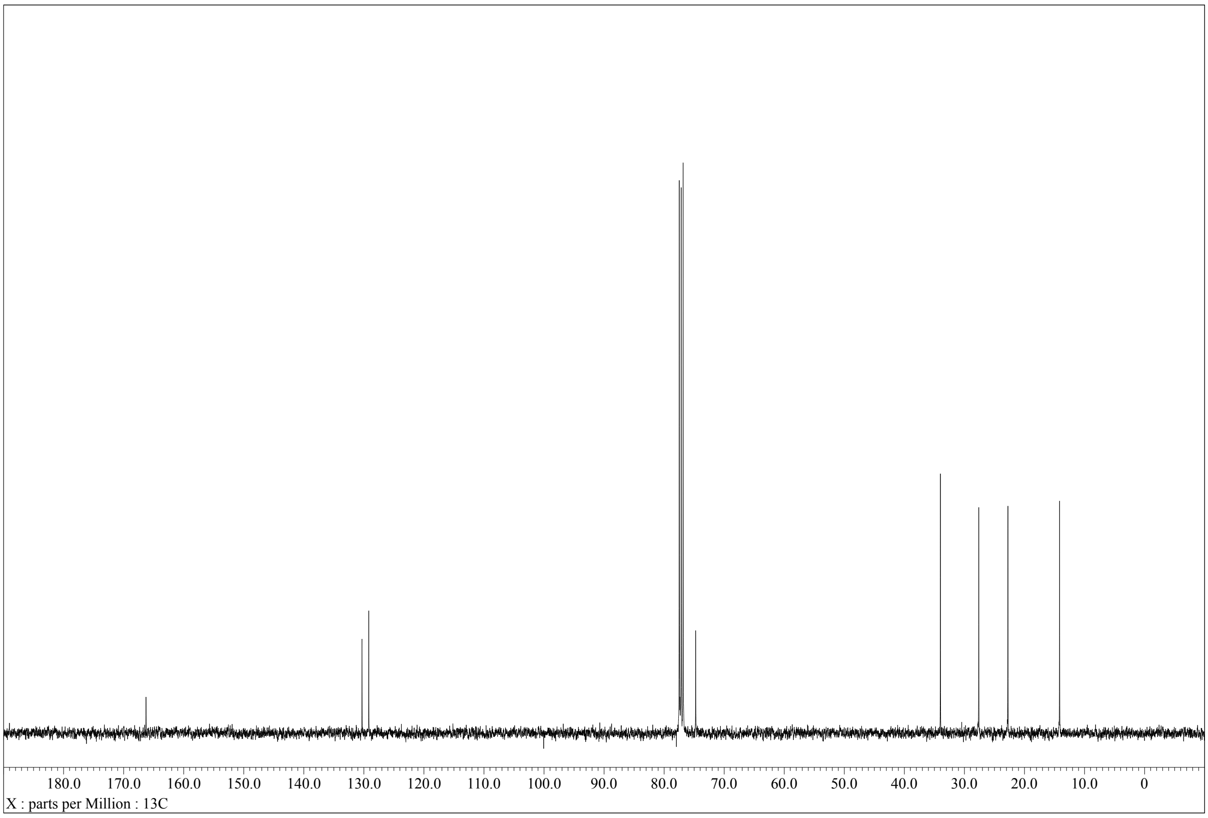

^{1}H and ^{13}C NMR spectra of Butane-1,4-diyl diacrylate (2i)

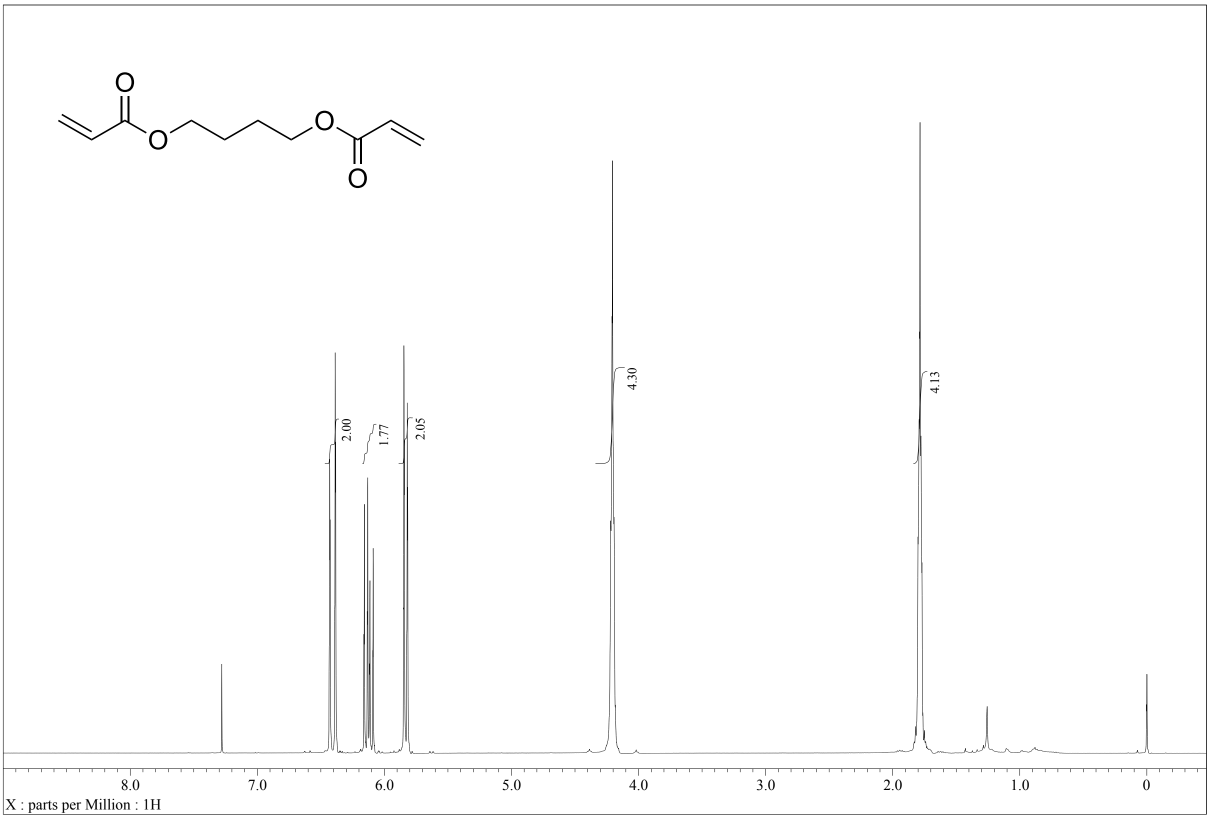

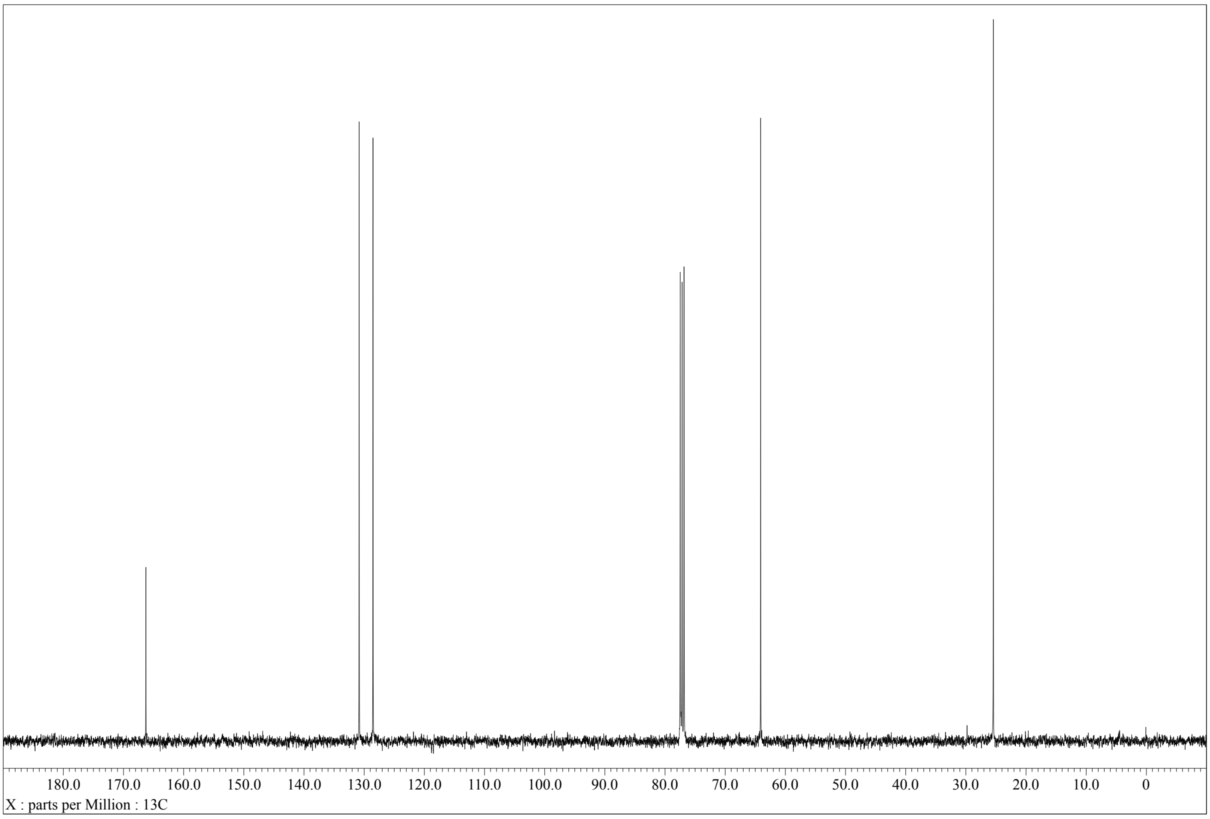

^{1}H and ^{13}C NMR spectra of hexane-1,6-diyl diacrylate (2j)

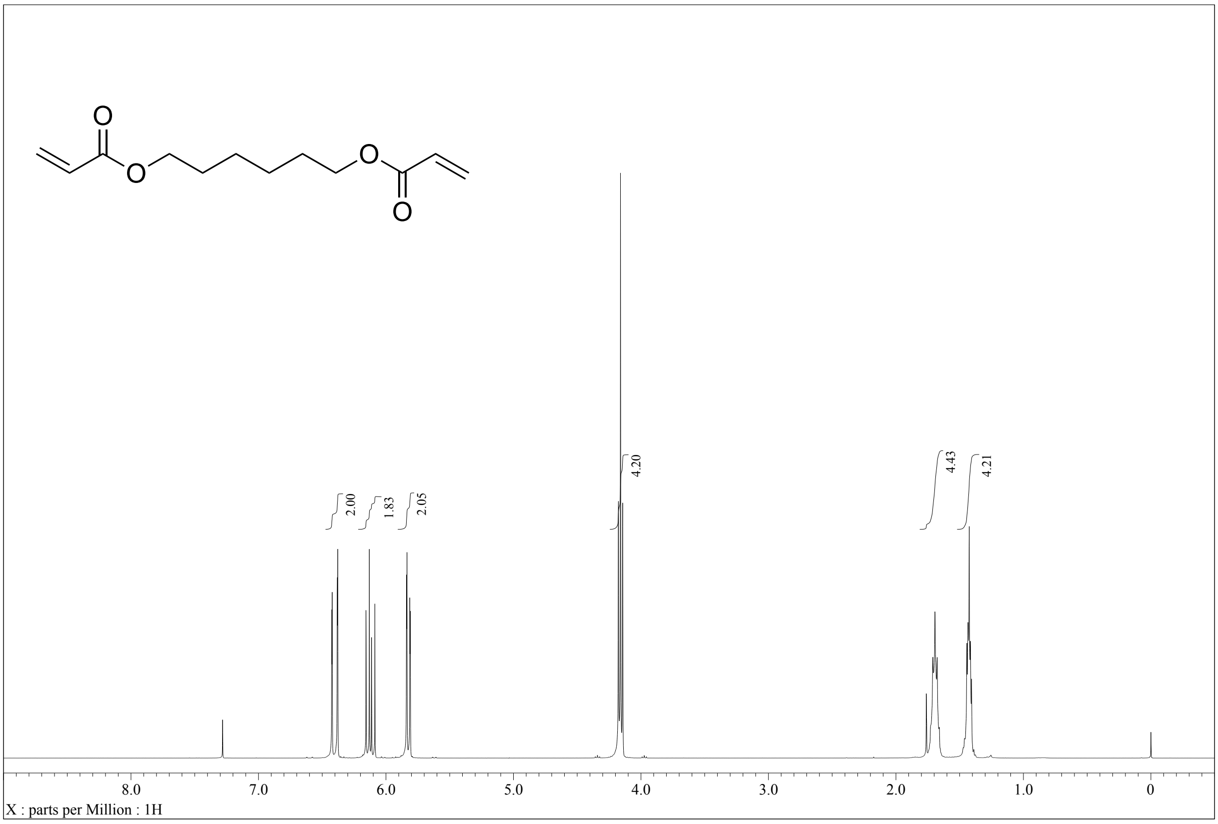

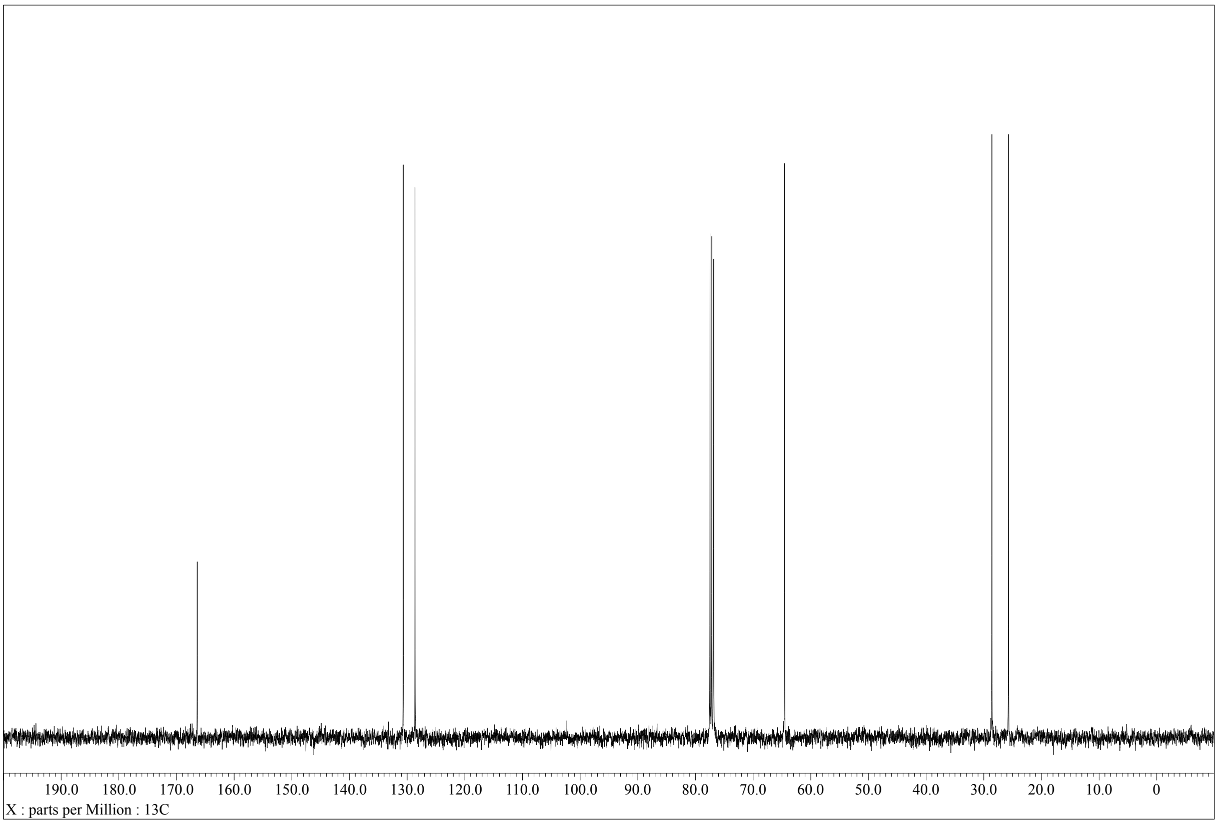

^{1}H and ^{13}C NMR spectra of (Ethane-1,2-diylbis(oxy))bis(ethane-2,1-diyl) diacrylate (2k)

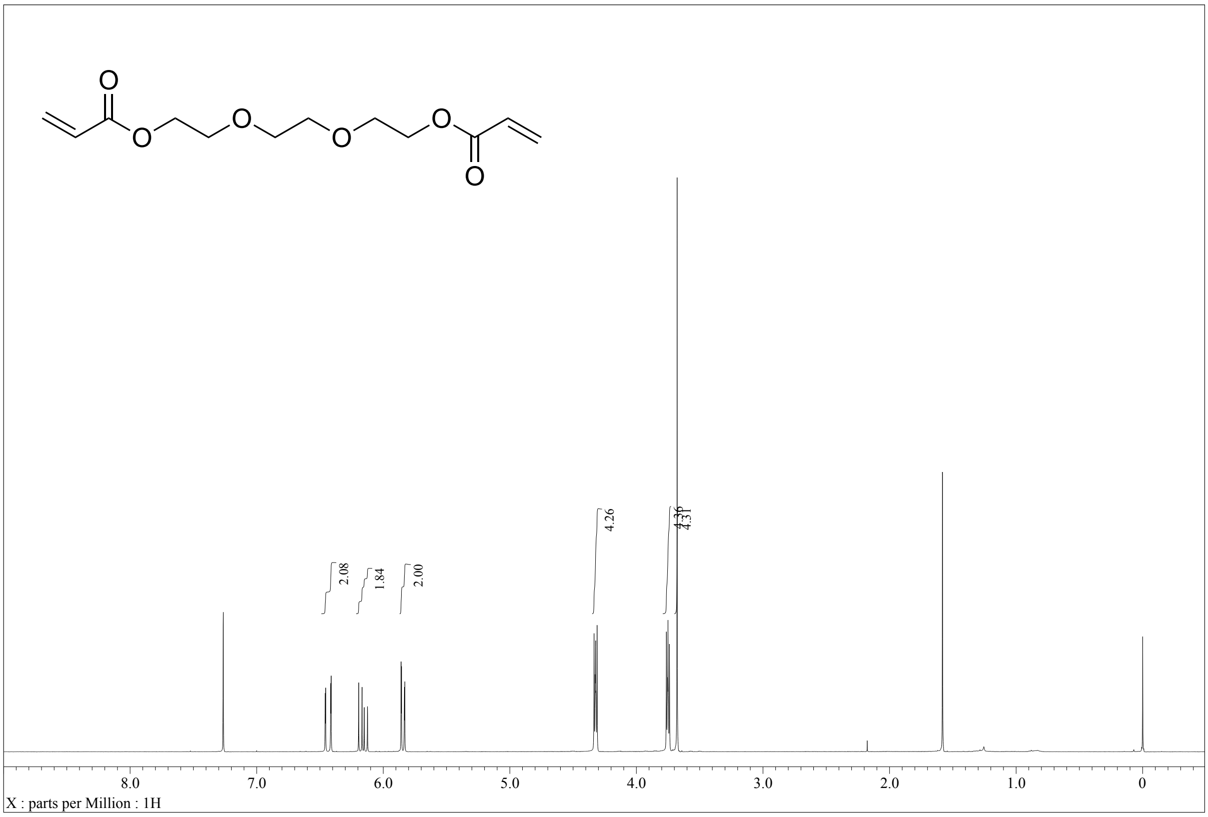

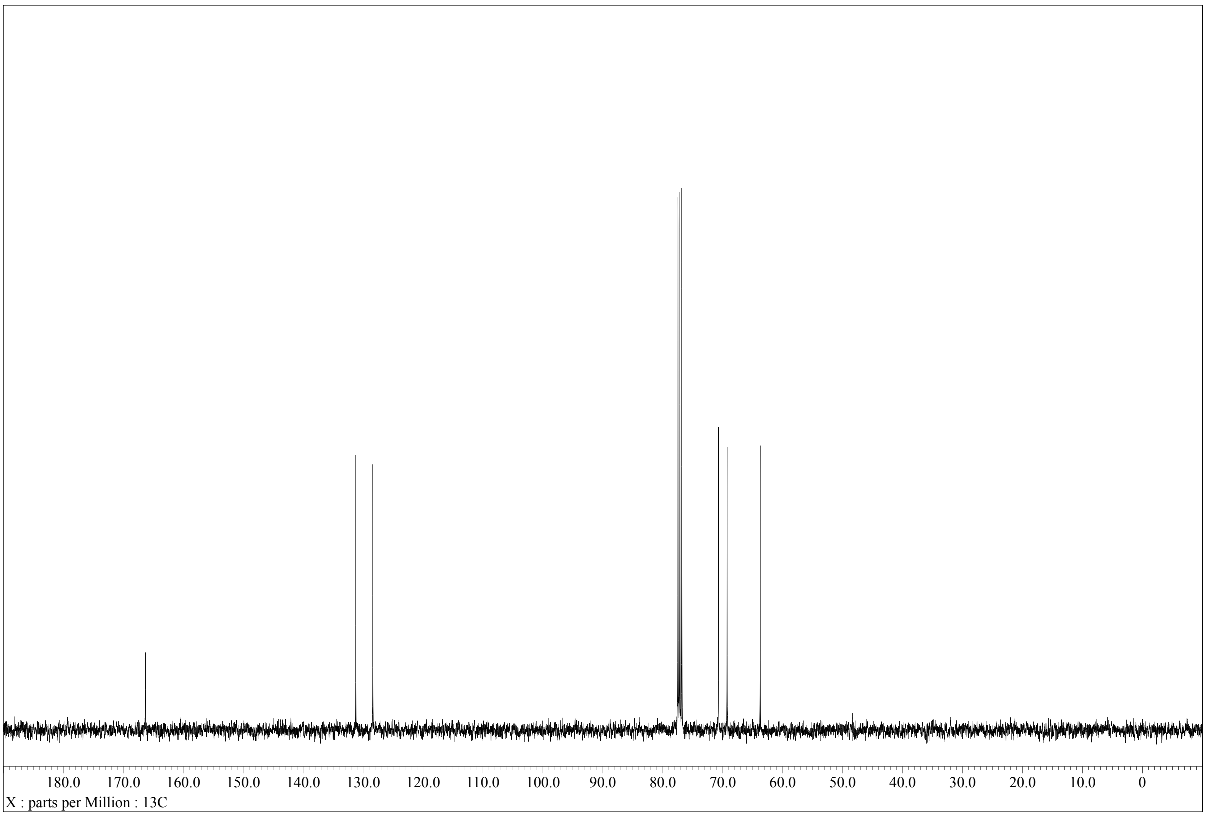

^{1}H and ^{13}C NMR spectra of 2-((Acryloyloxy)methyl)-2-ethylpropane-1,3-diyl diacrylate (2l)

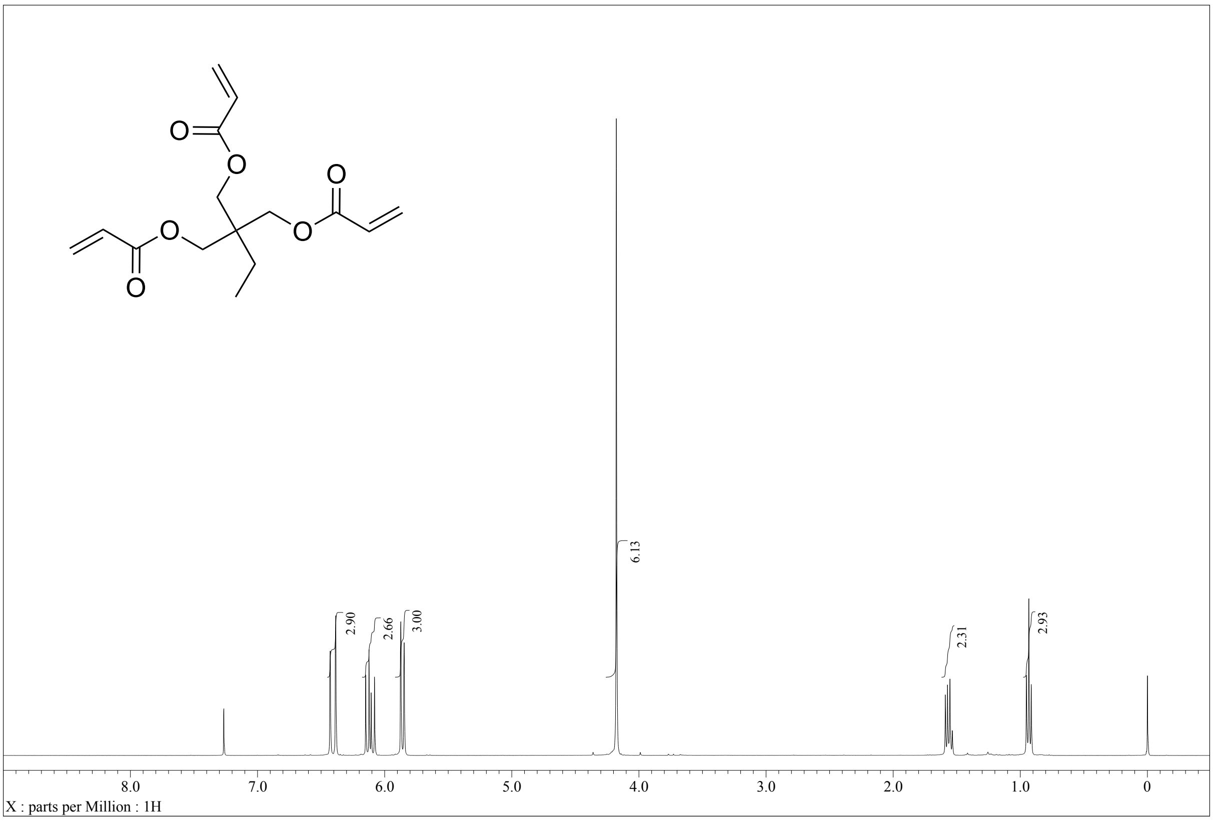

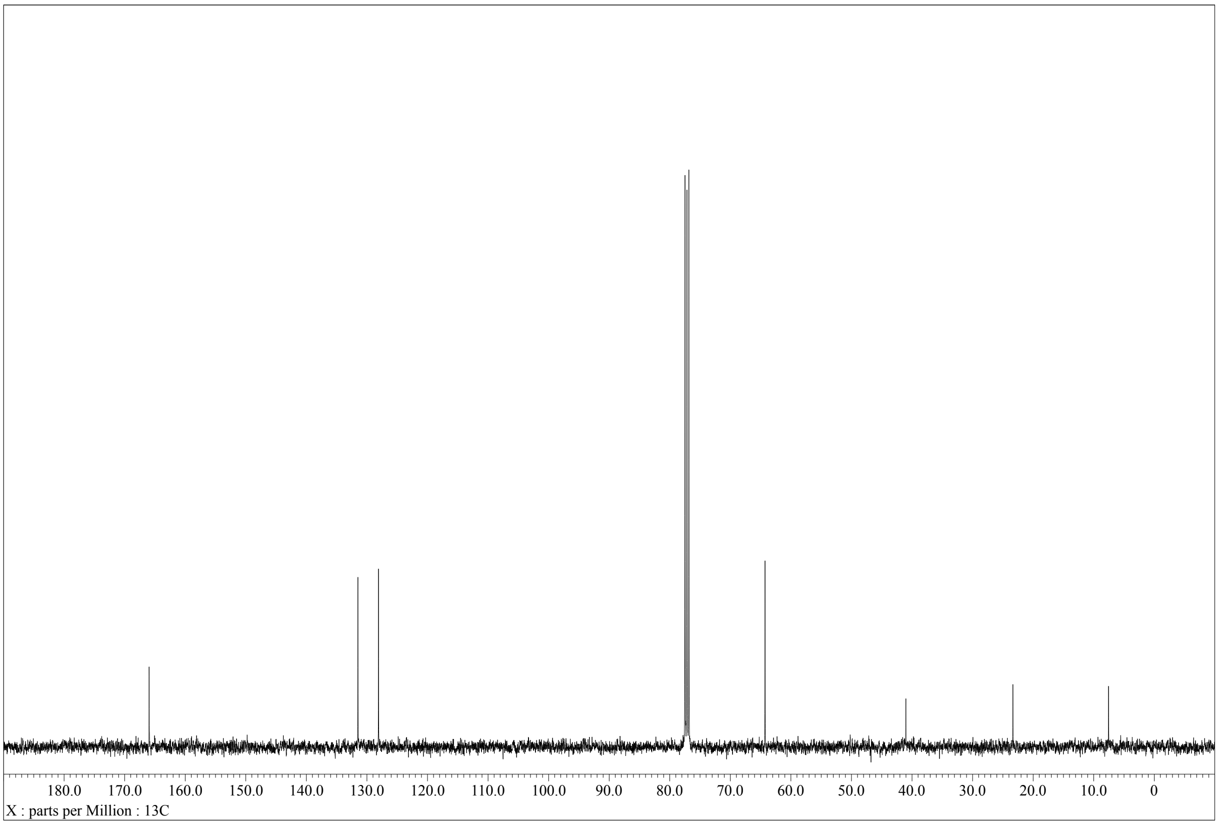

^{1}H and ^{13}C NMR spectra of Dodecyl methacrylate (7c)

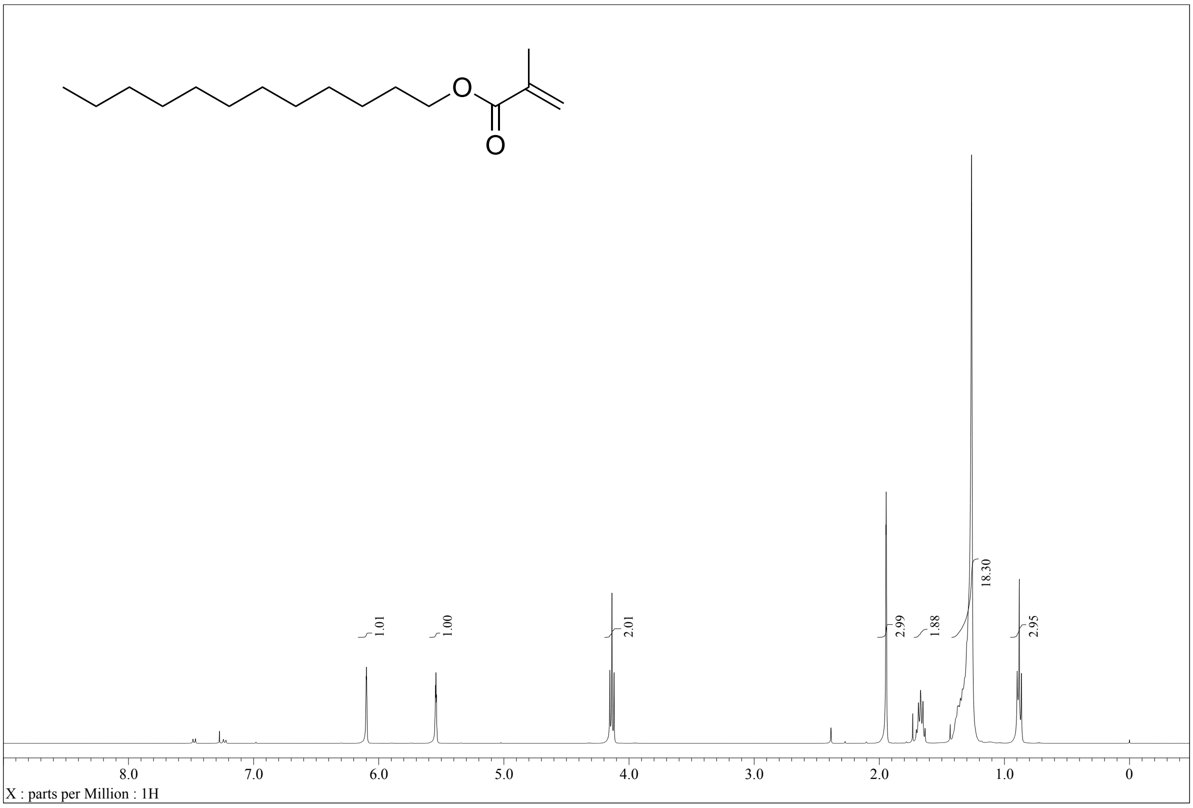

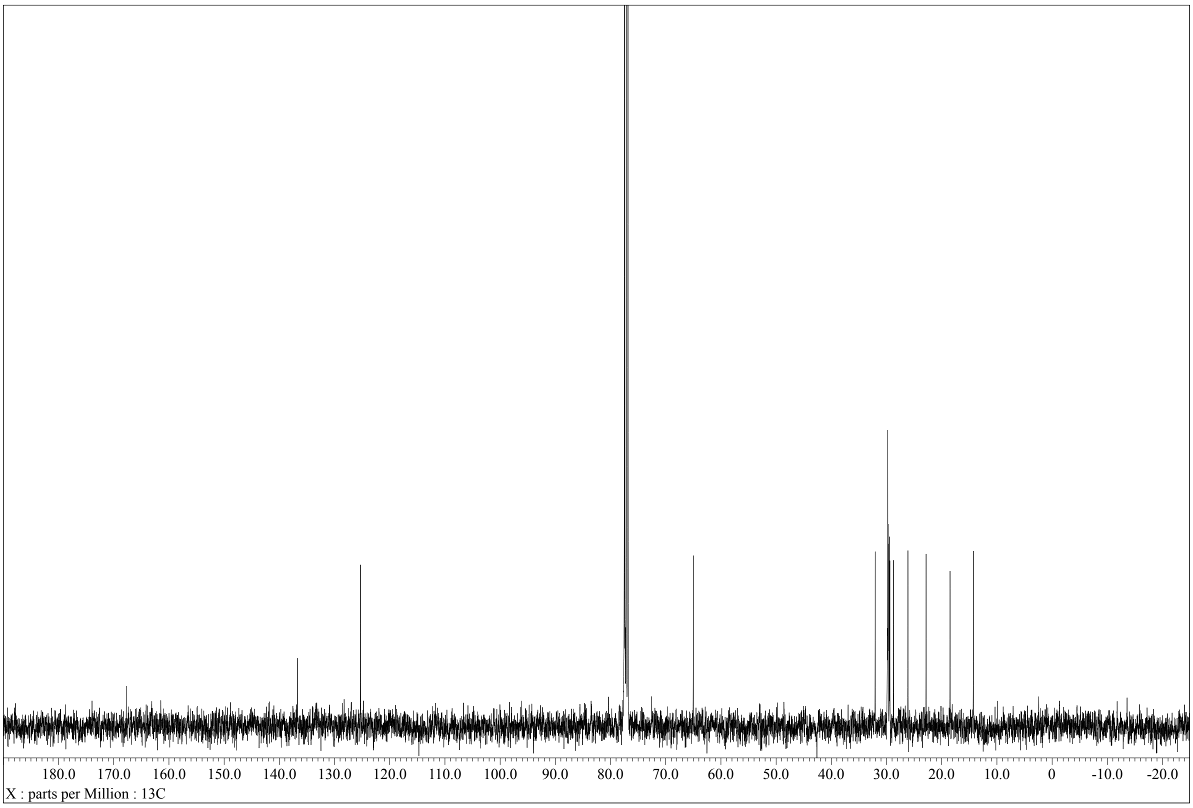

^{1}H and ^{13}C NMR spectra of Geranyl methacrylate (7e)

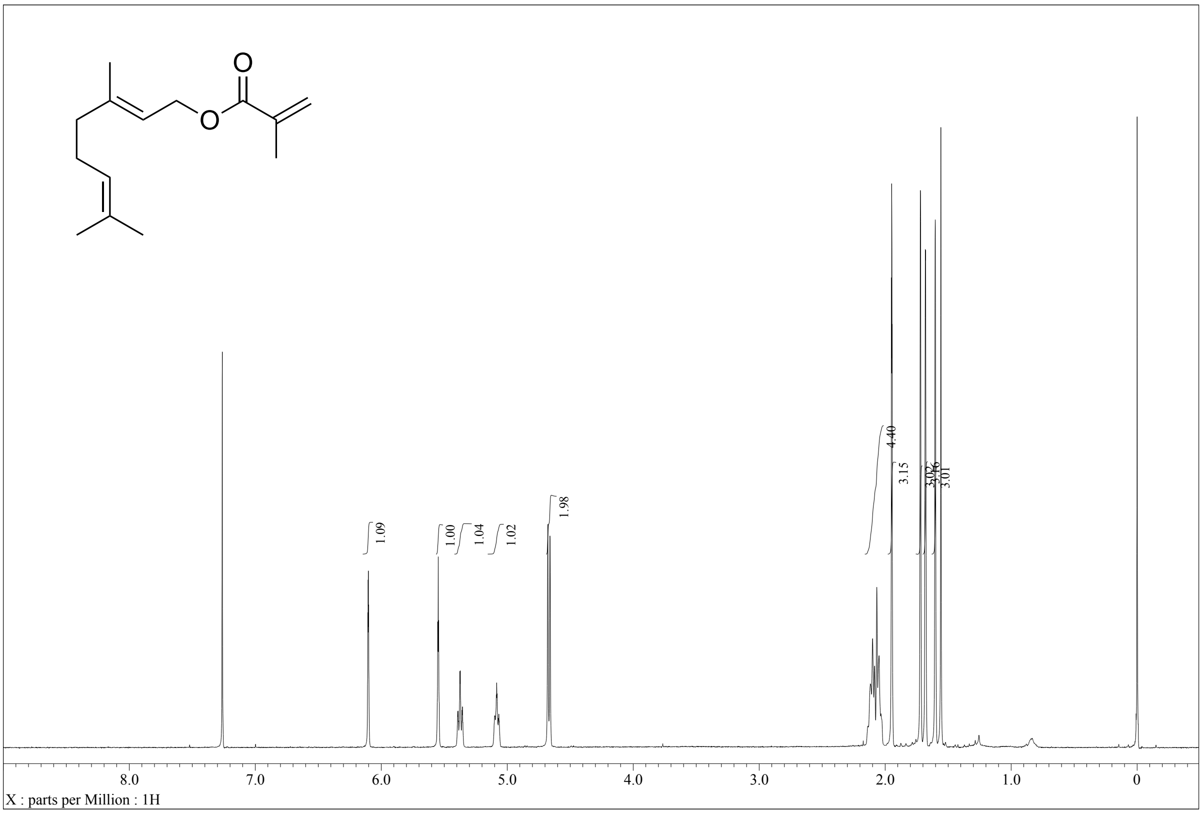

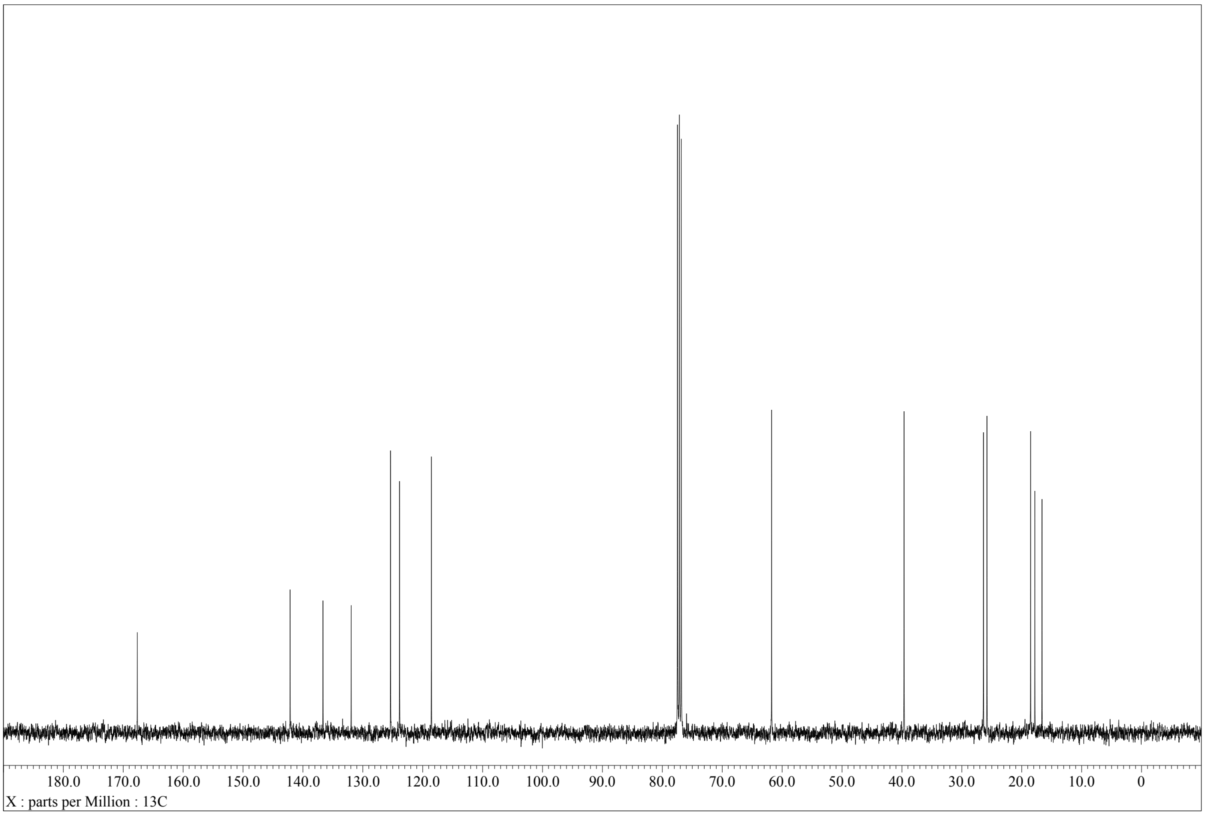

^{1}H and ^{13}C NMR spectra of Benzyl methacrylate (7a):

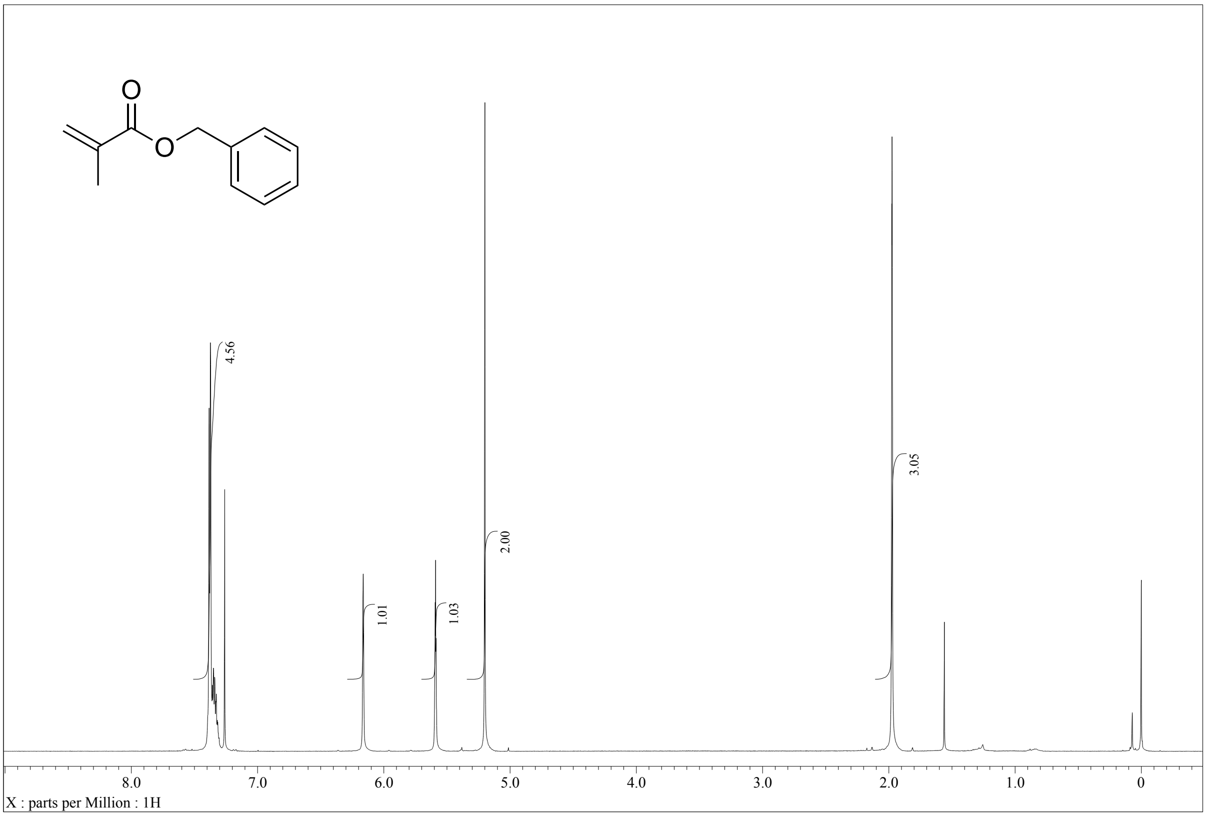

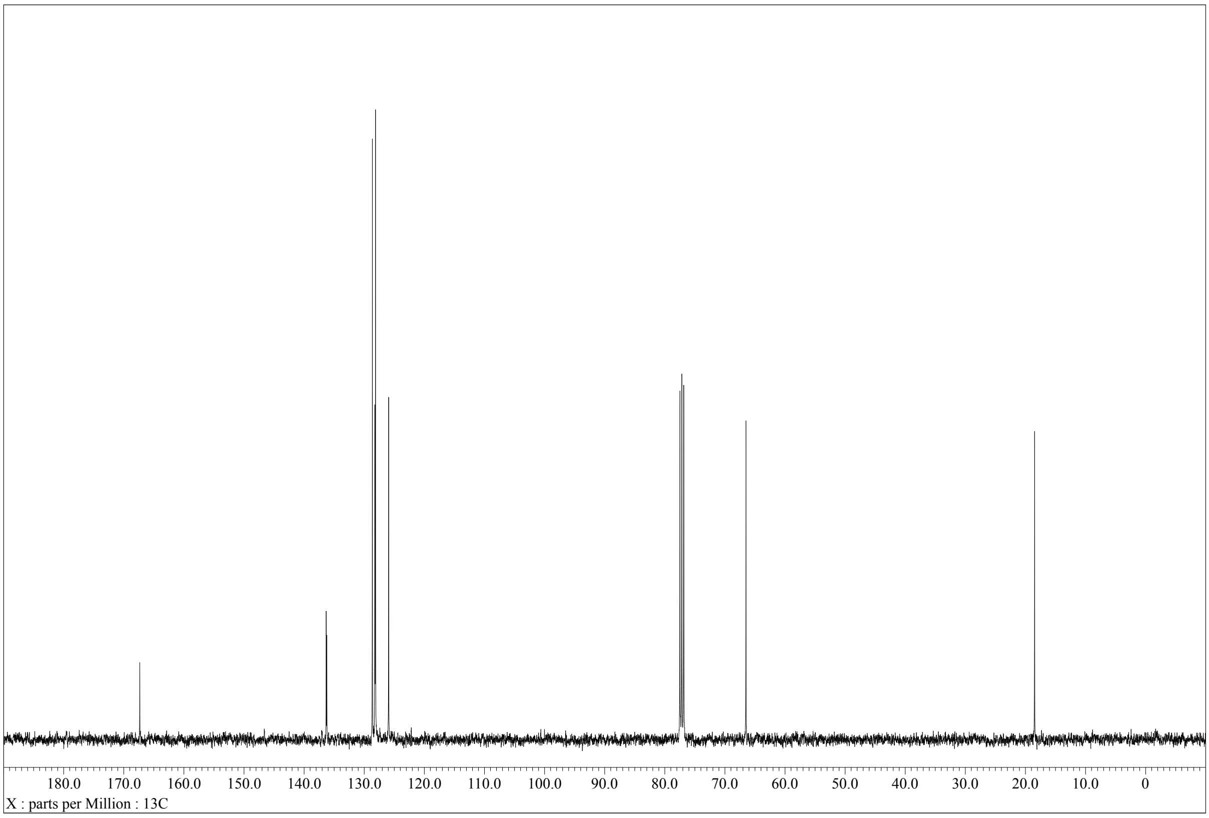

^{1}H and ^{13}C NMR spectra of Oxiran-2-ylmethyl methacrylate (7m):

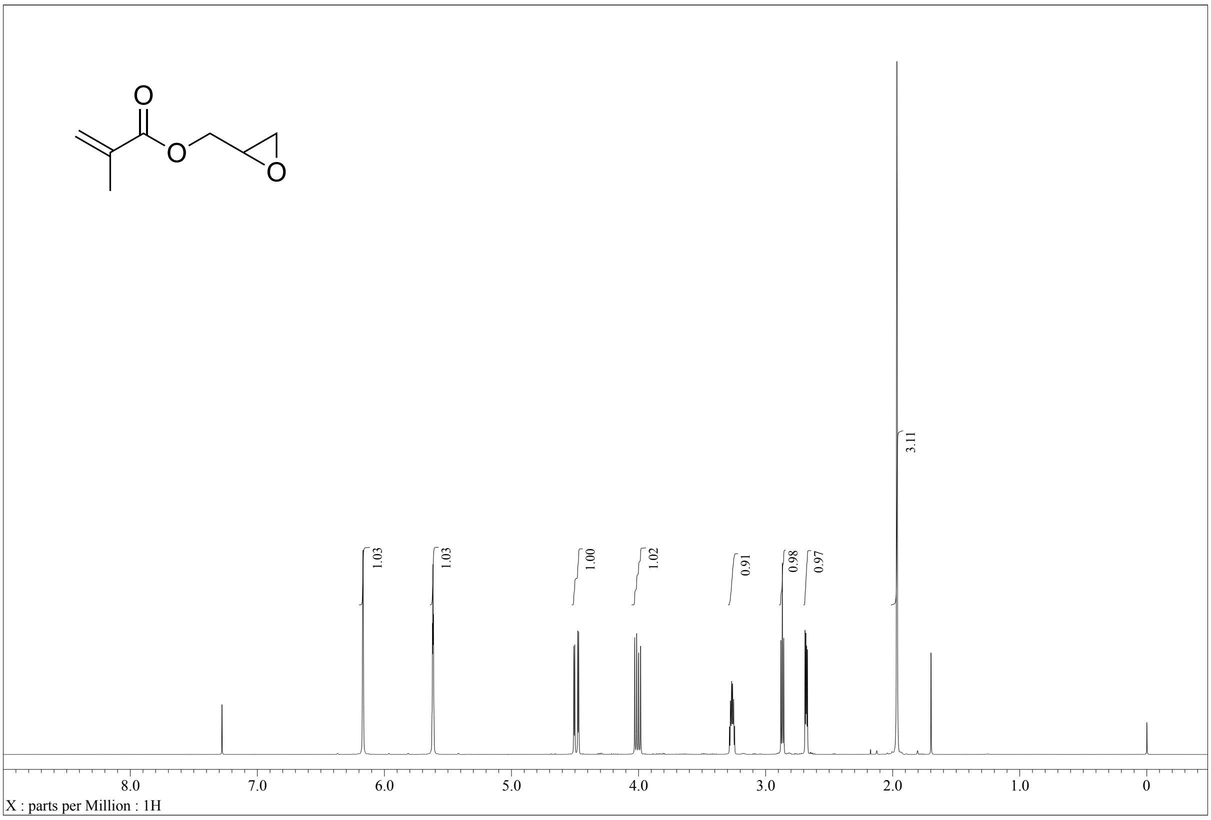

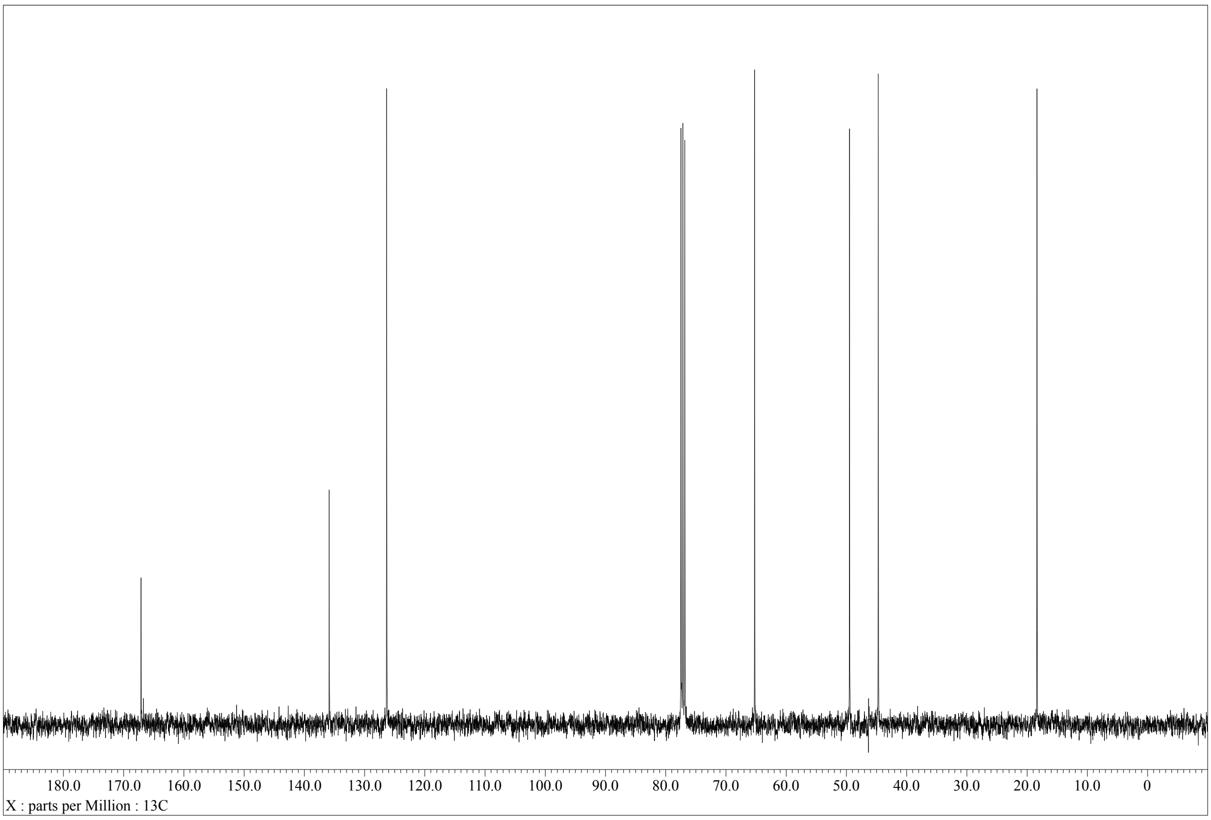

^{1}H,^{13}C and ^{19}F NMR spectra of 2,2,2-Trifluoromethyl methacrylate (7n)

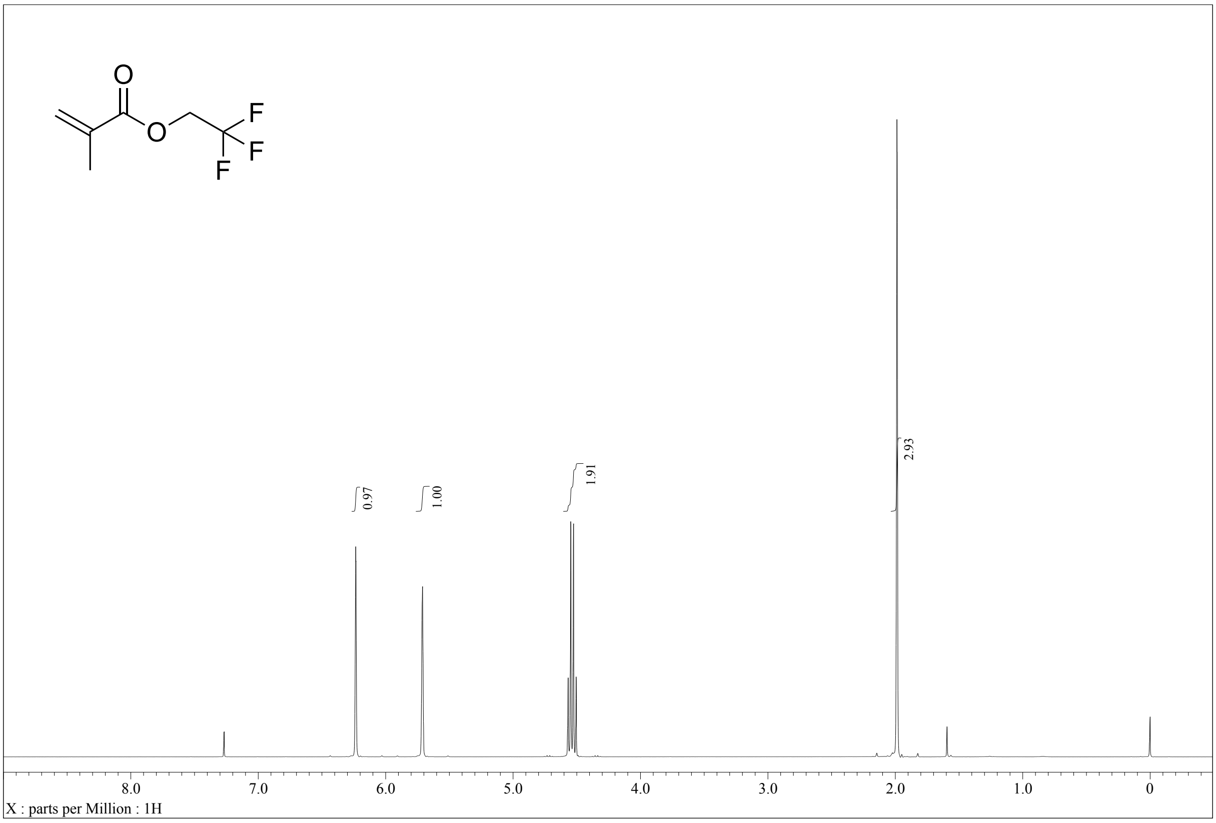

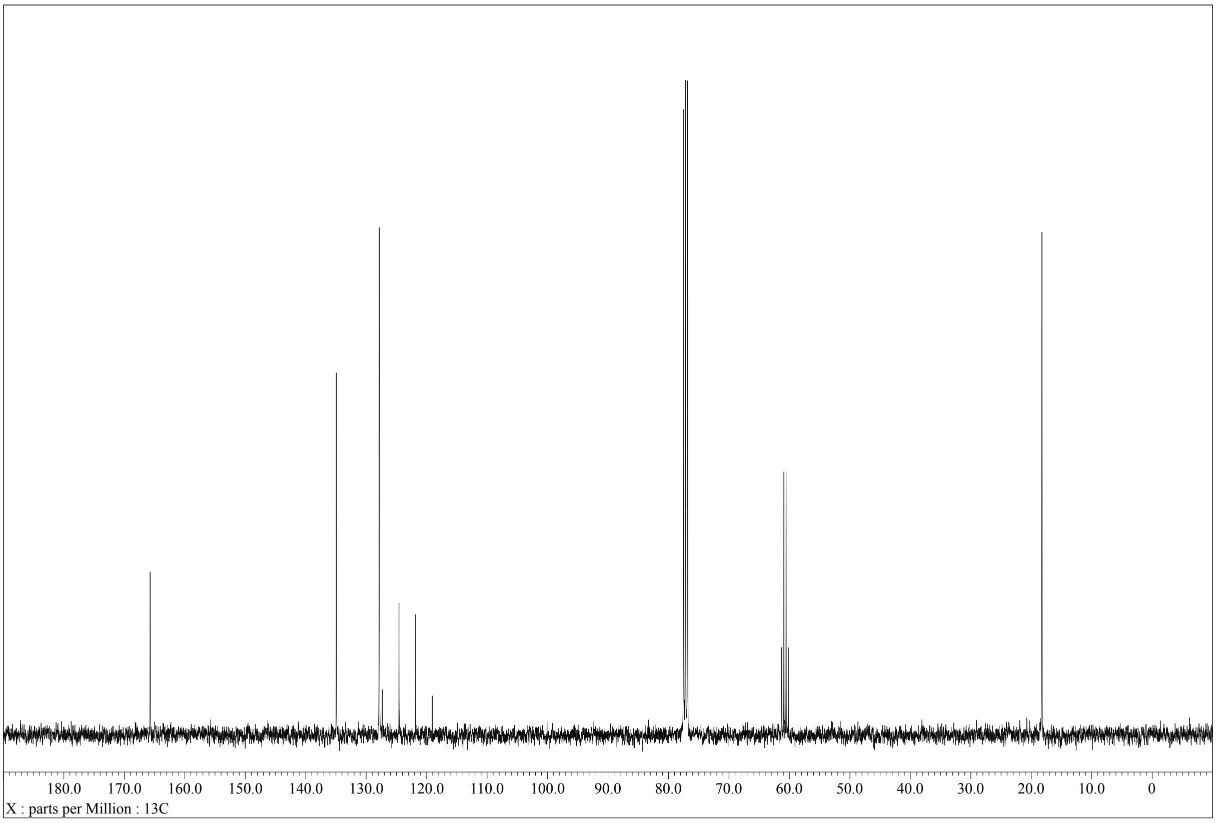

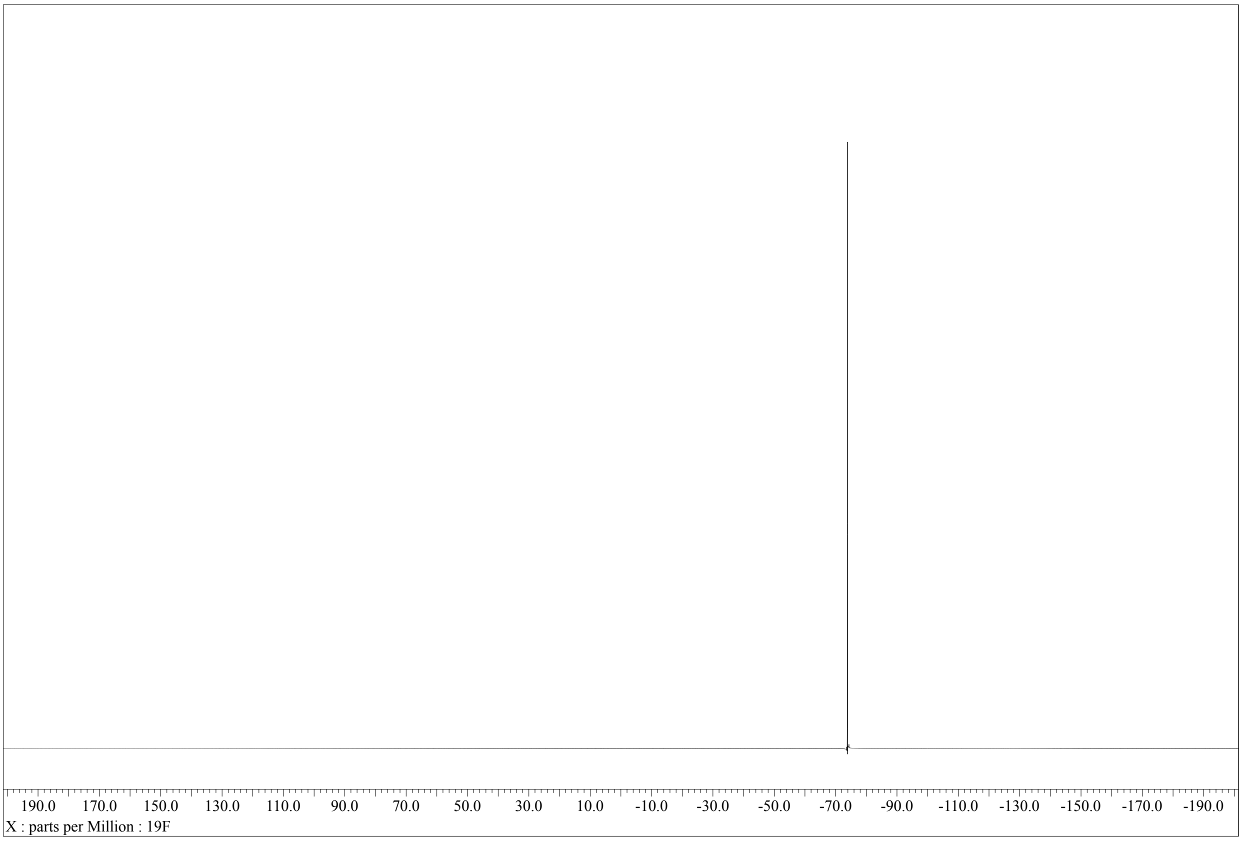
190.0 170.0 150.0 130.0 110.0 90.0 70.0 50.0 30.0 10.0 -10.0 -30.0 -50.0 -70.0 -90.0 -110.0 -130.0 -150.0 -170.0 -190.0
X : parts per Million : 19F

^{1}H and ^{13}C NMR spectra of Isobornyl methacrylate (7b)

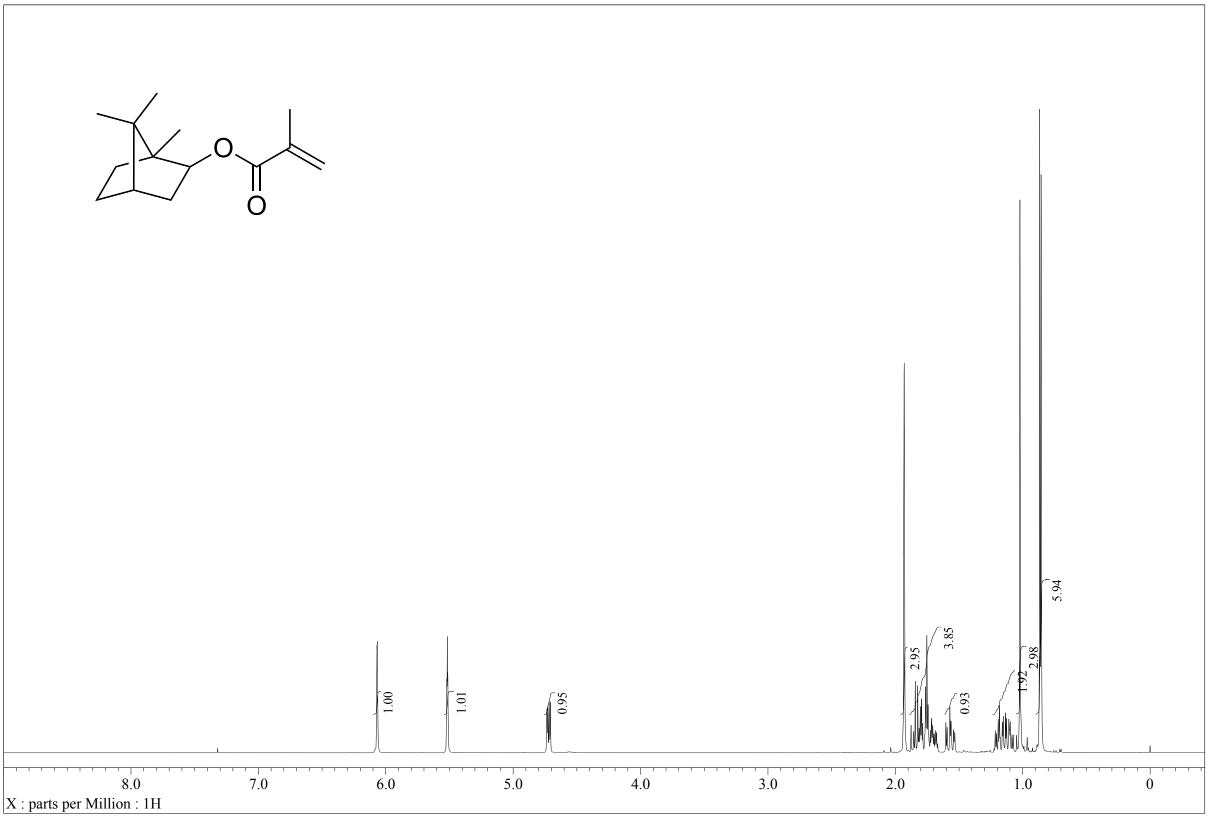

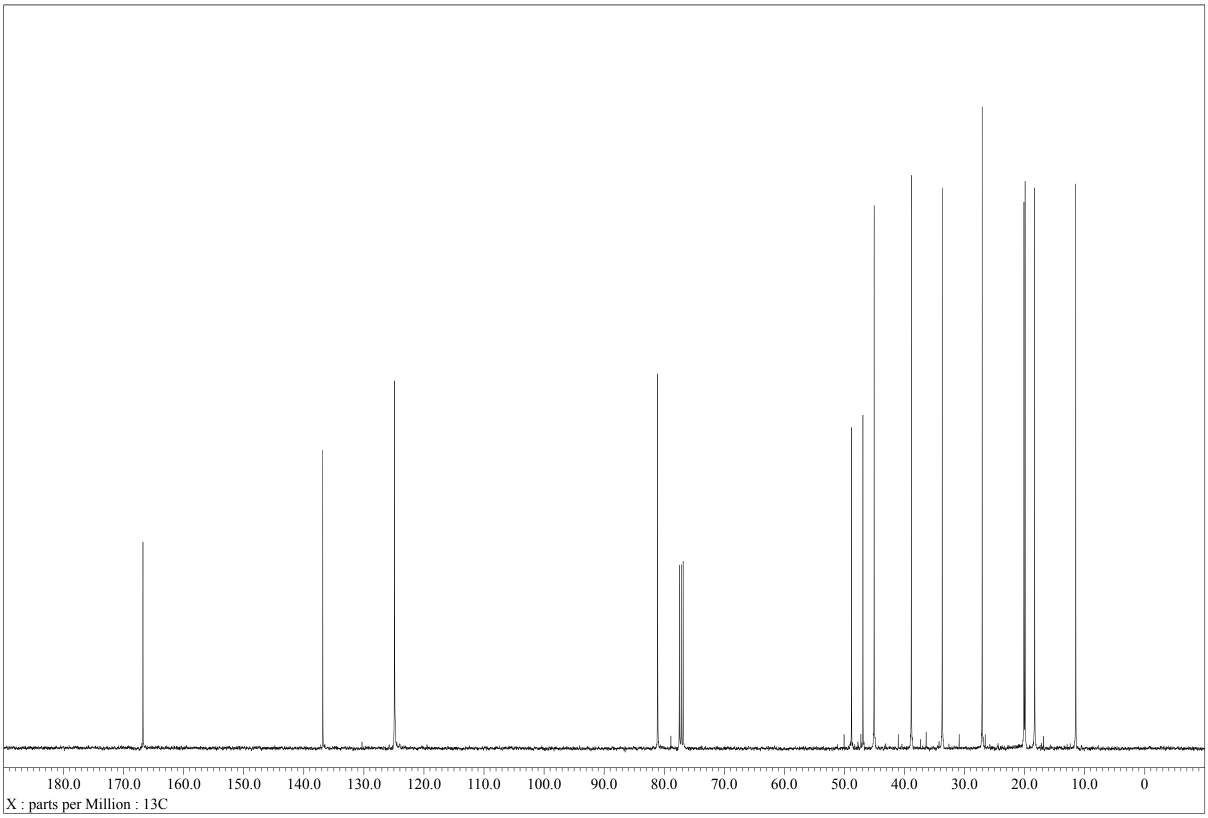

^{1}H and ^{13}C NMR spectra of L-Menthyl methacrylate (7g)

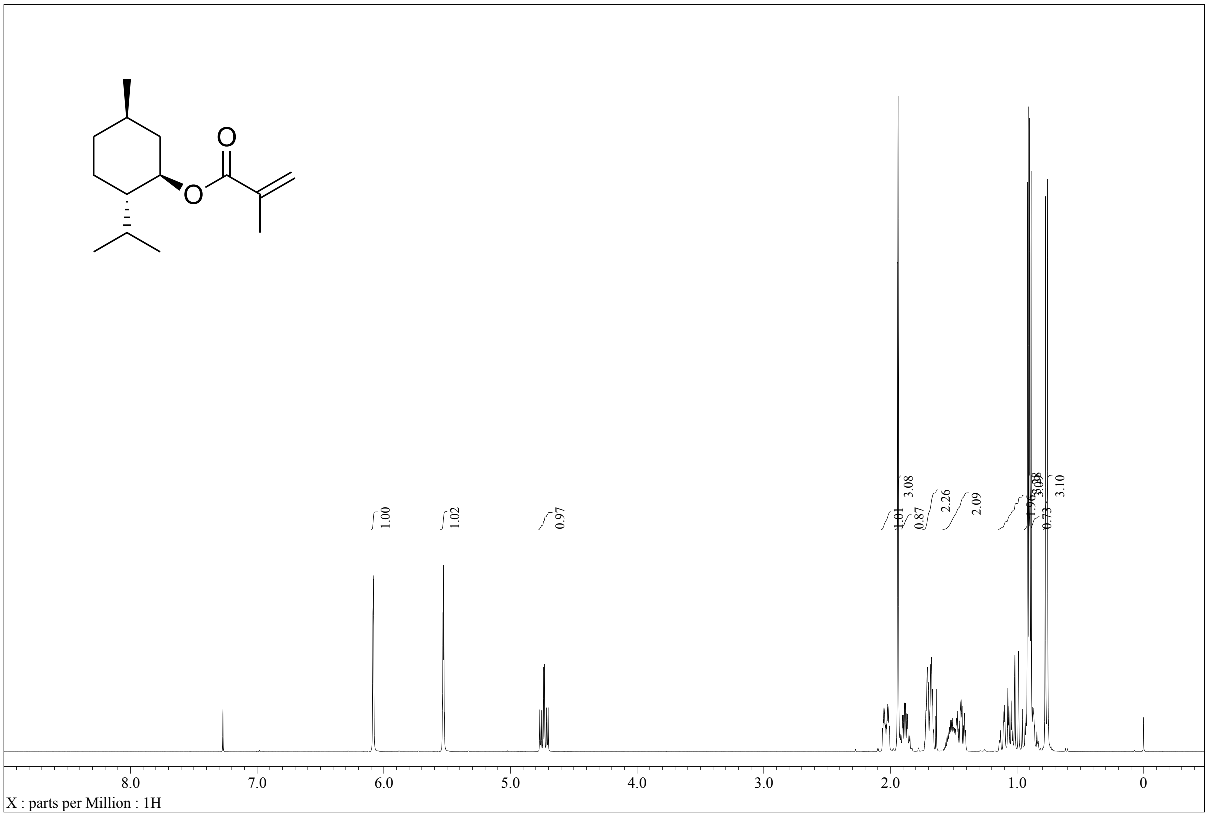

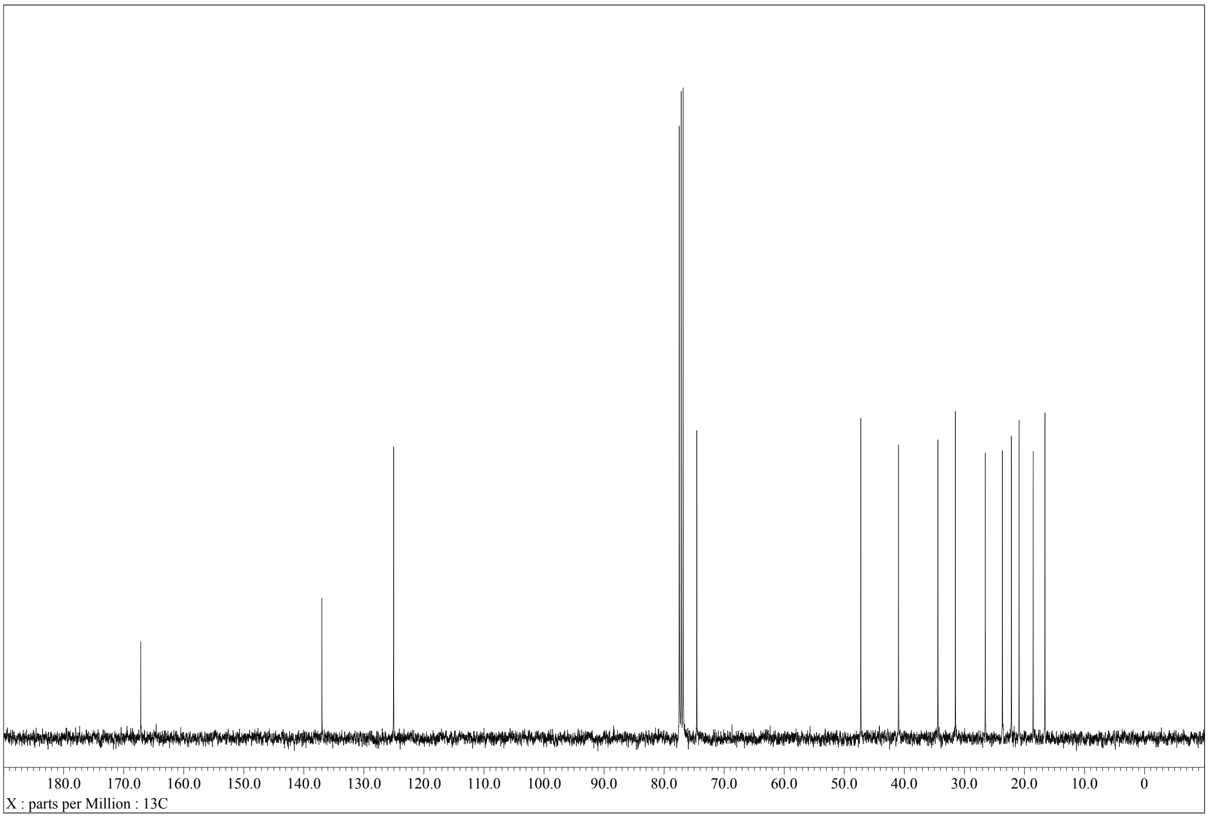

^{1}H and ^{13}C NMR spectra of Nonan-5-yl methacrylate 7h)

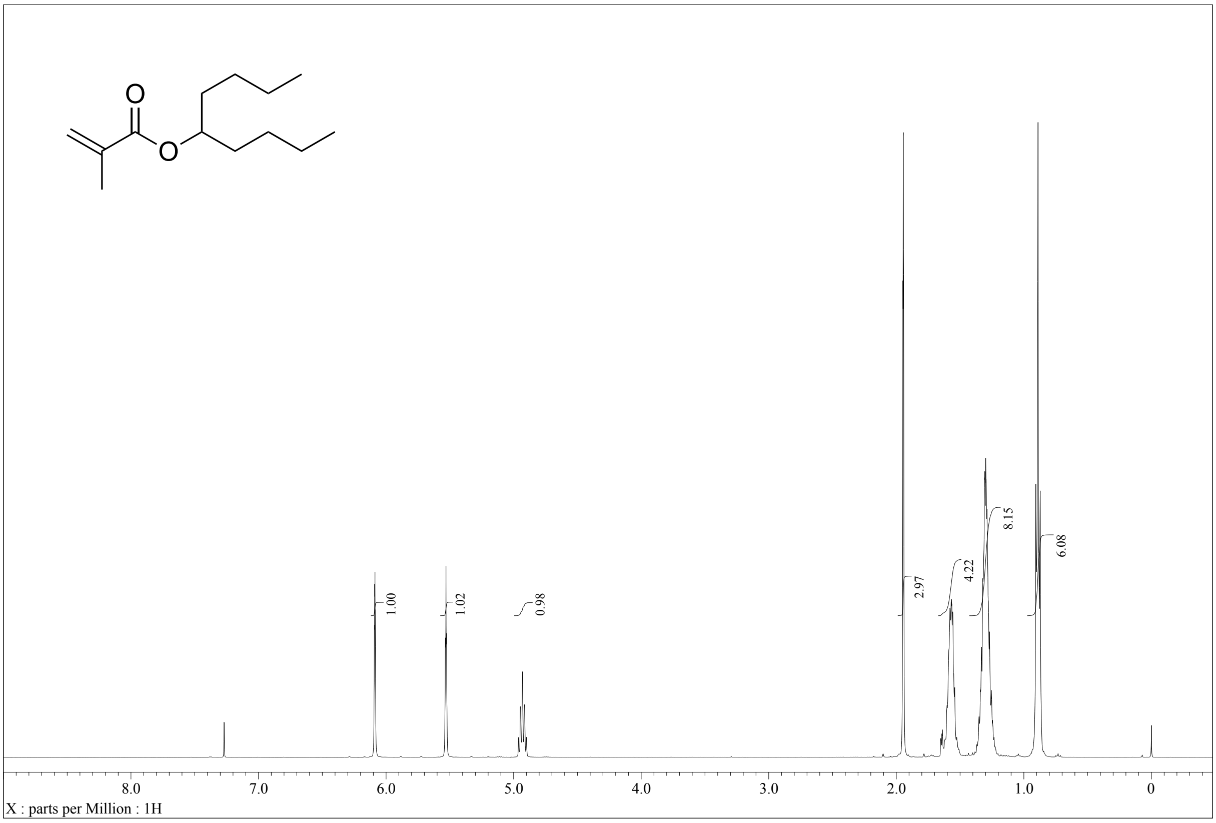

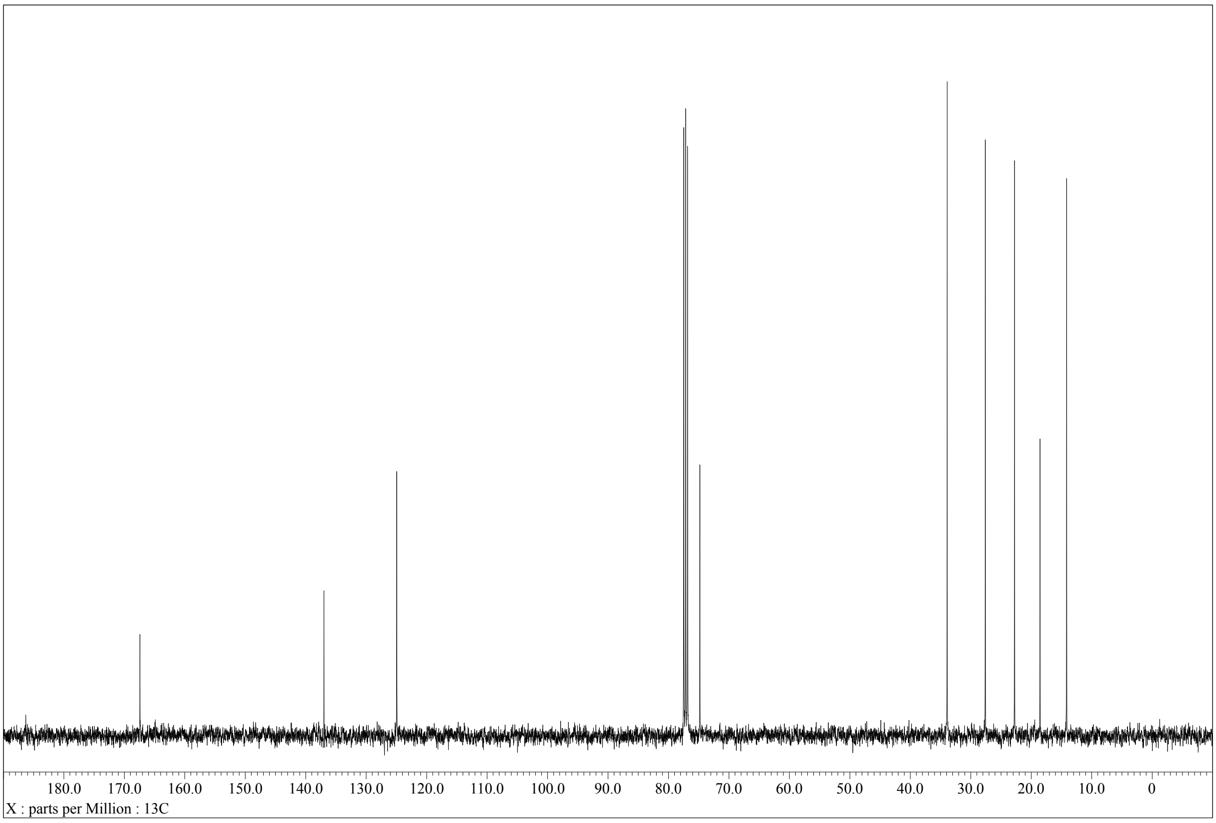

^{1}H and ^{13}C NMR spectra of Cholesteryl methacrylate (7o):

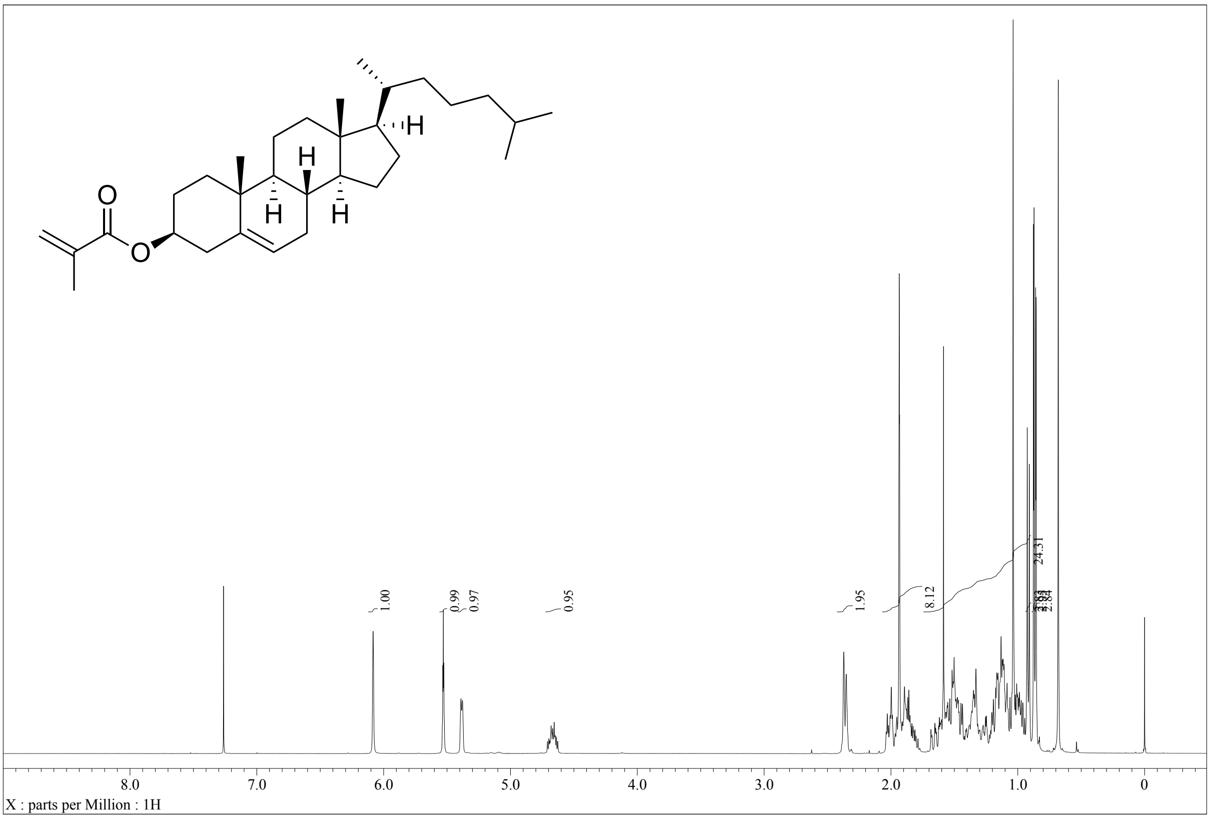

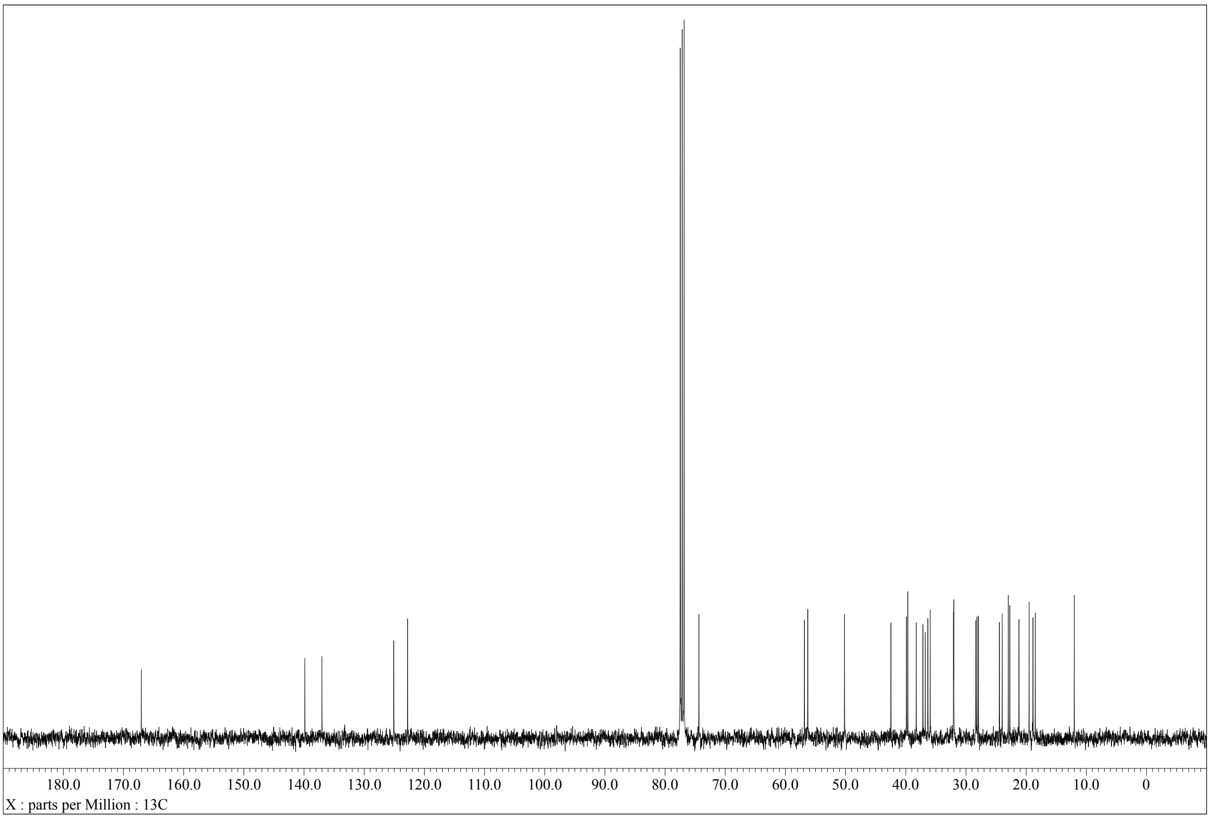

^{1}H and ^{13}C NMR spectra of Quinine methacrylate (7p)

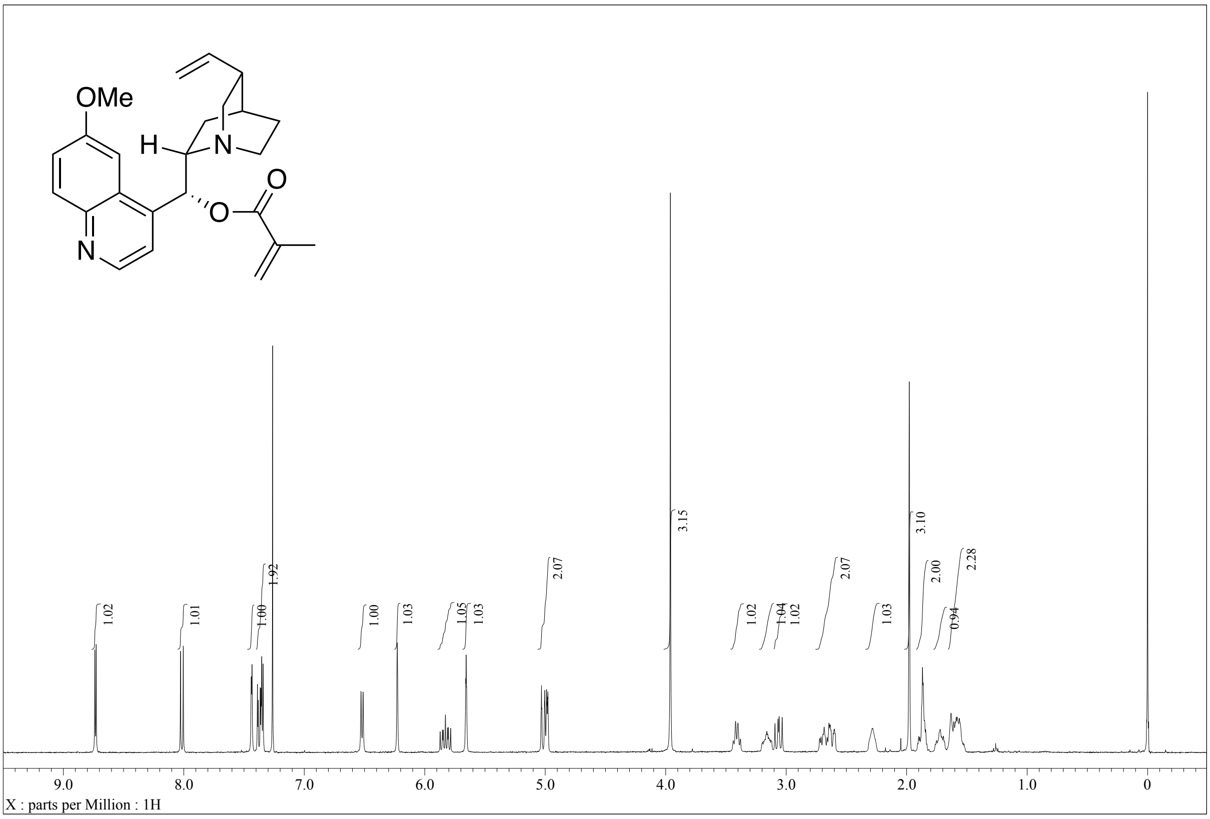

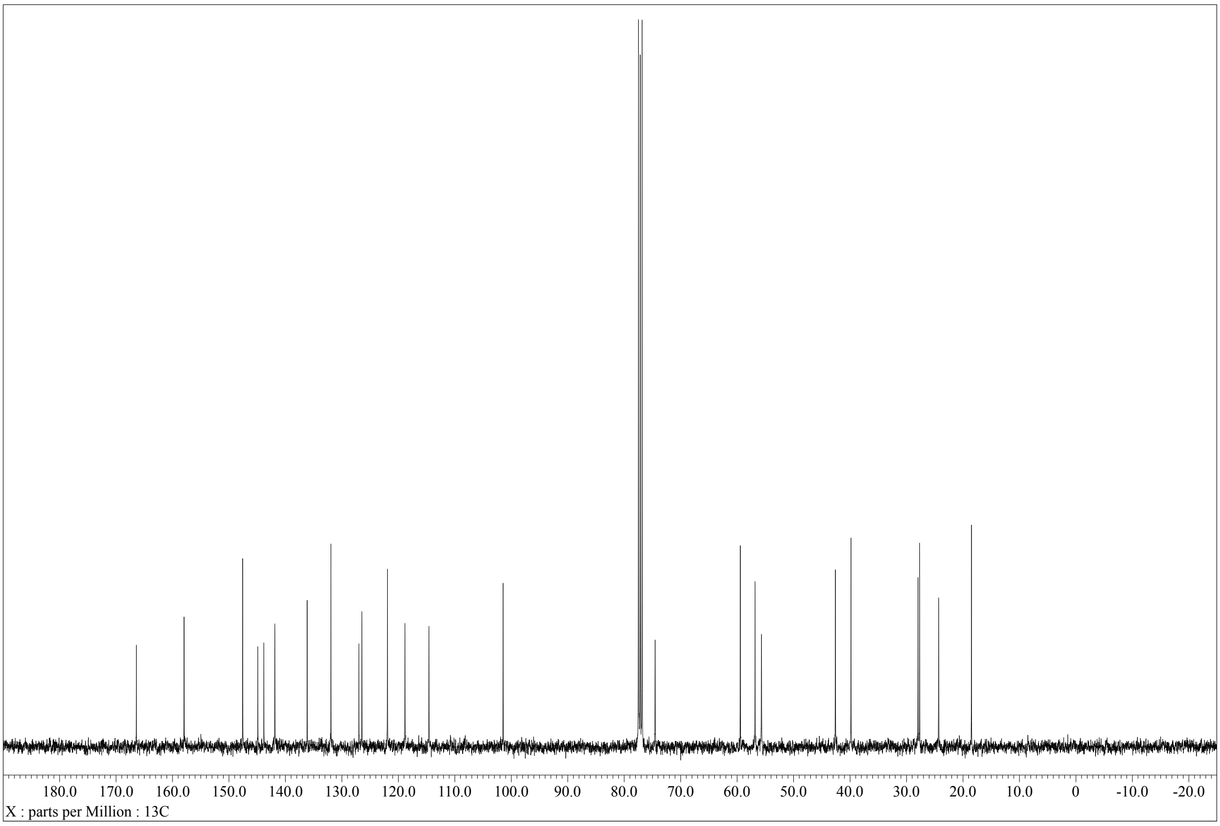

^{1}H and ^{13}C NMR spectra of Tropinyl methacrylate (7q)

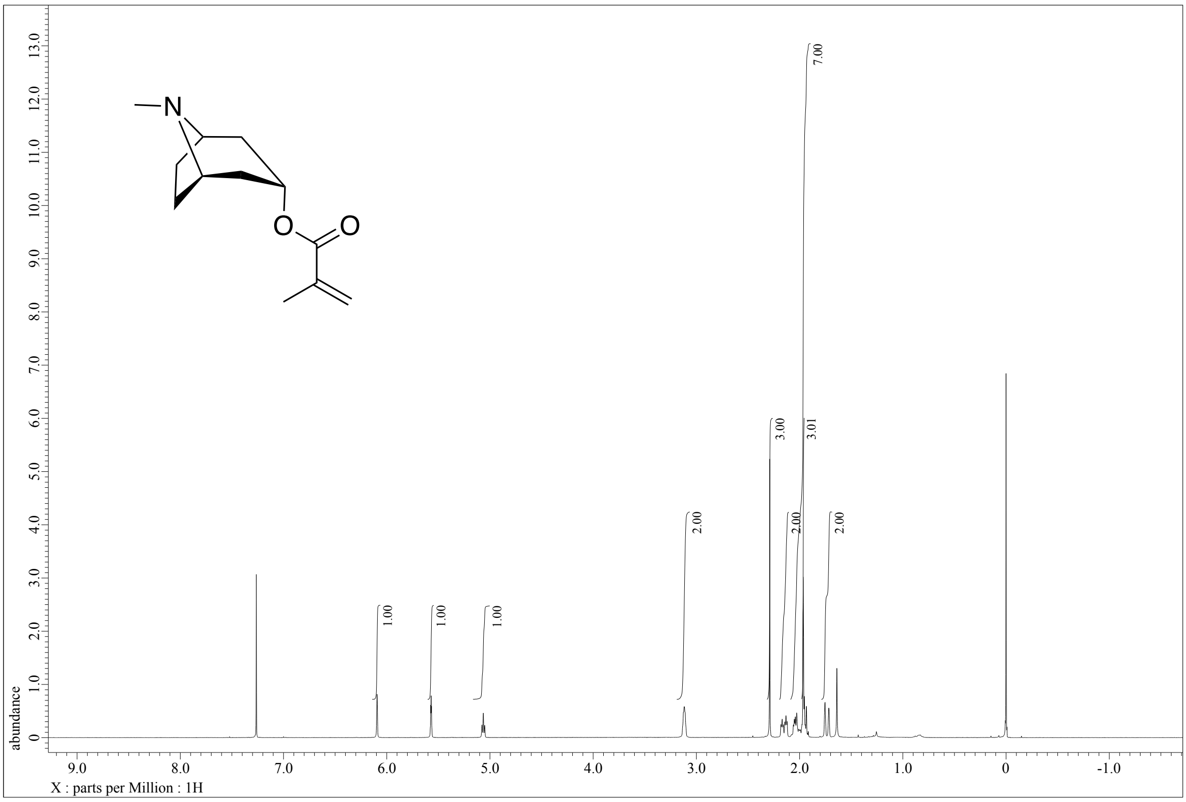

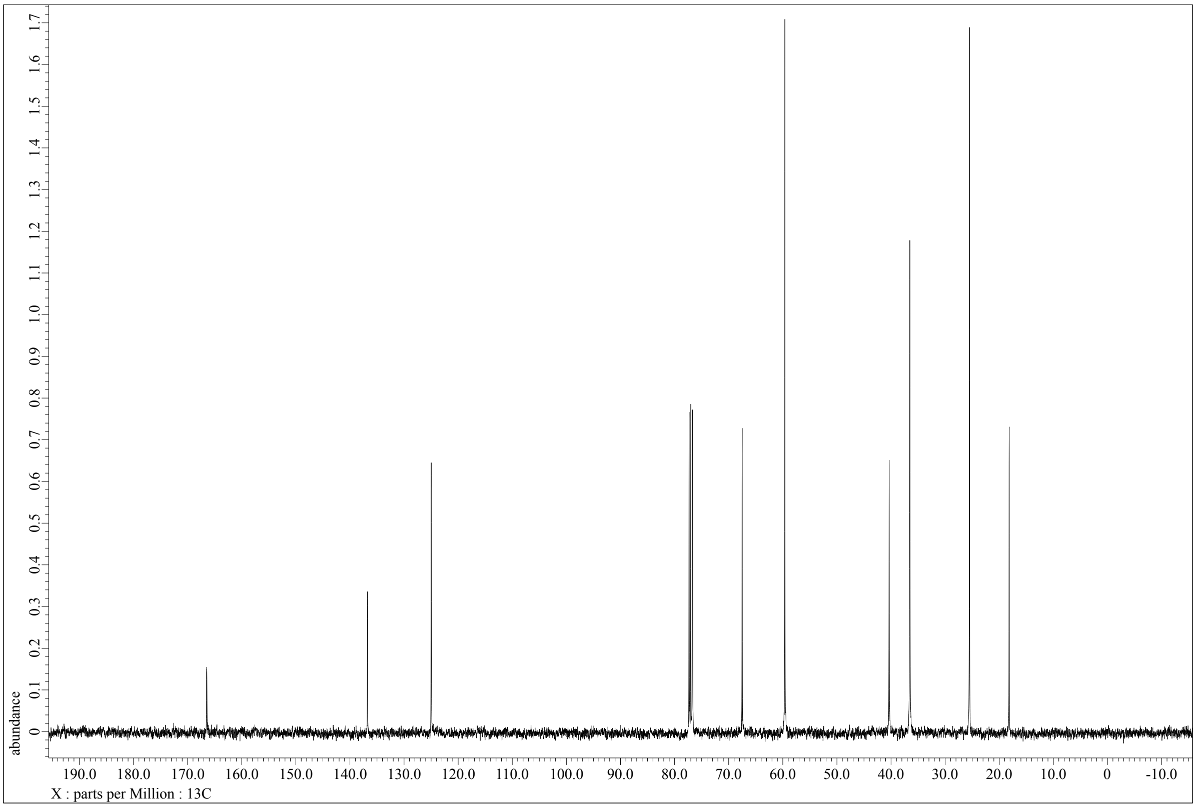

^{1}H and ^{13}C NMR spectra of 1,4-Phenylenebis(methylene) bis(2-methylacrylate) (7r)

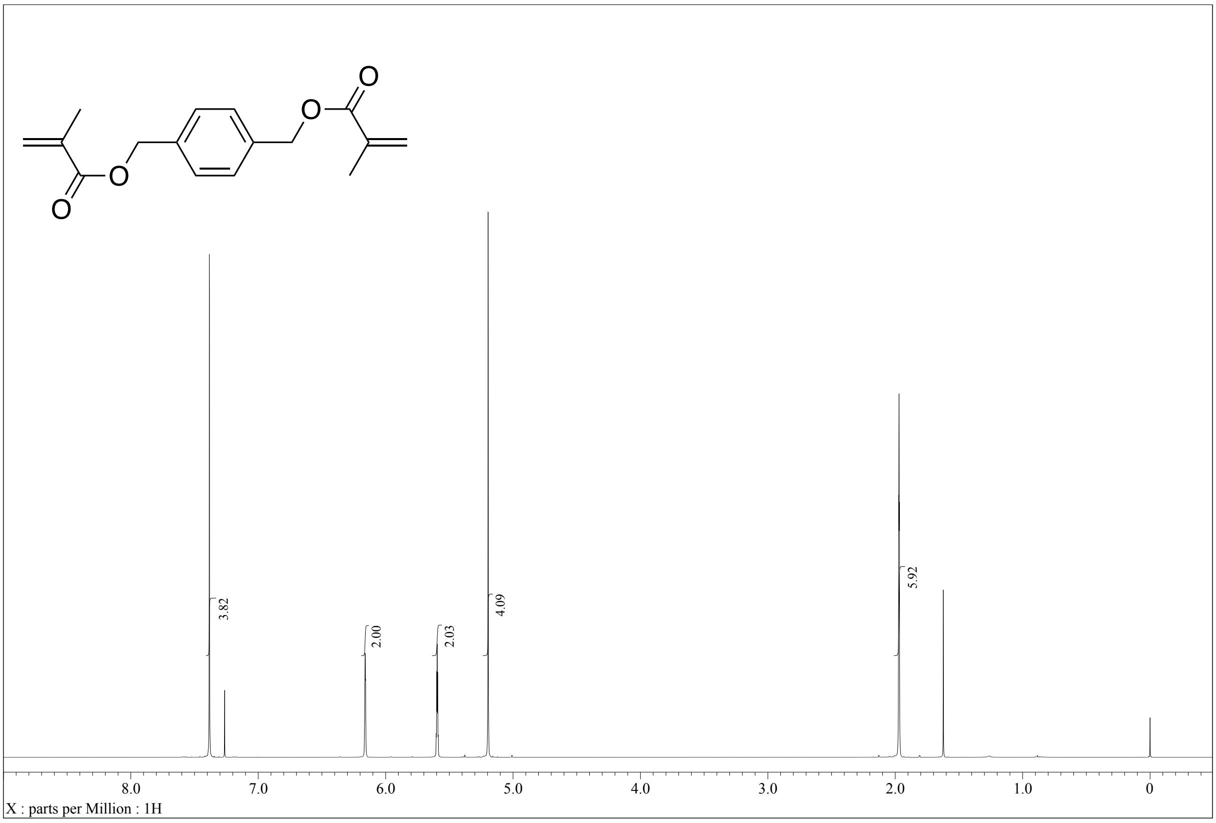

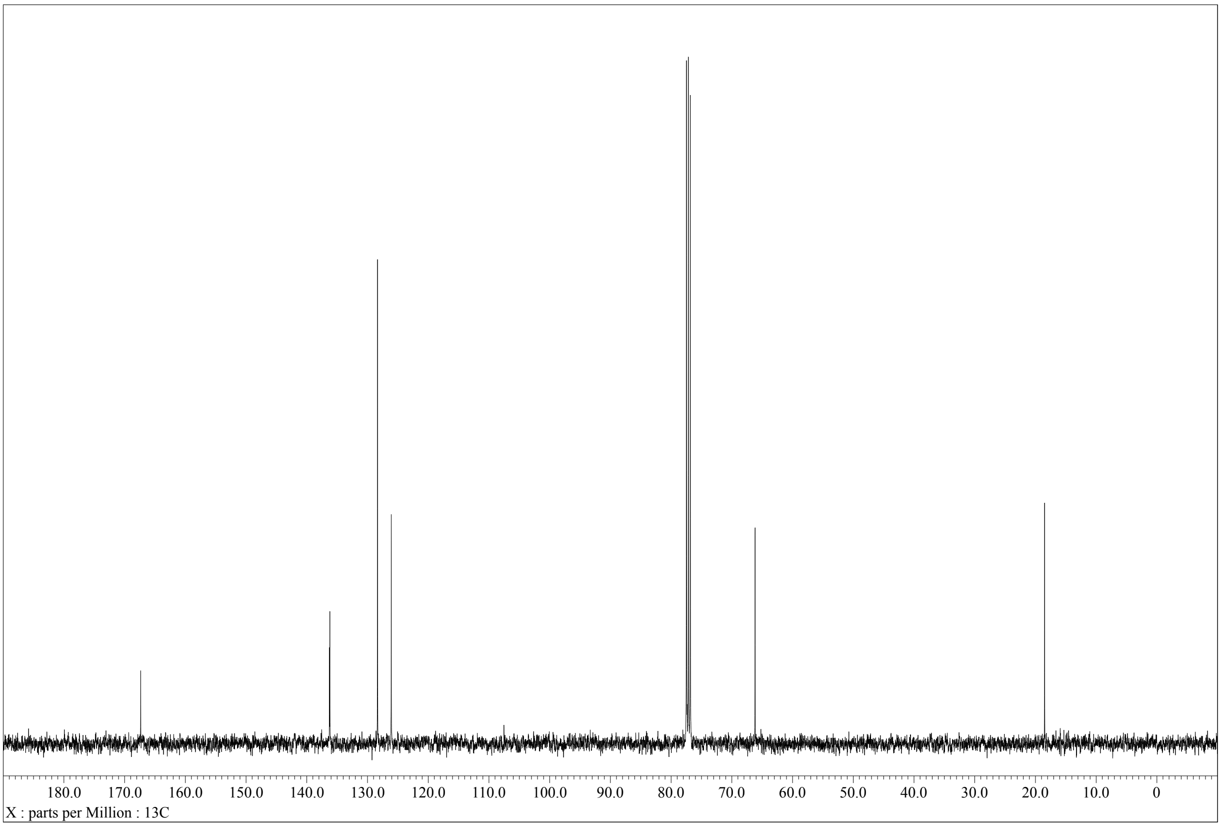

^{1}H and ^{13}C NMR spectra of Butane-1,4-diyl dimethacrylate (7i)

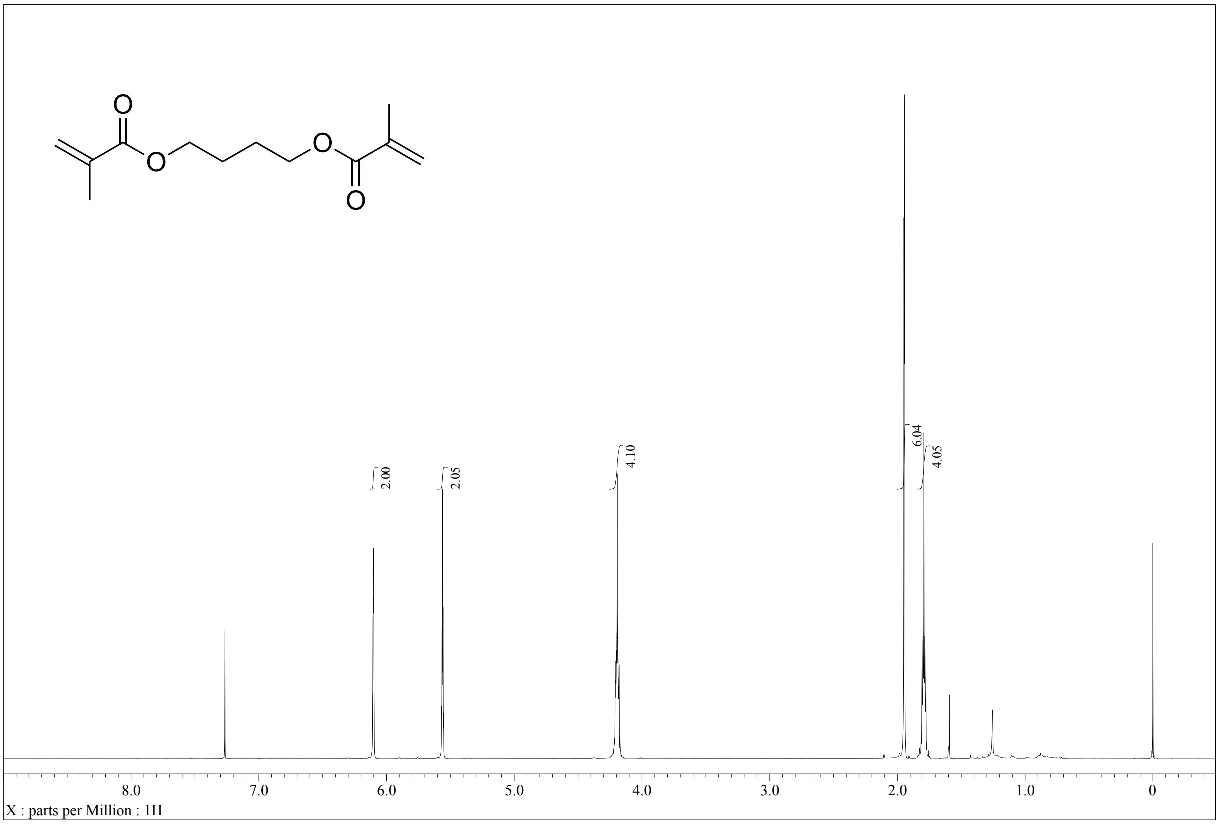

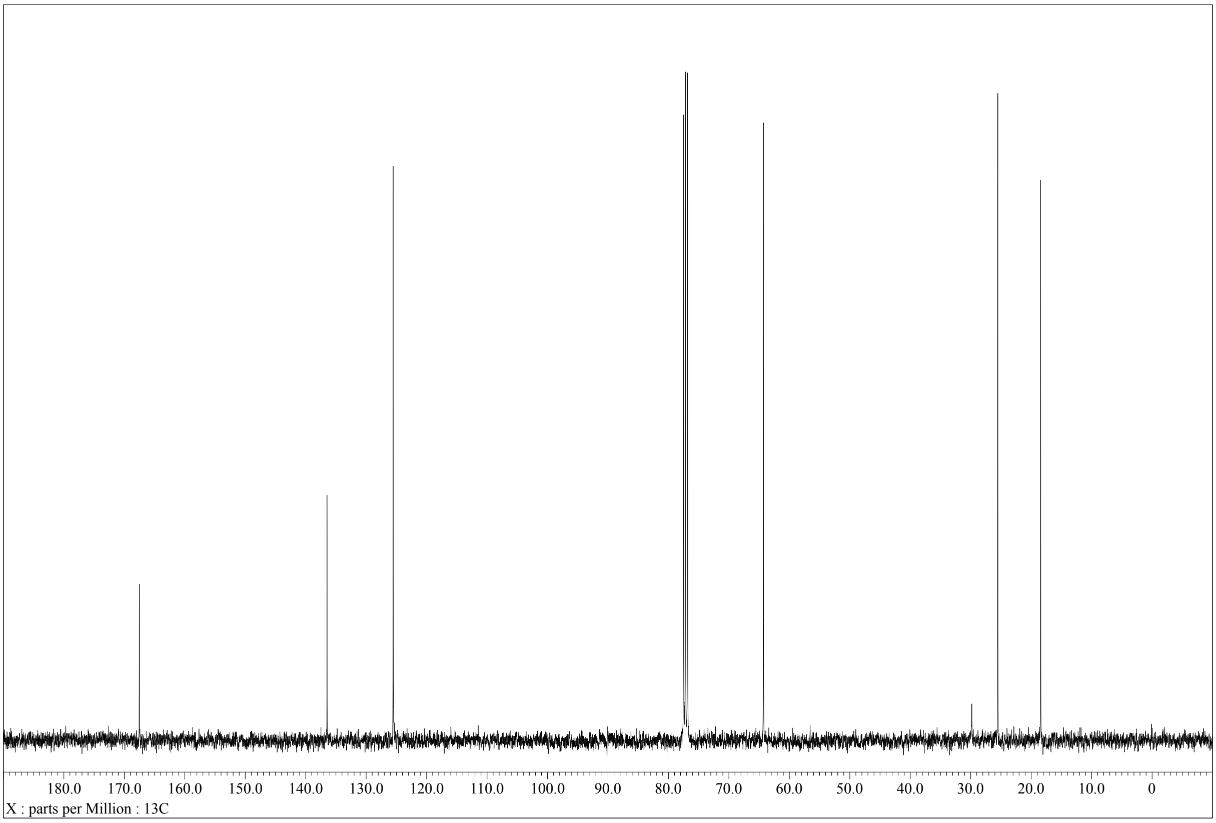

^{1}H and ^{13}C NMR spectra of (Ethane-1,2-diylbis(oxy))bis(ethane-2,1-diyl) dimethacrylate (7k)

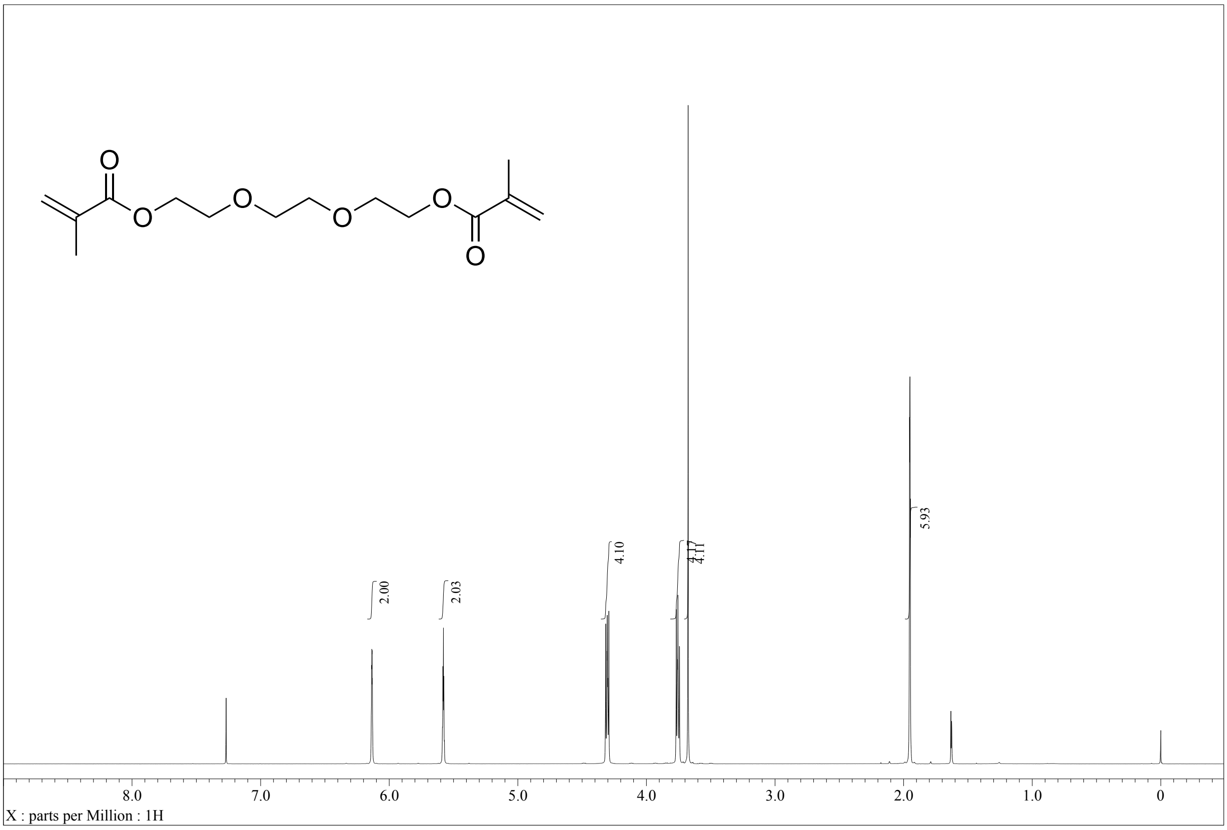

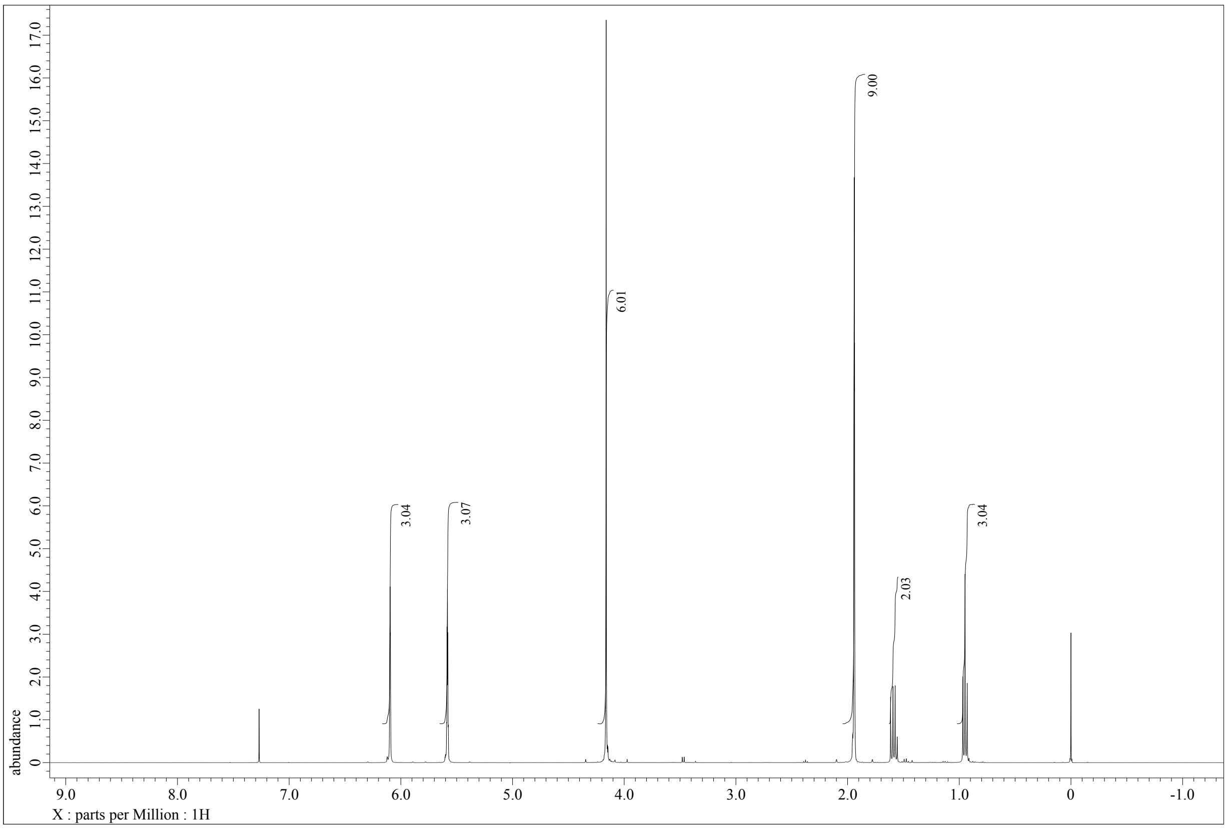

^{1}H and ^{13}C NMR spectra of 1,1,1-Trimethylolpropane trimethacrylate (7i)

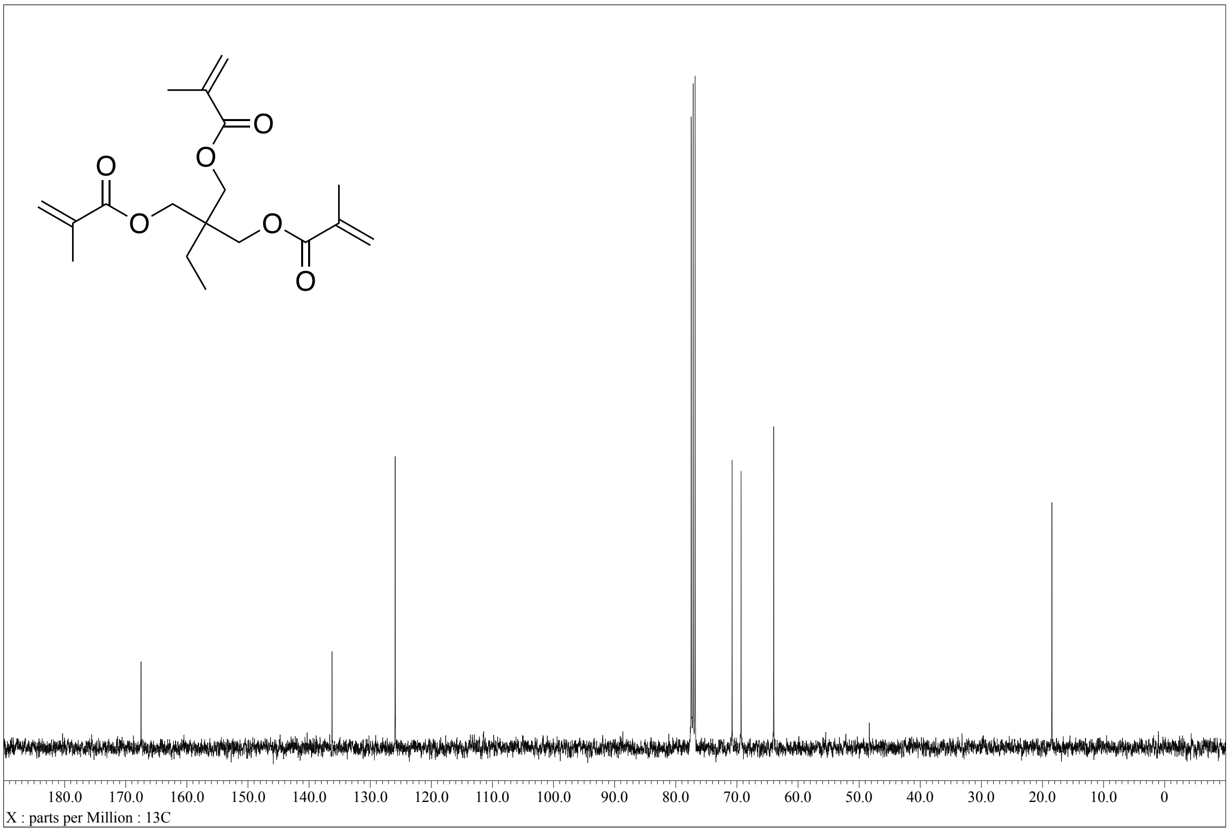

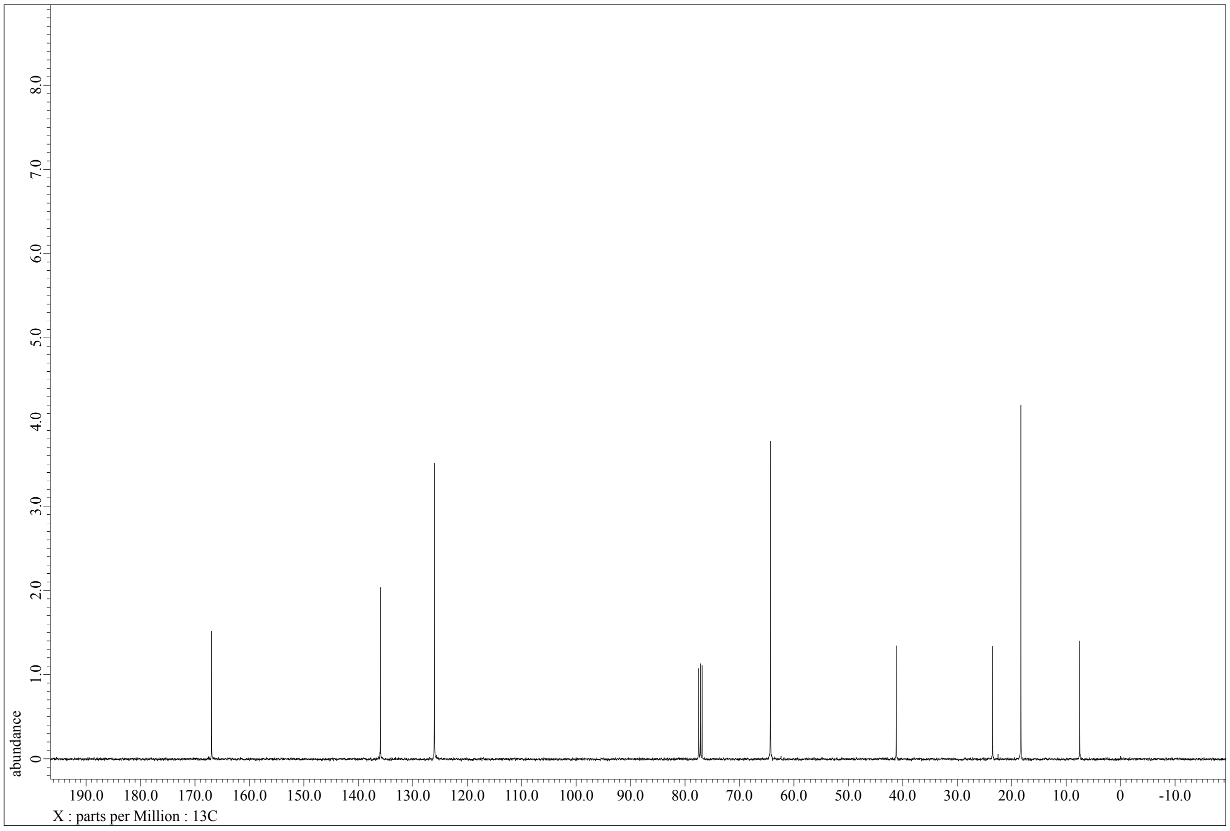

Supplement: SC-014-D2SC05413B-s001 [file SC-014-D2SC05413B-s001.pdf]
